# Engineering Pyrrolysine Systems for Genetic Code Expansion and Reprogramming

**DOI:** 10.1021/acs.chemrev.4c00243

**Published:** 2024-09-05

**Authors:** Daniel L. Dunkelmann, Jason W. Chin

**Affiliations:** † Medical Research Council Laboratory of Molecular Biology, Francis Crick Avenue, Cambridge CB2 0QH, England, United Kingdom; ‡ Max Planck Institute of Molecular Plant Physiology, Am Mühlenberg 1, 14476 Potsdam-Golm, Germany

## Abstract

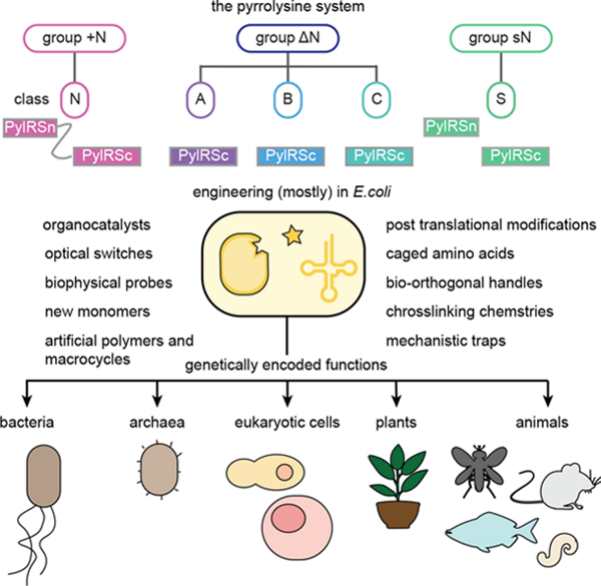

Over the past 16 years, genetic code expansion and reprogramming in living organisms has been transformed by advances that leverage the unique properties of pyrrolysyl-tRNA synthetase (PylRS)/tRNA^Pyl^ pairs. Here we summarize the discovery of the pyrrolysine system and describe the unique properties of PylRS/tRNA^Pyl^ pairs that provide a foundation for their transformational role in genetic code expansion and reprogramming. We describe the development of genetic code expansion, from *E. coli* to all domains of life, using PylRS/tRNA^Pyl^ pairs, and the development of systems that biosynthesize and incorporate ncAAs using pyl systems. We review applications that have been uniquely enabled by the development of PylRS/tRNA^Pyl^ pairs for incorporating new noncanonical amino acids (ncAAs), and strategies for engineering PylRS/tRNA^Pyl^ pairs to add noncanonical monomers, beyond α-*L*-amino acids, to the genetic code of living organisms. We review rapid progress in the discovery and scalable generation of mutually orthogonal PylRS/tRNA^Pyl^ pairs that can be directed to incorporate diverse ncAAs in response to diverse codons, and we review strategies for incorporating multiple distinct ncAAs into proteins using mutually orthogonal PylRS/tRNA^Pyl^ pairs. Finally, we review recent advances in the encoded cellular synthesis of noncanonical polymers and macrocycles and discuss future developments for PylRS/tRNA^Pyl^ pairs.

## 1 Introduction

Over the past 16 years pyrrolysyl-tRNA synthetase (PylRS)/ pyrrolysyl tRNA (tRNA^Pyl^) pairs have been extensively engineered to add noncanonical α-*L*-amino acids (ncAAs) and other noncanonical monomers (ncMs) to the genetic code of diverse organisms. Pyrrolysine (pyl) systems have been central to essentially every major development in genetic code expansion and reprogramming.^1−6^ Here we summarize the discovery of the pyl system, and its basic properties (Section 2), we then describe the key features of the initially characterized PylRS/tRNA^Pyl^ pairs for genetic code expansion (Section 3); these sections provide the necessary background on the properties of the pyl system that make it amenable to engineering. We then review the development and optimization of PylRS/tRNA^Pyl^ pairs for genetic code expansion in *E. coli*, and for genetic code expansion in prokaryotic and eukaryotic systems (Section 4), and the concerted biosynthesis and incorporation of ncAAs using PylRS/tRNA^Pyl^ pairs (Section 5). We review the scope of the ncAAs that can be site-specifically incorporated with PylRS/tRNA^Pyl^ pairs and summarize the types of applications they enable (Section 6). While genetic code expansion in living cells has, until recently, essentially been limited to α-*L*-amino acids with variant side chains or closely related α-*L*-hydroxy acids, we review recent progress in adding new classes of ncMs to the genetic code (Section 7). We then review the discovery of mutually orthogonal, triply orthogonal and quintuply orthogonal PylRS/tRNA^Pyl^ pairs from newly discovered and characterized PylRS/tRNA^Pyl^ classes (Section 8), and efforts to direct PylRS/tRNA^Pyl^ pairs to codons beyond the amber codon (Section 9). We describe progress on combining multiple engineered mutually orthogonal PylRS/tRNA^Pyl^ pairs that recognize distinct ncAAs and decode distinct codons for encoding multiple distinct ncAAs into proteins (Section 10), and recent progress on realizing genetically encoded cellular noncanonical polymer synthesis (Section 11). Finally, we describe the challenge and opportunities that may be addressed with pyl systems in the future (Section 12). While our focus is on genetic code expansion in living organisms, pyl systems have also contributed to *in vitro* code expansion^7−12^ and we mention *in vitro* work when it has directly informed *in vivo* advances.

## 2 Discovery of the Pyrrolysine System

### 2.1 Pyrrolysine in Methanogens

**Figure 1:**
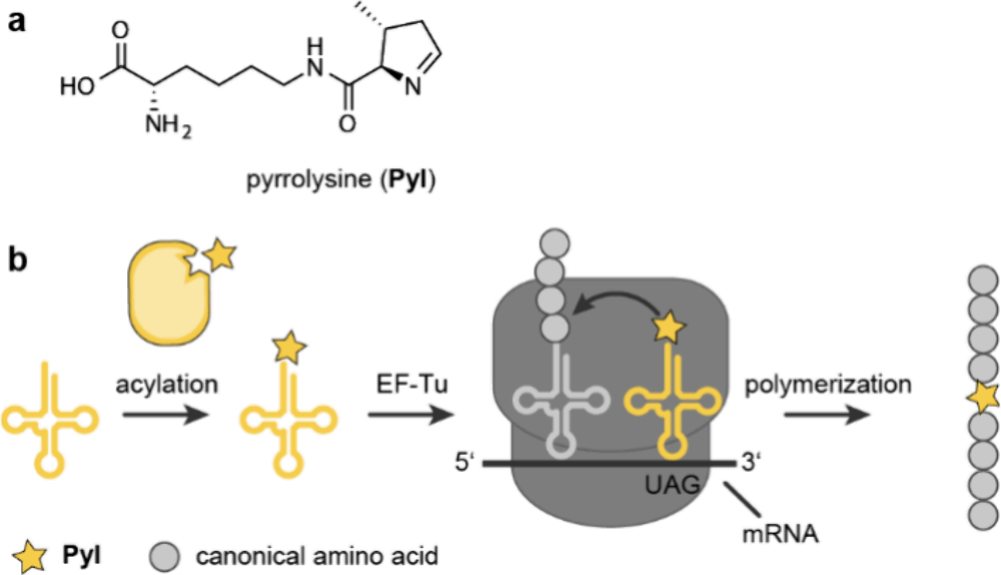
**Encoded cellular incorporation of Pyl at amber codons, via natural genetic code expansion. a**, The chemical structure of **Pyl**. **b**, The amber suppressor tRNA, tRNA^Pyl^_CUA_, is selectively charged by PylRS with **Pyl**. EF-Tu transports the aminoacylated pyl-tRNA^Pyl^_CUA_ to the ribosome, where **Pyl** is site-specifically incorporated into a protein in response to an amber stop (UAG) codon in the mRNA. Adapted from Dunkelmann et al.^19^ – copyright © The Author(s) 2024 CCBY http://creativecommons.org/licenses/by/4.0/.

The efficient read-through of an in-frame amber codon (TAG in genes, UAG in transcripts) in mono-, di- and trimethylamine methyltransferases (*mtmB*, *mtbB*, and *mttB*) in *Methanosarcina barkeri (Mb)* first pointed toward the expansion of the genetic code beyond the canonical 20 amino acids in some methanogenic archaea.^13−15^ The crystal structure of the mtmB protein of *Mb* revealed a modified lysine residue forming an ε-amide bond with a (4*R*,5*R*)-4-substituted-pyrroline-5-carboxylate, later termed pyrrolysine (**Pyl** – we use bold letters when explicitly referring to the amino acid), which was incorporated in response to an in frame amber codon (Figure 1a,b).^16^ The precise chemical composition of the 4-substituent of the pyrroline ring was not resolved in the initial crystal structure and the 4-methyl group, and the final structure of **Pyl**, was confirmed by mass spectrometry (MS).^17^ The unique chemistry of **Pyl** assists the transfer of the methyl group of mono-, di-, or trimethylamine (MMA, DMA and TMA, respectively) to the corrinoid cofactor of the corrinoid proteins MtmC, MtbC, or MttC respectively, this enables the relevant organisms to use methylamines as an energy source. Recent work has provided structural insight into the mechanism of methyl group transfer.^18^

### 2.2 PylRS/tRNA^Pyl^ Pairs Direct Incorporation of Pyl

In parallel with the discovery of **Pyl** in the crystal structure of mtmB a highly unusual tRNA gene - *pylT* - encoding a tRNA with a CUA anticodon was revealed,^20^ this gene was proximal to the *mtmB* gene in *Mb* Fusaro. The deletion of *pylT* from the genome of *Methanosarcina acetivorans* (*Mac*) rendered the archaea unable to grow on methylamine substrates.^21^*PylS*, a gene directly adjacent to *pylT*, encoded an aminoacyl-tRNA synthetase (aaRS) bearing low sequence similarity to canonical aaRS enzymes. The aaRS enzyme, termed pyrrolysyl-tRNA synthetase (PylRS), belongs to class IIc synthetases and is likely to have evolved from phenylalanyl-tRNA synthetase (PheRS). The *Mac*PylRS/*Mac*tRNA^Pyl^_CUA_ pair, when expressed in *E. coli* containing *MtmB202TAG*, led to **Pyl**-dependent synthesis of full length MtmB containing **Pyl** at position 202, as judged by tandem mass spectrometry (MS/MS) of a tryptic fragment; this provided evidence that *Mac*PylRS aminoacylates *Mac*tRNA^Pyl^_CUA_ with **Pyl** to enable the cotranslational incorporation of this amino acid in response to the amber codon (Figure 1b).^22^

### 2.3 Pyl Biosynthesis

**Figure 2:**
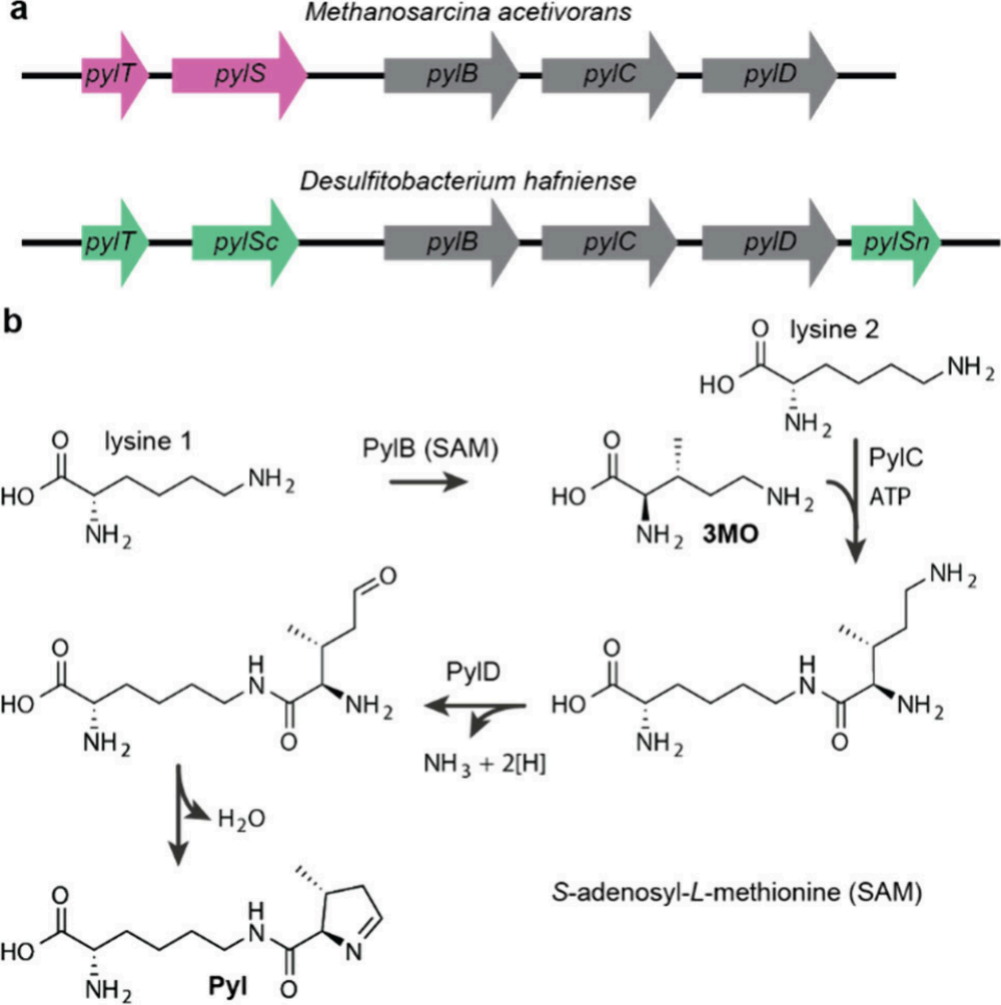
**Pyl biosynthesis is mediated by***pylBCD***. a**, Operon structure of the gene cluster *pylTSBCD* in the archaeon *Methanosarcina acetivorans* and the bacterium *Desulfitobacterium hafniense*. **b**, Biosynthetic pathway of **Pyl** from two molecules of lysine mediated by pylBCD. First, the radical *S*-adenosyl methionine (SAM) enzyme PylB converts lysine into (3*R*)-3-methyl-*D*-ornithine (**3MO**). Subsequently PylC ligates a second lysine to **3MO** and PylD oxidizes the terminal amine of the conjugate to an aldehyde. The pyrroline ring is then spontaneously formed by a condensation reaction.

The gene cluster encoding *mtmB*, *mtbB*, *mttB*, and the genes for the *Mb*PylRS/*Mb*tRNA^Pyl^_CUA_ pair in *Mb* Fusaro also contains *pylB*, *pylC*, and *pylD*. The sequence similarity of these genes to known metabolic enzymes directly indicated a role in the biosynthesis of **Pyl**. Interestingly, the phylogenetically distant Gram-positive bacterium *Desulfitobacterium hafniense* (*Dh*), the genome sequence of which was analyzed after the identification of an *mtmB* gene with an in frame amber codon, encodes homologous genes to *pylT* and *pylS* together with *pylB*, *pylC*, and *pylD* (Figure 2a).^20^ The occurrence of the same gene cluster in distantly related organisms suggested that the *pylTSBCD* gene cluster is a self-contained biosynthetic pathway, transferred through horizontal gene transfer, that directs the incorporation of **Pyl** in response to an amber codon. Indeed, expression of *Mac pylTSBCD* in *E. coli* resulted in the genetic encoding of **Pyl** in response to amber codons.^23^

The biosynthetic pathway of **Pyl** (Figure 2b) was elucidated by stable isotopic labeling, MS, and genetics of *E. coli* cells transformed with variants of the *pylTSBCD* operon from *Mac* and the *Mb* gene *mtmB*. Two molecules of lysine were found to be the precursor of **Pyl**.^24^ These mechanistic studies elucidated the function of PylC and PylD and provided hints toward the function of PylB; the mechanism of PylB was confirmed in subsequent experiments.^25^ In the pathway, PylB, which is a radical iron–sulfur-*S*-adenosyl-*L*-methionine protein, converts lysine into 3-methyl-*D*-ornithine (**3MO**) through a rearrangement of the carbon backbone; this is consistent with prior work showing that addition of *D*-ornithine (*D***O**) to *E. coli* cells harboring *pylTSBCD* boosted amber suppression efficiency 7-fold.^26^ PylC, a member of the carbamoyl phosphate synthetase family, ligates **3MO** to the ε-nitrogen of lysine forming an amide bond in an ATP-dependent manner. Finally, PylD, a nicotinamide adenine dinucleotide dependent dehydrogenase, oxidizes the δ-amine of the **3MO** residue of 3-methyl-*D*-ornithyl-*N*^ε^-*L*-lysine and catalyzes the ring closure at the C-5 position of the 3-methyl-*D*-ornithyl group by dehydrogenation. Crystal structures of all three enzymes have given further insights into the mechanism of **Pyl** biosynthesis.^27,28^ Additional experiments have shown that the pyl biosynthetic pathway can function in all domains of life.^29^ Interestingly, in *Acetohalobium arabaticum* the expression of *pylTSBCD*, and therefore the incorporation of **Pyl** in response to amber codons, is regulated by the growth condition of the bacterium.^30^ When grown on pyruvate, in the absence of TMA, the genes in *pylTSBCD* are not sufficiently expressed and amber codons are not suppressed. However, when TMA is added to the growth medium the pyl pathway is expressed and **Pyl** incorporated into mttB and other proteins.

The genes of the *pylTSBCD* operon were initially believed to be stereotypically ordered in archaea, with *pylT* being followed by *pylS*, *pylB*, *pylC*, and *pylD*, and in a more variable, but compact, configuration in bacteria.^31^ Furthermore, only rare examples of organisms harboring the full pyl system together with genes other than *mtmB*, *mtbB*, and *mttB* with in-frame amber codons have been reported.^32,33^ Recent data from culture and metagenomic approaches hint at much wider distribution of PylRS/tRNA^Pyl^ pairs in archaea and an astonishing sequence diversity within the isoacceptor class.^34−42^

## 3 PylRS/tRNA^Pyl^Pairs

Pyrrolysine systems were initially identified from select methanogenic archaea from the order of *Methanosarcinales*, and from certain methanogenic bacteria. However, culture and metagenomic based approaches have provided a basis for substantially expanding our understanding of pyl system diversity.^35−39^ Known PylRS enzymes display three distinct architectures. In the first architecture, the C-terminal domain (PylRSc) is covalently connected via a flexible linker to a highly basic, yet hydrophobic, N-terminal domain (PylRSn). In the second architecture–initially described for bacterial PylRS systems, but now also identified in archaeal genomes–the PylRSn and PylRSc are expressed as separate polypeptides. In the third architecture–found in *Methanomassiliicoccales* and other archaea–no N-terminal domain has been identified, and the protein functions as a stand-alone C-terminal domain.^35,36,38−40,42^

In this section we introduce the nomenclature used throughout this review for PylRS/tRNA^Pyl^ pairs (Section 3.1) and then describe insights into the intrinsic properties of PylRS/tRNA^Pyl^ pairs derived from the characterization of initially discovered systems. Most of this work focused on the archaeal systems from *Mb*, and *Methanosarcina mazei* (*Mm*) and the bacterial system from *Dh*. The insights into the active site of the C-terminal catalytic domain in these PylRS enzymes (Section 3.2) appear to broadly translate to other PylRS systems that have been investigated. Studies on the N-terminal domain of PylRS enzymes (Section 3.3) provide information about systems containing this domain. Detailed insights into the interface between PylRS and tRNA^Pyl^ (Section 3.4) are likely to be system specific, but the overall compact structure of tRNA^Pyl^ and the topology of the complexes is thought to be a general feature of pyl systems. The observation that the active site of PylRS enzymes can accommodate **Pyl** analogs (Section 3.5) seems to hold for other pyl systems tested. The observation that PylRS enzymes do not recognize the anticodon of tRNA^Pyl^ (Section 3.6) also appears to extend to other pyl systems where this has been tested.

### 3.1 Nomenclature of PylRS/tRNA^Pyl^ Pairs

**Figure 3:**
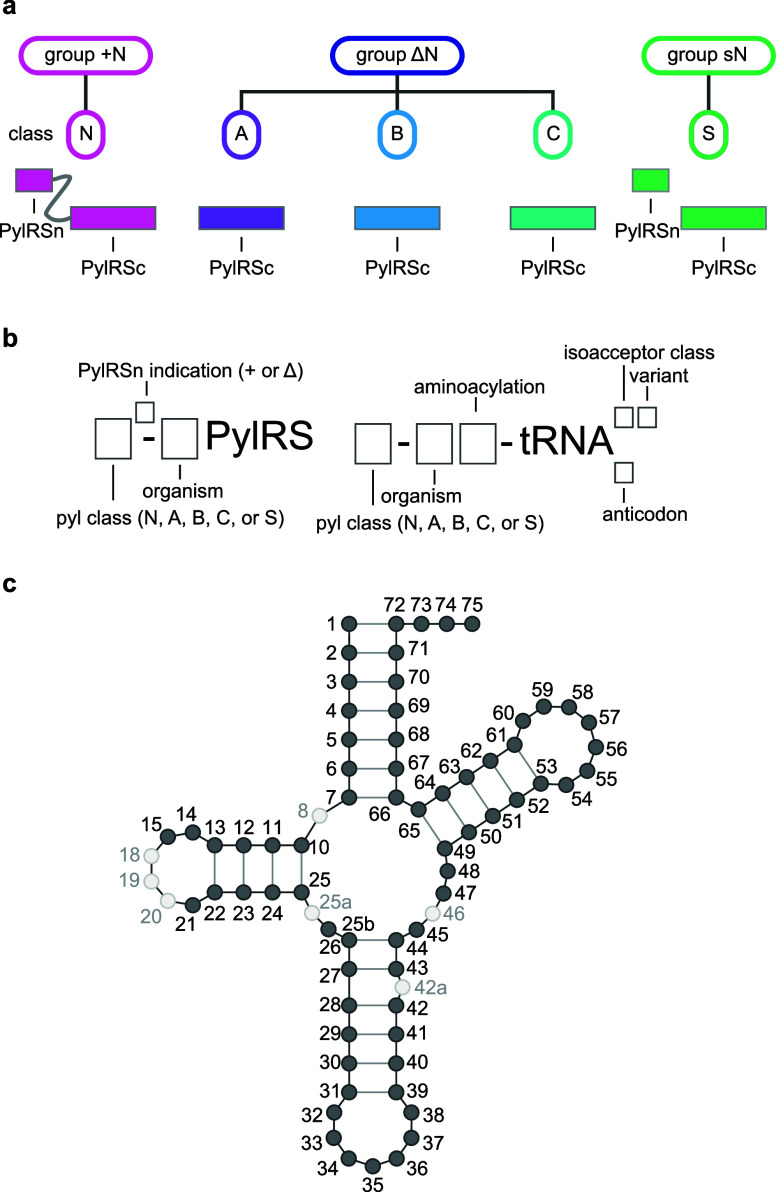
**PylRS/tRNA**^**Pyl**^**pair nomenclature. a**, Division of PylRS/tRNA^Pyl^ pairs into three groups and five classes. The groups are defined by the architecture of the PylRS enzyme. The + N group contains PylRS enzymes where PylRSn and PylRSc are covalently connected by a flexible linker, the Δ*g*roup is comprised of PylRS enzymes lacking PylRSn in their host genome, and the sN group is composed of PylRS enzymes where PylRSn and PylRSc are produced *in trans* from distinct genes. The classes (N, A, B, C, and S) represent a finer subdivision of the pyl system based on sequence identity clustering of PylRS-, and tRNA^Pyl^ sequences and the aminoacylation specificity of the PylRS/tRNA^Pyl^ pairs with respect to each other. **b**, Nomenclature used for PylRS enzymes and pyl tRNAs in this review. The tRNA nomenclature is in line with International Union of Pure and Applied Chemistry (IUPAC) rules and extended to include pyl class information, as well as tRNA^Pyl^ variant information. We note that when referring to tRNA^Pyl^ in plural, we write pyl tRNAs, in accordance with IUPAC rules. **c**, Numbering of residues in tRNA^Pyl^. The numbering is in line with the general convention for tRNAs according to Sprinzl et al.^43^ However, some common nucleotides are missing in pyl tRNAs (9, 16, 17, 18), and some unusual nucleotides are present (25a, 25b, 42a). Nucleotides in dark gray are present in all described pyl tRNAs, nucleotides in light gray are present in some pyl tRNAs.

For clarity we use the following nomenclature to accurately, and unambiguously describe PylRS enzymes and pyl tRNAs. First, we subdivide the PylRS/tRNA^Pyl^ pairs into groups (Figure 3a). Three major groups can be defined based on the architecture of the pyl system: the + N group (where PylRSn is covalently linked to PylRSc), the ΔN group (which lacks the PylRSn), and the sN group (where PylRSn and PylRSc are expressed *in trans* from separate genes). The definition of groups is solely based on the architecture, inferred from genomic sequence, of the PylRS enzyme. We then define five pyl classes (A, B, C, N and S) based on the sequence and function of a PylRS/tRNA^Pyl^ pair; the five PylRS/tRNA^Pyl^ classes are discussed in detail in Section 8 (Figure 3a). When referring to a PylRS enzyme we use the letter of the class and specify if PylRSc is present in combination with PylRSn (*in trans*, or covalently linked) by adding the suffix ‘^+^’, or lacks PylRSn by adding the suffix “^Δ^” after the letter assigning the class. As an example, *Mm*PylRS is a member of class N and is referred to as N^+^-*Mm*PylRS, whereas its tRNA is referred to as N-*Mm*tRNA^Pyl^ (Figure 3b). *Dh*PylRS is a member of class S, and if *Dh*PylRSc is expressed in the presence of the *Dh*PylRSn we refer to it as S^+^-*Dh*PylRS and if it is expressed in absence of *Dh*PylRSn we use S^Δ^-*Dh*PylRS, the cognate tRNA^Pyl^ is referred to as S-*Dh*tRNA^Pyl^. We refer to all distinct tRNA mutants by adding a descriptive suffix after the suffix “^Pyl^”. Furthermore, we refer to cognate interactions as those between PylRS/tRNA^Pyl^ pairs of the same class, and noncognate interactions as those between PylRS/tRNA^Pyl^ pairs of distinct classes. Finally, when referring to the nucleotide number in pyl tRNAs we use the numbering system outlined (Figure 3c).

### 3.2 The C-Terminal Catalytic Domain of PylRS Enzymes

The C-terminal domain of N^+^-PylRS and S^+^-PylRS contains all the sequence motifs that define the catalytic domains of class IIc aaRS enzymes. Insight into the structure of PylRS resulted from the X-ray crystal structure of the C-terminal catalytic domain of N^+^-*Mm*PylRS (N-*Mm*PylRSc270, residues 185–454).^44,45^ As expected from sequence homology, N-*Mm*PylRSc270 closely resembles the structure of other class II synthetases in which a β-sheet core is surrounded by several long helices.^46^ All three sequence motifs of class II synthetases are present; sequence motif 1 is responsible for the dimerization of the N^+^-*Mm*PylRS and sequence motifs 2 and 3 build the nucleotide interface.^47^ The apo structure of S-*Dh*PylRSc is very similar to that of the C-terminal domain of the archaeal PylRS.^48^ Structures of additional PylRS systems have revealed broadly similar active site structures.^7,9,49−59^ Phylogenetic and structural analysis suggests that PylRS enzymes arose from PheRS – by gene duplication and neo-functionalization–before the last universal common ancestor (LUCA).^42,45^

The crystal structures of the catalytic domain of N^+^-*Mm*PylRS (N-*Mm*PylRSc270) alone, and in complex with (i) the nonhydrolyzable adenosine triphosphate (ATP) analog adenylyl imidodiphosphate (AMP-PNP), (ii) the **Pyl** analog *N*^ε^-((cyclopentyloxy)carbonyl)-*L*-lysine (**CycK**) with ATP and, (iii) **Pyl**-AMP, provided structural insights into substrate-binding in the catalytic domain.^44,45^ A notable feature of these structures is the deep hydrophobic pocket in which the amino acid substrate is bound.

**Figure 4:**
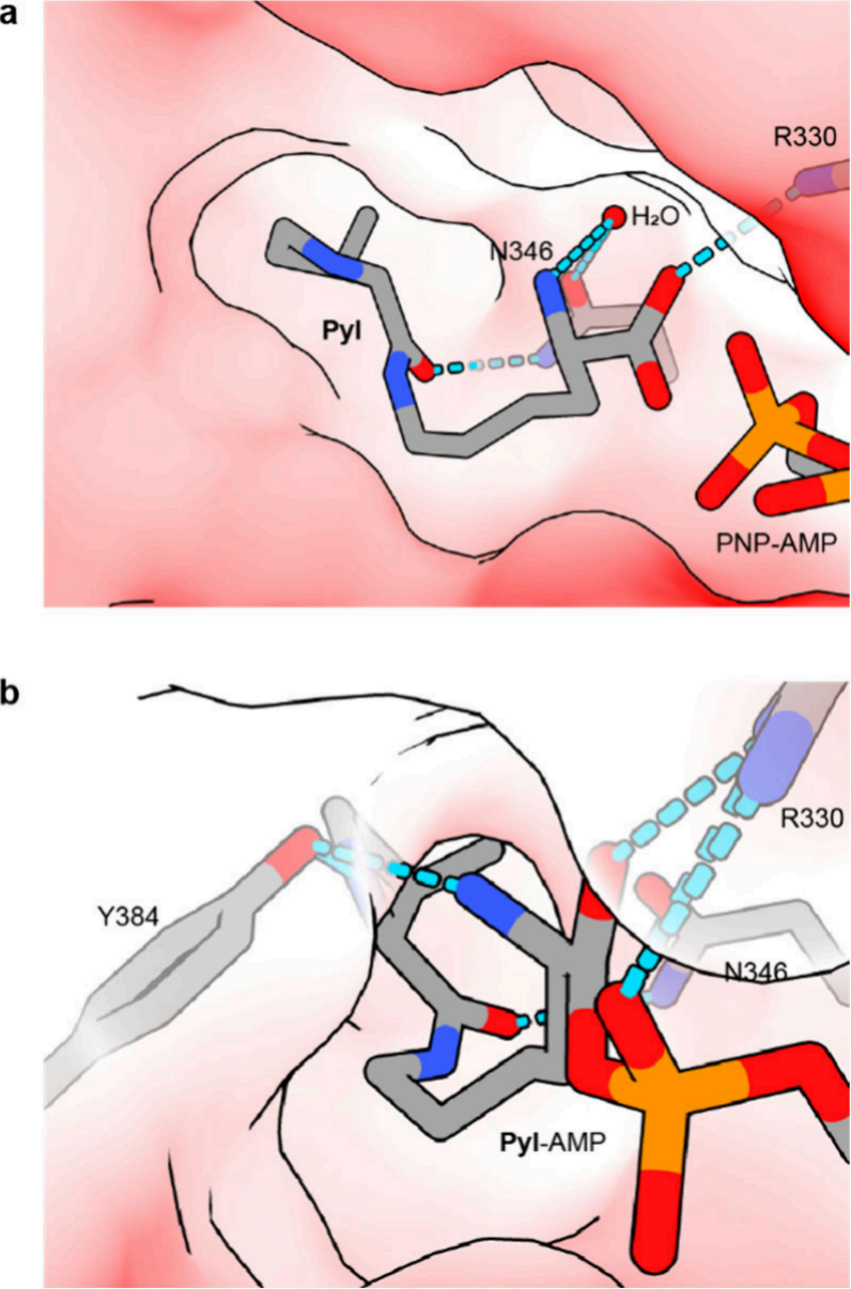
**Pyl PNP-AMP and Pyl-AMP binding in the active site of N**^**+**^**-***Mm***PylRS. a**, Binding of **Pyl** and PNP-AMP in the deep hydrophobic pocket of the active site of N-*Mm*PylRSc (PDB 2ZCE).^59^ Direct hydrogen bonds are formed between the primary (backbone) carbonyl of **Pyl** and R330 as well as the secondary carbonyl (side chain) of **Pyl** and N346. The α-amine forms a hydrogen bond with a coordinated water molecule. **Pyl** and the interaction partners are shown as sticks representation, N-*Mm*PylRSc is shown as a transparent electrostatic surface (red negatively charged, white noncharged, blue positively charged). **b**, Recognition of **Pyl**-AMP by N-*Mm*PylRSc (2Q7H).^45^**Pyl**-AMP forms the same direct hydrogen bonding network with N-*Mm*PylRSc as observed for **Pyl** in the structure shown in panel **a** with an additional hydrogen bond being formed between the α-amine of **Pyl** and Y384. Y384 is part of a flexible loop which closes the active site and was not visible in the crystal structure depicted in panel **a**. **Pyl**-AMP and the interacting amino acids within PylRS are shown in stick representation, PylRS is shown as a transparent electrostatic surface (red negatively charged, white noncharged, blue positively charged).

A comparison of the crystal structures of the catalytic domain of N^+^-*Mm*PylRS in its apo form and in complex with AMP-PNP, suggests that ATP binding leads to substantial changes in the architecture of N^+^-*Mm*PylRS.^59^ In contrast, the structure of the backbone of N-*Mm*PylRSc only changes minimally between the three structures with bound substrates. Two direct hydrogen bonds are formed with the amino acid substrates: R330 forms a hydrogen bond with the primary (backbone) carbonyl, and N346 forms a hydrogen bond with the secondary (side chain) carbonyl. Besides these two hydrogen bonds, the positioning of the substrate is mainly mediated through nondirected van der Waals interactions (Figure 4a).^45,59^ The relaxed substrate recognition could stem from the absence of **Pyl**-like metabolites in methanogenic bacteria and archaea, limiting the evolutionary pressure for a tight active site fit of the substrate. In accordance with this hypothesis, PylRS enzymes do not contain a substrate editing domain.

Interestingly, the α-amine of the amino acid substrate is not involved in a direct hydrogen-bonding network with essential residues in N^+^-*Mm*PylRS^+^. Although the hydroxy group of Y384 is involved in a hydrogen bond with the α-amine in the crystal structure of the complex of N-*Mm*PylRSc270 and **Pyl**-AMP (Figure 4b),^45^ a Y384F mutation did not impede the reactivity of N^+^-*Mm*PylRS.^58^ On the contrary, the Y384F mutation enhanced N^+^-*Mm*PylRS activity.^58,60^ The absence of α-amine binding by PylRS is in stark contrast to its structurally closely related homologue PheRS, which forms a tight hydrogen-bonding network with the α-amine.^61^^62^

### 3.3 The N-Terminal Domain of PylRS Binds tRNA^Pyl^

When discovered, the N-terminal domain of PylRS bore no significant sequence similarity to known-families of RNA-binding proteins and its function was unclear.^63^ Early studies suggested that the archaeal N^+^-*Mb*PylRS enzyme required the N-terminal domain to support amber suppression activity *in vivo*, as several N-terminal truncations of N^+^-*Mb*PylRS did not produce measurable amber suppression when paired with its tRNA^Pyl^ in *E. coli*.^64^ However, it is unclear whether increasing the levels of N-*Mb*PylRSc would have led to *in vivo* activity in these assays. Interestingly, N-*Mm*PylRSc retained some *in vitro* acylation activity.^59^

S^Δ^-*Dh*PylRS (the C-terminal domain of S^+^-*Dh*PylRS) also displayed some activity *in vitro* in the absence of its N-terminal domain (S-*Dh*PylRSn). Initial observations pointed toward low activity of S^+^-*Dh*PylRS *in vivo* in *E. coli* when tested with the large **Pyl** analog **CycK** (activity was only detectable with a highly sensitive genetic assay).^57^ However, when assayed with the smaller **Pyl** analog, *N*^ε^-((allyloxy)carbonyl)-*L*-lysine (**AllocK**), S^Δ^-*Dh*PylRS alone led to enhanced amber suppression in *E. coli*. The preference of S^+^-*Dh*PylRS for smaller **Pyl** analogues is consistent with it having a smaller substrate binding pocket, than archaeal N^+^-PylRS enzymes.^65^

The N-terminal domains of archaeal and bacterial PylRS enzymes bind tRNA^Pyl,^ and the affinity of S-*Dh*PylRSn for S-*Dh*tRNA^Pyl^ was at least an order of magnitude higher than the affinity of S^Δ^-*Dh*PylRS for S-*Dh*tRNA^Pyl^.^63,64^ A mutational screen of S-*Dh*tRNA^Pyl^ suggested that S-*Dh*PylRSn bound the D- and T-stem as well as the T- and variable loops of S-*Dh*tRNA^Pyl^_._^63^ Our understanding of N-terminal domain binding has been augmented by a crystal structure of the N-terminal domain of N^+^-*Mm*PylRS bound to N-*Mm*tRNA^Pyl^ (see Section 3.4).^60^ Additional experiments suggested that the N-terminal domain of PylRS system can contribute to tRNA^Pyl^ specificity as well as affinity.^40^

These studies established PylRSn as a previously unknown RNA binding domain that increases the affinity, and may alter the specificity, of certain PylRS systems for tRNA^Pyl^. It remains unclear whether there is allostery between the C-terminal domain and N-terminal domain in the catalytic cycle for aminoacylation.

### 3.4 PylRS Form Unique Interfaces with tRNA^Pyl^

**Figure 5:**
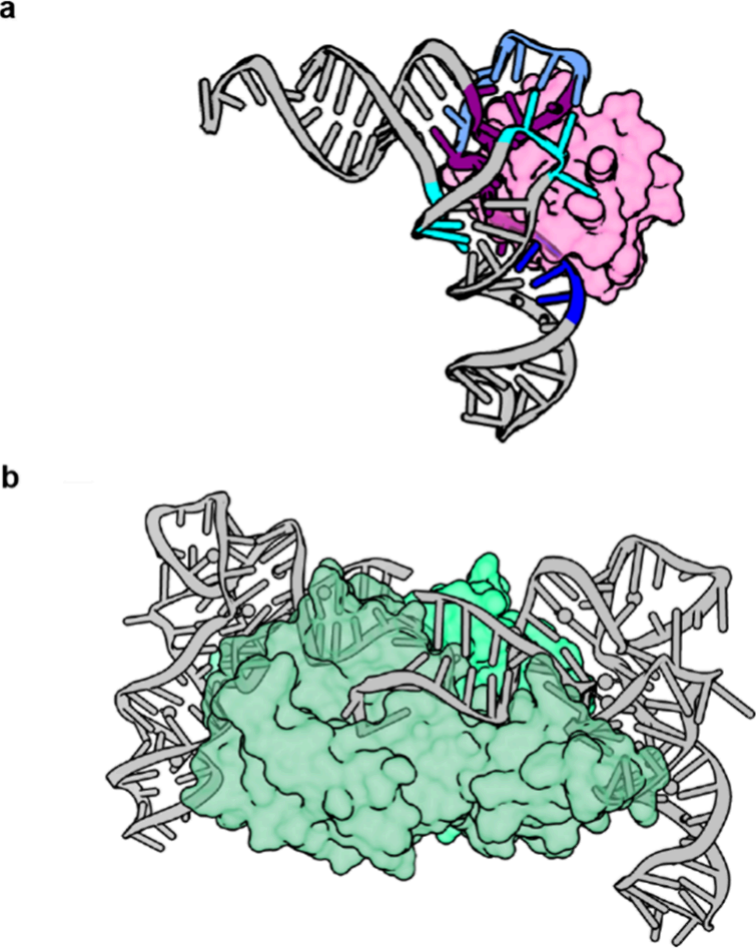
**The PylRS:tRNA**^**Pyl**^**binding interface. a**, Crystal structure of N-*Mm*PylRSn bound to N-*Mm*tRNA^Pyl^ (PDB 5UD5).^60^ N-*Mm*PylRSn interacts with the variable loop (dark blue), D-stem (cyan), T-loop (purple), and T-stem (light blue) of N-*Mm*tRNA^Pyl^. **b**, Crystal structure of S^Δ^-*Dh*PylRS in complex with S-*Dh*tRNA^Pyl^ (PDB 2ZNI).^57^ S^Δ^-*Dh*PylRS forms a dimer in the crystal structure and *in vivo* where each protomer (colored in two shades of green) predominantly interacts with one tRNA^Pyl^, while forming some interactions with the second tRNA^Pyl^.

N-*Mm*tRNA^Pyl^ and S-*Dh*tRNA^Pyl^ form canonical cloverleaf secondary structures. They also form L-shaped tertiary conformations and, like canonical tRNAs, are likely to interact with elongation factor thermo unstable (EF-Tu) in the translation cycle.^63,66,67^ As with all tRNAs, the tertiary core of tRNA^Pyl^ is formed by the interaction between nucleotides, in the D- and T- loops (Figure 5a). However, several unique features–mainly a short variable and D- loop (three and five nucleotides, respectively) – result in the tRNA^Pyl^ core being exceptionally compact.^57^ Furthermore, unusually few nucleotide modifications (4-thiouridine at position 8 and 1-methyl-pseudouridine at position 50) have been identified in tRNA^Pyl^ to date.^68^

S^Δ^-*Dh*PylRS forms a dimer in the crystal structure and in solution.^57^ In the crystal structure, the asymmetric unit contains two S^Δ^-*Dh*PylRS and two S-*Dh*tRNA^Pyl^ molecules (Figure 5b). Although each tRNA^Pyl^ predominantly interacts with one protomer, the synthetase dimer forms a concave structure that complements the shape of the acceptor helix and directs the 3′ end of the tRNA to the catalytic site.

As with all class II synthetases,^69^ S^Δ^-*Dh*PylRS approaches S-*Dh*tRNA^Pyl^ from the major groove. However, it has evolved several distinctive features to recognize the unusual shape of its cognate S-*Dh*tRNA^Pyl^. For instance, G8 of S-*Dh*tRNA^Pyl^, which is idiosyncratically flipped out of the tRNA body, is accommodated by a unique cation-π interaction from R140 of the synthetase. Moreover, the tRNA-binding domain 1, which is exclusive to PylRS enzymes, makes conserved specific interactions with the compact tertiary core of tRNA^Pyl^. As a result, tRNAs from other iso-acceptor classes, which possess bulkier cores, are unlikely to be sterically compatible with PylRS, which helps explain the orthogonality of the PylRS/tRNA^Pyl^ pair.

The crystallization of the N-terminal domain of N^+^-*Mm*PylRS resulted in a more complete understanding of the structure of the PylRS-tRNA^Pyl^ complex (Figure 5a).^60^ The N-terminal domain folds into a compact globular protein which binds one zinc ion. The fold is unique among known synthetases. The N-terminal domain tightly fits into the concave surface generated by the T-loop and the variable loop. The compact fit prevents canonical tRNAs with larger variable loops from being efficiently recognized. The N-terminal domain and C-terminal domain surround the tRNA^Pyl^ and form the largest interaction surface known for an aaRS-tRNA complex.^60^

### 3.5 The Active Site of PylRS Accepts Pyl Analogs

Early observations demonstrated that N^+^-*Mb*PylRS can activate a variety of different **Pyl** analogs;^70,71^ these experiments were performed with analogs, as access to **Pyl** was limited due to its challenging chemical synthesis.^72−74^ Later, it was shown that the active site of S^Δ^-*Dh*PylRS is also promiscuous, but restricted to smaller **Pyl** analogs.^65^ These observations are in line with S^Δ^-*Dh*PylRS having a smaller active site, as L309 in N^+^-*Mm*PylRS corresponds to W139 in S^Δ^-*Dh*PylRS.

The promiscuity of PylRS enzymes is essentially limited to changes in the substituent at the secondary carbonyl.^58^ The relaxed fit of **Pyl** in the large hydrophobic pocket, in which the side chain binds mainly through nondirected van der Waals interactions, is consistent with the observed promiscuity. Thus, PylRS predominantly recognizes amino acid substrates composed of a secondary carbonyl (which may engage in hydrogen bonding with N346^45^) linked to large hydrophobic side chains.

### 3.6 PylRS Does Not Recognize the Anticodon of tRNA^Pyl^

Biochemical characterization of S^+^-*Dh*PylRS/S-*Dh*tRNA^Pyl^ demonstrated that the anticodon is not a recognition element of PylRS enzymes.^67^ The anticodon of S-*Dh*tRNA^Pyl^ could be switched without significantly affecting S-*Dh*PylRSn binding or S^Δ^-*Dh*PylRS acylation efficiencies. Numerous experiments demonstrated that PylRS enzymes do not require conserved nucleotides in the anticodon stem of their pyl tRNAs for efficient aminoacylation.^57,59,60,63,75−80^ Structural analysis of the PylRS-tRNA^Pyl^ complex substantiated previous observations that PylRS does not interact with anticodon stem-loop.^59,75^

While mutations in the anticodon stem loop have little effect on aminoacylation, ribosomal translation in *E. coli* is more efficient with tRNAs bearing specific nucleotides adjacent to the anticodon;^81^ therefore, translational readthrough of the amber codon in *E. coli* is sensitive to the identity of nucleotides in the anticodon stem and loop. This explains why mutagenesis of nucleotides adjacent to the anticodon (U33 and A37) in N^+^-*Mb*tRNA^Pyl^ decreased translational readthrough of amber codons in *E. coli*.^75^ Similarly, while some archaeal pyl tRNAs have C37 in their natural anticodon stem loop,^35,38−40^ the C37A mutants of these pyl tRNAs led to more efficient amber suppression than the native sequence in *E. coli*.^39^

## 4 Genetic Code Expansion with PylRS/tRNA^Pyl^ Pairs

A number of aaRS/tRNA pairs have been developed for genetic code expansion.^20,22,35,38−40,82−92^ However, the PylRS/tRNA^Pyl^ pair has several properties that make it ideal for genetic code expansion: (1) The PylRS/tRNA^Pyl^ pair is orthogonal in *E. coli* (Section 4.1),^82^ (2) the pair does not use canonical amino acids and can be engineered to accept a wide-range of new substrates (Section 4.2), (3) the pair is a natural amber suppressor, (4) the anticodon of tRNA^Pyl^ can be altered to decode diverse codons, and (5) the PylRS/tRNA^Pyl^ pairs tested are orthogonal in all kingdoms of life,^3,20,82,93−108^ such that variants evolved for new substrates in *E. coli* can be transplanted to other prokaryotes, eukaryotic cells, plants, and animals (Section 4.3). A number of innovative approaches have been developed to increase the efficiency of ncAA incorporation with PylRS/tRNA^Pyl^ systems in both prokaryotic and eukaryotic systems (Section 4.4). Because of their unique advantages PylRS/tRNA^Pyl^ pairs have rapidly become the most widely used systems for genetic code expansion and reprogramming.

### 4.1 PylRS/tRNA^Pyl^ Pairs Are Orthogonal in *E. coli*

Orthogonal aaRS enzymes aminoacylate their cognate (orthogonal) tRNA but minimally acylate other tRNAs in the cell of interest. Similarly orthogonal tRNAs are substrates for their cognate (orthogonal) aaRS but not efficient substrates for any endogenous synthetases in the cell of interest. An orthogonal pair is composed of an orthogonal aaRS and orthogonal tRNA. The N^+^-*Mb*PylRS/N-*Mb*tRNA^Pyl^ pair (and the analogous *Mm* pair) are orthogonal in *E. coli*;^82^ N-*Mb*tRNA^Pyl^ is minimally acylated by endogenous synthetases in this host.^22,68,82^ N^+^-*Mb*PylRS and its active site variants direct the incorporation of their substrates in response to the amber codon without directing measurable misincorporation of their substrates, in competition with canonical amino acids, at sense codons.^82^

### 4.2 Engineering PylRS Enzymes for the Incorporation of ncAAs

With an orthogonal aaRS/tRNA pair in hand, the next challenge was to engineer the synthetase such that it uses a desired ncAA and no canonical amino acids. The most common way to engineer PylRS to use ncAAs uses double-sieve selection on the amber suppression activity of PylRS/tRNA^Pyl^_CUA_ pairs (Section 4.2.1). Recent work has also explored the use of chimeras between PylRS and other synthetases as a starting point for generating enzymes for ncAA incorporation (Section 4.2.2).

#### 4.2.1 Selections for PylRS Variants That Direct ncAAs into Proteins

**Figure 6:**
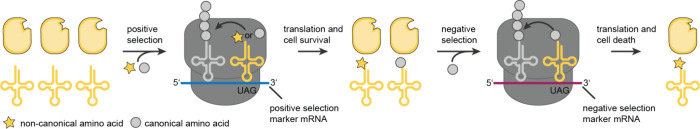
**Double-sieve selection strategy for directed evolution of ncAA specificity in orthogonal aaRS enzymes**. Aminoacyl-tRNA synthetase libraries are first submitted to a round of positive selection in the presence of the target ncAA. In this step, the acylation of a suppressor tRNA and ribosomal translation through an amber codon in a positive selection marker mRNA (frequently chloramphenicol acetyltransferase) is linked to cell survival. Next, surviving library members are submitted to a negative selection step in the absence of the ncAA, where acylation of a suppressor tRNA and ribosomal translation through an amber codon in a negative selection marker (frequently barnase) is linked to cell death. Aminoacyl-tRNA synthetase variants that selectively charge the target ncAA, and no canonical amino acids, onto their cognate tRNA survive both steps of selection. For this selection approach to work, ncAAs must function with the ribosome and other translation factors. Additional rounds of selection can be performed. Adapted with permission from Chin et al.^3^ – copyright © 2014 Annual Reviews.

The most established and general method to alter the specificity of orthogonal aaRS enzymes to selectively incorporate a ncAA of interest relies on double-sieve selections on a library of synthetase mutants; these libraries commonly use saturation mutagenesis at five or more positions in the region of the gene corresponding to the active site of an enzyme, though small intelligent libraries that mutate several positions to a smaller subset of codon possibilities have also proved useful.^1−6^ Double-sieve selections for ncAA incorporation were first successfully demonstrated for the evolution of *Methanococcus jannaschii* tyrosyl–tRNA synthetase (*Mj*TyrRS) to direct the incorporation of the photo-cross-linker *para*-benzoyl-*L*-phenylalanine (**BpA**) in response to the amber codon in *E. coli*.^109^ In this approach (Figure 6), synthetase variants that load an amino acid (the ncAA or a canonical amino acid) are selected in a positive selection step; this step uses a positive selection marker (e.g., chloramphenicol acetyl transferase (cat)) containing an amber codon at a permissive site in its gene, and selects for synthetases that acylate their cognate tRNA and enable the production of the positive selection marker in the presence of the added ncAA. Synthetase genes that survive the positive selection are then subjected to a negative selection step; this step uses a negative selection marker (e.g., barnase) containing one or more amber codons at permissive sites in its gene, and selects against synthetases that acylate their cognate tRNA and enable the production of the negative selection marker in the absence of the added ncAA.^82^ Double-sieve selections have been extended to select for ncAA specific synthetases in eukaryotic cells.^110^

In some cases, positive and negative screens, using fluorescence-based readouts have been used in place of selections,^111−116^ and these approaches have been extended to *in vitro* screening in liposomes.^117^ Negative screens have the advantage of allowing the experimenter to more precisely gate the amount of synthetase activity permissible in the absence of the ncAA (something that can in principle be achieved by tunable negative selections);^118^ this is potentially advantageous for identifying the most active and sufficiently selective synthetases,^119^ since the fidelity of the genetic code is in part controlled by competition.^120,121^ However, screening approaches generally come at the cost of lower throughput.

The double-sieve selection was adapted to select N^+^-*Mb*PylRS/N-*Mb*tRNA^Pyl^ variants that direct the cotranslational incorporation of *N*^ε^-acetyl-*L*-lysine (**AcK**), a key post translational modification (PTM), in response to the amber codon in *E. coli*.^82^ This demonstrated that PylRS could be evolved in the laboratory to incorporate ncAAs. Over the past 16 years PylRS/tRNA^Pyl^ pairs have been evolved and engineered^58^ to incorporate hundreds of ncAAs with numerous applications (Section 6), including numerous and diverse acylated lysine derivatives.

Furthermore, the active site of PylRS enzymes can easily be remodeled to accommodate residues with side chains containing aromatic groups.^56^ A number of phenylalanine derivatives are substrates for engineered PylRS enzymes, and PylRS may also direct the incorporation of larger aromatic ring systems.^19,120,122−132^ PylRS enzymes for histidine,^19,133,134^ tyrosine,^135−138^ or tryptophan^139−141^ analogs have also been developed. We note that a manually curated databank of PylRS active site variants and the ncAAs that these variants have been reported to incorporate (as measured by intact MS verification of the modified protein) has been generated.^142^

Parallel positive selections coupled to deep sequencing, developed to generate a phosphothreonine specific aaRS from the phosphoseryl-tRNA synthetase (SepRS) of *Methanococcus maripaludis*, have also been used to evolve PylRS enzymes in proof-of-principle experiments in *E. coli*.^143^ In this method, two or more positive cat selections are run in parallel in the presence and absence of the desired ncAA. All surviving colonies are subsequently isolated and the gene pool is subjected to next-generation sequencing (NGS). Analysis of the selected synthetase sequences leads to the identification of aaRS variants that are enriched in the plus ncAA sample with respect to the minus ncAA sample.

Until recently, all selections for synthetases for ncAA incorporation relied on protein synthesis-based read-outs and consumed substantial quantities of ncAA. Recent work, aimed at incorporating ncMs that may not function efficiently in protein synthesis or be efficient ribosomal substrates, developed tRNA display^19^ – a direct selection for synthetases that aminoacylate their cognate tRNAs with ncMs, whether or not the resulting acylated tRNAs function in translation. tRNA display has been used to evolve N^+^-*Mm*PylRS variants for eight ncAAs and eight ncMs, including six β-amino acids, one α,α-disubstituted-amino acid and one β-hydroxy acid. This approach is discussed in detail in Section 7.

#### 4.2.2 PylRS Chimeras for ncAA Incorporation

**Figure 7:**
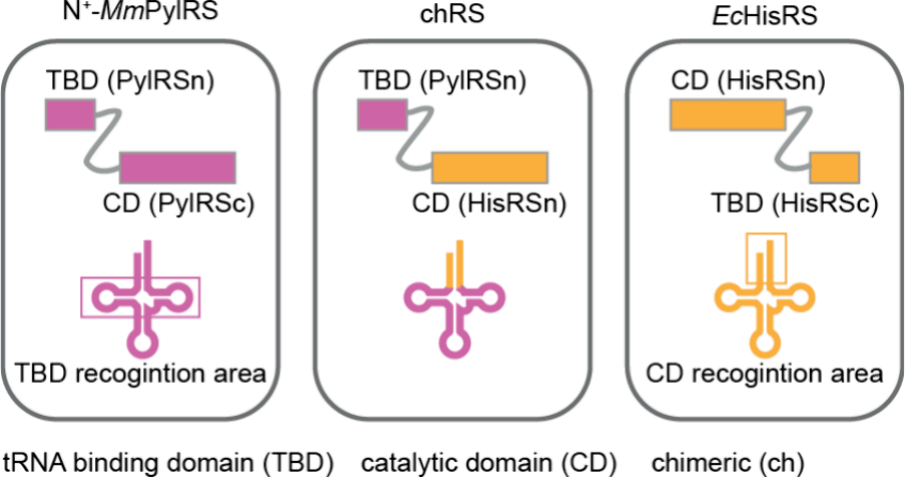
**Generation of chimeric aminoacyl-tRNA synthetase/tRNA (chRS/chtRNA) pairs for genetic code expansion**. The tRNA binding function of PylRS enzymes can be coupled to the catalytic domain of certain canonical aaRS enzymes forming orthogonal chRS/chtRNA pairs. Like N^+^-*Mm*PylRS, *E. coli* histidyl-tRNA synthetase (*Ec*HisRS) has two distinct domains connected by a flexible linker. One domain is responsible for tRNA binding (C-terminal domain - HisRSc), and one for the catalytic activity (N-terminal domain - HisRSn). The catalytic domain (CD) predominantly interacts with the acceptor stem of *Ec*tRNA^His^ and the tRNA binding domain (TBD) predominantly interacts with the anticodon stem and loop of *Ec*tRNA^His^. A chRS is generated through the fusion of the TBD of N^+^-*Mm*PylRS (which predominantly interacts with the T-, and D-stem, as well as the T- and variable loop of N-*Mm*tRNA^Pyl^) with the CD of *Ec*HisRS. The combination of the chRS with the engineered chtRNA, where the acceptor stem in N-*Mm*tRNA^Pyl^ was replaced with the acceptor stem of *Ec*tRNA^His^, resulted in an orthogonal chRS/chtRNA pair. This pair combined the aminoacylation specificity of *Ec*HisRS with the tRNA recognition and orthogonality of N^+^-*Mm*PylRS and could be used in prokaryotic and mammalian cells. A chimeric PheRS/tRNA^Phe^ pair was also engineered. Adapted from Ding et al.^144^ – copyright © The Author(s) 2020 CCBY http://creativecommons.org/licenses/by/4.0/.

Recent work has attempted to leverage the unique characteristics of the PylRS/tRNA^Pyl^ pair by generating chimeras that combine the tRNA binding abilities of the N-terminal domain of PylRS enzymes with the substrate scope of the catalytic domains of aaRS/tRNA pairs for canonical amino acids (Figure 7).^144^ In a proof-of-concept experiment, the catalytic domain of *E. coli* histidyl-tRNA synthetase (*Ec*HisRS) was linked through a flexible linker to N-*Mb*PylRSn. The architecture of *Ec*HisRS, with a distinct N- and C-terminal domain, in which the N-terminal domain performs catalysis and the C-terminal domain is responsible for anticodon recognition, facilitated the design of the fusion protein. The catalytic domain of *Ec*HisRS predominantly binds the acceptor arm of its cognate tRNA and N-*Mb*PylRSn mainly interacts with the D- and T-stem and the variable and T-loop of tRNA^Pyl^. Therefore, tRNA chimeras composed of the body of N^+^-*Mb*tRNA^Pyl^ and the acceptor arm of *Ec*tRNA^His^ were generated. A further mutated version of the pair led to 60% amber suppression efficiency for incorporating histidine when compared to wild type (wt) green fluorescent protein (GFP) production.^144,145^

As aaRS/tRNA pairs for other canonical amino acids do not commonly have easily separatable N- and C-terminal domains, with distinct catalytic or tRNA binding functions, generalizing this approach to produce efficient chimeras for other chemotypes has proven challenging. Nonetheless, the chimera between the N-*Mb*PylRSn and the catalytic domain from human mitochondrial PheRS, led to an orthogonal chimeric pair (chPheRS/chtRNA^Phe^) with 6% amber suppression efficiency when compared to wt GFP production. This pair was extensively engineered and evolved using double-sieve selections, and versions of the pair were developed to incorporate a number of phenylalanine, tyrosine, and tryptophan analogs.^144^ Extensive engineering and evolution of this system enabled the efficient and selective incorporation of *p*-azido-*L*-phenylalanine (*p***-AzF**), and the authors generated an *E. coli* strain that was synthetically auxotrophic for this ncAA.^145^ The highly engineered chPheRS/chtRNA^Phe^ was also used for the genetic encoding of tryptophan analogues that can be deprotected *in vivo* to reveal tryptophan residues.^146^

### 4.3 Transplanting PylRS Systems for ncAA Incorporation to Other Organisms

**Figure 8:**
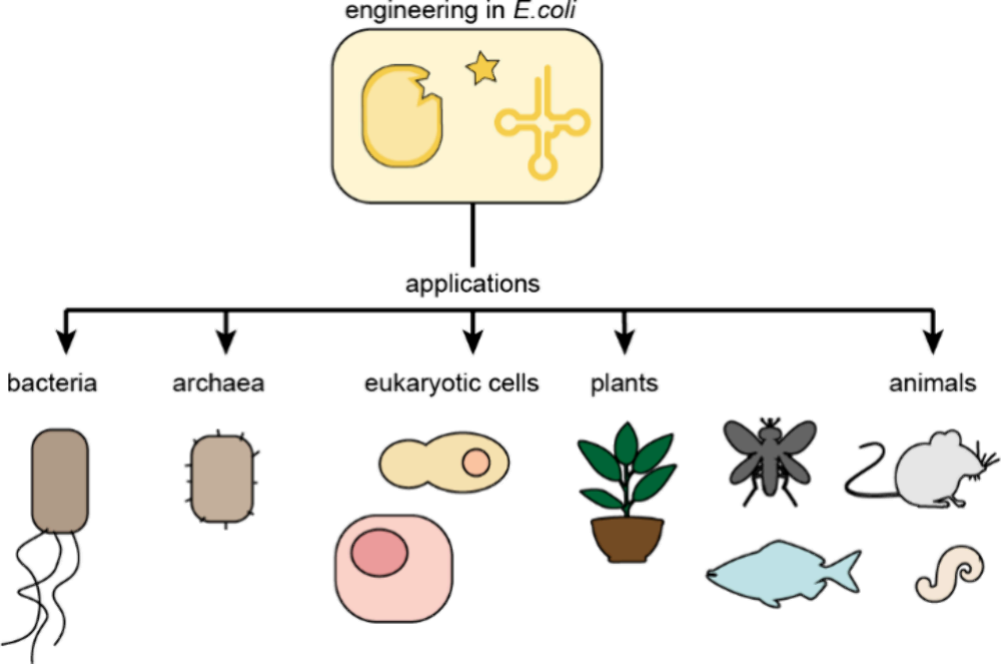
**PylRS/tRNA**^**Pyl**^**pairs are orthogonal and have been used for ncAA incorporation across all domains of life.** PylRS/tRNA^Pyl^ pairs can be engineered for ncAA specificity in *E. coli* cells, using directed evolution approaches, and then used for genetic code expansion in diverse host organisms including bacteria, archaea, eukaryotic cells, a plant species, and several animals.

PylRS/tRNA^Pyl^ pairs have been used to expand the genetic code across all domains of life and PylRS variants developed in *E. coli*, where directed evolution and engineering are most straightforward, have now been transplanted to a wide range of other organisms (Figure 8). The underpinnings of this approach have been extensively reviewed,^3^ and this section provides a brief summary and update.

#### 4.3.1 Genetic Code Expansion in Other Prokaryotes

PylRS/tRNA^Pyl^ pairs are orthogonal, and have been used for genetic code expansion in diverse prokaryotes including *Rhodobacter sphaeroides*,^99^*Neisseria meningitidis*,^100^*cyanobacteria*,^101,147^*Pseudomonas aeruginosa*,^102^*Bacillus subtilis*,^148^*Lactococcus lactis*,^103^*Streptomyces albus*,^104^*Shigella* and *Salmonella*.^105^

#### 4.3.2 Genetic Code Expansion in Eukaryotic Cells

PylRS/tRNA^Pyl^ pairs are orthogonal in eukaryotic cells. However, the expression, processing, and nuclear export of heterologous tRNAs poses specific challenges for genetic code expansion in eukaryotic cells. Eukaryotic tRNA genes contain internal A-and B-box elements that are required for their RNA polymerase III-dependent transcription. Genes encoding tRNA^Pyl^ lack these A- and B-box elements. Therefore, extragenic RNA polymerase III promoters, which contain their own A- and B-box sequences, have been used to transcribe tRNA^Pyl^.^93,149^ This approach has the additional advantage of disentangling the sequence of the tRNA from its ability to be transcribed, enabling the sequence of the tRNA to be optimized for intrinsic function. Although the addition of the 3′CCA end is part of tRNA maturation in eukaryotes,^150^ the tRNA^Pyl^ gene is usually introduced with the 3′CCA end included.^93,149^

In *Saccharomyces cerevisiae* (*S. cerevisiae*), placing eukaryotic tRNA genes with their own A- and B-box sequences upstream of the N-*Mm*tRNA^Pyl^ gene, and expressing N^+^-*Mm*PylRS from standard promoters enables the creation of functional genetic code expansion systems. An early system used a human leucine tRNA gene as a promoter for the N-*Mm*tRNA^Pyl^ gene, and reported preliminary growth phenotypes consistent with weak ncAA dependent amber suppression.^93^ Using the yeast tRNA^Arg^_UCU_ gene as a promoter for the N-*Mm*tRNA^Pyl^ gene led to high level expression of *Mm*tRNA^Pyl^, this system was used to demonstrate that N^+^-*Mm*PylRS and N-*Mm*tRNA^Pyl^ (but not N-*Mb*tRNA^Pyl^) are orthogonal in yeast. Using this system five different ncAAs were site-specifically incorporated into recombinant proteins, and ncAA incorporation was characterized by MS and MS/MS.^151^ Recent work has demonstrated that the SNR52 promoter (which also contains A- and B-box elements) can be used to express *Methanomethylophilus alvus* (*alv*) A-*alvt*RNA^Pyl^ in *S. cerevisiae*; this enabled ncAA incorporation with the A^Δ^-*alv*PylRS/A-*alvt*RNA^Pyl^ pair in *S. cerevisiae*;^152^ these experiments demonstrated how the development of orthogonal PylRS/*t*RNA^Pyl^ pairs in *E. coli* can lead to rapid advances in other organisms.

PylRS/tRNA^Pyl^ pairs are also orthogonal in mammalian cells, where the expression of tRNA^Pyl^ is commonly driven by a U6 promoter (which contains A- and B-box sequences) with or without a cytomegalovirus (CMV) enhancer.^91,93,144,145,153−156^

A systematic optimization of ncAA incorporation efficiency with the N^+^-*Mm*PylRS/N-*Mm*tRNA^Pyl^ pair in mammalian cells, revealed that tRNA^Pyl^ concentration is a key factor in increasing the amount of ncAA containing recombinant protein produced.^149^ N-*Mm*tRNA^Pyl^ levels were optimized by using eight copies of the N-*Mm*tRNA^Pyl^ gene, each under a U6 promoter; this led to substantially more tRNA production than a CMV enhancer U6 promoter system, and the increase in tRNA levels was correlated with a ten to 20-fold increase in ncAA incorporation efficiency.

Transient transfection of PylRS/tRNA^Pyl^ genes in mammalian cells leads to a heterogeneous population of cells with great variability in the levels of PylRS and tRNA^Pyl^ between cells. This results in variability in stop codon read through levels, and therefore variability in ncAA incorporation efficiency, between cells.^106^ By using a PiggyBac system, eight copies of a gene encoding tRNA^Pyl^, a single copy of a gene encoding PylRS, and genes of interest containing amber stop codons at the desired positions, were introduced into the genomes of diverse mammalian cell lines.^106^ Stable integration of the genes encoding a PylRS/tRNA^Pyl^ pair variant permitted the site-specific installation of preacylated lysine residues in histones, providing an orthogonal means to study the consequence of a PTM at a specific site in a protein without the pleotropic effects that may result from manipulating acetyl-transferases or deacetylases. These experiments also defined genes that are up or down regulated as a result of amber suppression in mouse embryonic stem cells. Other stable cell lines for PylRS/tRNA^Pyl^ pairs have since been developed,^157−159^ and PylRS/tRNA^Pyl^ pairs have also been used to incorporate ncAA into viruses,^160−162^ insect cells^163^ and brain organoids.^164^

PylRS/tRNA^Pyl^ pairs have also been used to expand the genetic code of plants and animals. The first genetic code expansion in a multicellular system was demonstrated in the nematode *Caenorhabditis elegans* (*C. elegans*).^95,98,165^ The orthogonality of the PylRS/tRNA^Pyl^ pair, and ncAA incorporation, was further demonstrated in several model organisms, including the plant *Arabidopsis thaliana*,^97^ and animals: *Drosophila melanogaster*,^94,166,167^*Mus musculus*,^108,168^ and *Danio rerio*.^107,169^

### 4.4 Improving the Efficiency of ncAA Incorporation with PylRS/tRNA Pairs

PylRS/tRNA^Pyl^ variants that enable ncAAs incorporation have provided a starting point for work aimed at improving the efficiency of ncAA incorporation through further engineering of PylRS and tRNA^Pyl^. These approaches complement strategies to enhance ncAA incorporation at a target codon by minimizing competition with translation factors that otherwise decode that codon, or improving strains for ncAA amino acid incorporation.^77,149,152,170−173^ Here we focus on strategies for engineering the intrinsic properties of PylRS/tRNA^Pyl^ systems, rather than the expression level of the pair, to increase ncAA incorporation, and on approaches where PylRS/tRNA^Pyl^ systems were central to the development of strategies for improving ncAA incorporation.

#### 4.4.1 Improving the Efficiency of ncAA Incorporation in *E. coli*

A continuous evolution approach, based on phage assisted continuous evolution (PACE),^174^ was investigated to improve the activity of a chimeric PylRS (N^+^-chPylRS - residues 1–149 of N^+^-*Mb*PylRS fused to residues 185–454 of N^+^-*Mm*PylRS) with *N*^ε^-(*tert*-butoxycarbonyl)-*L*-lysine (**BocK**). This approach selected for the ability of a N^+^-chPylRS/N-tRNA^Pyl^ pair to read through amber stop codons in the M13 *pIII* gene (or to read through amber stop codons in a *T7 RNA polymerase* gene, in a less stringent system where T7 RNA polymerase transcribed the *pIII* gene), in mutagenic *E. coli* provided with **BocK**. The pIII protein is required for generating infective phage particles. Active N^+^-chPylRS/N-tRNA^Pyl^ pairs led to production of infective phage carrying the corresponding N^+^-chPylRS gene, but inactive N^+^-chPylRS/N-tRNA^Pyl^ pairs did not. The phage carrying active N^+^-chPylRS, and bearing the pIII protein could infect fresh mutagenic cells where they were subject to further mutations in N^+^-chPylRS. This approach led to four consensus mutations in the N-terminal domain of the chimera (V32I, T56P, H62Y, and A100E). The combination of these mutations in N^+^-chPylRS led to a 1.5-fold increase in the production of GFP from a gene containing an amber stop codon (from 40% to 60% of a wt GFP control) and an about 4-fold increase in GFP from a gene containing three amber stop codons. Transfer of the mutations to PylRS variants for other ncAAs also led to increases in incorporation efficiency. Experiments that mutated PylRS, its N-terminal domain, or the linker connecting the N- and C-terminal domains, led to similar increases in efficiency.^111,117,175−177^ N-terminal solubility tags have also been used to increase the amber suppression efficiency of the N+-*Mb*PylRS/*Mb*tRNA^Pyl^ pair several fold.^178^ Additionally, a mutation in the N-terminal domain of N^+^-*Mm*PylRS may increase stability to proteolysis and enhance the performance of the enzyme.^179^

Intriguingly, PACE also generated split N^+^-chPylRS enzymes, where mutations generated a stop codon separating N- and C-terminal domains into two fragments (an in sequence AGT codon enabled the translation initiation of the C-terminal domain). Maximal amber suppression activity required the presence of both domains. Continuous evolution approaches have the potential to improve the ncAA dependent activity of PylRS/tRNA^Pyl^ pairs.^180−185^

One strategy to improve ncAA incorporation with PylRS/tRNA^Pyl^ pairs has focused on the mutation of nucleotides in the T-stem and acceptor stem of N-*Mm*tRNA^Pyl^ (49:65, 50:64, 6:67 and 7:66). These nucleotide positions are known to be important for recognition of *E. coli* tRNAs by endogenous EF-Tu.^186^ The variant tRNA^Pyl^, named N-*Mm*tRNA^Pyl-opt^ led to a 3-fold increase in **AcK** incorporation in response to one amber codon and a 5-fold increase in incorporation in response to two amber codons, when compared to the parent tRNA^Pyl^. Although N-*Mm*tRNA^Pyl-opt^ improved the incorporation of multiple chemically distinct ncAAs, the extent of the increase in yield varied substantially; this observation is consistent with the thermodynamic compensation between amino acid and tRNA binding to EF-Tu^187^ and suggests that tRNA^Pyl^ may need to be independently optimized for each ncAA.

#### 4.4.2 Improving the Efficiency and Specificity of ncAA Incorporation with PylRS/tRNA^Pyl^ Pairs in Eukaryotic Systems

Archaeal pyl tRNAs have secondary structures which are highly divergent from canonical mammalian tRNAs, but are shared by mitochondrial tRNA^Ser^_UGA_. Researchers attempted to improve overall tRNA^Pyl^ activity in mammalian cells by substituting single bases or base pairs in N-*Mm*tRNA^Pyl^ with the corresponding bases from human tRNAs. In a series of experiments, they inserted tRNA^Pyl^ recognition elements for PylRS enzymes from group +N and sN into *Bos taurus* (*Bt*)tRNA^Ser^_UGA_ and assessed the amber suppression activity of all hybrid tRNAs in mammalian cells (in prior work, the sequences recognized by N^+^-*Mb*PylRS within N-*Mb*tRNA^Pyl^ had been transplanted into the mitochondrial tRNA from *Bt*tRNA^Ser^_UGA_ to generate chimeras, some of which were active in *E. coli*(75)). Mutants bearing the highest number of tRNA^Pyl^ bases generally performed best. One chimera based on N-*Mm*tRNA^Pyl^ - termed N-*Mm*tRNA^Pyl-M15^ - led to a roughly 2.5-fold improvement of **BocK** incorporation when paired with a N^+^-*Mb*PylRS variant in mammalian cells.^188^ Interestingly, the most active mutants in mammalian cells acquired a canonical B-box in the T arm, which is absent in native tRNA^Pyl^. The study confirmed that tRNA^Pyl^ engineering can increase the efficiency of ncAA incorporation in eukaryotic cells. However, the increase in activity is limited to incorporating some ncAAs, and maximal incorporation efficiency may require a new tRNA^Pyl^ for each ncAA.^188^

**Figure 9:**
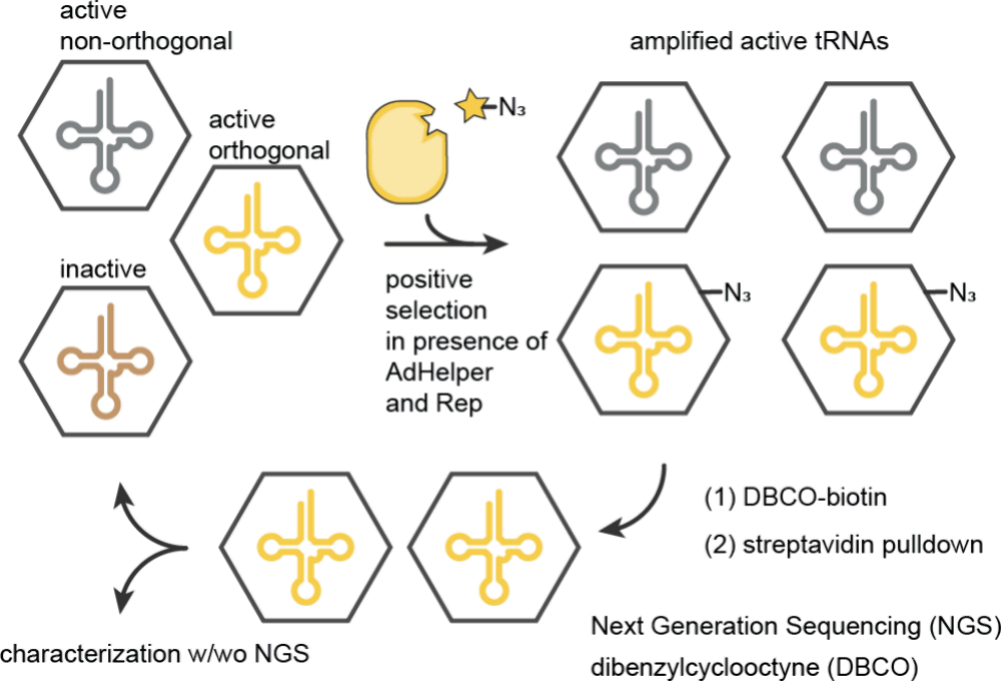
**Virus-assisted directed evolution of tRNAs (VADER) in mammalian cells:** tRNA^Pyl^ libraries are encoded in the DNA of adeno-associated viruses 2 (AAV2), such that each virus (hexagon) only carries one tRNA variant. The replication of the virus is coupled to amber suppression. N^+^-*Mm*PylRS dependent amber suppression leads to the incorporation of an azide functionality on the surface of the virus. Viruses harboring a selective and active N-*Mm*tRNA^Pyl^ can be isolated on streptavidin beads by bio-orthogonal labeling and either submitted to additional rounds of evolution, or further characterization by single colony sequencing or NGS. Adapted from Jewel et al.^190^ – copyright © The Author(s) 2020 CCBY-NC-ND https://creativecommons.org/licenses/by-nc-nd/4.0/.

Since tRNA^Pyl^ needs to be transcribed, processed, modified, and exported from the nucleus and function with the endogenous translational machinery, it is not straightforward to predict what sequence changes may lead to optimized ncAA incorporation in mammalian cells. These considerations suggested that there may be value in developing methods for the directed evolution of tRNAs in these cells. “Virus-assisted directed evolution of tRNAs” (VADER) has been developed for directed evolution of tRNAs that direct more efficient ncAA incorporation in mammalian cells (Figure 9).^189^

In VADER, tRNA gene libraries were encoded in the DNA of adeno-associated viruses 2 (AAV2), such that one virus carried one tRNA genotype, and the replication of the virus was rendered dependent on the suppression of amber codons introduced into the gene for a virus capsid protein. Mammalian cells were infected with the virus, and transfected with the gene for a N^+^-*Mb*PylRS variant that–when provided with a cognate N-*Mm*tRNA^Pyl^ – directed the incorporation of an azide containing ncAA in response to amber codons in the viral gene; cells also contained the other genes necessary for virus replication.

Inactive N-*Mm*tRNA^Pyl^ variants did not make viral particles, and active N-*Mm*tRNA^Pyl^ variants led to the production of viral particles. Cells which contained active N-*Mm*tRNA^Pyl^ variants, that are aminoacylated by the N^+^-*Mb*PylRS variant, produced virus particles that displayed an azide on their capsid and contained the gene for the N-*Mm*tRNA^Pyl^ variant in their DNA. These viruses were labeled with a biotinylated alkyne probe, and affinity purified for sequencing or further rounds of evolution.

The most active hit (N-*Mm*tRNA^Pyl-A2.1^), selected from a library of acceptor stem mutants, led to a 3-fold increase of the incorporation of the azide containing lysine derivative in mammalian cells. Similar increases in ncAA incorporation were observed for other ncAAs when using N-*Mm*tRNA^Pyl-A2.1^. There was no increase in ncAA incorporation with N-*Mm*tRNA^Pyl-A2.1^ in *E. coli*, suggesting that the observed increases in ncAA incorporation were host cell specific. Recent improvements in VADER have enabled the identification of more active N-*Mm*tRNA^Pyl^ variants in mammalian cells.^190^ As this approach relies on the use of a particular ncAA, it may be challenging to adapt it to select tRNAs that are optimized for different ncAA chemotypes.

Analysis of the spatial distribution of an active site mutant of N^+^-*Mm*PylRS in mammalian cells suggested that the mutant enzyme predominantly clustered in the nucleus. Sequence analysis identified a putative nuclear localization signal (NLS) near the N-terminus of N^+^-*Mm*PylRS. The authors theorized that a large proportion of the mutant N^+^-*Mm*PylRS/N-*Mm*tRNA^Pyl^ pair was segregated from cytosolic translation, hampering the overall efficiency of ncAA incorporation in mammalian cells.^191^ In these experiments, attaching a nuclear export signal (NES) to this mutant redirected the enzyme to the cytosol. The authors reported a 15-fold increase in amber suppression efficiency with the mutant N^+^-*Mm*PylRS enzyme bearing an NES and used their system for super resolution imaging by ‘points accumulation for imaging in nanoscale topography’ (PAINT).^108^ Prior work had shown that some N-*Mb*tRNA^Pyl^ was in the nucleus and used a system without an NES for super resolution imaging by ‘stochastic optical reconstruction microscopy’ (STORM).^192^ It is unclear to what extent the effect of the NES on ncAA incorporation efficiency reported in the later work was a function of the specific PylRS mutant and the expression level of the PylRS/tRNA^Pyl^ pair.

The use of PylRS enzymes from thermophilic archaea (*Methanosarcina thermophila (Mth* – 50 °C) and *Methanosarcina flavescens (Mfl −45 °C))*, which live at temperatures significantly above those observed for N^+^-*Mb*PylRS (37 °C) and N^+^-*Mm*PylRS (35 °C), has shown promise in optimizing ncAA incorporation experiments in mammalian cells.^193^ The wt N^+^-*Mth*PylRS, and N^+^-*Mfl*PylRS promoted the incorporation of **BocK** at an amber codon to an equivalent or lower level than N^+^-*Mb*PylRS; however, a series of PylRS variants (derived from active site transplants from evolved PylRS mutants) were more active in the N^+^-*Mth*PylRS and N^+^-*Mfl*PylRS scaffolds, than in the N^+^-*Mb*PylRS scaffold. A potential reason for this difference could be the higher tolerance of the more thermally stable enzymes to active site perturbations. All measured active site transplants led to enzymes with lower thermal stability than their scaffolds, but starting with a thermostable scaffold led to more stable transplants.

A eukaryotic release factor 1 mutant (eRF1 - E55D) was discovered that maintains efficient termination on UGA and UAA codons, but facilitated increased ncAA incorporation at amber codons with N^+^-*Mm*PylRS/N-*Mm*tRNA^Pyl^ pairs. This system was used to produce GFP bearing a ncAA in yields rivaling wt protein production, and faciliated the incorporation of two or three copies of a ncAA into GFP in mammalian cells.^149^ The use of tailored expression and delivery systems, as well as eRF1 mutants, provided additional examples of optimized systems for PylRS/tRNA^Pyl^ pair performance in mammalian cells.^194−196^

An important factor affecting the efficiency of ncAA incorporation via amber suppression is the sequence context in which the ncAA is to be encoded. Recent work has employed the piggyBac system expressing the PylRS/tRNA^Pyl^ pair in combination with stochastic orthogonal recoding of translation with enrichment (SORT-E, see Section 9.4.1)^166,167^ to capture and quantify polypeptides resulting from read through of endogenous amber codons. Using this data, the authors generated a model to predict sequence contexts that favor amber suppression and validated this model experimentally.^173^

Despite the large number of endogenous amber codons in mammalian cells, ncAAs are selectively incorporated into target proteins in response to in frame amber codons introduced into transgenes, and there is minimal steady state incorporation of ncAAs at endogenous termination codons.^192,197^

Investigators have colocalized genetic code expansion components to membraneless, phase separated, spatially localized, subcellular compartments with the goal of enhancing the specificity with which a stop codon of interest is decoded to incorporate a ncAA.^198^ To achieve this, target mRNAs bearing a stop codon were equipped with an ms2 tag that is specifically bound by major capsid protein (MCP). N^+^-*Mm*PylRS and MCP were fused to proteins that undergo phase separation in cells, and to kinesin motor proteins (which are specifically enriched at microtubule plus ends). The resulting localized membraneless organelles supported protein translation with an efficiency of about 40% that of cytoplasmic translation. Translation through an amber codon in an ms2 tagged message (localized to a subcompartment) was favored over translation through an amber codon in non-ms2 tagged message (localized to the cytosol) by a factor of 5. In an extension of this work, PylRS variants for distinct ncAAs were directed to distinct sub cellular compartments, and distinct mRNAs bearing amber stop codons were directed to each compartment. This enabled the incorporation of a different ncAA in response to the amber codon in each message.^199^ This work increased the efficiency of translation in sub compartments to about 80% of cytoplasmic translation. It also increased the specificity for translating through an amber codon in a sub compartment to translating through and amber codon in the cytosol to 20-fold. These interesting approaches have thus far been investigated using transient transfection to introduce the genes of interest. In future work it will be interesting to investigate what happens to these systems during cell division and in long-term cultures. If synthetic subcompartments can be stably embedded in cells, without adversely perturbing natural biology, they may provide a powerful approach for generating compartmentalized genetic codes; this strategy could contribute to efforts to study *in vivo* biology with genetic code expansion, without effects that may arise from global amber suppression.

## 5 Biosynthesis of ncAA for Incorporation with PylRS/tRNA^Pyl^ Systems

A discrete body of work has focused on coupling strategies for the cellular biosynthesis (or semisynthesis) of ncAAs to the incorporation of the biosynthesized ncAAs into proteins using orthogonal aaRS/tRNA pairs in *E. coli*.^26,200−204^ Most recent work with the pyl systems has focused on exploiting the permissiveness of the natural pyl biosynthetic enzymes to analogues of their natural substrates, or engineering the pyl biosynthetic enzymes to accept new substrates; PylRS or its variants are then used to incorporate the biosynthetic products into proteins in *E. coli*. Endogenous metabolic enzymes have also been used to generate substrates for engineered PylRS variants.

**Figure 10:**
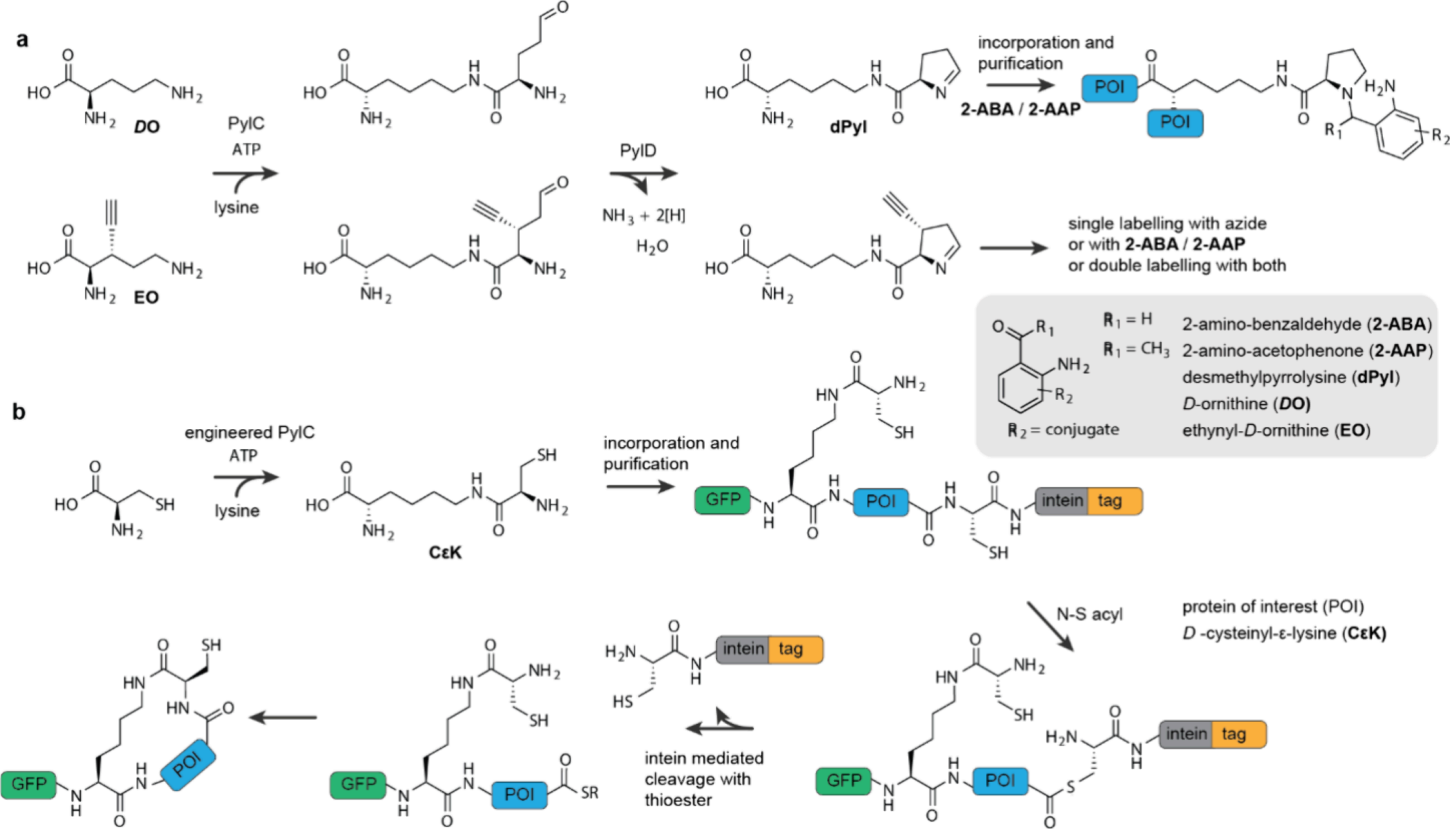
**Exploiting substrate promiscuity and engineering the pyl pathway for ncAA incorporation. a**, PylC and PylD can accept *D*-ornithine (*D***O**), or *S*-ethynyl-*D*-ornithine (**EO**) forming desmethylpyrrolysine (**dPyl**) with or without an alkyne substituent on the pyrroline ring. The imine of **dPyl** can be functionalized with 2-amino-benzaldehyde (**2-ABA**) or 2-amino-acetophenone (**2-AAP**) derived compounds. Furthermore, the alkyne can be labeled in bio-orthogonal reactions resulting in the double labeling of recombinant proteins. **b**, PylC can be engineered to accept d-cysteine forming *D*-cysteinyl-ε-lysine (**CεK**), which can be incorporated into recombinant proteins by an engineered N^+^-*Mb*PylRS/N-*Mb*tRNA^Pyl^ pair and used to cyclize proteins which contain intein-derived C-terminal thioesters.

PylC can direct the formation of an isopeptide bond between lysine and *D***O** (when added to *E. coli* in place of the **3MO**, the natural substrate of PylC) and PylD converts the product to desmethylpyrrolysine (**dPyl**).^24,29^ Since **dPyl** is a substrate for N^+^-*Mm*PylRS, feeding *D***O** to *E. coli* expressing PylC, PylD and PylRS/tRNA^Pyl^ pairs led to the incorporation of this ncAA in response to amber codons.^26^ The electrophilic imine of **dPyl** in proteins was used to conjugate proteins with diverse molecules through 2-amino-benzaldehyde (**2-ABA**) or 2-amino-acetophenone (**2-AAP**) reagents (Figure 10a).^202^ Similarly, the substrate promiscuity of PylC, PylD, and PylRS subsequently enabled *in vivo* production of proteins with an alkyne bio-orthogonal handle from *E. coli* cells transformed with *pylTSCD* and supplemented with 3-*S*-ethynyl-*D*-ornithine (**EO**) (Figure 10a).^203^

Recent work has aimed to engineer the substrate specificity of both PylC and PylRS to expand the range of ncAAs that can be biosynthesized in *E. coli* and incorporated into proteins (Figure 10b). In the first step, a N^+^-*Mb*PylRS/N-*Mb*tRNA^Pyl^ pair was engineered to incorporate *D*-cysteinyl-ε-lysine (**CεK**) into proteins in response to the amber codon, upon addition of the ncAA to *E. coli*.^204^ In the second step, several positions in the active site of PylC were subject to saturation mutagenesis, and cells containing the resulting PylC library, and an engineered N^+^-*Mb*PylRS/N-*Mb*tRNA^Pyl^ variant for **CεK**, were selected for on the basis of their ability to support amber suppression upon addition of d-cysteine to cells. This led to the discovery of a PylC mutant (S177N, E179P, D233S, and T256 V) that used added d-cysteine and endogenous lysine for the *in vivo* synthesis of **CεK**. Cells that biosynthesized **CεK** supported amber suppression by the engineered N^+^-*Mb*PylRS/N-*Mb*tRNA^Pyl^ variant at comparable levels to cells to when 4 mM, chemically synthesized, **CεK** was added.

Components of the pyrrolysine biosynthesis pathway, notably PylB, are suboptimally expressed in *E. coli*.^23^ Linking the N-terminus of PylB to small ubiquitin-related modifier (SUMO) increased solubility enabling purification and crystallization.^25^ Phage assisted noncontinuous evolution (PANCE) on a SUMO-PylB, PylC, PylD operon led to improved production of **Pyl** in *E. coli*.^205^ Most mutations accumulated in the *SUMO*-*pylB* gene, and were ascribed to increasing the solubility as well as the protease resistance of SUMO-PylB. When combined with the PylRS/tRNA^Pyl^ pair the evolved pathway yielded up to 32 times higher yields of proteins encoding **Pyl** than the starting pathway.

Other work has used enzymes that are endogenous to *E. coli* to assemble substrates for engineered N^+^-*Mm*PylRS variants. Addition of allyl mercaptan to *E. coli* led to the synthesis of *S*-allyl-*L*-cysteine via the reaction with endogenous *O*-acetyl-*L*-serine, a metabolic intermediate in cysteine biosynthesis. This reaction was catalyzed by endogenous *O*-acetyl-serine sulfhydrylase enzymes. A N^+^-*Mm*PylRS variant was evolved to direct the incorporation of *S*-allyl-*L*-cysteine.^206^

Given the range of chemotypes that can now be incorporated by PylRS variants it seems likely that both further engineering of the **Pyl** biosynthesis pathway as well as further engineering of other biosynthetic pathways will yield scalable and sustainable routes to the synthesis or semisynthesis of monomers that can be incorporated by variant PylRS/tRNA^Pyl^ pairs.

## 6 Applications of ncAAs Genetically Encoded by PylRS/tRNA^Pyl^ Pairs

Genetic code expansion permits the site-specific introduction of new chemical functionalities into proteins in live cells and animals. This has enabled the scalable production and purification of recombinant proteins bearing defined modifications, and the development of approaches for imaging and controlling protein function in live cells and animals. Excellent overviews of applications based on the genetically encoded site-specific introduction of ncAAs into proteins are available,^1,3,4,62,207−210^ and are beyond the scope of this review. Here we focus on classes of ncAAs and applications that have been uniquely enabled by PylRS/tRNA^Pyl^ pairs. These applications include: (1) encoding ncAAs corresponding to PTMs, particularly lysine PTMs (Section 6.1); (2) caging canonical amino acids for triggered activation of protein function, where PylRS systems have expanded both the range of canonical amino acids that can be caged and, the caging modalities and the range of organisms where caging can be easily applied (Section 6.2); (3) encoding bio-orthogonal groups, where PylRS systems have provided access to aliphatic long chain ncAAs that undergo rapid bio-orthogonal labeling and thereby enabled rapid site-specific protein labeling, and dual labeling, in cells and organisms (Section 6.3); (4) strategies for reversible photocontrol and installing biophysical probes, enabled by rapid bio-orthogonal chemistry or the encoding of photoswitches and probes with PylRS/tRNA^Pyl^ pairs (Section 6.4, 6.5); (5) genetically encoding ncAAs for identifying protein interactions, where PylRS systems provide facile access to cross-linkers in diverse cells and organisms, access to more flexible cross-linking ncAAs that may capture protein interactions more efficiently, and access to new functional groups for cross-linking and new cross-linking modalities (Section 6.6); (6) encoding ncAAs that can be used as mechanism based traps of enzyme substrates, and to augment enzyme function, where PylRS systems provide access to unique chemical functionality (Section 6.7, Section 6.8); (7) strategies for translational control in cells and animals, where PylRS systems benefit from bioavailable ncAAs and enables the approaches to be used in diverse cells and organisms (Section 6.9).

### 6.1 Post Translational Modifications

**Figure 11:**
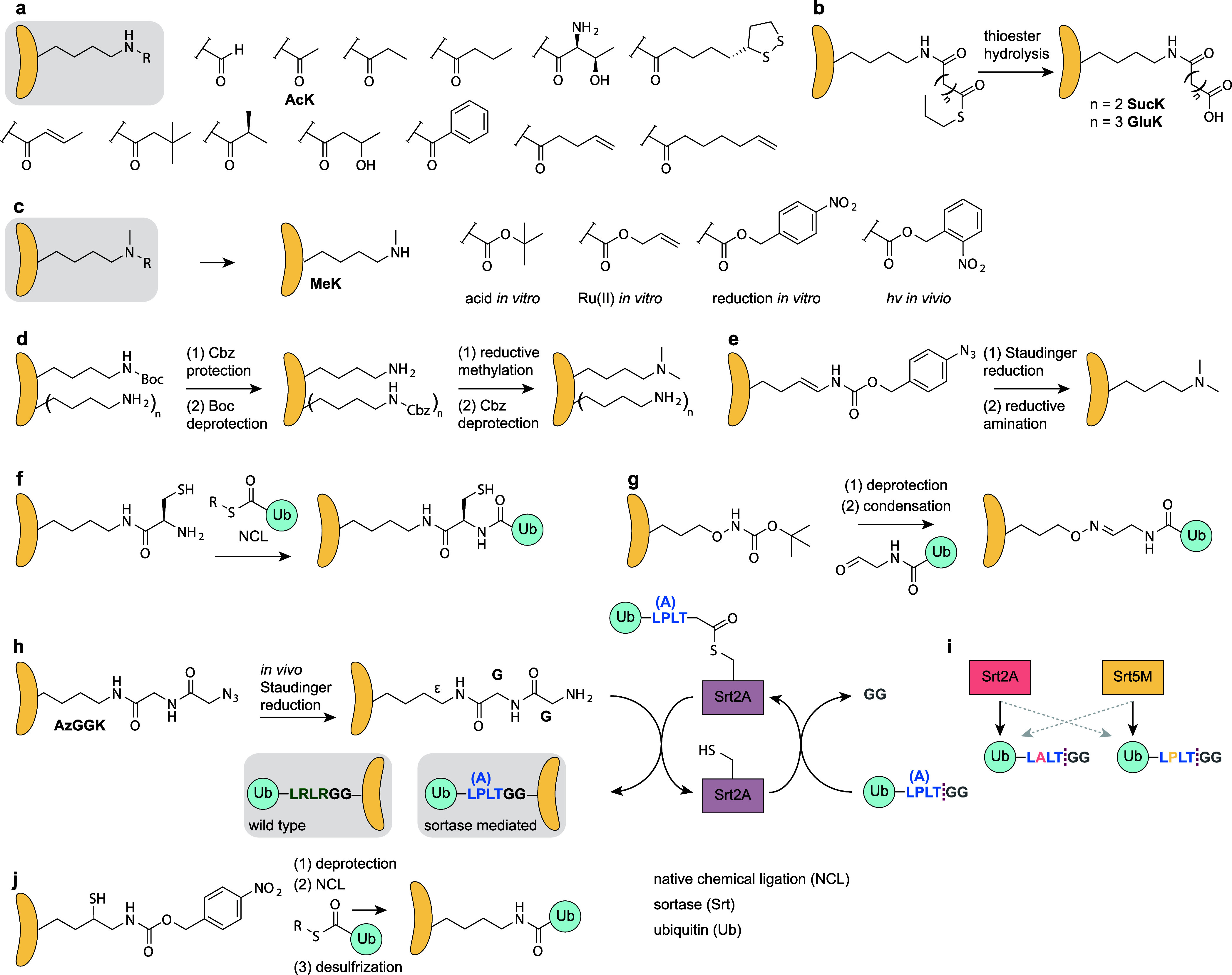
**PylRS-mediated genetic encoding of ncAAs corresponding to post-translationally modified forms of canonical amino acids. a**, Acylated derivatives of lysine for which PylRS variants have been evolved. The structures include non-natural PTM mimetics; trifluoroacetyl-lysine (not shown) can also be incorporated. **b**, Strategy to genetically direct the succinyl-lysine (**SucK**) and glutaryl-lysine (**GluK**) in proteins. **c**, Protected versions of *N*^*ϵ*^-methyl-*L*-lysine (**MeK**), which have been genetically encoded into proteins. Following incorporation, the ncAAs have been deprotected to produce proteins with monomethylated lysine residues at specific sites in proteins. **d, e**, Strategies to generate proteins bearing dimethylated lysine residues. **f to i**, Strategies to generate site-specifically ubiquitinated proteins containing one to two non-natural linkages between lysine and ubiquitin. **j**, Strategy to genetically direct a natural lysine-ubiquitin linkage.

Genetic code expansion permits the direct installation of ncAAs that correspond to the post-translationally modified forms of canonical amino acids, commonly described as “genetically encoded PTMs”. This enables the study of the structural, mechanistic and functional consequence of PTMs at specific sites in proteins. The majority of this work has focused on expressing and purifying recombinant proteins bearing defined PTMs that can be used for structural or mechanistic studies; this approach, along with complementary approaches,^211,212^ addresses the challenge of making recombinant proteins bearing PTMs at defined sites when the enzyme that naturally installs the modification is unknown (common for PTMs identified by proteomics) or modifies other sites in the protein *in vitro*.^82,106,137,143,213−219^ A smaller, but important, body of work has genetically installed PTMs at specific-sites in proteins produced in physiologically relevant hosts, to address the functional consequences of these modifications *in vivo*;^94,95,219−221^ these approaches commonly aim to address the functional consequences of these modifications, without the pleiotropic effects that result from manipulating the enzymes that naturally add or remove the modification. PylRS/tRNA^Pyl^ pairs have provided access to PTMs of lysine (Figure 11), which cannot be accessed by other orthogonal aaRS/tRNA pairs, as well as some PTMs of other canonical amino acids.

The first directed evolution of a PylRS enzyme toward a new substrate focused on introducing **AcK** into proteins in *E. coli*.^82^ The genetic encoding of **AcK** has enabled the consequences of lysine acetylation, on the structure and function of recombinant proteins, to be studied.^220−231^ The genetic encoding of **AcK** has been extended to various hosts, including live animals, enabling studies on the functional consequences of acetylation in a cellular or organismal context.^94,95,106,219−221^ Genetically encoded nonhydrolyzable analogues of **AcK** have provided a stable version of the modification in cells.^232−234^ PylRS variants have been engineered and evolved to encode many other lysine acylations including formylation,^235^ propionylation,^236,237^ butyrylation,^219,236,237^ 2-hydroxyisobutyrylation,^238,239^ β-hydroxybutyrylation,^239^ crotonylation,^236,237,240^ lactylation,^239^ lipoylation,^239^ benzoylation,^241−243^ and threonylation.^244^ Acylated lysine derivatives with long-chain terminal olefins have also been encoded using engineered PylRS enzymes.^239,245^ These approaches have provided a wealth of insight into the diverse roles of lysine acylations (Figure 11a).^219,235−246^ Recently, lysine derivatives bearing protected succinyl- or glutaryl-groups were genetically encoded and deprotected on the protein to succinyl-lysine (**SucK**) and glutaryl-lysine (**GluK**); this approach was used to study the function of these negatively charged PTMs (Figure 11b).^247^ The genetic encoding of an azido-norleucine, followed by a traceless Staudinger ligation, enabled the generation of various acylated lysine derivatives at site-specific positions in ubiquitin and histone H3 with modest efficiency.^248^ Genetic code expansion-based approaches to study lysine acylations have recently been extensively reviewed.^249^

Lysine methylations (mono-, di- and trimethylation) are another important class of PTM that have been tackled by genetic code expansion with PylRS/tRNA^Pyl^ pairs. It was challenging to create an active site that would distinguish between lysine and methylated lysines. For monomethylated lysine researchers addressed this challenge by encoding protected versions of *N*^*ϵ*^-methyl-*L*-lysine (**MeK**), in which the protecting group further differentiated the structure of the ncAA from lysine (Figure 11c).^250^ This concept was demonstrated using a *tert*-butyloxycarbonyl (**Boc**) protecting group, and used to make histone H3 **MeK**9; binding to the methylation reader protein HP1 was demonstrated. A variety of other protected ncAAs have been used to encode **MeK**, and these approaches now enable *in vitro*,^251−253^ or *in vivo* deprotection.^254,255^ Di- and trimethylation have been more challenging to encode, but N^+^-PylRS/N-tRNA^Pyl^ pairs have been used as part of strategies to selectively install dimethyl-lysine in histone H3^256,257^ and p53 proteins (Figure 11d,e).^257^

Ubiquitin and ubiquitin-like proteins constitute an important class of lysine modification that cannot be directly genetically encoded. Several approaches have used N^+^-PylRS/N-tRNA^Pyl^ pairs to encode ncAAs that can be linked to ubiquitin; these approaches complement a variety of other methods developed to access ubiquitinated proteins.^258−262^ Several of these efforts lead to non-native sequence in or around the linkage. Genetically encoding **CεK** followed by reaction with a C-terminal thioester of ubiquitin 1–75, produced protein-ubiquitin conjugates (Figure 11f). However, the native chemical ligation reaction (NCL) resulted in a nonstandard linkage in which G76 of ubiquitin was replaced by cysteine.^263^ Additionally, the genetic encoding of protected ϵ-aminooxy-*L*-lysine derivatives enabled the production of recombinant, isosteric and nonhydrolyzable ubiquitin conjugates (Figure 11g).^264^ An emerging method to generate site-specifically ubiquitinated, and SUMOylated proteins uses an engineered N^+^-*Mb*PylRS/N-*Mb*tRNA^Pyl^ pair to encode *N*^*ϵ*^-((2-azidoacetyl)glycyl)-*L*-lysine (**AzGGK**) into proteins (Figure 11h). Following reduction of the azide to an amine, the protein of interest is reacted with ubiquitin, bearing mutations at positions 72 and 74 that generate a sortase recognition motif, in a sortase-mediated transpeptidation. The resulting protein conjugate has a native isopeptide bond, but contains two mutations in ubiquitin. This approach has been used to make ubiquitinated proliferating cell nuclear antigen (PCNA) and has been extended to SUMOylation, and to ubiquitination in mammalian cells. The mutations in the C-terminus of ubiquitin confer resistance to some deubiquitinating enzymes, but appear to have minimal effects on the binding of ubiquitin binding proteins tested. In addition, these linkages can be formed under nondenaturing conditions.^216^ By using orthogonal sortase enzymes, that recognize distinct C-terminal mutants of ubiquitin, this approach has been extended to the assembly of ubiquitin chains (Figure 11i).^265^ Recently, this strategy has been further expanded to asparagine endopeptidase mediated ligation, which requires only one mutation in the ubiquitin C-terminus.^266^

Several methods have achieved entirely native linkages to ubiquitin. The genetic encoding of protected δ-thiol-*L*-lysine into proteins was achieved with an evolved N^+^-*Mb*PylRS/N-*Mb*tRNA^Pyl^ pair. In combination with chemistry first demonstrated in synthetic peptides,^267^ this enabled the traceless site-specific ubiquitination of recombinant proteins (Figure 11j).^217^ Furthermore, the coordinated use of chemical protection/deprotection schemes, enabled by N^+^-*Mb*PylRS/N-*Mb*tRNA^Pyl^ pair-mediated ncAA incorporation, led to the synthesis of site-specifically linked ubiquitin chains with native linkages; this resulted in the structure of K6 linked diubiquitin and revealed a K29 specific deubiquitinating enzyme.^218^

Several other approaches have been used to generate a variety of native and non-native PTMs. The reaction of nucleophiles with dehydroalanine is a well-established route to installing a wide-variety of PTMs into proteins.^268,269^*Se*-alkylselenocysteine has been encoded using an engineered N^+^-*Mm*PylRS/N-*Mm*tRNA^Pyl^ pair. The encoded ncAA has been converted to dehydroalanine and used to generate various PTM mimetics.^270^ A challenge with dehydroalanine-based approaches is that they commonly lead to racemization of the α carbon to generate a mixture of proteins that includes a stereochemically non-native backbone. The site-specific incorporation of a protected phospho-tyrosine precursor, which can be converted to phospho-tyrosine *in vitro*, under denaturing conditions, enabled the site-specific genetic encoding of the modification into recombinant proteins produced in *E. coli*; this method requires the protein to be refolded.^137^ Halo-tyrosine derivatives have also been site-specifically encoded.^135^ Proteins can also be modified by bio-orthogonal chemistry (Section 6.3) and these approach has been used to generate non-native PTMs, including glycosylation.^271,272^

PylRS systems have been crucial for encoding diverse PTMs, most notably lysine acylations and ubiquitination. The tools provided by these approaches have provided unique insight into the roles of PTMs and combinations of PTMs. Future work may focus on genetically encoding other key modifications including glycosylation and tyrosine phosphorylation, and on the use of genetically encoded PTMs and their nonremovable analogs to interrogate and control the state of living cells and organisms.

### 6.2 Controlling Protein Function by Caging Canonical Amino Acids

**Figure 12:**
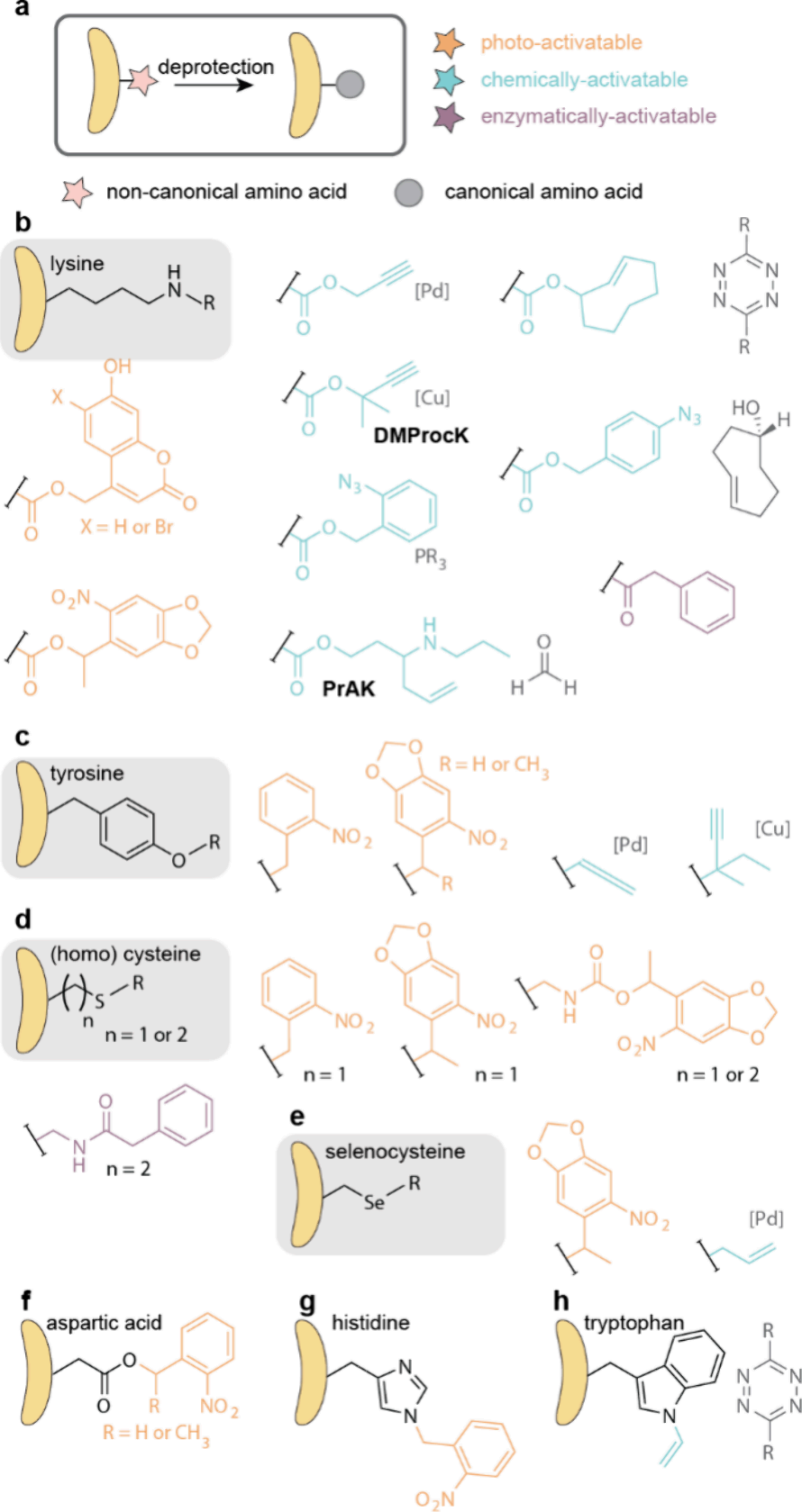
Non-canonical amino acids, corresponding to caged canonical amino acids, that can be genetically encoded into proteins and deprotected to the corresponding canonical amino acids. **a**, Schematic representation of the deprotection of ncAAs, corresponding to caged canonical amino acids, to canonical amino acids. The deprotection can be done with light (orange structures in panels **b**-**h**), by the addition of a small molecule (blue structures in panels **b**-**h**, with deprotecting agent in gray), or enzymatically (purple structures). **b** to **h** Genetically encoded ncAAs that can be deprotected to lysine, tyrosine, (homo)-cysteine, selenocysteine, aspartic acid, histidine and tryptophan derivatives, respectively.

Activatable proteins can be created by the site-specific introduction of protected versions of canonical amino acids, in place of the corresponding canonical amino acids, at positions that mediate an important function of a protein (Figure 12a). The protected amino acid is converted to a canonical amino acid in the protein, by optical, chemical or enzymatic activation, to restore the native protein sequence and function. These experiments are commonly performed using transgenes and are commonly applied to proteins that provide new activities to the cell (e.g.: cre recombinase or TEV protease), or constitutively active versions of native proteins (e.g.: constitutively active kinase mutants).^273−275^ Non-pyl aaRS/tRNA pairs have been used to encode photocaged amino acids and generate photoactivatable proteins: photocaged tyrosine and cysteine have been encoded in *E. coli*, photocaged serine has been encoded in yeast, and photocaged glutamate has been encoded in yeast and in mammalian cells.^276−279^ The development of PylRS/tRNA^Pyl^ pairs for genetic code expansion enabled facile photocaging, and photoactivation, of proteins in mammalian cells, and in some cases animals, for lysine, tyrosine, cysteine, aspartic acid and histidine residues.^136,138,153,280−284^ Optical decaging is commonly fast, can be executed with millisecond pulses of (blue (410 nm) or UV (365 nm)) light at powers that do not trigger optical DNA damage responses, and can be spatially controlled. Optical decaging may be difficult to achieve in deep tissue samples, but in some cases 2-photon approaches have been developed to address this challenge. PylRS/tRNA^Pyl^ pairs have also enabled the caging and chemical decaging of lysine, tyrosine, selenocysteine and tryptophan residues. Chemical decaging commonly occurs over time scales of minutes to hours and may not go to completion, is commonly not spatially confined, but may be easier to effect in deep tissues.

#### 6.2.1 Optical Decaging of ncAAs Encoded Using PylRS/tRNA^Pyl^ Pairs

The N^+^-*Mb*PylRS/N-*Mb*tRNA^Pyl^ pair was evolved to incorporate a photocaged derivative of lysine (Figure 12b); this ncAA was first used in place of key lysine residues in a nuclear localization sequence to enable the optically triggered nuclear localization of target proteins from the cytosol to the nucleus of mammalian cells.^153^ The genetic encoding of photocaged lysine has been extended to generate photoactivatable enzymes, including kinases,^285,286^ polymerases,^287^ DNA helicases,^288^ and recombinases^98,165,289^ for spatially and temporally controlled activation, and lysine derivatives that can be deprotected with two-photon optics have been developed.^280^ A recent exciting application of genetically encoded photocaged lysine was the optical activation of cre recombinase in one of a pair of bilaterally symmetric neurons in *Caenorhabditis elegans* (*C. elegans*); these neurons cannot be genetically distinguished.^98^ This enabled the cre-mediated activation of expression of channelrhodopsin in a single neuron and subsequent optogenetic stimulation of a single neuron in freely moving animals.^98^ These experiments provided an approach that enabled the unique contributions of single neurons, within bilaterally symmetric pairs, to be determined.

The N^+^-*Mb*PylRS/N-*Mb*tRNA^Pyl^ pair has been evolved to incorporate photocaged derivatives of tyrosine (Figure 12c).^136,138^ Encoding a photocaged tyrosine in place of tyrosine at a phosphorylation site in STAT1 in mammalian cells, enabled the tyrosine phosphorylation site to be blocked. The phosphorylation site was revealed by optical activation leading to light-induced phosphorylation of the tyrosine residue. Photocaged tyrosine has also been used to activate kinases,^290^ and proteases,^136^ including caspases.^290^ Anthrax toxin component lethal factor has been caged for optical pro-drug activation.^290^

N^+^-*Mb*PylRS enzymes have also been engineered for genetically encoded incorporation of photocaged cysteine and homocysteine (Figure 12d).^281,282^ Encoding photocaged cysteine enabled the creation of photoactivated TEV protease in mammalian cells. The development of a photocaged selenocysteine enabled the incorporation of selenocysteine into proteins without using the selenocysteine insertion sequence (SECIS) normally required in mRNAs for the incorporation of selenocysteine (Figure 12e).^291^

Engineered N^+^-*Mb*PylRS/N-*Mb*tRNA^Pyl^ pairs have been used to encode a photocaged aspartic acid for optically activating kinase and GTPase activity in mammalian cells (Figure 12f).^283^ Engineered N^+^-*Mb*PylRS/N-*Mb*tRNA^Pyl^ pairs have also been developed to encode a photocaged histidine, which was used to optically activate luciferase in mammalian cells (Figure 12g);^284^ this is an exciting development and, following refinement of the current system, this advance may enable the photocaging of many enzyme activities and binding activities mediated by histidine residues.

#### 6.2.2 Chemical Decaging of ncAAs Encoded Using PylRS/tRNA^Pyl^ Pairs

PylRS/tRNA^Pyl^ pairs have been used to site-specifically incorporate ncAAs in which a lysine residue is connected through a carbamate linkage to functional groups to form a protecting group; the protecting group reacts with added chemicals to reveal the lysine residue (Figure 12b). Several chemistries have been used to effect this deprotection including palladium catalyzed reactions with encoded alkynes,^292^ the reaction of tetrazines with encoded *trans*-cyclooctenes (**TCO**s),^293−297^ Staudinger reductions with encoded ortho-azido benzyl groups,^298^ the reaction of **TCO**s with encoded para-azido benzyl groups,^299^ the aza-Cope reaction of *N*^*ϵ*^-((prop-2-yn-1-yloxy)carbonyl)-*L*-lysine (**PrAK**) with formaldehyde–providing the basis for a formaldehyde sensor,^300^ and the reaction of *N*^*ϵ*^-(((2-methylbut-3-yn-2-yl)oxy)carbonyl)-*L*-lysine (**DMProcK**) with Cu(I)-BTTAA.^301^ With the exception of the copper-triggered approach, which was only used to activate proteins on cell surfaces, these approaches have been used to activate protein function in live cells.

N^+^-*Mm*PylRS/N-*Mm*tRNA^Pyl^ pairs have been used to site-specifically incorporate ncAAs corresponding to protected tyrosine residues (Figure 12c). An allene ether derivative of tyrosine was incorporated into proteins and deprotected to tyrosine using palladium reagents in mammalian cells.^302^ Similarly, a disubstituted propargyl group caged tyrosine was incorporated into proteins and deprotected to tyrosine with Cu(I)-BTTAA.^301^ The palladium-triggered approach has been used to activate protein function in live cells, while the copper-triggered approach was demonstrated on cell surfaces. An engineered N^+^-*Mb*PylRS/N-*Mb*tRNA^Pyl^ pair has enabled the palladium mediated deprotection of site-specifically installed *Se*-allyl-selenocysteine in *E. coli*.^303^

Recently a N^1^-vinyl tryptophan was encoded using a derivative of chPheRS/chtRNA^Phe^.^144^ This ncAA was deprotected in cells through an inverse electron demand Diels–Alder reaction with tetrazines (Figure 12h).^146^ This approach was used to regulate a variety of protein activities and protein interactions.

Enzymatic routes have also been explored for the deprotection of ncAAs (Figure 12b,d).^304,305^ A phenylacetamidomethyl protected derivative of homocysteine was site-specifically incorporated into proteins using an engineered N^+^-*Mb*PylRS/N-*Mb*tRNA^Pyl^ pair and deprotected using penicillin G acylase, as part of a strategy for protein dual labeling.^305^ While enzymatic deprotection has the potential to be substantially faster than current small molecule based deprotections on proteins it may be more restricted in the sites on proteins at which it can mediate deprotection.

In future work it will be interesting to see if chemical deprotection can be accelerated to control processes that occur on a wider range of biological time scales, and to develop approaches to spatially localize chemical deprotection.

### 6.3 Genetically Encoding Bio-orthogonal Groups for Site-Specific Labeling of Proteins

**Figure 13:**
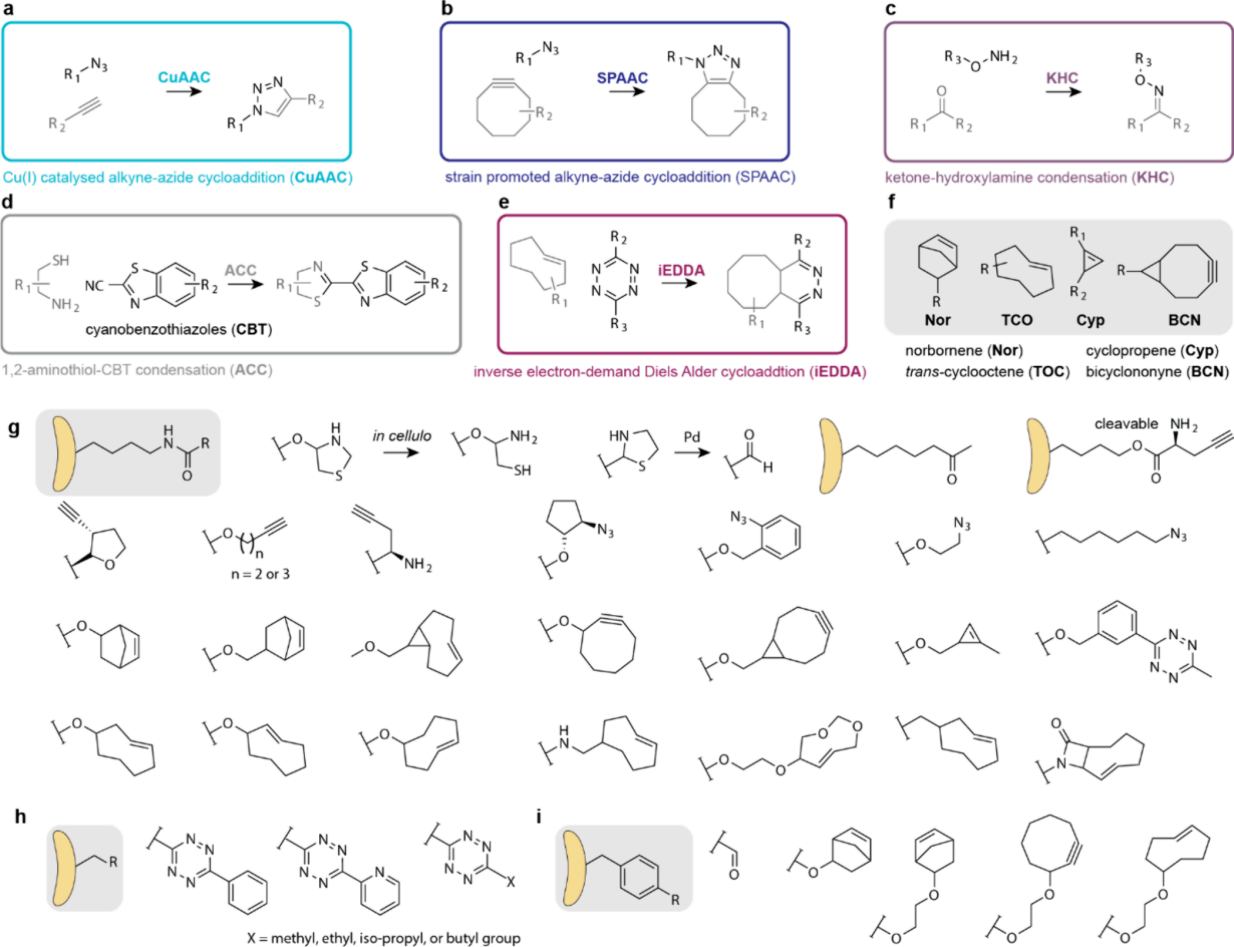
**Bio-orthogonal handles that have been genetically encoded using (engineered) PylRS/tRNA**^**Pyl**^**pairs for protein labeling. a** to **e**, Schematic representations of some commonly used bio-orthogonal reactions for genetic code expansion mediated site-specific labeling of proteins. (**a**) CuAAC, (**b**) SPAAC, (**c**) KHC, (**d**) ACC, and (**e**) iEDDA. **f**, Structure of the some of the most-important bio-orthogonal handles used to date. Noncanonical amino acids bearing **Nor**, **TCO**, **Cyp**, as well as **BCN** groups have all been genetically encoded using engineered PylRS/tRNA^Pyl^ pairs and extensively used for iEDDA-based labeling, and SPAAC-based labeling. **g**, List of lysine derivatives bearing a variety of bio-orthogonal handles including azides, ketones, tetrazine, strained alkene, and (strained) alkynes. **h**, As in (**g**) but for alanine derivatives. **i**, As in (**g**) but for phenylalanine derivatives. Parts of this figure are reprinted with permission from Lang, K.; Chin, J. W. Bioorthogonal Reactions for Labeling Proteins. ACS Chem. Biol. 2014, 9, 16–20 - copyright © 2014 American Chemical Society.

Genetic code expansion enables the site-specific installation of ncAAs bearing bio-orthogonal groups that can be selectively labeled with molecules bearing a bio-orthogonal reaction partner (Figure 13a to f). This paradigm substantially expands the range of chemical functionalities that can be attached to proteins and has provided routes to labeling proteins with molecules for imaging, controlling, and augmenting protein function; these approaches have provided new insight into basic biology and new approaches to generating defined protein modifications for a variety of applications, including the generation of potential therapeutics.^207,208,306−313^ PylRS/tRNA^Pyl^ pairs have enabled the encoding of ncAAs with aliphatic side chains bearing bio-orthogonal groups (Figure 13g).

PylRS/tRNA^Pyl^ pairs have been used to genetically encode lysine derivatives bearing azides and alkynes.^58,314−320^ Some labeling of azides can be achieved using Staudinger ligations, but these are slow and often do not go to completion. Azides and alkynes can be labeled by Cu(I) mediated chemistry, this is faster and can be driven to completion, but the reaction conditions may cause oxidative damage of proteins (Figure 13a,g,i).^58,314−319,321,322^ Encoded azides may also be labeled with ring strained alkynes,^320^ but these reactions are again slow (Figure 13b,g).^207,323,324^ Genetically encoded alkynes have been used for palladium mediated protein labeling with iodophenyl reagents,^325,326^ and ruthenium(II) mediated hydrosilylation.^327^ Genetically encoded fluorosulfates have been developed for palladium mediated protein labeling by Suzuki cross-couplings.^328^ Genetically encoded alkynes have been used for thiol–yne reactions with reactive thiols,^329^ and genetically encoded alkenes have been labeled with thiol–ene reactions;^330,331^ the thiol reactants in these reactions are commonly not bio-orthogonal. A ncAA in which an alkyne is linked to lysine through a hydrolyzable ester has enabled the capture and release of a protein in which it is encoded.^332^ Noncanonical amino acids bearing ketones,^234^ and (caged) aldehydes,^333,334^ have been incorporated with PylRS/tRNA^Pyl^ pairs and used for labeling with hydroxylamine reagents, and acrylamide functionalities have also been encoded for polymerization (Figure 13c,g,i).^88^ Genetically encoded cyclopropenones have been encoded for the reaction with phosphines.^335^ A quadricyclane containing ncAA has been encoded and reacted with nickel bis(dithiolene) in a formal [2σ + 2σ + 2π] cycloaddition.^336^ Biotin analogs have also been encoded.^337^

The rate and specificity of bio-orthogonal reactions is crucial for labeling in living systems.^208^ Encoding components of fast, metal free, bio-orthogonal reactions has been essential for achieving efficient and quantitative labeling of recombinant proteins and for achieving labeling on and in live mammalian cells. Labeling proteins within cells is generally considered more challenging than labeling on the cell surface, in part because the fluorophore label must enter the cell, but excess fluorophore needs to be washed out. Engineered PylRS/tRNA^Pyl^ pairs have been central to encoding ncAAs bearing components of rapid bio-orthogonal reactions with on protein rate constants greater than 1 M^–1^ s^–1^, and have enabled the site-specific labeling of proteins in diverse systems, including mammalian cells.

1,2-Aminothiols^338^ have been genetically encoded with N^+^-*Mm*PylRS/N-*Mm*tRNA^Pyl^ pair variants, and used for rapid labeling with cyanobenzothiazoles (**CBT**s); this chemistry uses free thiols and its primary utility is for *in vitro* labeling (Figure 13d,g). Components of inverse electron demand Diels–Alder reactions between strained (or otherwise activated) alkenes or alkynes and tetrazines have been genetically encoded (Figure 13e,g,h,i). Noncanonical amino acids containing norbornenes (**Nor**s),^53,323,339−341^ cyclopropenes (**Cyp**s),^166,342,343^ bicyclononynes (**BCN**s)^197,343,344^ and **TCO**s,^197,323,341,343−347^ other reactive alkenes,^348−350^ and isocyanides^351^ have been genetically encoded using PylRS/tRNA^Pyl^ pairs (Figure 13f,g,i). Genetically encoding a **Nor** containing ncAA with a N^+^-*Mb*PylRS/N-*Mb*tRNA^Pyl^ pair enabled the first labeling of a genetically encoded ncAA in mammalian cells.^339^

Genetically encoded **BCN**s and **TCO**s enabled rapid labeling of proteins in *E. coli* and mammalian cells with tetrazine probes, that exhibit turn-on fluorescence, in seconds to minutes.^197^ These approaches demonstrated that, despite the large number of endogenous amber codons in mammalian cells, target proteins could be specifically labeled.^192,197^ The bio-orthogonal labeling of genetically installed **Cyp**, **BCN** and **TCO** ncAAs have been further improved, and extensively used to image and control protein function (Figure 13g,i) (refs (157, 161, 166, 167, 192, 271, 309, 344, and 352−383)).

Reactive tetrazines have recently been encoded using engineered N^+^-*Mb*PylRS/N-*Mb*tRNA^Pyl^ pairs, enabling this class of ncAAs to be encoded in *E. coli* and mammalian cells (Figure 13g,h).^384−387^ Encoded tetrazines may also be reacted with cyclopropenone-caged dibenzoannulated bicyclononynes (photo-**DMBO**), following ultraviolet (UV) illumination.^384^ Noncanonical amino acids bearing strained alkenes have also been encoded, using engineered PylRS/tRNA^Pyl^ pairs, for “photoclick reactions”: 2 + 3 cycloadditions with hydrazonoyl chlorides generated *in situ* by illumination of tetrazoles with UV light. Photoclick reactions have been demonstrated with encoded **Nor**s, **Cyp**s, (spirocyclic) alkenes,^97,388,389^ and the phototransducing dibenzo[b,f][1,4,5] thiadiazepine.^390^

**Figure 14:**
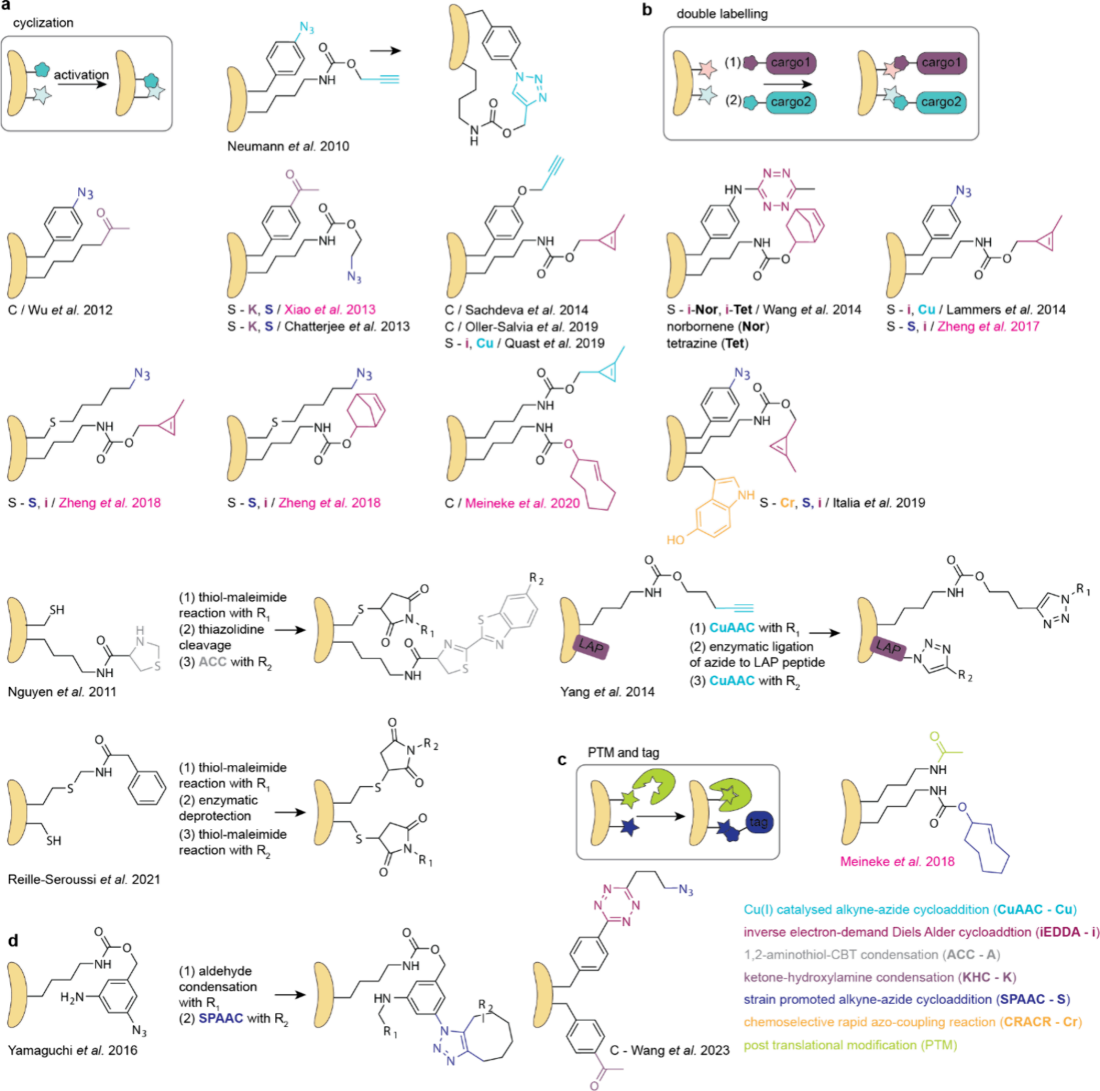
**Site-specific double labeling of proteins. a**, Schematic representation of a protein cyclization mediated by encoded ncAAs containing bio-orthogonal groups, together with the chemistry used in the first genetically programmed, ncAA-mediated, protein cyclization. **b**, Schematic representation of protein double labeling, distinct encoded ncAAs (stars) are labeled with complementary functional groups in sequential or one pot, concerted labeling reactions. Examples of protein double labeling are shown. The bio-orthogonal handles are colored with respect to the bio-orthogonal reaction for which they were used in the initial examples: ACC (gray), CuAAC (light blue), KHC (purple), iEDDA (red), SPAAC (blue), and CRACR (orange). Labeling was sequential (S) or concerted (C). The exact reaction partners and conditions for each reaction are provided in the indicated references and we note that the mutual orthogonality of many reactions will rely on the exact molecules used, and the reaction conditions. For sequential labeling the order of labeling is indicated. Citations labeled in describe reactions performed in mammalian cells. **c**, Schematic representation of the encoding of a PTM together with a bio-orthogonal handle; the ncAA corresponding to a post translationally modified canonical amino acid (green star) can bind its specific readers (green blob) and the PTM containing protein can be enriched using bio-orthogonal reactions. A specific example, which was established for potential applications in mammalian cells is shown. **d**, Genetically encoding single ncAAs bearing two bio-orthogonal handles for protein double, and triple labeling. The bio-orthogonal handles are colored with respect to the bio-orthogonal reaction for which they were used: KHC (purple), iEDDA (red) and SPAAC (blue). Labeling order of functional groups is specified when not concerted (C).

Strategies for cyclizing proteins by encoding two distinct ncAAs with compatible bio-orthogonal groups (Figure 14a), labeling recombinant proteins at two to three distinct sites (Figure 14b), encoding one PTM together with a ncM for labeling (Figure 14c), as well as the labeling of two distinct proteins within a cell, have commonly used PylRS to encode a bio-orthogonal group at one site.^76,88,155,305,338,344,380,391−407^ Single ncAAs containing two distinct functionalities (an amine and an azide, or an azide and a tetrazine, respectively) (Figure 14d) have also been encoded and used for dual,^408^ or triple (together with a genetically encoded ketone bearing ncAA)^409^ labeling of proteins. Strategies to site-specifically dual label proteins by genetic code expansion have recently been reviewed.^410^

### 6.4 Optical Switching of Protein Function in Live Cells

The optical switching of protein function provides a route for reversible control. Noncanonical amino acid–based approaches to optical switching have focused on attaching photoisomerizable groups (notably azobenzene) to proteins, and coupling isomerization of the azobenzene to a change in the state of the protein. In photobio-orthogonal ligand tethering (photo-BOLT), **a BCN** containing ncAA was encoded proximal to the active site of a kinase expressed in mammalian cells. A tetrazine-azobenzene-kinase inhibitor conjugate was tethered to the protein through a bio-orthogonal reaction, and illumination at different wavelengths of light was used to repeatedly turn the kinase on and off in live cells. Building on prior work,^411^ the direct genetic encoding of ncAAs containing azobenzenes has also been achieved using engineered PylRS/tRNA^Pyl^ pairs.^412−414^ Azobenzenes bearing halo-alkane substitutions have also been encoded; these have been reacted with a cysteine residue proximal to the site of encoding to form a covalent bridge, and photoisomerization allowed conformational switching *in vitro*.^413,415^ Red shifted azobenzenes containing ncAAs were developed and,^414^ several of these azobenzenes were directly encoded and used to reversibly control luciferase activity in live mammalian cells.^412^ These experiments highlighted the challenges of identifying sites in the protein at which isomerization of the azobenzene side chain led to marked changes in protein activity; these challenges might be addressed by developing computational approaches to predict positions in proteins at which photoswitchable amino acids could be used to control protein activity. The encoding of photoswitchable ncAAs has the potential to enable the reversible control of diverse protein activities *in vitro* and *in vivo*.

### 6.5 Biophysical Probes

Biophysical probes, including coumarin and acridonyl-based fluorophores.^280,416,417^ and nuclear magnetic resonance (NMR),^139,418,419^ electron paramagnetic resonance (EPR),^420^ and Raman spectroscopy^349^ probes have been site-specifically incorporated using PylRS/tRNA^Pyl^ pairs. Noncanonical amino acids have also been incorporated into nanopores to alter their sensing properties.^421^ These approaches complement and extend the functionalities that can be added to proteins by installing ncAAs bearing bio-orthogonal groups and subsequent derivatization (Section 6.3). The direct installation of fluorophores typically requires high concentrations of fluorescent ncAAs to be added to cells, and the fluorophores that can be encoded are commonly excited with light in the blue region of the electromagnetic spectrum.^280,416,417^ The range of fluorophores that can be attached by rapid bio-orthogonal chemistry, and the lower concentrations of added fluorophores required for bio-orthogonal labeling, have made bio-orthogonal labeling of proteins the preferred route for most ncAA-mediated fluorescent labeling of proteins in cells;^208^ this approach has enabled the labeling and imaging of proteins that cannot be tagged in a functional form with fluorescent protein fusions, precise labeling of proteins for super resolution imaging, and the construction of fluorescence resonance energy transfer (FRET) probes for following protein conformational changes in live cells.^192,309,323,339,343,344,370,399,400^ Fluorinated amino acids have been encoded to provide a unique signal for F19 NMR and a TMS containing ncAA has been encoded.^139,418,419^

### 6.6 Genetically Encoding Cross-Linkers

**Figure 15:**
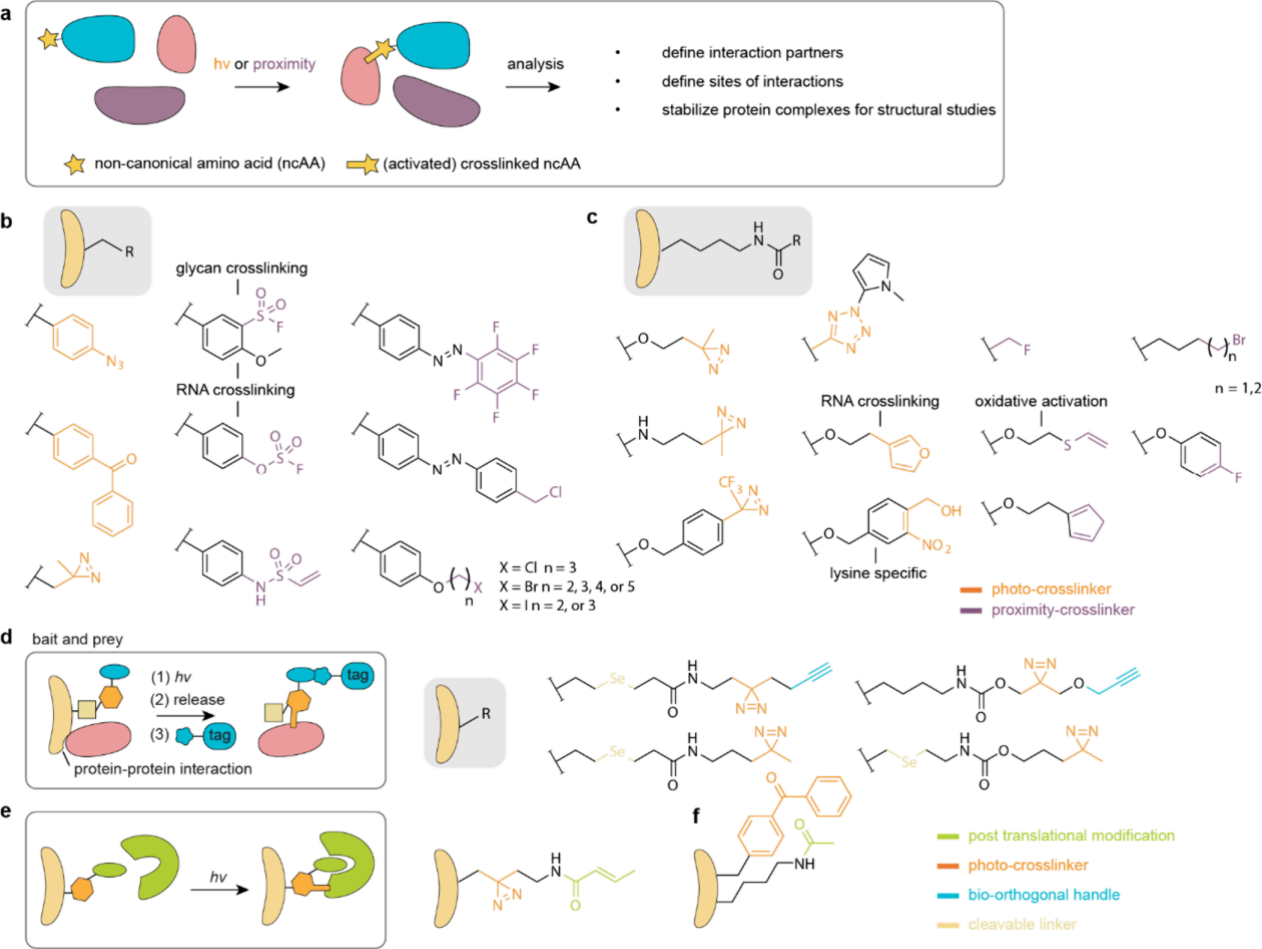
**Protein cross-linking using genetically encoded ncAAs. a**, Schematic representation of method to identify protein–protein interactions by genetic code expansion mediated site-specific installation of cross-linking ncAAs. Proteins bearing an either photo- (orange), or proximity- (purple) inducible cross-linking ncAA are used in cells to capture interaction partners. The cross-linked proteins are analyzed (by gel electrophoresis or mass spectrometry-based methods) to define interaction partners of the target proteins and the sites of interaction. In some cases cross-links between specific proteins have been used to stabilize complexes for structural studies. **b**, Phenylalanine derived cross-linking ncAAs incorporated by engineered PylRS variants. **c**, Lysine derived cross-linking ncAAs incorporated by engineered PylRS variants. **d**, Schematic representation of the bait and pray concept for a trifunctional ncAA; the bait protein, yellow, contains a ncAA with a cleavable linker, a photo cross-linking functionality and a bio-orthogonal handle. Illumination activates the cross-linker to cross-link to a prey protein, the bait protein is released to facilitate analysis of the resulting stump on the prey protein, and the bio-orthogonal handle is used to pull down the covalent bait-prey complex for analysis by MS. Examples of ncAAs used for this approach, in some cases the site of cleavage at Se is also used for capture and enrichment. **e**, Schematic representation of a bifunctional ncAA containing both a photo-cross-linking functionality and a PTM; the PTM interacts with a binding protein which is covalently captured, upon illumination, by cross-linking. **f**, Distinct ncAAs bearing photo-cross-linkers and PTMs of canonical amino acids can be encoded in a single protein; this provides an alternate route to capturing PTM specific interactions.

The site-specific installation of ncAAs with side chains bearing photo-cross-linking functionalities has provided powerful approaches to map protein–protein interactions *in vitro* and *in vivo*,^209,422−424^ and to stabilize protein complexes for structural studies (Figure 15a).^425,426^ These ncAAs contain functional groups (aryl azides, diazirines, benzophenones) that, upon UV illumination, can be converted to reactive species (nitrenes, carbenes, triplet diradicals) that react (through insertion into C–H, or heteroatom-H bonds) with adjacent molecules to form covalent bonds; the cross-linking is commonly analyzed by following the gel electrophoretic mobility shift of the protein containing the cross-linking moiety, and mass spectrometry.

**Bpa**, and *p***-AzF** were incorporated with engineered *Mj*TyrRS/*Mj*tRNA^Tyr^ pairs in *E. coli* and with engineered *Ec*TyrRS/*Ec*tRNA^Tyr^ pairs in eukaryotic cells (Figure 15b);^109,110,427^ these ncAAs have subsequently been incorporated using N^+^-*Mm*PylRS variants.^127,130^ PylRS/tRNA^Pyl^ pairs have uniquely enabled the incorporation of lysine derivatives bearing diazirines (Figure 15c).^105,428−431^ Unlike benzophenones, which can be reversibly photoexcited and have minimal reaction with solvent, the carbenes resulting from photoactivation of diazirines are formed irreversibly and may react nonproductively with solvent. Nonetheless, lysine derivatives bearing diazirines have proved popular because the cross-linking group is small and may minimally perturb protein interactions, and because the longer, flexible lysine side chain may enable the cross-linker within the bait protein to reach suitable reactive groups on the interacting prey proteins. Following the first genetic encoding of a diazirine containing lysine derivative, and the demonstration of its utility for photo-cross-linking protein interactions *in vitro* and *in vivo*,^428^ diazirine containing lysine derivatives have been extensively used for the study of protein interactions.^166,167,431−437^ A diazirine containing ncAA with a shorter side chain has been genetically encoded, and may be used in future cross-linking studies.^438^ Lysine derivatives bearing tetrazoles have also been used for cross-linking,^439^ and lysine derivative bearing furans have been reported to cross-link to RNA following red light activation.^440^ An *o*-nitrobenzyl alcohol derivative of lysine has been encoded with an engineered N^+^-*Mm*PylRS/*Mm*tRNA^Pyl^ pair for the residue-selective-photo-cross-linking of proteins.^441^

Identifying cross-linked proteins, and the sites of cross-linking, by MS can be challenging. Therefore, researchers have aimed to develop methods that, following cross-linking, allow cleavage of the covalent linker between the bait and prey proteins and thereby label the prey protein with a mass tag (Figure 15 d). To achieve this, researchers have created lysine derivatives bearing a diazirine, in which selenium atoms were used to replace the gamma or delta carbon atom of lysine. Following photo-cross-linking, selenium–carbon bonds were oxidatively cleaved and the resulting selenic acid was used as a handle for enriching modified bait proteins. In an alternative design, oxidative cleavage leads to a N-(4,4-bis-substituted-pentyl)acrylamide (NPAA) moiety in the prey protein, which can be readily identified by MS.^442−445^ Alternatively, the cross-linker was extended to include an alkyne functionality, which enabled cleaved bait proteins to be enriched through bio-orthogonal reactions with azide-biotin.^407^ Bifunctional ncAAs containing a diazirine and an alkyne moiety were used for photo-cross-linking and labeling of proteins^446^ and to cross-link proteins to RNA.^447^

Identifying PTM dependent protein interactions is an outstanding challenge. To address this challenge for lysine crotonylation, researchers developed ncAAs in which the diazirine functionality was attached to the gamma carbon of crotonyl lysine, distal from the crotonyl group (Figure 15e). This ncAA (and a matched protected lysine control) was encoded using a N^+^-*Mm*PylRS/*Mm*tRNA^Pyl^ pair and used to trap protein interactions.^448^

PTMs and cross-linkers have also been encoded in two distinct ncAAs in a protein in *E. coli* (Figure 15f).^449^ Another study incorporated **BpA** and a photocaged tyrosine into an epidermal growth factor receptor (EGFR)-targeting antibody fragment (7D12). The binding of 7D12 to EGFR was dependent on the optical decaging of photocaged tyrosine, and the covalent linkage of 7D12 to EGFR was also induced by light.^450^

Noncanonical amino acids bearing electrophilic chemical moieties that undergo reactions with nearby nucleophiles provide a complement to photo-cross-linking approaches (Figure 15a,b,c).^209,451^ In contrast to photo-cross-linking approaches, these approaches require the ncAA to be incorporated in proximity to a nucleophile with which the cross-link is formed. Engineered PylRS enzymes have enabled the site-specific genetic encoding of (activatable) Michael acceptors,^452−454^ aryl carbamates,^455^ dienes,^456^ and various halides.^413,415,457−463^ Early examples that used halides for cross-linking focused on intramolecular protein stapling and cross-linking high affinity protein–protein interactions;^457−459^ later a bromo alkane derivative of phenylalanine was used to study interfaces within protein complexes.^464−466^ Lysine derivatives, with longer chain bromo alkane appendages of C4–C7, enabled the *in vivo* stabilization of a protein complex with a dissociation constant of 10–30 μM, and enabled structural studies.^460,467,468^ Aryl fluorosulfate containing ncAAs, initially developed for the proximity induced reaction with lysine, histidine and tyrosine residues^461^ and used to covalently label protein drugs,^469^ undergo proximity-induced sulfur-fluoride exchange with the 2’ hydroxyl of the RNA backbone, and display some selectivity for RNA in cells.^462^ These ncAAs also show some selectivity for glycans on cell surfaces.^470^

### 6.7 Mechanism Based Traps for Interrogating Enzyme Function

**Figure 16:**
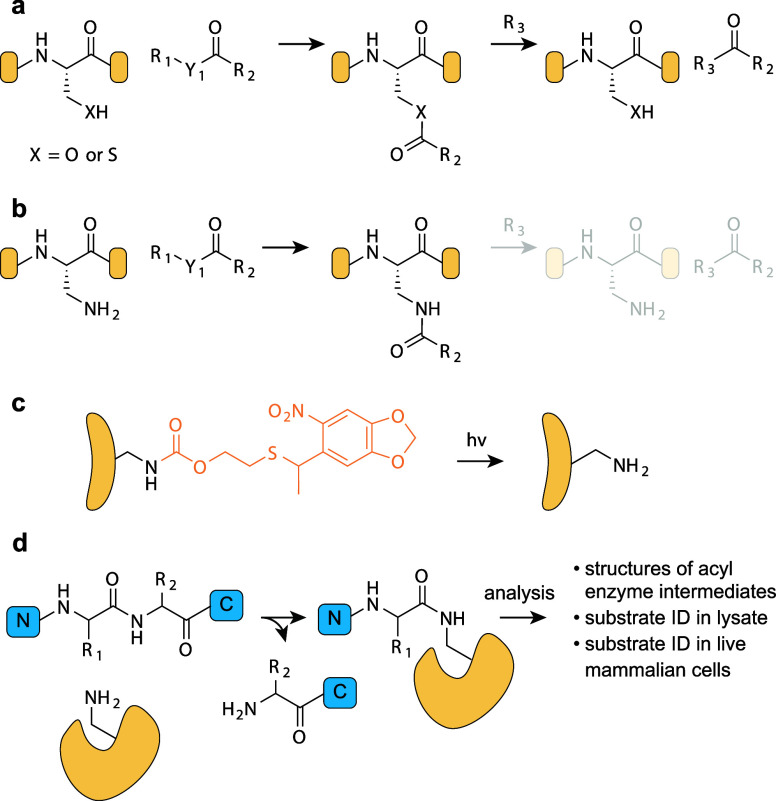
**Trapping acyl-enzyme intermediates by PylRS mediated genetic encoding of photocaged 2,3-diaminopropionic acid** (**pcDap). a**, Active site serines or cysteines react with the carbonyl groups of their target forming an acyl-enzyme intermediate. The active enzyme is regenerated by the nucleophilic substitution of the intermediate with a hydroxyl, amine, or thiol functionality (R3). **b**, By introducing **Dap**, instead of the catalytic cysteine or serine, a cleavage resistant acyl-intermediate may be formed. **c**, Light activation of a genetically encoded **pcDap** to **Dap**. **d**, Genetically encoding **pcDap** and conversion to **Dap** in target proteins enables the N-terminal fragment of protein and peptide substrates (or the analogous portion of other classes of substrate molecules) to be covalently captured. *In vitro* experiments with defined substrates enables structural studies of acyl-enzyme intermediates. Experiments in cell lysates or live mammalian cells, using tagged hydrolases, enable substrate identification by MS. Panels **a** and **b** adapted with permission from Huguenin-Dezot et al.^471^ - copyright © 2018 Springer Nature Limited.

Many enzymes, including serine hydrolases, cysteine proteases and the ubiquitination machinery, react with their substrates through serine or cysteine nucleophiles to form acyl-enzyme intermediates. These ester or thioester intermediates are unstable and commonly have half-lives of seconds to minutes (Figure 16a). In contrast to esters or thioesters, amides are exceptionally stable (Figure 16b). It was hypothesized that replacing serine or cysteine residues with 2,3-diaminopropionic acid (**Dap**), in which an amino group replaces the hydroxyl or thiol group, would create “enzymes” that could form a stable amide link with substrates. **Dap** would be hard to genetically encode, due to its similarity to serine and cysteine, therefore a photocaged version of **Dap** (**pcDap**) was designed, and a N^+^-*Mb*PylRS/N-*Mb*tRNA^Pyl^ pair evolved to incorporate this ncAA (Figure 16c).^471^ The incorporation of **pcDap** into recombinant proteins (and the post-translational deprotection of **pcDap** to **Dap**) has been used to generate stable intermediates for structural studies. Incorporating **Dap** into the thioesterase domain of a nonribosomal peptide synthase enabled a series of stable acyl-enzyme “intermediates” to be captured and structurally characterized, providing structural insight into how the nonribosomal peptide synthetase controls peptide elongation and cyclization to make a defined macrocycle (Figure 16d).^471^ By adding a **Dap** containing protease to a cell lysate, putative substrates of the protease were selectively captured. Genetically encoded **pcDap** has also been used to capture hydrolase substrates in live mammalian cells (Figure 16d). Following the encoding of **pcDap** in place of the catalytic residue of a membrane embedded protease (RHBDL4), an enzyme trap was photoactivated in mammalian cells and RHBDL4 substrates were identified. Encoding **pcDap** into RBBP9, an orphan hydrolase in mammalian cells, was used to discover that it is an aromatic amino-peptidase.^472^ Extensions of this approach enabled protein fusions to a newly discovered PETase to be coupled to polyethylene terephthalate (PET).^473^ It seems likely that this approach will be extended to discover the substrates of diverse hydrolases and to make further useful protein conjugates.

### 6.8 Altering Enzyme Function

Genetic code expansion has been used to augment and probe enzyme function.^133,474−486^ The genetic encoding of proximal ligands with new-to-nature chemical properties in heme proteins has emerged as an efficient method to tune the enzymatic properties of heme enzymes. The genetic encoding of 3-*N*-methyl histidine (**NmH**) into an engineered ascorbate peroxidase, using a N^+^-*Mb*PylRS/N-*Mb*tRNA^Pyl^ pair increased the turnover number of the enzyme up to 5-fold.^482^ Genetically encoding **NmH** in place of an iron coordinating histidine in myoglobin increased peroxidase activity 4-fold, and subsequent directed evolution and screening led to a protein with a peroxidase activity a thousand-fold higher than myoglobin. Myoglobins containing **NmH** also enabled cyclopropanation of styrene in the absence of reductant and under aerobic conditions, and a variety of histidine analogs were incorporated into myoglobin, using N^+^-*Mb*PylRS/N-*Mb*tRNA^Pyl^ pairs, to further tune reactivity.^133,483,484^ The genetic encoding of **NmH** as proximal ligand in a myoglobin scaffold enabled the capture of a reactive heme-carbenoid complex.^484^

Introducing **NmH** in place of a catalytic histidine residue, in a previously reported computational enzyme design,^485^ generated an enzyme that performed hydrolysis of a model ester. The parent enzyme rapidly formed an acyl enzyme intermediate between the histidine residue and the substrate, but hydrolysis of this intermediate was slow, leading to burst phase kinetics for product formation. The **NmH**-containing enzyme did not show burst phase kinetics, suggesting that the acyl-enzyme intermediate was more rapidly hydrolyzed. Directed evolution and screening of the **NmH**-containing enzyme led to a 15-fold improvement in catalytic efficiency.^486^

In future work it will be interesting to encode a wider range of ncAAs with the potential to expand the catalytic power of proteins. It will also be interesting to leverage emerging approaches for encoding combinations of ncAAs into a single proteins, to discover enzymes in which new functions emerge from combinations of ncAAs.

### 6.9 PylRS/tRNA^Pyl^ Pairs for Translational Control of Gene Expression

PylRS/tRNA^Pyl^ pairs have been central to the development of strategies for the translational control of gene expression, in which the production of proteins from genes bearing amber codons is conditional upon addition of a ncAA substrate. The facile development of PylRS/tRNA^Pyl^ pairs for eukaryotic cells and animals and the bioavailability of ncAA substrates (including an alkyne lysine derivative) for PylRS in multicellular systems have enabled these approaches.^3,96^

**Figure 17:**
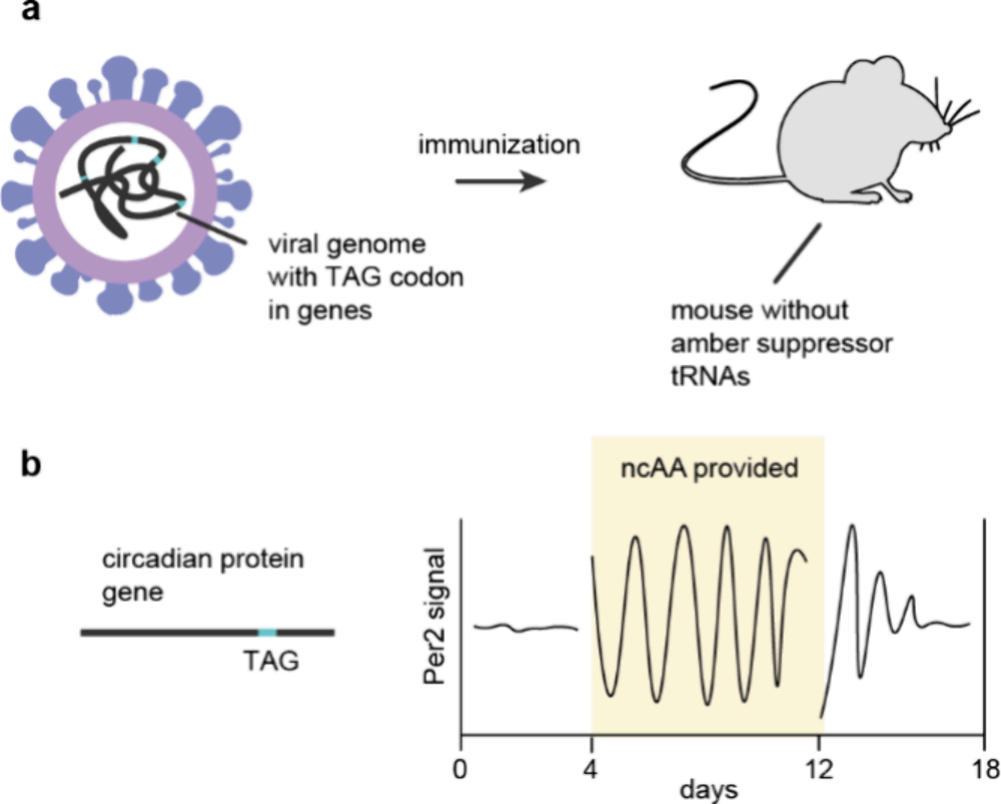
Genetic code expansion mediated translation control for the production of attenuated viruses and the ncAA induced restoration of circadian rhythms. **a**, Viral genomes were engineered to contain UAG stop codons in essential viral protein coding genes. Viruses were produced in host cells encoding PylRS/tRNA^Pyl^ pairs. The production of the viruses was dependent on the presence of the PylRS/tRNA^Pyl^ as well as its ncAA substrate. The viruses were then harvested and used to vaccinate animals, because the animals do not contain amber suppressor tRNAs the viruses cannot reproduce in the animal; this approach provides a strategy for generating attenuated viruses for immunization. Adapted with permission from Chin et al.^4^ - copyright © 2017 Springer Nature Limited. **b**, By making the production of a regulatory protein of circadian rhythms (Cry1) dependent on the ncAA induced N^+^-*Mm*PylRS/N-*Mm*tRNA^Pyl^ pair mediated translation, the circadian rhythm of otherwise arhythmic (Cry1/2 null) mice, as measured by their wheel running behavior, can be induced by providing the mice with the ncAA in their drinking water. When the ncAA is withdrawn, the circadian rhythm is switched off again. The data shows a circadian reporter (per2-luciferase) as measured by luciferase levels in suprachiasmatic nucleus slices derived from Cry1/2 null mice. When the ncAA was added to the culture medium the Cry1-dependent molecular clockwork was initiated. Adapted from Maywood et al.^487^ - copyright © The Author(s) 2018 CCBY http://creativecommons.org/licenses/by/4.0/.

Translational control has been used for conditional production of attenuated viruses for immunization (Figure 17a).^160,488−490^ In this approach amber (UAG) stop or quadruplet (UAGA) codons are introduced into the protein coding genes in a viral genome to make the production of viral proteins, and therefore the virus, dependent on the presence of a PylRS/tRNA^Pyl^ pair and cognate ncAA. In the cells used to produce the virus the PylRS/tRNA^Pyl^ pair and ncAA are provided, but in cells or animals challenged with the resulting virus the PylRS/tRNA^Pyl^ pair is absent and the ncAA is withheld; as a result, virus reproduction is attenuated in these cells or animals. This approach has been investigated as a strategy for immunization in animal models for Influenza A, HIV-1 and the RNA virus Enterovirus 71.^160,489,490^ For mice bearing the PylRS/tRNA^Pyl^ pair, and infected with Enterovirus 71 bearing amber codons in their genome, it was demonstrated that ncAA addition could be used to elicit a dose dependent increase in viral RNA and a corresponding increase in antibody response.^490^ This provides an intriguing, and potentially generalizable, strategy to tune the level of viral attenuation for immunization.

Translational control in live mice has provided a powerful approach for reversibly controlling behavior. Building on the first approaches for genetic code expansion in live mice using the N^+^-*Mm*PylRS/N-*Mm*tRNA^Pyl^ pair,^96^ AAVs were used to deliver the genes for a N^+^-*Mm*PylRS/N-*Mm*tRNA^Pyl^ pair, with N^+^-*Mm*PylRS expressed from cell type specific promoters, along with a Cry1 gene bearing an amber codon, to the brains of Cry1/2 deficient mice. These mice lack circadian rhythms. Addition of a ncAA to the drinking water of the mice turned on the circadian behavior, and removal of the ncAA turned off circadian behavior (Figure 17b). This approach provided a translational switch to study circadian biology and has led to several previously inaccessible insights.^487,491^ Translational control using N^+^-*Mm*PylRS/N-*Mm*tRNA^Pyl^ pairs has also been used to restore dystrophin expression in mice bearing amber codons in the dystrophin gene.^492^

Split aminoacyl-tRNA synthetases genes, which produce protein fragments that can assemble to produce a functional synthetase, provide a potential approach to refining the temporal and spatial regulation of translational control.^493,494^ Recent work has shown that A^Δ^-*alv*PylRS can be split into two polypeptides at several positions, and fusing the genes for interacting polypeptides to the genes encoding the resulting N-, and C-terminal fragments enables reconstitution of A^Δ^-*alv*PylRS function. This approach was demonstrated with interacting coiled-coils, and small molecule dependent dimerization domains, and used as a basis for screening protein–protein interactions.^495^

## 7 Genetically Encoding ncMs with PylRS/tRNA^Pyl^ Pairs

A body of work has focused on genetically encoding ncMs beyond α-L-amino acids. Alpha-hydroxy acids are substrates for ribosomal translation in *E. coli*(496−503) and PylRS/tRNA^Pyl^ pairs have been generated to incorporate α-hydroxy acids (Section 7.1). The permissiveness of PylRS, and designed mutants, for ncMs has been explored (Section 7.2), primarily *in vitro*, where weak and nonspecific activities can be measured. Powerful selections have been developed to discover PylRS variants that selectively acylate tRNA^Pyl^ with a desired ncM and ncAA *in vivo*, regardless of whether the ncM is a ribosomal substrate,^19^ these approaches have enabled the addition of new ncMs to the genetic code of *E. coli* (Section 7.3).

### 7.1 Adding α-Hydroxy Acids to the Genetic Code of *E. coli* with PylRS Systems

PylRS/tRNA^Pyl^ pairs provide unique advantages for incorporating α-hydroxy acids with both aliphatic and aromatic side chains into proteins.

#### 7.1.1 Genetically Encoding Hydroxy Acids with Aliphatic Side Chains

N^+^-*Mm*PylRS can acylate its cognate N-*Mm*tRNA^Pyl^ with the hydroxy acid analogue of **BocK –** (*S*)-6-(((*tert*-butoxy)carbonyl)amino)-2-hydroxyhexanoic acid (**BocK–OH**), and this enables the incorporation of this ncM into proteins in response to the amber codon, as confirmed by MS and selective, base-mediated cleavage, of the resulting ester bond. Glutathione *S*-transferase (GST) incorporating **BocK–OH** in response to an amber codon was produced at approximately 10% of the wt protein yield.^499^ The incorporation of **BocK–OH** into the leader sequence of a lanthipeptide enabled removal of the leader sequence by alkaline hydrolysis of the resulting backbone ester.^498^ To investigate the oligomerization state of lysosomal-associated membrane protein type 2A (LAMP2A) in mammalian cells, (*S*)-6-(((allyloxy)carbonyl)amino)-2-hydroxyhexanoic acid **AllocK–OH** was introduced at defined positions in one monomer of the protein bearing distinct N and C terminal tags. Upon photo-cross-linking with a second monomer of LAMP2A that site specifically incorporated **BpA**, and hydrolytic cleavage of the ester bond, the sites of cross-linking could be localized to the N- or C-terminal side of the hydroxy acid by gel electrophoresis and immunoblotting against the N- and C-terminal tags.^503^ This approach defined protein interfaces and provided insight into the in vivo geometry and multimerization state of the LAMP2A complex.

**Figure 18:**
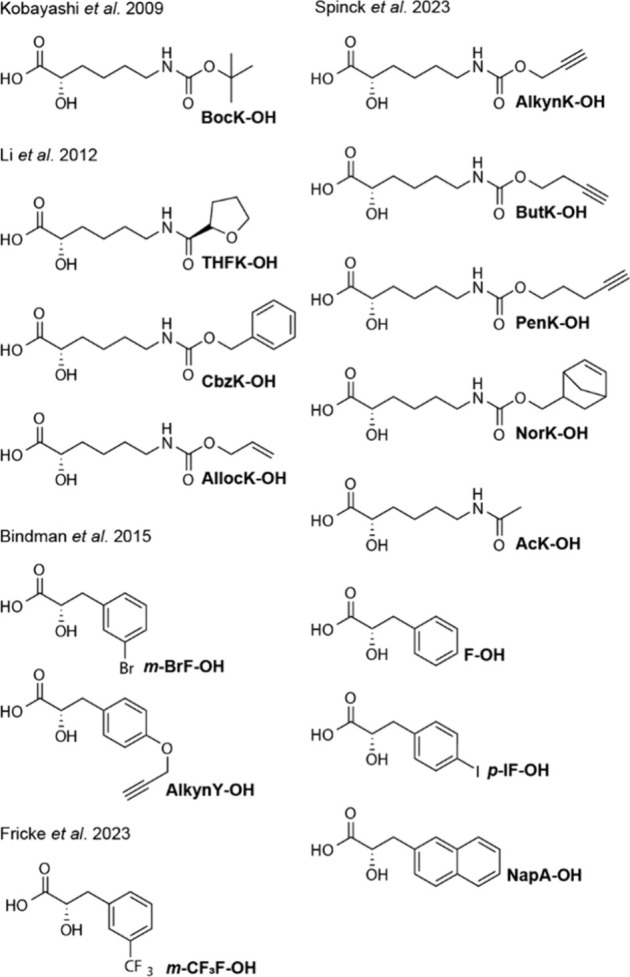
**Hydroxy acids incorporated by PylRS/tRNA**^**Pyl**^**pairs***in vivo*: Chemical structures of hydroxy acids that have been genetically encoded into proteins with PylRS**/**tRNA^Pyl^ pairs. Only examples for which the incorporation has been confirmed by MS are listed. The hydroxy acids **BocK–OH**, **AllocK–OH**, **AlkynK–OH**, **ButK–OH**, **PenK–OH**, **NorK–OH**, **CbzK–OH** and **AcK–OH** are all substrates for wt N^+^-*Mm*PylRS. The wt A^Δ^*-1r26*PylRS has a similar substrate scope, but does not recognize **NorK–OH**, and **AcK–OH**. The engineered N^+^-*Mb*PylRS(L274A, C313 V) charges **THFK–OH**, **CbzK–OH**, **AllocK–OH**, and **BocK–OH**. The promiscuous PylRS enzymes N^+^-*Mb*PylRS(L274A, C313 V), and A^Δ^*-alv*PylRS(N166A, V168A), charge the aromatic residues *m***-BrF–OH** and **Alkyn-F–OH**, or *m***-CF**_**3**_**F–OH**, respectively. PylRS enzymes were specifically evolved to charge aromatic hydroxy acids, remarkably those enzymes do not show a measurable background incorporation with aromatic canonical amino acids.^132^ The two aromatic hydroxy acid selective mutants N^+^-*Mm*PylRS(M300S, A302H, M344L, N346A) and N^+^-*Mm*PylRS(M300S, A302H, M344L, N346A, C348S, V401L, W417T) both charge **F–OH**, *p***-IF–OH** and **NapA–OH**, with the latter being more active with the bulkier substrates, and the former more active with **F–OH**.

Active site mutants of N^+^-PylRS enzymes were permissive to (*S*)-2-hydroxy-6-((S)-tetrahydrofuran-2-carboxamido)hexanoic acid **THFK–OH**, (*S*)-6-(((benzyloxy)carbonyl)amino)-2-hydroxyhexanoic acid **CbzK–OH**, **AllocK–OH**, (*S*)-3-(3-bromophenyl)-2-hydroxypropanoic acid *m***-BrF–OH** and (*S*)-2-hydroxy-3-(4-(prop-2-yn-1-yloxy)phenyl)propanoic acid **AlkyneY–OH** (Figure 18).^497,498^ Cellular incorporation of **BocK–OH** into a protein, followed by hydrazine mediated cleavage of the resulting ester, generated a protein fragment bearing a C-terminal hydrazide; this was chemically ligated to a protein fragment bearing an N-terminal cysteine.^497^

Recent work investigated the metabolism of α-hydroxy acids and their incorporation into proteins in *E. coli*, using N^+^-*Mm*PylRS/N-*Mm*tRNA^Pyl^ pairs, in more detail.^132^ When 2 mM **BocK–OH** was added to *E. coli*, a substantial fraction was converted to **BocK**. However, N^+^-*Mm*PylRS/N-*Mm*tRNA^Pyl^ selectively directed the incorporation of the hydroxy acid into proteins. Remarkably, even upon addition of equimolar amounts of a hydroxy-acid (**BocK–OH**, **AllocK–OH** or, **CbzK–OH)** and an amino acid with an identical side chain, N^+^-*Mm*PylRS selectively mediated the incorporation of the hydroxy acid into proteins in *E. coli*; there was no detectable amino acid incorporation, as assayed by MS and an ester bond-hydrolysis assay, into the proteins produced in these experiments.

#### 7.1.2 Genetically Encoding Hydroxy Acids with Aromatic Side Chains

Hydroxy acids with aromatic side chains, (*S*)-2-hydroxy-3-(4-iodophenyl)propanoic acid (*p***-IF–OH**) and (*S*)-2-hydroxy-3-(naphthalen-2-yl)propanoic acid (**NapA–OH**), were also substantially converted to the corresponding amino acids when added to *E. coli*. The deletion of transaminases that may convert a fraction of added hydroxy acids to amino acids, while apparently sufficient to support the incorporation of phenyllactic acid (**F–OH**) with an engineered *Mj*TyrRS/*Mj*tRNA^Tyr^ pair,^502^ does not provide a general strategy for ablating the conversion of hydroxy acids to amino acids.^132^ Tyrosyl-tRNA synthetase/tRNA^Tyr^ pairs, unlike PylRS, recognize the α-amine in their active sites, and they commonly lead to incorporation of amino acids when cells are provided with the corresponding hydroxy acid.^132^ As a result, hydroxy acids bearing aromatic side chains have been challenging to genetically encode using orthogonal TyrRS/tRNA^Tyr^ pairs.^132^ Recent work leveraged the preference of PylRS enzymes for hydroxy acids over amino acids to evolve N^+^-*Mm*PylRS variants that selectively acylate N-*Mm*tRNA^Pyl^ with hydroxy acids bearing aromatic side chains (*p***-IF–OH**, **NapA–OH**, and even **F–OH**, which only differs from phenylalanine by replacement of the α-amine with a hydroxy group) in *E. coli*.^132^

The evolved PylRS enzymes for hydroxy acids with aromatic side chains were selective for these hydroxy acids with respect to hydroxy acids with aliphatic side chains. Similarly, the wt PylRS, and engineered versions of PylRS, for hydroxy acids with aliphatic side chains were selective for these hydroxy acids with respect to hydroxy acids with aromatic side chains. Thus, the active sites of PylRS variants for hydroxy acids with aliphatic side chains and the active sites of PylRS variants for hydroxy acids with aromatic side chains are commonly mutually orthogonal in their hydroxy acid substrate recognition.

A nonpeer reviewed preprint built on the development of a N^+^-*Mb*PylRS variant that encodes **pcDap** and its deprotection to **Dap** as a route to trapping hydrolase substrates.^471,472^ Building on the preference of PylRS systems for hydroxy acids,^132,499^ the authors used the N^+^-*Mb*PylRS variant for **pcDap** to encode the hydroxy analogue of **pcDap, pcDap–OH**. Upon photo deprotection of incorporated **pcDap–OH**, the resulting free amine reversibly attacked the ester bond through a kinetically favored five membered transition state.^504−506^ The equilibrium for this reaction favors the formation of the thermodynamically favored peptide bond, and leads to the formation of a protein containing a β-amino acid linkage with a hydroxy substituent on the 2 position. This linkage is formed in low yield, due to low ncM incorporation efficiencies of **pcDap–OH** (1.4-fold over background misincorporation when incorporated into GFP, and 5.2 fold over background when assayed in a more sensitive NanoLuc expression system).

### 7.2 *In Vitro* and Nonspecific Activities of PylRS and Its Designed Variants with ncMs

Evidence that N^+^-*Mm*PylRS is able to aminoacylate N-*Mmt*RNA^Pyl^ with 6-((*tert*-butoxycarbonyl)amino)hexanoic acid (**BocAhx**) - a **BocK** derivative lacking the α-amine, *N*^ε^-(tert-butoxycarbonyl)-*N*^α^-methyl-*L*-lysine (**MeBocK**),^499^**BocK–OH** and *N*^ε^-(*tert*-butoxycarbonyl)-*D*-lysine (*D***-BocK**) was provided by *in vitro* aminoacylation assays with each compound. These assays used the electrophoretic mobility shift of N-*Mmt*RNA^Pyl^ upon aminoacylation to follow the acylation reaction, and did not explicitly identify the species that is acylated on to N-*Mmt*RNA^Pyl^. Therefore, it remained possible that in some cases the acylation resulted from a contaminant in the reaction; later work did not detect activity for other PylRS enzymes with D-amino acids.^507^ Nonetheless, these experiments provided the first compelling evidence that the weak recognition of the α-amine by PylRS could be exploited to acylate tRNA^Pyl^ with alternative substrates.

The A^Δ^-*alv*PylRS/*alvt*RNA^Pyl^ pair has recently been developed for genetic code expansion (Section 8).^38^*In vitro* experiments with this pair, and three of its active site variants (N166A V168L (A^Δ^-*alv*PylRS(1)), N166A V168 K (A^Δ^-*alv*PylRS(2)), and N166A V166 V (A^Δ^-*alv*PylRS(3))), demonstrated that A^Δ^-*alv*PylRS derived enzymes can acylate A^Δ^-*alvt*RNA^Pyl^ with α-hydroxy-acids (wt and all three mutants), *N*-formyl-*L*-α-amino acids (A^Δ^-*alv*PylRS(1) and (2)), as well as α-carboxy acid monomers (all three mutants). A^Δ^- *alv*PylRS(1) and (2) also showed a low, but measurable, acylation activity with α-thio acids and *N*-methyl-*L*-α-amino acids. A MS-based assay corroborated the identity of each acylated monomer. A crystal structure of A^Δ^-*alv*PylRS(3) with the α-carboxy monomer 2-(3-(trifluoromethyl)benzyl)malonic acid (*m***-CF**_**3**_**BME**), and AMP-PNP provided insight into monomer recognition.^507^ The A^Δ^-*alv*PylRS(3) enzyme appeared to display modest selectivity for α-carboxy acid derivatives, over natural amino acids and most of the monomers tested were not loaded onto A^Δ^-*alvt*RNA^Pyl^ at levels substantially above background mis-acylation with natural amino acids, notably phenylalanine. Each class of monomer (with the exception of *N*-methyl-*L*-α-amino acids) was genetically encoded *in vitro* at the first position of a peptide in an *in vitro* translation reaction based on start codon skipping and devoid of phenylalanine. This approach ensured that the ribosome did not need to use the variant functional groups in a bond forming reaction, and allowed weak the activities of the synthetases to be utilized in the absence of competition with phenylalanine. The mutant synthetases do not appear to be specific or active enough with these monomers (except the hydroxy acid) to support their site-specific *in vivo* incorporation.

In another nonpeer reviewed preprint (published in its final form after this review was submitted),^508^ it was reported that wt A^Δ^-*alv*PylRS could aminoacylate its cognate tRNA with (*S*)-β^2^, and (*R*)-β^2^**BocK–OH** derivatives *in vitro*. The A^Δ^-*alv*PylRS(3) mutant was reported to accept the (*R*)-β^2^ hydroxy acid - (*R*)-3-hydroxy-2-(3-(trifluoromethyl)benzyl)propanoic acid (**(***R***)-β**^**2**^**-***m***-CF**_**3**_**F–OH**) - with low efficiency, and the addition of the (*S*)-β^2^ enantiomer did not yield any acylated A^Δ^-*alv*tRNA^Pyl^. The wt A^Δ^-*alv*PylRS/A-*alv*tRNA^Pyl^ pair facilitated the incorporation of some (*S*)-6-((*tert*-butoxycarbonyl)amino)-2-(hydroxymethyl)hexanoic acid (**(***S***)-β**^**2**^**-Bock–OH**) monomer at one to two positions in GFP (position 3, and an insertion between positions 213 and 214 into a loop of GFP) in *E. coli*. However, a substantial amount of glutamine–minimally 30% – was incorporated at the same position as the hydroxy acid monomers, and the overall yield was low. The nonspecific activities reported in these experiments further highlighted the challenges in moving from *in vitro* acylation–where low and nonspecific synthetase activities can be detected–to *in vivo* experiments where more active and specific variants are required to outcompete misincorporation processes.

### 7.3 Evolving PylRS Enzymes for Selective *In Vivo* Acylation

The most powerful approaches for discovering aaRS/tRNA pairs for new ncAAs have used directed evolution to select synthetase variants from a library of active site mutants (Section 4). These approaches have relied on translational read outs and therefore required the monomers to be ribosomal substrates, frequently at specific positions in the reporter protein. This limitation is not a problem for most α-*L*-amino acids with variant side chains, which are generally well-tolerated by the ribosome and the rest of the translation machinery. However, many ncMs may not be good *in vivo* substrates for the translational machinery of cells^509^ and therefore double-sieve selections that use translational readouts are likely of limited utility for discovering orthogonal aaRS/tRNA pairs for ncMs. An evolutionary deadlock has been identified for translation-based selections, including double-sieve selections: ribosomes, and other translational components, cannot be evolved to polymerize ncMs that cannot be acylated onto tRNAs, and aaRS enzymes cannot be evolved to acylate tRNAs with ncMs that are not substrates for translation. Recent work has broken this deadlock by developing direct selections for tRNA acylation (Section 7.3.1),^19^ these selections enabled the discovery of PylRS variants for several classes of ncMs (Section 7.3.2), and the addition of several ncMs to the genetic code of *E. coli* (Section 7.3.3).

#### 7.3.1 The Development of a Scalable tRNA Display Platform

tRNA display enables the direct selection of aaRS enzymes that selectively and efficiently acylate their cognate tRNA with a desired ncM, regardless of whether the ncM, when loaded on to the tRNA, is a substrate for ribosomal polymerization.^19^ To develop tRNA display, methods were created to (1) selectively isolate acylated tRNAs with respect to tRNAs that were not acylated, (2), link the *in vivo* acylation of a tRNA to the sequence of the aaRS mutant responsible for the acylation, and (3) identify aaRS sequences that selectively acylate their tRNA with the ncM of interest rather than a canonical amino acid. These methods link the phenotype (acylation with a ncAA or ncM) to genotype (sequence of the aaRS that performs the acylation).

**Figure 19:**
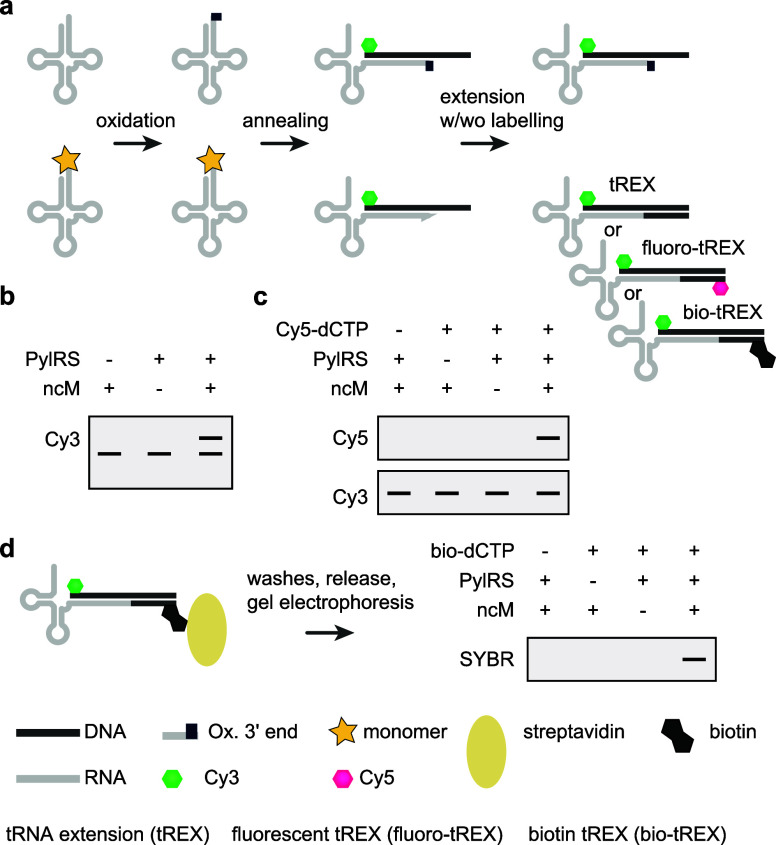
**Transfer RNA extension protocols for the analytical, and physical separation of acylated tRNAs from free tRNAs. a**, Isolated tRNAs are oxidized with sodium periodate, during which the diol functionality of the 3′-ribose of acylated tRNAs is protected from oxidation to the aldehyde. A DNA probe bearing a 3′Cy3 label is annealed to the tRNA and the tRNA is extended by Klenow (exo-) DNA polymerase fragment. For fluoro- and bio-tREX a DNA probe that has a 5′poly G stretch, and is otherwise devoid of G, is used together with an extension mix that contains dNTPs without dCTPs and is supplemented with either biotinylated- or Cy5 labeled dCTPs. **b**, In tREX, the difference in mass between the extended (previously acylated) and nonextended (previously nonacylated) tRNAs is visualized by gel electrophoresis to identify acylated tRNAs. **c**, In fluoro-tREX the previously acylated tRNAs are labeled with Cy5 and can be visualized by gel electrophoresis. **d**, In bio-tREX, the previously acylated tRNAs are biotinylated, bound to streptavidin beads and can then be eluted and analyzed by gel electrophoresis. Adapted from Dunkelmann et al.^19^ – copyright © The Author(s) 2024 CCBY http://creativecommons.org/licenses/by/4.0/.

To selectively isolate the acylated form of N-*Mm*tRNA^Pyl^ from cells the authors developed–based on the previously developed tRNA extension (tREX) protocol^83^ (Figure 19a,b) – fluorescent tRNA extension (fluoro-tREX) (Figure 19a,c), and biotin tRNA extension (bio-tREX) (Figure 19a,d). In bio-tREX, tRNAs are isolated from cells, and oxidized with sodium periodate. During oxidation, the acyl-group of the 3′ ribose of acylated tRNAs acts as chemical protecting group, preventing the conversion of the diol functionality into a dialdehyde (preserving the ribose for enzymatic extension). Subsequently, the tRNAs are deacylated, and a DNA probe containing a 5′ overhang with a terminal poly G stretch and sequence complementary to the 3′ end of N-*Mm*tRNA^Pyl^ is hybridized to the 3′ end of N-*Mm*tRNA^Pyl^. The overhang is then extended with the DNA polymerase fragment Klenow (exo-) using a nucleotide mixture containing biotinylated deoxycytidine triphosphates (bio-dCTPs), instead of dCTPs. Only the formerly acylated tRNAs are biotinylated, and can be isolated on streptavidin beads. Bio-tREX provided an efficient method to selectively isolate acylated tRNAs. In fluoro-tREX, Cy5-dCTPs are used instead of bio-dCTPs and the extension product is directly visualized following gel electrophoresis.

**Figure 20:**
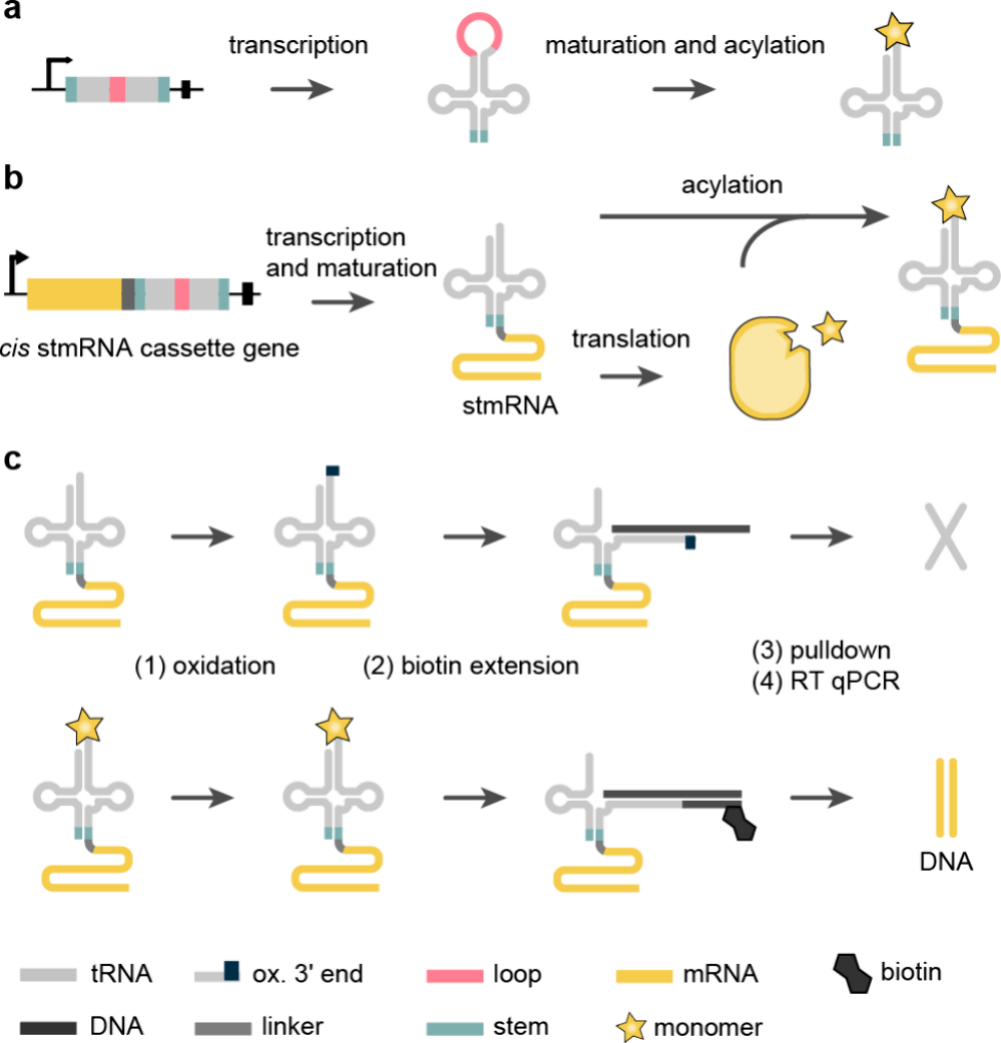
**Methodological basis of the tRNA display platform. a**, Assembly, maturation and acylation of split tRNAs *in vivo*. Pyrrolysyl tRNA can be split, at the anticodon, into two halves and expressed as circularly permutated tRNA from one construct in cells. The split tRNA is recognized and acylated by PylRS *in vivo*. **b**, Split tRNA-mRNA fusions (stmRNAs) can be produced from one transcript by circular permutation of the split tRNA and attaching the PylRS mRNA to the 5′ end of the 3′ half of the tRNA. Split tRNA-mRNA fusions serve as the mRNA for the production of PylRS enzymes, as well as tRNA substrates of PylRS, thereby connecting genotype (PylRS mRNA) to phenotype (acylated tRNA^Pyl^). **c**, Biotin mRNA extension (bio-mREX) leads to the selective isolation of the DNA sequences of active PylRS enzymes. Split tRNA-mRNA fusions are oxidized with sodium periodate. During the oxidation the 3′ end of acylated stmRNAs is protected, while the 3′ end of free stmRNAs is inactivated. A DNA probe is annealed to the stmRNA, extended and the stmRNAs with intact 3′ ends are biotinylated. Therefore, only formerly acylated stmRNAs get biotinylated. The biotinylated stmRNAs are isolated on streptavidin beads, reverse transcribed, and either submitted to quantitative PCR (qPCR) or cloned into a new backbone for multiple rounds of selection. Adapted from Dunkelmann et al.^19^ – copyright © The Author(s) 2024 CCBY http://creativecommons.org/licenses/by/4.0/.

To link the *in vivo* acylation of N-*Mm*tRNA^Pyl^ to the sequence of the N^+^-*Mm*PylRS mutant responsible for the acylation the authors developed several new approaches. First, they showed that the RNA sequence of N-*Mm*tRNA^Pyl^ can be split at the anticodon to create a 5′ half and a 3′ half, and that these two halves of N-*Mm*tRNA^Pyl^ can be produced, through *in vivo* RNA processing, from a single transcript (Figure 20a). The two halves assemble *in vivo* to form a split tRNA, which is acylated by N^+^-*Mm*PylRS as efficiently as the parent tRNA in *E. coli*. Next, they covalently linked the mRNA of N^+^-*Mm*PylRS to the 5′ end of the 3′ half of the split tRNA to create a split tRNA-mRNA fusion (stmRNA). The stmRNA encodes the PylRS enzyme (genotype), while also containing the substrate (tRNA^Pyl^). Within each cell the activity of the PylRS enzyme determines the extent to which its encoding stmRNA is acylated (Figure 20b).

Several additional advances enabled researchers to identify aaRS sequences that selectively acylate their tRNA with a monomer of interest. First, the bio-tREX protocol was adapted for the selective capture and reverse transcription of stmRNAs that were acylated in the cell (creating biotin mRNA extension (bio-mREX)) (Figure 20c). Bio-mREX recovered 300-fold more stmRNA molecules encoding wt N^+^-*Mm*PylRS than encoding an attenuated activity mutant of N^+^-*Mm*PylRS, when cells were provided with **BocK**. Second, the expression level of N^+^-*Mm*PylRS from the stmRNA was tuned such that the recovery of stmRNAs molecules, encoding N^+^-*Mm*PylRS variants for a ncAA, by bio-mREX was well correlated with the amber suppression efficiency of the corresponding N^+^-*Mm*PylRS/N-*Mm*tRNA^Pyl^ pair with the respective ncAA.

**Figure 21:**
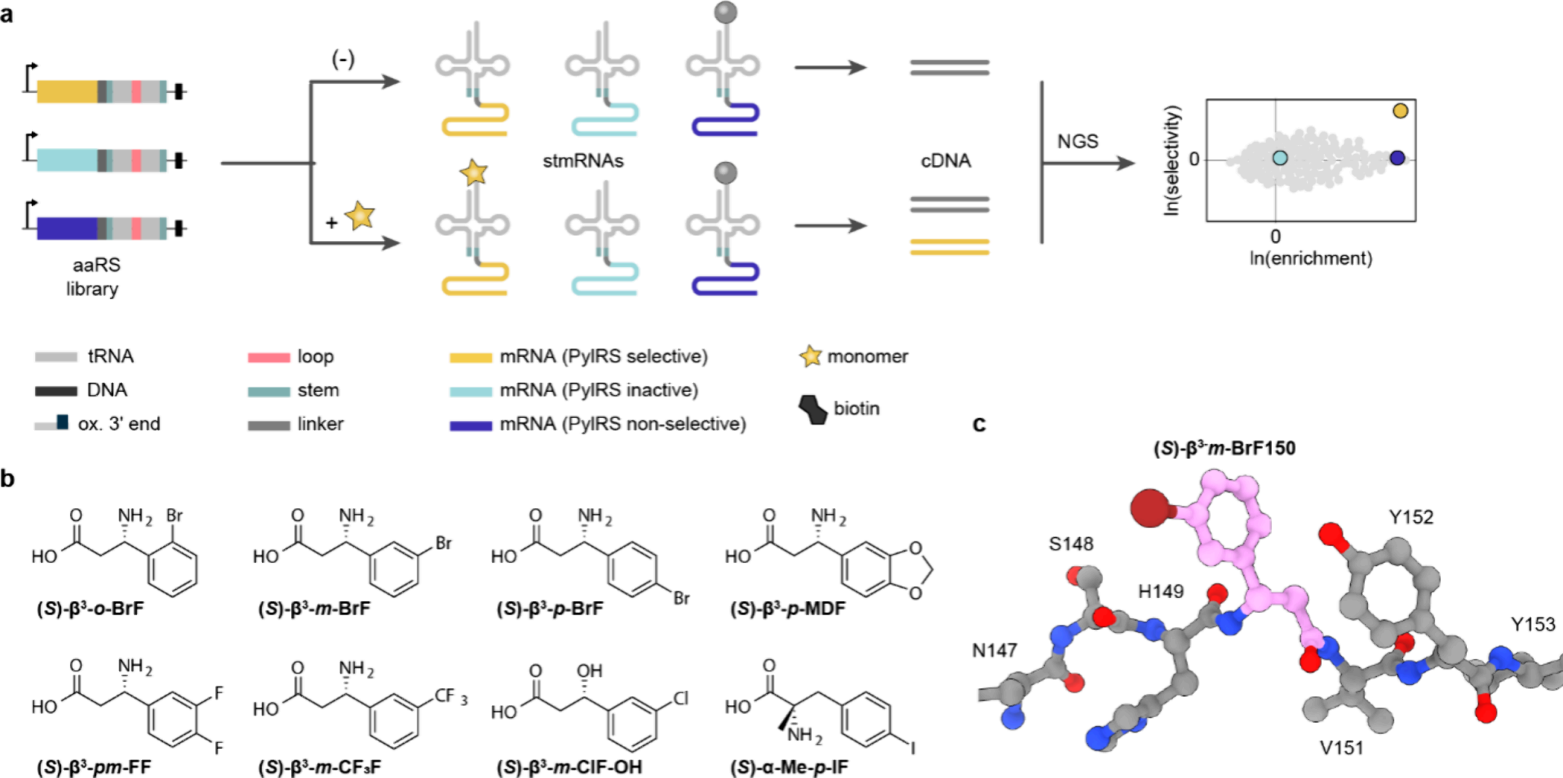
**Evolution of β-aminoacyl-, β -hydroxyacyl-, and α-, α-disubstituted aminoacyl-tRNA synthetases by tRNA display. a**, Schematic representation of the tRNA display protocol. Split tRNA-mRNA fusions encoded PylRS active site libraries are submitted to two parallel bio-mREX experiments, in presence and absence of the target ncM. Isolated cDNA is submitted to NGS. Two parameters are determined: (1) Enrichment–a proxy for acylation activity–which is calculated as the ratio of the relative abundance of a sequence after the selection, divided by the relative abundance of the same sequence in the input library. (2) Selectivity–a proxy for acylation specificity–which is calculated as the relative abundance of a sequence after selection in the presence of the ncM, divided by the relative abundance of the same sequence after selection in absence of the ncM. Enrichment and selectivity are plotted in spindle plots, and highly enriched and selective sequences further characterized. **b**, Schematic representation of substrates for evolved N^+^-*Mm*PylRS variants. Substrates **(***S***)-β**^**3**^**-***m***-BrF**, **(***S***)-β**^**3**^**-***m***-CF**_**3**_**F**, **(***S***)-β**^**3**^**-***p***-BrF**, and **(***S***)-α-Me-***p***-IF–OH** were site-specifically genetically encoded in a protein. **c**, Crystal structure (PDB 8OVY) of β-amino acid **(S)- β**^**3**^**-***m***-BrF** at position 150 in green fluorescent protein incorporated with an evolved N^+^-*Mm*PylRS/N-*Mm*tRNA^Pyl^ pair. Adapted from Dunkelmann et al.^19^ – copyright © The Author(s) 2024 CCBY http://creativecommons.org/licenses/by/4.0/.

tRNA display used stmRNA libraries (which vary the sequence of N^+^-*Mm*PylRS). These libraries were subject to parallel bio-mREX-based selections in the presence and absence of ncMs and, the N^+^-*Mm*PylRS sequence within the captured stmRNAs was determined by NGS (Figure 21a). A measure of enrichment, a proxy for acylation activity, was provided by comparing the abundance of a sequence after selection in the presence of a ncM to its abundance in the input. A measure of selectivity, a proxy for acylation specificity, was provided by comparing the abundance of a sequence after selection in the presence of the ncM to the abundance of a sequence following selection in absence of the ncM. Desired sequences were enriched and selective.

Experiments with N^+^-*Mm*PylRS libraries and ncAAs that are known ribosomal substrates demonstrated the power of tRNA display. Active and selective sequences were obtained directly from the sequencing data and there was a strong correlation between the enrichment of PylRS sequences in tRNA display and the activity of the resulting N^+^-*Mm*PylRS/N-*Mm*tRNA^Pyl^ pair in amber suppression-dependent protein expression. N^+^-*Mm*PylRS variants for eight ncAAs were discovered from several large libraries by tRNA display. Highly active and selective pairs, with convergent N^+^-*Mm*PylRS sequences, were discovered directly by sequence-based criteria. These experiments also used 50–100 times less compound than previous double sieve-based selection methods.

A nonpeer reviewed preprint reported a translation independent method for PylRS enzyme engineering (termed “selection for tRNA-acylation without ribosomal translation” (START); this paper was published after submission of this review).^510^ Transfer RNA display and START both rely on the periodate oxidation step to chemically differentiate between charged and free tRNAs, and probe-mediated extension of the previously acylated tRNA, as previously described in tREX.^83,511^ However, START relies on cutting out the gel band generated by the original tREX protocol to isolate previously acylated tRNAs,^83^ while tRNA display uses selective biotinylation of the formerly acylated tRNA and subsequent pulldowns. START aims to indirectly correlate genotype and phenotype by adding a barcode to the tRNA anticodon stem loop and sequencing plasmids which encode the barcoded tRNAs gene together with the gene for the active site variants of PylRS. In contrast, tRNA display directly links the tRNA substrate to the mRNA of the synthetase and so directly connects the genotype to the phenotype.

tRNA display leads to 30-fold higher enrichments of active over attenuated PylRS variants (300-fold vs 11-fold), and tRNA display enables effective selections from libraries that are 3 orders of magnitude larger than those interrogated by START (10^8^ member vs 10^5^ members). Furthermore, with tRNA display the active mutants are directly isolated and can be submitted for further rounds of evolution, while START can only be run in one step. While START has been used to evolve active site variants for ncAAs which have previously been incorporated, only tRNA display has yielded PylRS enzymes, which are active and selective with new monomers (Section 7.3.2).^19^

#### 7.3.2 PylRS Variants for ncMs from tRNA Display

One or two rounds of tRNA display selection, with highly diverse active site libraries of N^+^-*Mm*PylRS, led to the discovery of active and selective PylRS mutants for eight ncMs. These ncMs included six β^3^-amino acids, a β^3^-hydroxy acid, and an α-, α-disubstituted amino acid (Figure 21b). This work provided the first active and selective aaRS enzymes for these three classes of monomers.

Each evolved PylRS variant exhibited ncM dependent *in vivo* acylation of N-*Mm*tRNA^Pyl^, as directly characterized by fluoro-tREX. Importantly, the identity of the ncM loaded onto N-*Mm*tRNA^Pyl^ was directly confirmed by a MS-based assay. These assays provide important approaches to characterizing the specificity of aaRS systems that load ncMs onto their tRNAs, regardless of whether the acylated tRNAs function in translation.

An important aspect of tRNA display is the wealth of sequence information derived from the analysis of each selection. The observation that N^+^-*Mm*PylRS enzymes accepting β^3^-amino acids predominantly share a M300D mutation and a A302H mutation, provides insight into sequence motifs that may support recognition of this class of substrates. Interestingly, the aspartic acid in position 300, which may be in proximity to the β^3^ amine changes to an uncharged asparagine in the case of the mutants observed for β^3^-hydroxy acid, while the A302H mutation is conserved between both.

The generation of efficient and selective synthetases for some ncMs, was sufficient to enable their incorporation into proteins (Section 7.3.3). However, other ncM that were efficiently and specifically acylated were not incorporated into proteins; the ability to efficiently and selectively acylate tRNAs with these ncMs provides a starting point for using translational selections to evolve translational components (eg: tRNAs, EF-Tu, EF-P, ribosomes) to facilitate their genetically encoded incorporation.

We anticipate that direct selections for acylation will enable the generation of efficient and selective synthetases for diverse new classes of ncMs. It may also allow selection for other enzymatic activities that can be linked to tRNA acylation.

#### 7.3.3 Adding ncMs to the Genetic Code of *E. coli* Using PylRS/tRNA Pairs from tRNA Display

N^+^-*Mm*PylRS variants discovered by tRNA display, when combined with N-*Mm*tRNA^Pyl^ in cells containing a GFP reporter with an amber codon at position 150 and added ncM led to clean, site-specific genetic encoding of three β^3^-amino acids: (*S*)-3-amino-3-(3-bromophenyl)propanoic acid (**(***S***)-β**^**3**^**-***m-***BrF**), (S)-3-amino-3-(4-bromophenyl)propanoic acid (**(***S***)-β**^**3**^**-***p***-BrF**), (S)-3-amino-3-(3-(trifluoromethyl)phenyl)propanoic acid (**(***S***)-β**^**3**^**-***m-***CF**_**3**_**F**), and one α-, α-disubstituted amino acid (S)-2-amino-3-(4-iodophenyl)-2-methylpropanoic acid (**(***S***)-α-Me-***p***-IF**) into GFP at position 150, as judged by whole protein mass spectrometry and tryptic MS/MS.

This work constituted the first expansion of the genetic code for the incorporation of these classes of monomers into a protein in a living organism. Protein yields were 3–35 mg L^–1^ of culture and the high efficiency of ncM incorporation permitted the production of the first crystal structure of a β^3^-amino acid containing protein through expansion of the genetic code (Figure 21c).

We anticipate that the ability to genetically encode these new classes of ncMs will enable translational selections to enhance the efficiency with which they are incorporated at diverse sites in proteins. By combining these advances with advances in encoding noncanonical polymers (Section 11) it may be possible to genetically encode the cellular synthesis of noncanonical polymers composed of ncMs.

## 8 Discovery and Engineering of Orthogonal and Multiply Orthogonal PylRS/tRNA^Pyl^ Pairs

Recent work has demonstrated that the sequence diversity between and within PylRS/tRNA^Pyl^ groups discovered by culture and metagenomic approaches^35,37^ (Section 3) can be exploited as a starting point for discovering new orthogonal pairs (Section 8.1), sets of mutually orthogonal pairs (Section 8.2), triply orthogonal pairs (Section 8.3), and even quintuply orthogonal pairs (Section 8.4). We define two pairs X and Y as mutually orthogonal if the aaRS of X acylates the tRNA of X, but not the tRNA of Y, and the aaRS of Y acylates the tRNA of Y, but not the tRNA of X. Thus, we define mutual orthogonality in terms of acylation specificity. Triply and quintuply orthogonal pairs extend the idea of mutually orthogonal pairs to three or five pairs. In order to be used to uniquely direct the incorporation of distinct monomers, mutually orthogonal pairs may need further engineering to generate mutually orthogonal active sites–such that the active site of the aaRS of X recognizes its substrate and not the substrate of the aaRS of Y, and *vice versa*. In order to be used to decode distinct codons, the tRNA of X and the tRNA of Y must be altered, such that they uniquely decode distinct codons.

### 8.1 A^Δ^-PylRS/tRNA^Pyl^ Pairs Are Active and Orthogonal in *E. coli*

Pioneering efforts focused on characterizing five N^Δ^-PylRS enzymes (from *alv*, *Methanogenic archaeon ISO4-G1* (*g1*), *Methanogenic archaeon ISO4-H5* (*h5*), *Methanonatronarchaeum termitum* (*term*), and *Methanomassiliicoccus luminyensis* (*lum1*), respectively), for which tRNA^Pyl^ like sequences could be identified, in *E. coli*. All five pyl tRNAs were predicted to fold into a canonical cloverleaf structure. The most notable features of the pyl tRNAs from group ΔN, when compared to pyl tRNAs from group + N, are a shortened D-loop, the absence of the U8 nucleotide between acceptor stem and D-arm from the sequence (with the exception of *lum1*tRNA^Pyl^), as well as a unique bulge or loop in the anticodon stem (with the exception of *g1*tRNA^Pyl^); these features had not been observed in any other tRNAs sequences at the time this work was carried out.^35,38,512^

All five N^Δ^-PylRS/tRNA^Pyl^ pairs were active and orthogonal in *E. coli*. This demonstrated that the N^Δ^-PylRS genes encoded functional enzymes that did not require other components to acylate their tRNAs. Compared to the widely used N^+^-*Mm*PylRS/N-*Mm*tRNA^Pyl^ pair, A^Δ^-*alv*PylRS/A-*alv*tRNA^Pyl^ led to higher amber suppression activity, while A^Δ^-*g1*PylRS/A-*g1*tRNA^Pyl^ showed comparable activity; the other pairs were much less active. The low activity of some of the systems in *E. coli* was later rescued by installing a translationally favored A37 nucleotide into the anticodon loop of the pyl tRNAs. A^Δ^*-g1*PylRS, and A^Δ^*-alv*PylRS both belong to the class A (as defined later, Section 8.3) of group ΔN.

The high activity of the A^Δ^-*alv*PylRS/A-*alv*tRNA^Pyl^ pair correlates with a very low number of amber stop codons in the parent organism (1.6% of stop codons for *alv*, and 11% of stop codons for *lum1*, respectively) as well as a high number of in frame amber codons in proteins (19 for *alv* and three for *lum1*, respectively).^36^ However, *in vitro* aminoacylation kinetics for N^+^-*Mm*PylRS/N-*Mm*tRNA^Pyl^, N^+^-*Mb*PylRS/N-*Mb*tRNA^Pyl^, and A^Δ^-a*lv*PylRS/A-*alv*tRNA^Pyl^ pairs with **Pyl** suggested that the *k*_cat_/K_m_ for the A^Δ^-a*lv*PylRS/A-*alv*tRNA^Pyl^ pair is 3-fold lower than for the N^+^-*Mm*PylRS/N-*Mm*tRNA^Pyl^, and 11-fold lower than for the N^+^-*Mb*PylRS/N-*Mb*tRNA^Pyl^ pair.^42^ It is not uncommon for *in vitro* acylation kinetics to correlate poorly with *in vivo* incorporation measurements; there are several possible reasons for this: (1) *in vitro* measurements commonly use *in vitro* transcribed tRNAs–which may not reflect the modification, processing or folding state of tRNAs in cells–and (2) *in vitro* acylation measurements report on only one step in *in vivo* ncAA incorporation, which includes ncAA uptake and availability, EF-Tu binding, and ribosomal decoding.

As it was commonly assumed that PylRS systems required a N-terminal domain (in *cis* or *trans*) to function efficiently *in vivo*, the discovery and characterization of numerous PylRS systems that do not require an N-terminal domain and are very active and orthogonal was a surprising and important advance.

### 8.2 Mutually Orthogonal PylRS/tRNA^Pyl^ Pairs

A body of work has investigated the discovery and generation of mutually orthogonal PylRS/tRNA^Pyl^ pairs. Natural mutually orthogonal pairs have been discovered from distinct organisms and within a single organism (Section 8.2.1), and optimized mutually orthogonal pairs have been evolved and engineered in *E. coli* (Section 8.2.2), and subsequently been developed in mammalian cells (Section 8.2.3).

#### 8.2.1 Natural Mutually Orthogonal PylRS/tRNA^Pyl^ Pairs

A screen of the amber suppressor activities of intergroup and intragroup combinations of PylRS enzymes and pyl tRNAs provided pivotal insights into the preferences of PylRS enzymes from groups + N and ΔN for different pyl tRNAs.^38^ Both N^Δ^-PylRS enzymes, A^Δ^-*alv*PylRS as well as A^Δ^-*g1*PylRS, led to almost no amber suppression when paired with group + N tRNA N-*Mm*tRNA^Pyl^. This observation constituted the first example of complete *in vivo* orthogonality of PylRS enzymes toward pyl tRNAs, which are active with at least one alternative PylRS enzyme. Remarkably, the screen resulted in the discovery of a naturally mutual orthogonal PylRS/tRNA^Pyl^ pair, composed of the group ΔN pair (A^Δ^-*g1*PylRS/A-*g1*tRNA^Pyl^) and a group + N pair (N^+^-*Mm*PylRS/N-*Mm*tRNA^Pyl^). However, the lower activity of A^Δ^-*g1*PylRS in comparison to A^Δ^-*alv*PylRS combined with a small, but measurable, cross-reactivity of N^+^-*Mm*PylRS with A-*g1*tRNA^Pyl^ incentivized the development of more active mutually orthogonal pairs based on the highly active A^Δ^-*alv*PylRS/A-*alv*tRNA^Pyl^ pair (Section 8.2.2).

**Figure 22:**
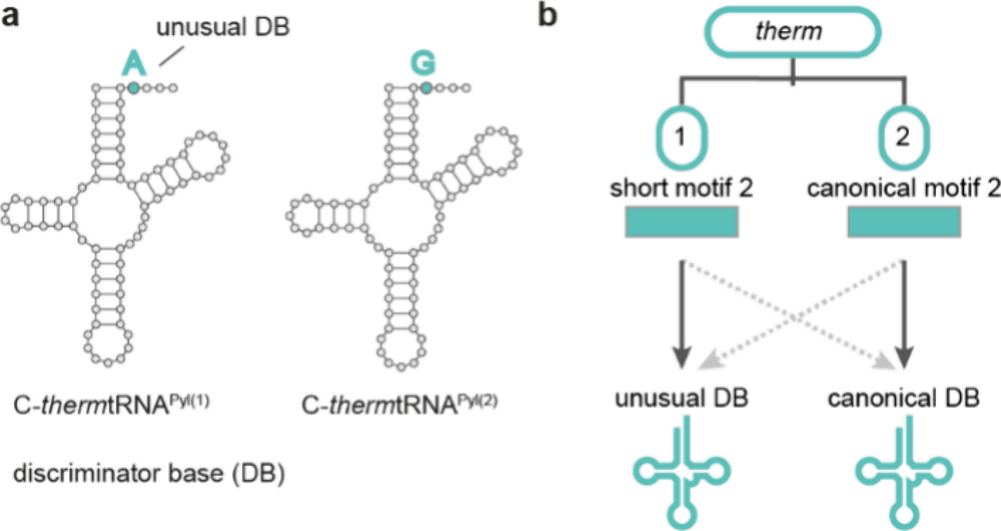
**Naturally mutually orthogonal PylRS/tRNA**^**Pyl**^**pairs in archaea. a**, The archaeon *Candidatus Methanohalarchaeum thermophilum* (*therm*) harbors two distinct PylRS/tRNA^Pyl^ pairs in its genome. The two pyl tRNAs carry distinct discriminator bases (DBs). **b**, The intraorganism mutual orthogonality between *therm*(1)PylRS/*therm*tRNA^Pyl(1)^ pair and *therm*(2)PylRS/*therm*tRNA^Pyl(2)^ is derived from the combination of an unusual DB in the *therm*tRNA^Pyl(1)^ which is only recognized *therm*(1)PylRS that has a shortened motif 2, and is orthogonal to *therm*tRNA^Pyl(2)^. Experiments defining mutual orthogonality were carried out in the model archaeon *Haloferax volcanii*.

Recent work has discovered genes for two sets of ΔN group PylRS/tRNA^Pyl^ pairs encoded in the genome of a single organism, the extremely halophilic euryarchaeal methanogen *Candidatus Methanohalarchaeum thermophilum* (*therm*).^513^ The two C^Δ^-*therm*PylRS pairs (1 and 2–the numbering is in accordance with the characterization of *therm*tRNA^Pyl(1)^ in *E. coli* in parallel work^40^) are formed from distinct C^Δ^-PylRS enzymes and C-pyl tRNAs. An amino acid deletion in sequence motif 2 of C^Δ^-*therm*PylRS1 distinguishes it from C^Δ^-*therm*PylRS2 (the enzymes have a sequence identity of 53%), and the most notable difference between the C-pyl *therm*tRNAs appears to be the change of the discriminator base from the canonical G in C-*therm*tRNA^Pyl(2)^ to A in C-*therm*tRNA^Pyl(1)^ (Figure 22a). Characterization of the C^Δ^-*therm*PylRS/C-*therm*tRNA^Pyl^ pairs in the model halophilic archaeon *Haloferax volcanii* (no activity was measured in amber suppression experiments in *E. coli*) revealed that the two pairs are mutually orthogonal, and that the mutual orthogonality is a direct consequence of both the discriminator base differences and amino acid deletion in motif 2 (Figure 22b). This work demonstrated the occurrence of naturally, mutually orthogonal aaRS/tRNA pairs of the same iso-acceptor class, derived from the same host species.

Pyrrolysine systems are the only isoacceptor systems for which orthogonal and mutually orthogonal pairs have been discovered within an isoacceptor class (and both inter and intra organism examples have been reported). This may be a function of: (1) the sequence and structural differences of pyl pairs from aaRS/tRNA pairs for canonical amino acids that favors orthogonality of pyl pairs to canonical pairs, and (2) the large sequence diversity of pyl pairs that has arisen through natural evolution. We note that these two points may be related, as the sequence and structural differences of pyl systems to canonical pairs may make their evolutionary trajectories less constrained–mutations in pyl systems that lead to toxic cross reactivity with canonical systems may be rarer than mutations in canonical systems that lead to toxic cross reactivity with each other.

#### 8.2.2 Engineered Mutually Orthogonal PylRS/tRNA^Pyl^ Pairs

**Figure 23:**
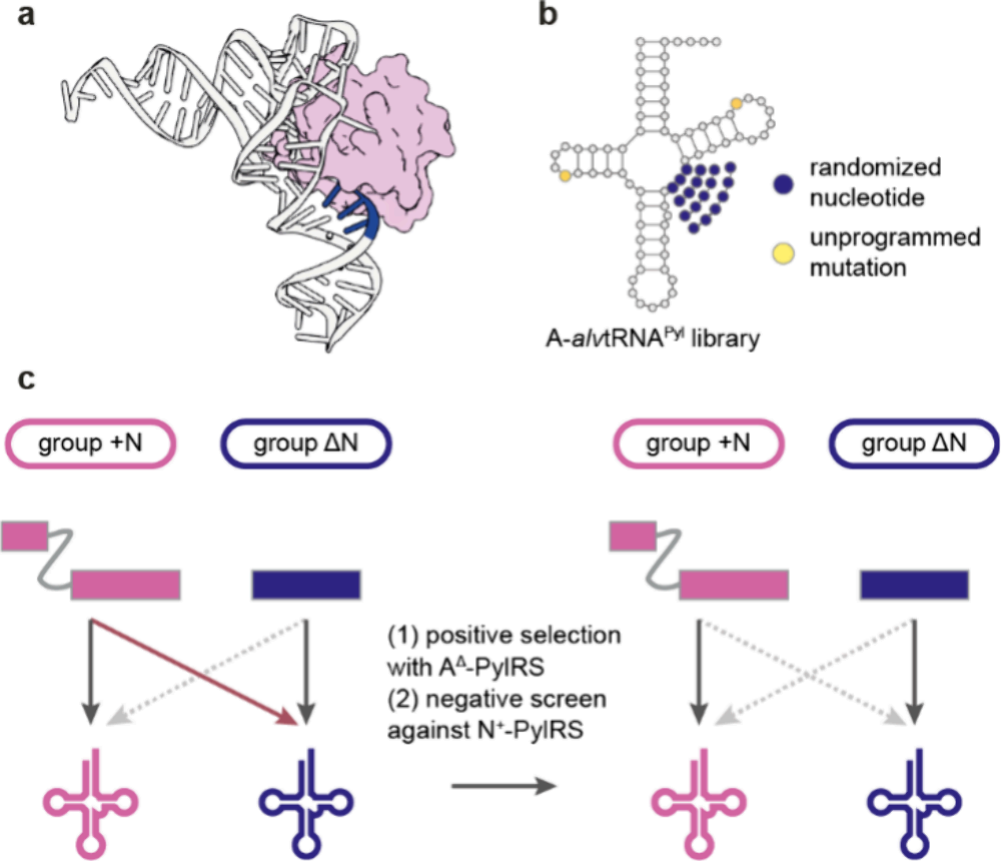
**Engineering mutually orthogonal PylRS/tRNA**^**Pyl**^**pairs in***E. coli***. a**, Structure of N-*Mm*PylRSn (pink) bound to *Mm*tRNA^Pyl^ (gray) (5UD5).^60^ The interaction interface of N-*Mm*PylRSn with the variable loop (blue) is dependent on the presence of the N-terminal domain. The absence N-PylRSn and therefore variable loop recognition in group ΔN PylRS enzymes provides a direct means to engineering PylRS specificity by variable loop extension, impeding the interaction interface with N^+^-*Mm*PylRS. **b**, Depiction of A-*alv*tRNA^Pyl^ libraries with extended anticodon loops. Nucleotides marked in blue were randomized, and changes to the nucleotides marked in yellow emerged as unprogrammed mutations during the selection. **c**, Schematic representation of the strategy employed to generate mutually orthogonal PylRS/tRNA^Pyl^ pairs formed of N^+^-*Mm*PylRS/N-*Mm*tRNA^Pyl^ and A^Δ^-*alv*PylRS/A-*alv*tRNA^Pyl^ by (1) identifying A-*alv*tRNA^Pyl^ variants with an expanded variable loop that are active with A^Δ^-*alv*PylRS, and (2) screening against cross-reactivity with N^+^-*Mm*PylRS.

Since the combination of N^+^-*Mm*PylRS and A-*alv*tRNA^Pyl^ led to high levels of amber suppression, the binding interface between N^+^-*Mm*PylRS and A-*alv*tRNA^Pyl^ was targeted for disruption in an effort to generate a highly active mutually orthogonal PylRS/tRNA^Pyl^ pair. The distinct architectures of N^+^-PylRS and N^Δ^-PylRS enzymes implied distinct tRNA^Pyl^ binding modes, with the absence of the N-terminal domain presumably weakening T-stem and D-stem, and T-loop and variable loop recognition by N^Δ^-PylRS enzymes (Figure 23a).^63^ The authors hypothesized that extension of the variable loop of A-*alv*tRNA^Pyl^ might be tolerated by N^Δ^-PylRS enzymes but not N^+^-PylRS enzymes (Figure 23b).^38^

To engineer orthogonal A-*alv*tRNA^Pyl^ variants, libraries with randomized variable loops of three to six nucleotides were subjected to a round of positive selection for activity with A^Δ^-*alv*PylRS and a negative screen for absence of activity with N^+^-*Mm*PylRS. As hypothesized, A^Δ^-*alv*PylRS enzymes tolerated variable loops of up to six nucleotides in A-*alv*tRNA^Pyl^ while retaining high activity. On the other hand, variable loop extensions in A-*alv*tRNA^Pyl^ completely abolished the activity with the N^+^-*Mm*PylRS enzyme (Figure 23c). Notably, unprogrammed compensatory mutations at position G60A, T, or C, or C21T of A-*alv*tRNA^Pyl^ emerged spontaneously in functional hits with extended variable loops; G60 is in direct contact with the variable loop and subsequent work showed that the mutation of G60 is necessary for enhanced activity and orthogonality of at least two extended variable loop sequences.^155^

Subsequent experiments have probed the nucleotides in A^Δ^-a*lv*tRNA^Pyl^ that may confer specificity for A^Δ^-a*lv*PylRS.^514^ The base pairs G3:C70, G5:C68, and the wobble pair G28:U42 in A^Δ^-a*lv*tRNA^Pyl^ were replaced with the corresponding base pairs from N-*Mm*tRNA^Pyl^, and the canonical U8 base from N-*Mm*tRNA^Pyl^ was inserted between the D loop and acceptor stem in A^Δ^-a*lv*tRNA^Pyl^. tRNAs bearing these substitutions and insertions were acylated less efficiently with A^Δ^-a*lv*PylRS, which led to the hypothesis that the nucleotides at these positions of A^Δ^-a*lv*tRNA^Pyl^ may be identity elements for A^Δ^-a*lv*PylRS. Transplanting the putative identity elements of A^Δ^-a*lv*tRNA^Pyl^ into N-*Mm*tRNA^Pyl^ led to its acylation by A^Δ^-a*lv*PylRS. The introduction of a G:C base pair in place of A26:U44 in N-*Mm*tRNA^Pyl^ further increase the activity with A^Δ^-a*lv*PylRS both, *in vitro* and *vivo*.

Superimposing the structures of A^Δ^-a*lv*tRNA^Pyl^ and N-*Mm*tRNA^Pyl^, obtained from cryogenic electron microscopy, provided a picture of how the absence of U8, and presence of the G28:U42 wobble pair, in A^Δ^-a*lv*tRNA^Pyl^ may alter the fold of this tRNA with respect to N-*Mm*tRNA^Pyl^.^124^ Molecular dynamics simulations suggested that G-C base pairs in the acceptor stem (G3:C70 and G5:C68) rigidifies A-a*lv*tRNA^Pyl^ with respect to N-*Mm*tRNA^Pyl^ which contains A-U pairs at these positions. The authors suggested that tRNA shape and rigidity may contribute to synthetase specificity.

Introducing mutations that confer specificity for *N*^ε^-((((1*R*,8S,9r)-bicyclo[6.1.0]nonan-9-yl)methoxy)carbonyl)-*L*-lysine (**BCNK**) or **CbzK** in N^+^-*Mm*PylRS into the active site of A^Δ^-*alv*PylRS generated **BCNK** or **CbzK** specific version of the enzyme; this demonstrated that, in some cases, the specificity of new PylRS systems may be altered to recognize new ncAAs by transplanting mutations from previously engineered PylRS systems into the homologous active sites of new PylRS systems. Additional studies of the A^Δ^-a*lv*PylRS/A-*alv*tRNA^Pyl^ pair have further investigated its substrate scope, and the scope of active site transfer for generating new ncAA specificity.^9,49,241,501,515−517^

N^+^-*Mb*PylRS systems had also previously been discovered with mutually orthogonal active sites. A **CbzK** specific mutant recognized **CbzK** but not *N*^ε^-(((2-methylcycloprop-2-en-1-yl)methoxy)carbonyl)-*L*-lysine (**CypK**), and wt N^+^-*Mb*PylRS recognized **CypK** but not **CbzK**. Transplanting the **CbzK** specific mutations into A^Δ^-*alv*PylRS generated a A^Δ^-*alv*PylRS variant that directed the incorporation of **CbzK** but not **CypK**, while N^+^-*Mm*PylRS directed the incorporation of **CypK** but not **CbzK**.^38^ This demonstrated that active sites of mutually orthogonal PylRS/tRNA^Pyl^ pairs can be diverged to generate mutually orthogonal ncAA substrate specificity.

To direct the incorporation of two distinct ncAAs into a protein in *E. coli* the anticodon of N-*Mm*tRNA^Pyl^ was expanded to decode a quadruplet codon (AGGA). The resulting N^+^-*Mm*PylRS/N-*Mm*tRNA^Pyl^_UCCU_ and engineered A^Δ^-a*lv*PylRS/A-*alv*tRNA^Pyl(6)^_CUA_ pair were used to incorporate **CypK** and **CbzK** in response to AGGA and UAG codons on an orthogonal message decoded by an orthogonal quadruplet decoding ribosome.^38^ Overall, this work engineered highly active mutually orthogonal PylRS/tRNA pairs to incorporate distinct ncAAs into proteins in response to distinct codons.

#### 8.2.3 Mutually Orthogonal PylRS/tRNA^Pyl^ Pairs in Mammalian Cells

The mutually orthogonal N^+^-*Mm*PylRS/N*-Mm*tRNA^Pyl^ and A^Δ^-a*lv*PylRS/A-a*lv*tRNA^Pyl^ pairs developed in *E. coli*(38) were also orthogonal and mutually orthogonal in mammalian cells.^91^ This demonstrated that directed evolution and screening platforms in *E. coli* could be exploited to create mutually orthogonal PylRS/tRNA^Pyl^ pairs for use in higher organisms. Independent work generated mutually orthogonal PylRS/tRNA^Pyl^ pairs from N^+^-*Mm*PylRS/N*-Mm*tRNA^Pyl^ and A^Δ^-a*lv*PylRS/A-a*lv*tRNA^Pyl^ by testing variable loop mutants of A-a*lv*tRNA^Pyl^ in mammalian cells.^154^ This route was lower throughput than the selection in *E. coli*, but still led to solutions with some activity in mammalian cells. However, low throughput screening in mammalian cells could not discover the spontaneous mutations that enhanced the activity of the mutually orthogonal A-a*lv*tRNA^Pyl^ variants from the *E. coli* selection.^38^

The insights gained during the *E. coli* evolution of A-a*lv*tRNA^Pyl^ permitted the rational engineering of improved mutual orthogonality for the N^+^-*Mm*PylRS/N*-Mm*tRNA^Pyl^ and A^Δ^-*g1*PylRS/A-*g1*tRNA^Pyl^ pairs in mammalian cells. The natural mutual orthogonality of these pairs had previously been established *in E. coli*,^38^ but low-level acylation of A-*g1*tRNA^Pyl^ by N^+^-*Mm*PylRS remained.

To minimize this mis-acylation, the acceptor stem sequence of A-*g1*tRNA^Pyl^ was converted to the acceptor stem sequence of A-a*lv*tRNA^Pyl^, and the G60A mutation and extended variable loop sequence discovered in selections with A-a*lv*tRNA^Pyl^ were transplanted into A-*g1*tRNA^Pyl^. The resulting A-*g1*tRNA^Pyl^ derivative showed improved orthogonality with respect to N^+^-*Mm*PylRS, while retaining most of its activity with A^Δ^-*g1*PylRS. These pairs were further engineered to recognize distinct ncAAs and decode distinct stop codons in mammalian cells, providing the basis for an approach for dual fluorescent labeling of proteins in mammalian cells.^155^

### 8.3 Discovery and Engineering of Triply Orthogonal PylRS/tRNA^Pyl^ Pairs

Interrogation of the sequence and function of 11 PylRS/tRNA^Pyl^ pairs from the ΔN group (including newly identified PylRS and tRNA^Pyl^ genes) led to the discovery of triply orthogonal PylRS/tRNA^Pyl^ pairs.^39^

Two distinct clusters emerged when the 11 tRNA^Pyl^ sequences from the ΔN group were clustered. One cluster (termed sequence class A – containing A-*alv*tRNA^Pyl^, an engineered version of which together with A^Δ^-*alv*PylRS forms a mutually orthogonal pair with the class N pair N^+^-*Mm*PylRS/N-*Mm*tRNA^Pyl^) contained four pyl tRNAs. The other cluster (termed sequence class B) contained seven pyl tRNAs. Interestingly, the bases conserved within the tRNA^Pyl^ sequences of each sequence class mainly occurred in the T-stem, T-loop, and acceptor stem; the latter two sequence motifs form important contacts with PylRS enzymes. When using the ΔN-PylRS sequences for hierarchical clustering, the same clusters emerged as seen for pyl tRNAs.^39^ These observations suggested that aaRS and tRNA sequences in the two classes might have distinct molecular recognition properties.

The measured specificities of the synthetases and tRNAs were generally well correlated with the observed clustering of their sequences: class A synthetases preferentially acylated class A pyl tRNAs over class B pyl tRNAs, and class B synthetases preferentially acylated class B pyl tRNAs over class A pyl tRNAs. Synthetases and pyl tRNAs were grouped into functional classes A and B on the basis of their activity. The N^Δ^-*term*PylRS/*term*tRNA^Pyl^ pair was an interesting exception; the PylRS of this pair is exceptionally orthogonal and only interacts with its cognate *term*tRNA^Pyl^, while the *term*tRNA^Pyl^ of this pair is a substrate for all tested N^Δ^-PylRS enzymes. The screen resulted in the discovery of 18 naturally mutually orthogonal PylRS/tRNA^Pyl^ pairs, each composed of an A^Δ^-PylRS/A-tRNA^Pyl^ pair, and a B^Δ^-PylRS/B-tRNA^Pyl^ pair.^39^

The correlation between classes derived from the hierarchical clustering of the tRNA^Pyl^ sequences, and the functional analysis of the N^Δ^-PylRS/tRNA^Pyl^ pairs hints at distinct identity elements for each class of pyl tRNAs. Those identity elements likely facilitate the class-specific tRNA^Pyl^ recognition by the cognate N^Δ^-PylRS enzymes, which in itself will be encoded in the amino acid sequence of the synthetases.^39^

**Figure 24:**
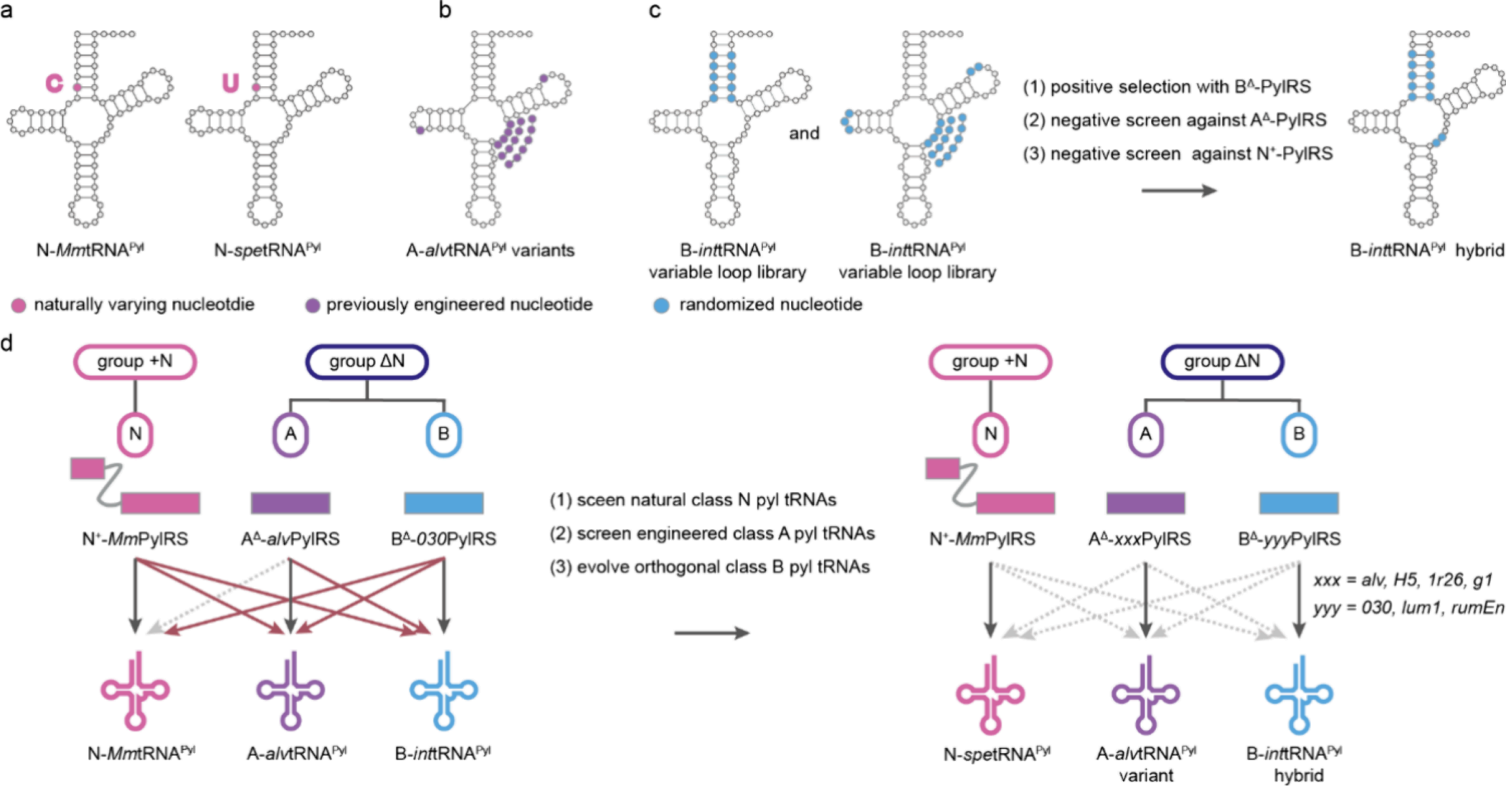
**Engineering triply orthogonal PylRS/tRNA**^**Pyl**^**pairs in***E. coli*. **a**, The screen of natural variance in N-pyl tRNAs led to the discovery that N-*spe*tRNA^Pyl^ is for class N tRNA^Pyl^ triply orthogonal sets. **b**, Depiction of previously engineered A-*alv*tRNA^Pyl^ variable loop extension mutants.^38^ Three mutants fulfilled the requirements to form the A-pyl tRNAs in triply orthogonal sets. **c**, Directed evolution strategy for identifying B-*int*tRNA^Pyl^ variants completing the triply orthogonal set. Acceptor stem and variable loop libraries were run independently and the most orthogonal B-*int*tRNA^Pyl^ variants combined into hybrid B-pyl *int*tRNAs. Multiple B-pyl *int*tRNAs fulfilled the orthogonality requirement to form triply orthogonal sets when paired with a B^Δ^-PylRS, a select class A N^Δ^ PylRS/tRNA^Pyl^ pair, and N+*Mm*PylRS/N-*spe*tRNA^Pyl^. **d**, Summary of the experimental strategy to generate triply orthogonal PylRS/tRNA^Pyl^ pairs including a depiction of all interactions that were controlled in the process (red arrows indicate undesired activity, gray dashed arrows orthogonality). Adapted with permission from Dunkelmann et al.^39^ - copyright ©2020 Nature Springer Limited.

It was postulated that triply orthogonal PylRS/tRNA^Pyl^ pairs could be engineered by combining the mutually orthogonal pairs derived from class N and class A, with a pair from the newly discovered class B. By screening several natural class N pyl tRNAs the authors discovered four N-pyl tRNAs which retained activity with the N^+^-*Mm*PylRS enzyme, while being orthogonal to all class A and B N^Δ^-PylRS enzymes. The cognate pair N^+^-*Mm*PylRS/ *Methanosarcina spelaei* (*spe*) N-*spe*tRNA^Pyl^ resulted in high activity and formed the first pair of the triply orthogonal set. Interestingly, N-*spe*tRNA^Pyl^ contains only one change in sequence with respect to N-*Mm*tRNA^Pyl^, the Watson–Crick pair C6:G67 is converted wobble pair U6:G67; this change is sufficient to render N-*spe*tRNA^Pyl^ entirely orthogonal to class A and B N^Δ^-PylRS enzymes (Figure 24a).^39^

To generate triply orthogonal class A pyl tRNAs, high cross reactivity with class N PylRS was controlled. Three A-*alv*tRNA^Pyl^ variants with expanded variable loops fulfilled the criteria for orthogonality,^38^ providing A-tRNA^Pyl^ variants which were only active with class A N^Δ^-PylRS enzymes and orthogonal to PylRS enzymes from class B, and class N, as required for the triplet set (Figure 24b).

Finally, B-pyl tRNAs were engineered to control potential cross reactivities with both, class N, and class A PylRS enzymes. This was achieved through parallel screening of acceptor stem and variable loop libraries, followed by the combination of the best hits from each library into hybrid B-pyl tRNAs. This led to the identification of several triply orthogonal B-*Candidatus Methanomassiliicoccus intestinalis* (*int*)tRNA^Pyl^ variants (Figure 24c). Overall, this work generated 12 sets of engineered, triply orthogonal PylRS/tRNA^Pyl^ pairs. Each set of triply orthogonal pairs was composed of pairs derived from class A, class B and class + N (Figure 24d).^39^

The triply orthogonal pairs: N^+^-*Mm*PylRS/N-*spe*tRNA^Pyl^_CUA_, *Candidatus Methanomethylophilus sp. 1r26* (*1r26*) A^Δ^-*1r26*PylRS(CbzK)/A-*alv*tRNA^Pyl-8^_UACU_, and B^Δ^-*lum1*PylRS(NmH)/B-*int*tRNA^Pyl-a17-vC10^_UCCU_, in which the synthetases had been altered to recognize distinct ncAAs and the anticodons had been altered to read distinct quadruplet codons or the amber codon, were used to incorporate three distinct ncAAs into a protein. These experiments were performed in *E. coli* cells bearing an orthogonal message, O-_*Strep*_*GFP(40TAG, 136AGGA, 150AGTA)*_*His6*_, and an orthogonal ribosome (ribo-Q1) that reads this message and efficiently decodes amber and quadruplet codons.^39^ When an engineered set of triply orthogonal PylRS/tRNA^Pyl^ pairs was later paired with computationally designed, highly active orthogonal mRNAs 2.6 ± 0.4 mg L^–1^ of GFP protein containing three distinct ncAAs was isolated (approximately 9% of wt GFP yield).^518^

Furthermore, the triply orthogonal set composed of N^+^-*Mm*PylRS/N-*spe*tRNA^Pyl^_UCUA_, A^Δ^-*g1*PylRS(CbzK)/A-*alv*tRNA^Pyl-8^_UACU_, and *Methanomassiliicoccales archaeon RumEn M1* (*rum*) B^Δ^-*rum*PylRS(NmH)/B-*int*tRNA^Pyl-a17-vC10^_UCCU_, combined with the orthogonal *Archaeoglobus fulgidus* (*Af*) *Af*TyrRS(PheI)/*Af*tRNA^Tyr-A01^_CUAG_, enabled the production of a GFP protein with four distinct ncAAs in its sequence. Each ncAA was incorporated in response to a quadruplet codon in a computationally designed O-mRNA read by ribo-Q1, thereby realizing a 68-codon code to incorporate four distinct ncAAs.^518^ The protein yield was 0.41 ± 0.03 mg L^–1^ (approximately 2% of wt GFP yield); this corresponds to an average quadruplet decoding efficiency of 38% per quadruplet, highlighting the suitability of engineered pyl tRNAs for decoding quadruplet codons.

### 8.4 Discovery and Engineering of Quintuply Orthogonal PylRS/tRNA^Pyl^ Pairs

The sequence-activity data gathered from screening the amber suppression efficiency of PylRS/tRNA^Pyl^ pairs, in which the (class A or class B) PylRS enzyme and tRNA^Pyl^ are from different organisms, revealed clear sequence thresholds for orthogonality. When two PylRS C-terminal domain sequences from different organisms share a sequence identity of 55% or more, the likelihood of a PylRS enzyme being active with the tRNA^Pyl^ from the other organism is approximately 90%. PylRS enzymes with lower sequence identity than 55% in their C-terminal domain may or may not form active pairs with the other tRNA^Pyl^. Similarly, when two pyl tRNAs from different organisms share a 75% or higher sequence identity, the corresponding PylRS enzymes will aminoacylate the tRNA^Pyl^ from the other organism in approximately 90% of cases. The PylRS enzymes of pyl tRNAs which share lower than 75% sequence identity, may or may not aminoacylate the other tRNA^Pyl^.

**Figure 25:**
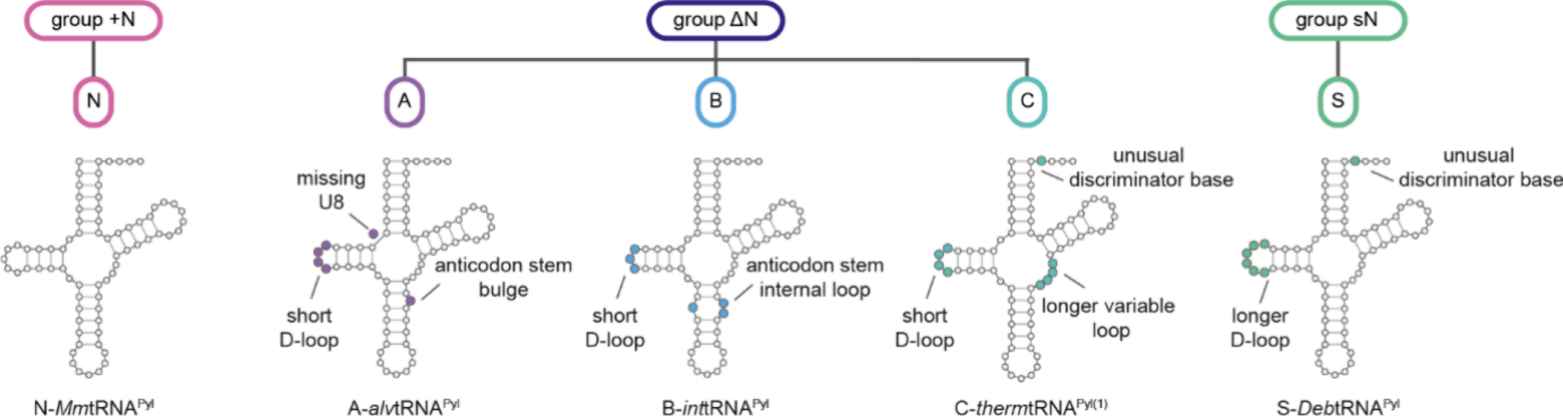
**The archetypical tRNA structures for pyl tRNAs of classes N, A, B, C, and S.** Important nucleotides and features are highlighted in the class-specific colors and labeled. Adapted with permission from Beattie et al.^40^*-* copyright © 2023 Nature Springer Limited.

PylRS C-terminal domain sequences, in a database of 351 sequences, were clustered according to the experimentally determined sequence identity thresholds for synthetase orthogonality, leading to 37 distinct clusters. Twenty-seven clusters belonged to the Ns group (25 of bacterial origin, and two of archaeal origin) and seven belonged to the archaeal ΔN group. Pyrrolysyl tRNAs, from the same organism as representative PylRS sequences, were identified for 95% of the PylRS clusters. These tRNAs were clustered using the experimentally determined threshold for tRNA orthogonality, leading to two new pyl classes. Class C, which constituted a loosely related group of six pyl tRNAs from the ΔN group, and class S, which was composed of all 25 bacterial members of the N group. Sixteen pyl tRNAs (and their synthetases) – including both representative members of all tRNA clusters and tRNAs selected for their unusual structural features (Figure 25) – were selected for experimental characterization. Out of the 16 PylRS/tRNA^Pyl^ pairs, three originated from the previously characterized classes A, B, and N, seven from the bacterial class S, and six from the archaeal class C.

The PylRSn domain of multiple class S PylRS enzymes remained recalcitrant to cloning, and even in the instances of successful class S^+^-PylRS assembly into expression constructs, the transformed *E. coli* strains displayed attenuated growth phenotypes. To remove the toxicity effects, the researchers engineered the synthetic class S^Δ^ for all S^+^-PylRS enzymes by removing the N-terminal domains from the constructs.

The amber suppression activity of the selected pyl tRNAs with selected synthetases were measured in an activity screen. Fifteen out of 16 pyl tRNAs resulted in **AllocK** dependent amber suppression, when paired with at least one PylRS enzyme. Thirteen out of 20 (including four instances where group S PylRS enzymes were tested with and without N-terminal domain) tested PylRS enzymes led to amber decoding dependent GFP production at a level at least 30% of the wt GFP. Forty-six naturally mutually orthogonal pairs emerged from these experiments. Mutually orthogonal pairs that used the same set of PylRS enzymes but different pyl tRNAs were defined as belonging to the same family. There were 15 families of mutually orthogonal pairs, and one family of triply orthogonal pairs. Overall, class S^Δ^ PylRS enzymes did not form an independent reactivity pattern, but were classified as B-like (S^ΔB^), or C-like (S^ΔC^).

To engineer quintuply orthogonal sets of PylRS/tRNA^Pyl^ pairs from the five pyl classes, 25 pairwise interactions between PylRS and pyl tRNAs needed to be controlled (five cognate interactions, 20 noncognate interactions). At the interclass and intraclass level, 20 of desired pairwise interactions were exemplified in the activity screen, while in the five pairwise interactions that were not fully exemplified at the class level from the screen, only one of two interactions remained to be controlled. This data did not provide specific pairs within each class that exemplified all the properties that could be found within the class as a whole.

**Figure 26:**
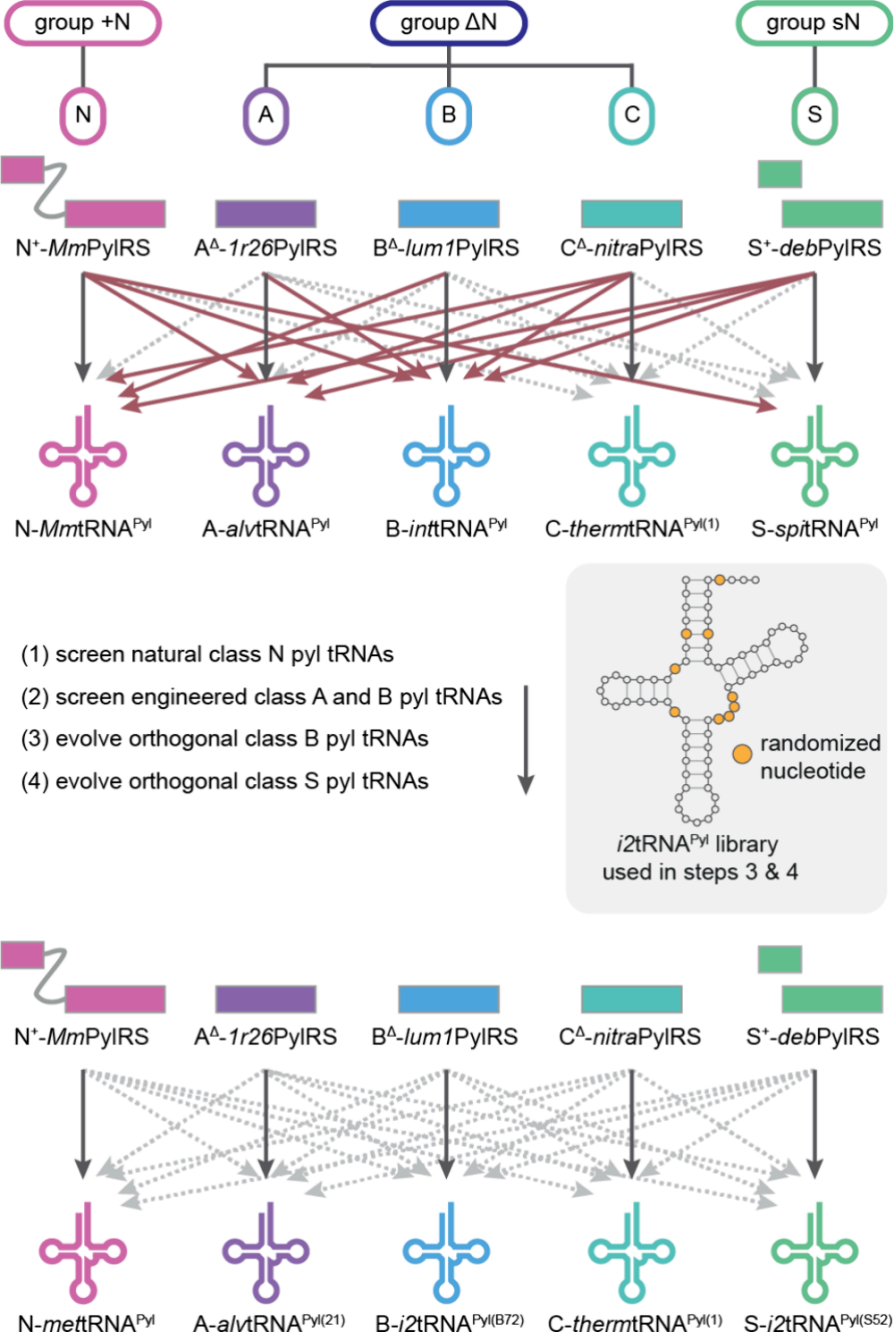
**Engineering quintuply orthogonal PylRS/tRNA**^**Pyl**^**pairs in*****E. coli*****.** Summary of the experimental strategy to generate quintuply orthogonal PylRS/tRNA^Pyl^ pairs including a depiction of all interactions that were controlled in the process (red arrows indicate undesired activity, gray dashed arrows orthogonality). Starting from a rationally chosen set of five PylRS/tRNA^Pyl^ pairs composed of one pair of each pyl class N, A, B, C and S, a series of screens and directed evolution experiments resulted in the generation of quintuply orthogonal sets. Quintuply orthogonal pyl tRNAs for B^Δ^-PylRS and S^+^-PylRS, respectively, were identified by a round of positive selection of the depicted tRNA^Pyl^ library, which is based on the S-*i2*tRNA^Pyl^ scaffold, in the presence of either B^Δ^-PylRS or S^+^-PylRS followed by negative screens against all other classes of PylRS enzymes (N, A, C and S or N, A, B and C, respectively).

A set of PylRS/tRNA^Pyl^ pairs, with one pair from each of the five classes was defined as the starting point for creating a set of quintuply orthogonal pairs (Figure 26). To control all pairwise interactions, within and between five specific PylRS/tRNA^Pyl^ pairs a sequence of screens and directed evolution experiments were performed (Figure 26). This process led to the discovery of 924 mutually orthogonal pairs in 22 families, 1,324 triply orthogonal pairs in 18 families, 128 quadruply orthogonal pairs in 7 families, and 8 quintuply orthogonal pairs in 1 family. All of the newly engineered PylRS/tRNA^Pyl^ sets, met or exceeded, the orthogonality of all previous triply orthogonal pairs. It is remarkable that while no mutually orthogonal pair has been discovered within any other isoacceptor class the pyl isoacceptor class has provided quintuply orthogonal pairs. Future work may aim to develop these pairs to decode distinct codons and incorporate distinct ncAAs or ncMs.

## 9 Directing pyl Systems to New Codons

PylRS enzymes do not recognize the anticodon of pyl tRNAs (Section 3.6),^60,63,75^ which has enabled pyl tRNAs to be directed to different codons, including stop codons (Section 9.1) and quadruplet codons (Sections 9.2), codons with noncanonical bases (Section 9.3) and sense codons (Section 9.4). Directing PylRS/tRNA^Pyl^ to sense codons provides a basis for proteome labeling strategies (Section 9.4.1). These approaches–in combination with strategies for generating mutually orthogonal synthetases and tRNAs (Section 8), strategies for generating orthogonal ribosomes that more efficiently read quadruplet codons and stop codons, and the synthesis of genomes with compressed genetic codes–have enabled the incorporation of multiple distinct ncAAs into a protein (Section 10) and the encoded synthesis of entirely non canonical polymers (Section 11).

### 9.1 Directing ncAA Incorporation at a Stop Codon

The absence of anticodon recognition by PylRS enzymes permitted the facile reassignment of tRNA^Pyl^_CUA_ to the two alternative stop codons, the opal (UGA) and ochre (UAA) codon in *E. coli*.^75^

### 9.2 Directing ncAA Incorporation at a Quadruplet Codon

As part of an *in vitro* screen for quadruplet decoding tRNAs, chemically acylated N-*Mac*tRNA^Pyl^ variants with extended anticodons were used to decode quadruplet codons.^519,520^ Later, evolution of N-*Mm*tRNA^Pyl^_UCCU_ variants for improved AGGA decoding in cells demonstrated that directed evolution experiments could improve quadruplet decoding of N-*Mm*tRNA^Pyl^_UCCU,_ and the selected N-*Mm*tRNA^Pyl^_UCCU_ variants enabled quadruplet decoding in mammalian cells.^80^ Recently, a quadruplet decoding tRNA^Pyl^ variant has been used to incorporate ncAAs in response to a quadruplet codon introduced into *C.elegans*(165) and quadruplet decoding N-pyl *Mm*tRNAs have been optimized for the decoding of UAGA, AGGA, AGUA, and CUAG on wt messages in mammalian cells.^521^ The reliance of these approaches on the natural ribosome, which does not efficiently decode quadruplet codons, may have limited the discovery of highly active, quadruplet decoding pyl tRNAs in these systems.^76^

N-*Mb*tRNA^Pyl^ was systematically evolved for enhanced decoding of UAGA, AGGA, AGUA, and CUAG codons on orthogonal messages by the orthogonal quadruplet decoding ribosome RiboQ1 (O-RiboQ1) in *E. coli*.^406^ These quadruplet codons were chosen because the triplets that form the first three bases of the quadruplet are not recognition elements for endogenous aaRS enzymes in *E. coli*. Eight bases at positions flanking the extended anticodon, in the anticodon stem and anticodon loop of N-*Mb*tRNA^Pyl^_XXXX_, (tRNAs with fixed sequence extended anticodons) were randomized. The resulting libraries were subjected to a double-sieve selection to select against tRNAs that did not function with endogenous synthetases, and to select for tRNAs that decoded their cognate codon when provided with N^+^-*Mb*PylRS. For each quadruplet codon, an evolved N-*Mb*tRNA^Pyl^_XXXX_ mutant that worked with Ribo-Q1 to decode its cognate codon on an orthogonal message was identified.

### 9.3 Directing ncAA Incorporation at Codons with Noncanonical Bases

**Figure 27:**
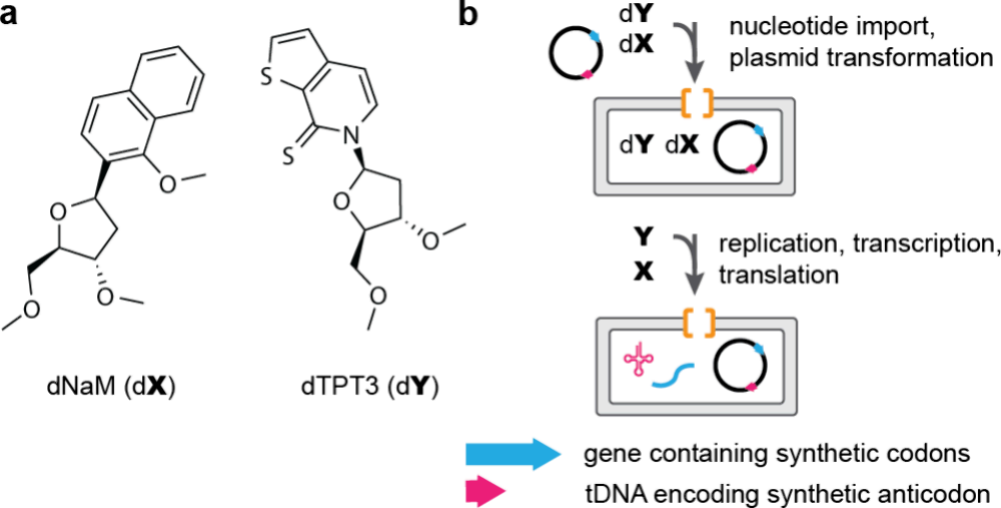
Transcription and translation with synthetic bases. **a**, Structure of synthetic base pair (d)NaM(d**X**):(d)TPT3(d**Y**). **b**, A heterologous nucleotide transporter enables the uptake of the triphosphate of d**X**, d**Y, X and Y** in *E. coli*. A plasmid harboring a tRNA gene with an anticodon containing a synthetic codon, as well as a gene using a synthetic codon in its sequence is transformed and maintained in *E. coli*. The gene and tRNA harboring synthetic codons are transcribed and used by the ribosome in translation. Adapted with permission from de la Torre et al.^2^ - copyright © 2021 Nature Springer Limited.

A body of work has developed non-natural “bases pairs” that do not pair with natural bases, and interact with each other in a variety of ways, including hydrophobic stacking and cross intercalation.^2^ The **NaM**:**TPT3** pair (Figure 27a) was incorporated into a plasmid at the second position of the anticodon of N-*Mm*tRNA^Pyl^ gene and in the second position of a codon in a GFP gene. The (deoxy) “nucleotide” triphosphates of **NaM** and **TPT3** were taken up by *E. coli*, provided with a heterologous nucleotide triphosphate transporter, and the deoxyNaMtriphosphate (**dNaMTP**) and deoxyTPT3triphosphate (**dTPT3**) were used to replicate the plasmid containing the non-natural base pair.^78^ While substantial loss of the base pair occurred through replication and DNA repair, this loss could be counteracted to some extent by targeting the sequences resulting from repair for Cas9 cleavage.^78^ T7 transcription through the non-natural base pair (using **NaMTP** and **TPT3**) produced N-*Mm*tRNA^Pyl^ with a non-natural base at position 2 of its anticodon and a GFP mRNA with the complementary non-natural base at position 2 of a codon. Translation with the N^+^-*Mm*PylRS, the engineered N-*Mm*tRNA^Pyl^, and engineered GFP mRNA enabled protein production; 90% of the resulting protein incorporated a ncAA substrate of PylRS in response to the codon with the noncanonical base (Figure 27b). The authors have explored putting non-natural bases at different positions of the codon anticodon interaction using N-*Mm*tRNA^Pyl^ variants; the **NaM:TPT3** pair was not tolerated at the first or third position of the codon, but a **NaM:NaM** homopair between the third position of the codon and the third position of the anticodon was tolerated in some contexts.^79^

The orthogonality of codon anticodon interactions with non-natural bases at position 2 or 3 of the codon, and different natural bases at the remaining positions the codon, were explored using variant N^+^-*Mm*PylRS/N-*Mm*tRNA^Pyl^ pairs and an azido containing pyl analogue as a substrate. This screen identified a triply orthogonal set of codon anticodon interactions: AXC:GYT; GXT:AYC; AGX:XCT, where X= **NaM** and Y= **TPT3**, and the sequences are written codon 5′-3′:anticodon 5′-3′.^79^

### 9.4 Efforts to Direct ncAAs at Sense Codons

*In vitro* studies demonstrated that the anticodon of N^+^*Mb*tRNA^Pyl^ can be changed to various sense codons without apparent effects on aminoacylation efficiency by PylRS enzymes.^58,75^ Sense codons cannot commonly be reassigned to arbitrary ncAAs in cells for long periods. The sustained efficient reassignment of genomic sense codons to ncAAs leads to proteome mis-synthesis, and target genes containing sense codons are decoded by essential cellular tRNAs. Efforts to reassign the rare arginine codon AGG to homoarginine in *E. coli*, combined an engineered N^+^-*Mm*PylRS/N-*Mm*tRNA^Pyl^_CCU_ with temperature-dependent depletion of the endogenous *Ec*tRNA^Arg^_UCU_ that normally reads genomic AGG codons, and the recoding of 38 AGG codons in 32 essential genes to synonymous codons. Since homoarginine was somewhat tolerated in place of arginine in the genomically encoded proteins, this approach permitted the incorporation of homoarginine into a target protein.^522^ However, this approach is not efficient or generalizable. Efforts to reassign sense codons with PylRS/tRNA^Pyl^ pairs in wt cells have not yielded homogeneous proteins.^523,524^ The synthesis of an *E. coli* genome that compressed the number of sense codons used to encode the canonical amino acids, and enabled deletion of the tRNAs that normally decode the removed synonyms, has been used to cleanly encode ncAAs in response to sense codons in synthetic genes.^77,525^ This work is discussed in the context of incorporating multiple distinct ncAAs (Section 10).

#### 9.4.1 Stochastic Orthogonal Recoding of Translation

Methods for selectively tagging and identifying the proteome synthesized in specific cells and/or at specific times have proved very useful for deciphering a range of biological processes.^166,167,526,527^ The N^+^-*Mm*PylRS/N-*Mm*tRNA^Pyl^ pair has been adapted to direct the incorporation of ncAAs at substoichiometric levels into the proteome in response to sense codons. Since PylRS does not recognize the anticodon of its cognate tRNA, ncAAs can be directed in response to diverse sense codons. This approach, known as stochastic orthogonal recoding of translation (SORT), enables ncAA-dependent tagging of the proteome in response to diverse codons. Stochastic orthogonal recoding of translation complements approaches that use endogenous methionyl synthetases (or their mutants) and close analogs of methionine to label the proteome at methionine codons by selective pressure incorporation-based methods, and has several potential advantages over these methods. First, SORT uses an orthogonal synthetase and therefore ncAA incorporation efficiency, and the chemical structure of the ncAAs used, is not limited by the active sites of natural synthetases; this means that SORT can label proteomes with diverse chemistry and does not require minimal media or starvation. Second, SORT can be directed to diverse codons and therefore proteome coverage may be enhanced. Third, because PylRS/tRNA^Pyl^ pairs are orthogonal in all domains of life the approach is portable to many organisms.

Two versions of SORT were demonstrated in *E. coli*; SORT with chemo-selective modification (SORT-M) tags the newly synthesized proteome with ncAAs that are then labeled with fluorophores to visualize proteome synthesis, while SORT-E tags the newly synthesized proteome with ncAAs that are then labeled with biotin for selective enrichment and identification by MS. SORT-M and SORT-E have been implemented via incorporation of an alkyne containing ncAA or a cyclopropene containing ncAA and labeling with azide or tetrazine based probes, respectively.^96,166^

SORT-M was extended to *D. melanogaster*, where cell-type specific promoters were used to drive N^+^*Mm*PylRS to tag the proteome and visualize newly synthesized proteins in specific cells at specific developmental stages.^167^ Adeno-associated virus based expression of the SORT components, with N^+^*Mm*PylRS driven by cell-type specific promoters, enabled the use of SORT-M and SORT-E to investigate the proteomes of neuronal cultures, brain slices, and spatially and genetically defined regions of the brains of live mice.

Recent developments in the generation of mutually orthogonal PylRS/tRNA^Pyl^ pairs (Section 8),^38^ may enable further advances in SORT. Mutually orthogonal chemical handles may be encoded in response to distinct codons and in distinct tissues at distinct times. These advances should enable multicolor pulse-chase proteome labeling, and multicolor cell-type specific labeling.

## 10 Incorporating Multiple Distinct ncAAs into Proteins with pyl Systems

Strategies for incorporating distinct ncAAs in response to distinct codons have extensively utilized engineered PylRS/tRNA^Pyl^ pairs in combination with other orthogonal aminoacyl-tRNA synthetase/tRNA pairs. The codons used include a quadruplet codon and a stop codon, up to three stop codons, sets of quadruplet codons (Section 10.1), codons containing noncanonical bases (Section 10.2), and sense codons (Section 10.3). An extensive review on genetic code expansion mediated double incorporations has recently been published.^410^

### 10.1 Incorporation of ncAAs with pyl Systems at Quadruplet Codons and Stop Codons

The N^+^-*Mb*PylRS/N-*Mb*tRNA^Pyl^ pair and *Mj*TyrRS/*Mj*tRNA^Tyr^_CUA_ pair were shown to be mutually orthogonal in their aminoacylation specificity.^406^ The N^+^-*Mb*PylRS/N-*Mb*tRNA^Pyl^ pair and an engineered *Mj*TyrRS/*Mj*tRNA^Tyr^_UCCU_ pair were used to incorporate two distinct ncAAs in response to an amber and quadruplet codon on an orthogonal message, decoded by O-RiboQ1 in *E. coli*. Encoding azide and alkyne containing ncAAs into calmodulin at specific sites enabled copper catalyzed protein cyclization.

The N^+^-*Mm*PylRS/N-*Mm*tRNA^Pyl^_UUA_ pair was subsequently combined with active site variants of the *Mj*TyrRS/*Mj*tRNA^Tyr^_CUA_ pair to direct the incorporation of two distinct ncAAs at an ochre and amber codon in *E. coli*.^92,528^ This approach has been extended to mammalian cells, where the N^+^-*Mm*PylRS/N-*Mm*tRNA^Pyl^_UUA_ pair was partnered with derivatives of the mutually orthogonal *Ec*TyrRS/*Ec*tRNA^Tyr^_CUA_ pair to read the ochre and amber stop codons.^401^ This approach has also been extended to all three stop codons, using a system where the opal stop codon (UGA) was read by an engineered *Ec*TrpRS/tRNA^Trp^_UCA_, in *E. coli*;^529^ in this system the endogenous *Ec*TrpRS/tRNA^Trp^_CCA_ pair is replaced by the corresponding yeast pair. Other work has used the N^+^-*Mm*PylRS/N*-Mm*tRNA^Pyl^_UUA_ pair in combination with an engineered A^Δ^-a*lv*PylRS/A-a*lv*tRNA^Pyl^_CUA_, these previously described mutually orthogonal pairs (Section 8)^38^ were used to direct the incorporation of two ncAAs in response to the cognate stop codons, and a mutant *Mj*TyrRS/*Mj*tRNA^Tyr^_AUA_ pair that acts as an initiator tRNA was used to direct a third ncAA at position 1 of GFP.^530^ In general, efforts to reassign multiple stop codons resulted in low protein yields,^76^ and this approach is toxic for cells as the termination of endogenous proteins is impaired.

Evolved N^+^-*Mb*PylRS/N*-Mb*tRNA^Pyl^_XXXX_-derived pairs with extended anticodon tRNAs that decode UAGA, AGGA, AGUA, and CUAG codons (Section 9) were combined with evolved *Mj*TyrRS/*Mj*tRNA^Tyr^_CUA_ pairs, to encode 12 distinct pairs of ncAAs, using O-RiboQ1 and a cognate orthogonal message bearing the amber codon and a quadruplet codon of interest.^76^ This approach was used to label proteins at defined sites for FRET studies of protein conformational change,^76^ and formed the basis of an approach for the concerted, rapid, and quantitative dual-labeling of proteins, using two highly active mutually orthogonal chemistries.^391^ The combination of the evolved N^+^-*Mb*PylRS/N-*Mb*tRNA^Pyl^_CUAG_ pair with an engineered *Mj*TyrRS/*Mj*tRNA^Tyr^_CUA_ pair was used to encode two distinct ncAAs in an O-RiboQ1 dependent manner into a phage-displayed single-chain antibody variable fragment, expanding the chemical scope of phage display.^380^

Engineered mutually orthogonal PylRS/tRNA^Pyl^ pairs (Section 8) have also been used to incorporate pairs of ncAAs in response to an amber and quadruplet codon. An engineered N^+^-*Mm*PylRS/N*-Mm*tRNA^Pyl^_UCCU_ pair was combined with an engineered A^Δ^-a*lv*PylRS/A-a*lv*tRNA^Pyl^_CUA_ pair to direct the incorporation of distinct ncAAs in response to an AGGA and CUA codon on an orthogonal message decoded by O-RiboQ1.^38^ In extensions of this approach, triply orthogonal PylRS/tRNA^Pyl^ pairs (Section 8) were used to incorporate three distinct ncAAs in response to two quadruplet codons and an amber codon on an orthogonal message.^39,518^

**Figure 28:**
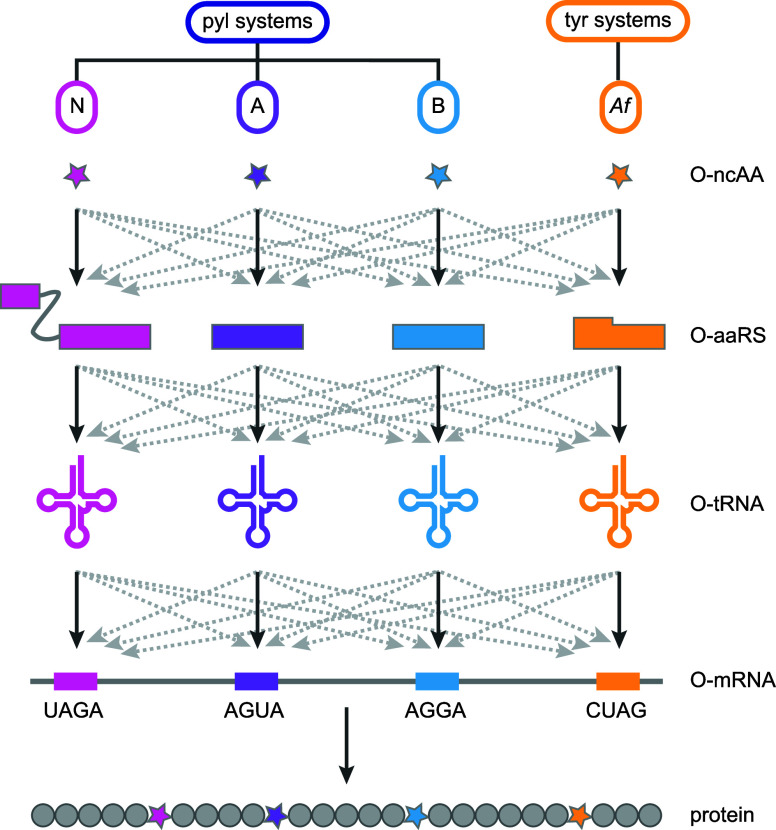
**Genetic encoding of four distinct ncAAs using a 68-codon genetic code.** The genetic incorporation of four distinct ncAAs requires the control of the orthogonality of the engineered translational machinery on several levels. First, active sites need to be engineered for each aaRS which are selective for the target ncAA, and exclude all other ncAAs as well as the canonical amino acids. Second, multiply orthogonal aaRS/tRNA pairs need to be engineered, which are compatible with each other. Third, each tRNA needs to be addressed to a distinct codon in the genetic code. Finally, the ribosome may need to be engineered to read alternative codons, or polymerize novel ncMs. By combining engineered triply orthogonal PylRS/tRNA^Pyl^ pairs with an orthogonal *Af*TyrRS/*Af*tRNA^Tyr^ pair, addressing each active site to a specific ncAA, and addressing each tRNA to a specific quadruplet codon, four distinct ncAAs were successfully encoded in *E. coli* using an evolved quadruplet decoding ribosome (RiboQ1). Adapted with permission from Dunkelmann et al.^518^ - copyright © 2021 Nature Springer Limited.

Sets of quadruplet codons have also been used to incorporate multiple distinct ncAAs into proteins. In one example, an engineered *Mj*TyrRS/*Mj*tRNA^Tyr^_UCCU_ pair was combined with an engineered N^+^-*Mb*PylRS/N*-Mb*tRNA^Pyl^_UCUA_ pair to produce a protein incorporating two distinct ncAAs in response to two quadruplet codons on an orthogonal message read by O-RiboQ1 in *E. coli*.^76^ In recent work, engineered triply orthogonal PylRS/tRNA^Pyl^ pairs were combined with an engineered orthogonal *Af*TyrRS/*Af*tRNA^Tyr^ to incorporate four distinct ncAAs in response to four quadruplet codons on an orthogonal message read by O-RiboQ1 in *E. coli*.^518^ To express all four tRNAs and aaRS enzymes in *E. coli*, an automated tRNA operon generator was developed, and strategies to express multiple aaRS as polycistronic operons from a single transcript were also developed. This work expanded the genetic code from 64, to 68 codons (Figure 28).

### 10.2 Incorporation Two Distinct ncAAs at Codons Containing Synthetic Bases

**Figure 29:**
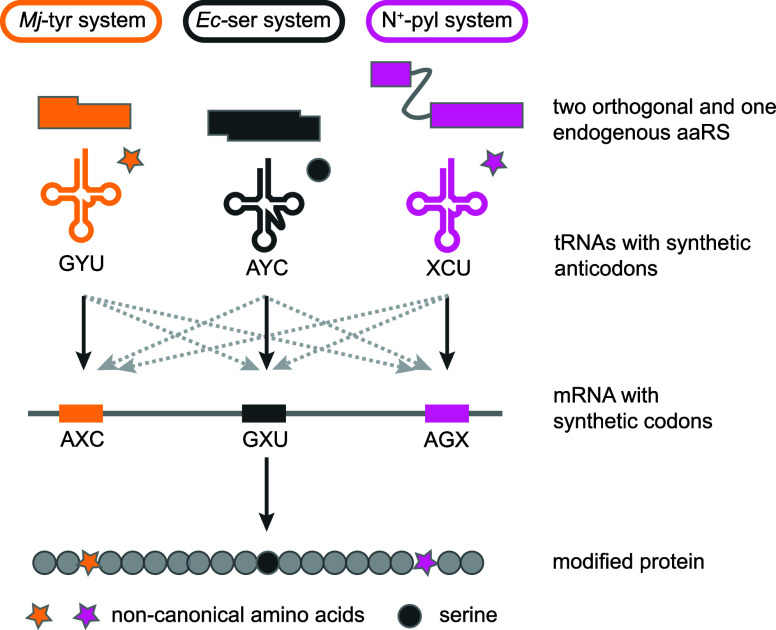
**Genetic encoding of two ncAAs using two mutually orthogonal synthetic codons.** Three synthetic codons are orthogonal to each other GYU:AXC, AYC:GXU, and XCU:AGX. The combination of the engineered *Mj*TyrRS/*Mj*tRNA^Tyr^_GYU_ and N^+^-*Mm*PylRS/N-*Mm*tRNA^Pyl^_XCU_ pairs together with an *Ec*SerRS enzyme and an *Ec*tRNA^Ser^_AYC_ variant permits the site-specific incorporation of two ncAAs and serine at synthetic-codon defined positions in a protein. In *Ec*SerRS, Ser has been typeset in non-italic.

The triply orthogonal set of codon anticodon interactions: AXC:GYU; GXU:AYC; AGX:XCU (where X= **NaM** and Y= **TPT3**, and the sequences are written codon 5′-3′:anticodon 5′-3′) discovered using N^+^-*Mm*PylRS/N-*Mm*tRNA^Pyl^ pairs (Section 9) were leveraged to incorporate two distinct ncAAs and incorporate serine in response to a noncanonical codon.^79^ To achieve this a variant of *Mj*tRNA^Tyr^ was engineered with a GYU anticodon, N-*Mm*tRNA^Pyl^ was engineered with a XCU anticodon, and *Ec*tRNA^Ser^ was engineered with an AYC anticodon. Cells containing these tRNAs, their cognate synthetases and a GFP gene with the cognate codons were used to direct two ncAAs and serine into GFP (Figure 29). Ninety six percent of the isolated protein contained all three amino acids, as judged by protein intact MS of the protein.^79^

### 10.3 Incorporation of Three Distinct ncAAs at Sense Codons

**Figure 30:**
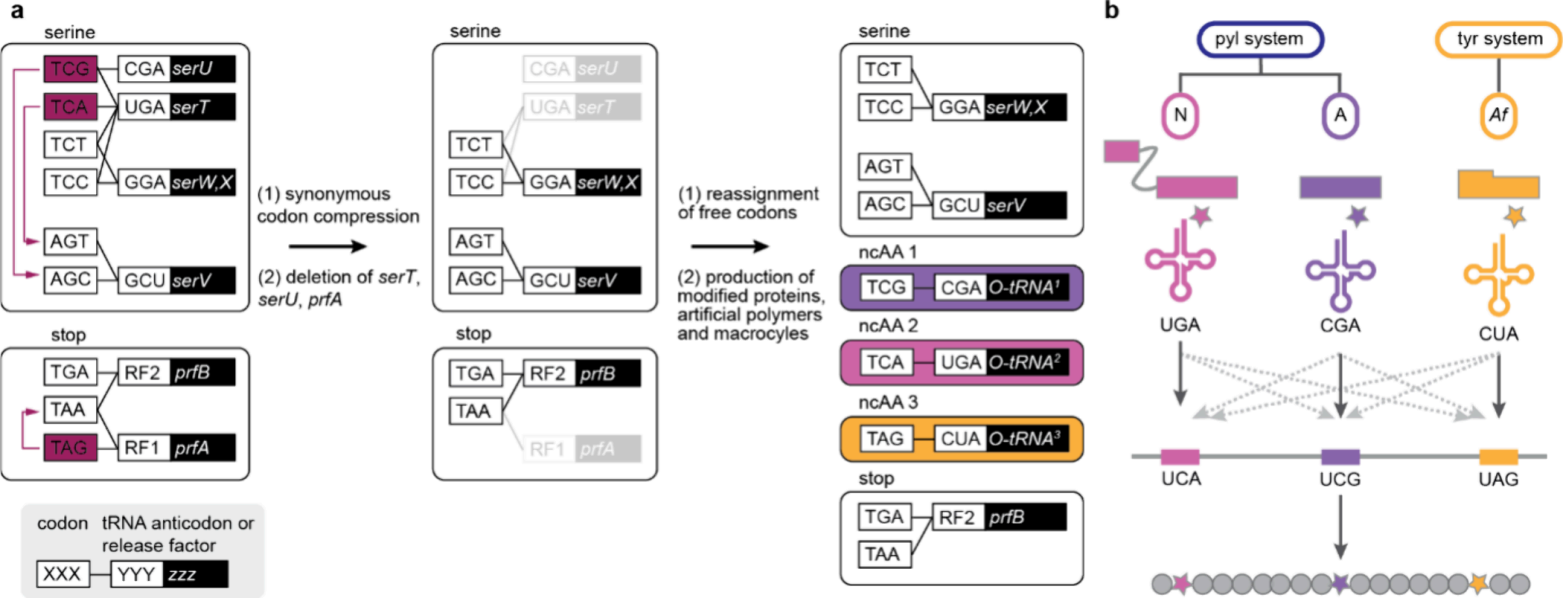
**Genetic encoding of three distinct ncAAs at sense codons in syn61Δ3. a**, Schematic showing the codon compression scheme used to generate syn61 and the steps taken to reassign all three free codons to new ncAAs. **b**, Genetic encoding of three distinct ncAAs at three distinct sense codons in syn61Δ3. Two mutually orthogonal PylRS/tRNA^Pyl^ pairs from classes N and A were used together with the *Af*TyrRS/AftRNA^Tyr^ pair. Parts of this figure are adapted with permission from Robertson et al.^77^ copyright © 2021, some rights reserved; exclusive licensee American Association for the Advancement of Science.

Recent work freed three codons, two sense codon (TCG and TCA) and the TAG codon for reassignment to ncAAs in *E. coli*. To achieve this a version of the four mega bases *E. coli* genome was synthesized in which every annotated occurrence of TCA and TCG codons was replaced with defined synonymous codons (AGC and AGT respectively); the TAG stop codon was also replaced with the TAA stop codon. The resulting strain, called syn61, contained over 18, 000 codon changes and used a compressed genetic code composed of 61 codons to encode the proteome of the cell.^525^ The endogenous tRNAs (*serT* and *serU)* that normally decode UCA and UCG codons, as well as release factor 1 (*prfA)* that normally terminates protein synthesis at TAG codons were deleted in an evolved version of Syn61, creating syn61Δ3.^525^ This freed these codons for reassignment to new monomers (Figure 30a).

Synthetic genes–that used the compressed genetic code for the canonical amino acids and the freed codons (TCA, TCG and TAG) to define the sites of ncAA incorporation–were introduced into syn61Δ3. A set of engineered triply orthogonal pairs, based on A^Δ^*-1r26*PylRS/A-*alv*tRNA^Pyl^_CGA,_ N^+^-*Mm*PylRS/N-*Mm*tRNA^Pyl^_CGA,_ and *Af*TyrRS/*Af*tRNA^Tyr^ (Section 8), were used to encode several combinations of ncAA into proteins with very high efficiency and fidelity in response to the freed codons (Figure 30b).^38,39,77,83^

## 11 Genetically Encoded Noncanonical Polymer and Macrocycle Synthesis

By writing synthetic genetic sequences composed of the freed codons, syn61Δ3 was leveraged for the genetically encoded synthesis of noncanonical polymers composed entirely of ncAAs. This required the efficient, scalable, sequential decoding of freed codons to incorporate ncAAs, which has not been achieved by other approaches used to encode ncAAs in proteins.

**Figure 31:**
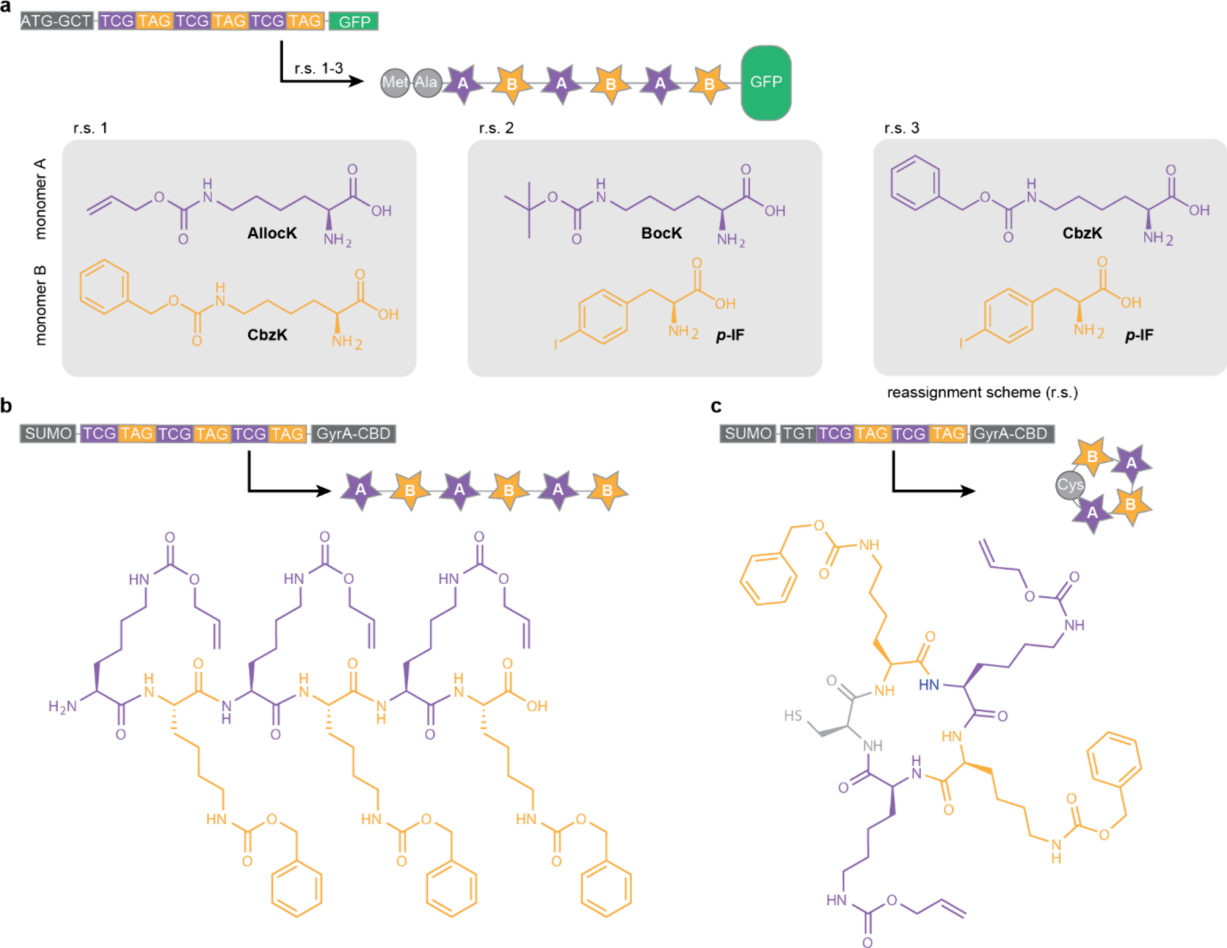
**Synthesis of noncanonical polymers in cells. a**, Synthesis of noncanonical polymers as GFP fusions. Synthetic genes for noncanonical polymers were expressed as N-terminal fusions to GFP; the order of codons in the sequence defined the pattern of monomer building blocks in the resulting polymer, one example of a synthetic gene is shown, and reassignmentschemes (r.s. 1–3) define the identity of the monomers. Two mutually orthogonal PylRS/tRNA^Pyl^ pairs from classes N and A were used for r.s. 1 and of one of the pyl pairs (from either class N or A, respectively) in combination with the *Af*TyrRS/*Af*tRNA^Tyr^ pair for r.s. 2 and 3. **b**, Synthetic genes for the encoded synthesis of free non canonical polymers. An example of a noncanonical hexamer synthesized in cells using recoding scheme 1 is shown. **c**, Synthetic genes for the encoded synthesis of a noncanonical macrocycle. An example of a cell-based noncanonical macrocycle synthesis using recoding scheme 1 is shown. Figure adapted with permission from Robertson et al.^77^ copyright © 2021, some rights reserved; exclusive licensee American Association for the Advancement of Science.

The encoded synthesis of noncanonical polymers composed of two distinct ncAAs or ncMs (A and B) requires the ribosome to accept each ncAA (or ncM) in four elementary polymerization steps (A+B → AB, B+A → BA, A+A → AA, B+B → BB); these steps are not investigated when incorporating a ncAA or ncM into a protein. These elementary reactions were demonstrated in response to four dicodon combinations inserted into GFP; this was done using three pairs of ncAAs and components of the triply orthogonal pairs based on A^Δ^*-1r26*PylRS/A-*alv*tRNA^Pyl^_CGA,_ N^+^-*Mm*PylRS/N-*Mm*tRNA^Pyl^_CGA,_ and *Af*TyrRS/*Af*tRNA^Tyr^ (Section 8). This work was further developed to encode six non-natural tetrameric sequences, and a hexameric sequence for each of the three pairs of ncAAs (Figure 31a). In addition, an octameric polymer sequence composed of **CbzK** and **AllocK** was encoded. All the encoded noncanonical polymer sequences generated were confirmed by MS.^77^ While noncanonical polymers were initially made as GFP fusions, tetramers and hexamers were subsequently produced as free molecules (Figure 31b).

**Figure 32:**
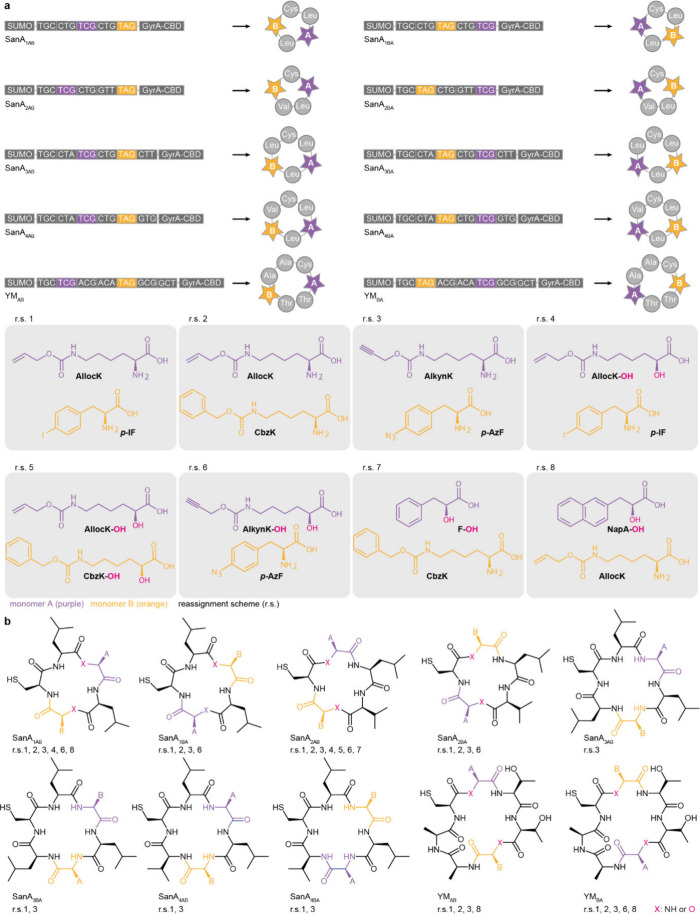
**Cell-based synthesis of macrocyclic (depsi)-peptides. a**, Strategy for the encoded cell-based synthesis of artificial macrocycles. The indicated synthetic genes were used with the indicated reassignmentschemes (r.s.). Two mutually orthogonal PylRS/tRNA^Pyl^ pairs from classes N and A as well as the *Af*TyrRS/AftRNA^Tyr^ (Y) pair were used in different combinations; r. s. 1,2, 4, 6 used a class N pyl pair and a Y pair: r. s. 2, 5, 7, 8 used a a class N pyl pair and a class A pyl pair. **b**, The ten core structures of 37 macrocyclic products from the encoded cell based synthesis that were isolated and characterized by MS. For each core structure the different recoding schemes, according to which the macrocyclic product were synthesized, are indicated. Adapted with permission from Spinck et al.^132^ - copyright © The Author(s) 2023 CCBY http://creativecommons.org/licenses/by/4.0/.

Extensions of this approach enabled the genetically programmed synthesis of noncanonical macrocycles. The first such macrocycles was formed entirely from ncAAs cyclized through a cysteine (Figure 31c).^77^ Subsequent work has demonstrated the synthesis of 37 genetically programmed noncanonical peptide or depsipeptide macrocycles containing diverse ncAAs or hydroxy acids at programmed sites (Figure 32).^132^ In future work it may be possible to directly synthesize libraries of genetically encoded noncanonical macrocycles in cells that enable direct selection for molecules that perform a specific function.

The work on genetically programmed noncanonical polymer synthesis exemplifies how sense codon reassignment can be used to make entirely noncanonical polymers and macrocycles composed of distinct building blocks; in these molecules the order of chemical building blocks is defined by the sequence of codons in a synthetic gene, and the identity of the chemical building blocks is defined by the monomers directed to the ribosome by the mutually orthogonal synthetases and tRNAs that read the distinct codons. In future work, strategies for encoding noncanonical polymers will be combined with strategies for expanding the chemical scope of cellular translation to ncMs (Section 7). This should enable the encoded cellular synthesis of an even wider range of noncanonical polymers.

## 12 Conclusion and Future Challenges

In the last two decades PylRS/tRNA^Pyl^ pairs have emerged as the most widely used orthogonal aaRS/tRNA pairs for genetic code expansion.^2^ PylRS/tRNA^Pyl^ pairs have enabled the incorporation of ncAAs with a range of chemical structures,^6^ the incorporation of ncAAs in all domains of life,^3^ and the incorporation of ncAAs in response to diverse codons.^2^ These advances build on several properties that are unique to the pair: the unique ncAAs accommodated by PylRS and the malleability of its active site,^45,58,70,82,93^ the unique sequence and structure of PylRS and tRNA^Pyl^ with respect to other aaRS/tRNA pairs,^20,57,60^ and the absence of anticodon recognition in all PylRS/tRNA^Pyl^ pairs where this has been investigated.^59,75^

Engineered PylRS/tRNA pairs, in combination with creative chemistry, have enabled new approaches for studying, and rapidly manipulating biological systems, and these approaches have led to numerous new insights.^1−6^ We anticipate that PylRS/tRNA^Pyl^ pairs will continue to be the systems of choice for adding ncAAs with new and useful functionalities to the genetic code.

The surprising discovery that different PylRS/tRNA^Pyl^ pairs can be mutually orthogonal in their acylation specificity (as well as orthgonal to endogenous pairs for canonical amino acid) has led to many new directions.^38^ Approaches for mining the astonishing diversity of PylRS/tRNA^Pyl^ pairs, in combination with engineering approaches has led to sets of up to five quintuply orthogonal PylRS/tRNA^Pyl^ pairs.^35,36,38−40^ Understanding the sequence variations, and molecular mechanisms, underpinning the orthogonality of PylRS/tRNA^Pyl^ pairs from distinct classes may provide further insight into how to engineer the next generation of aaRS/tRNA pairs for genetic code expansion. As more sequences become available it will be interesting to see whether even more sets of mutually orthogonal pairs can be discovered, or engineered *de novo*.

The combination of mutually orthogonal PylRS/tRNA^Pyl^ pairs that recognize distinct ncAAs and are directed to distinct codons has enabled the incorporation of several distinct ncAAs into proteins in response to diverse codons, including quadruplet codons, codons containing synthetic bases, and sense codons in organisms with compressed genetic codes.^2^ This has facilitated the labeling of proteins with multiple distinct functionalities, including: pairs of fluorophores for FRET studies, and cross-linkers and PTM analogs to trap PTM specific interactions.^410^ In future work, it will be interesting to explore the generation of emergent properties from combinations of ncAAs. This might include the generation of new enzyme active sites, where catalysis results from combinations of ncAAs, and this direction holds promise for generating new to nature catalytic function.

The combination of mutually orthogonal PylRS/tRNA^Pyl^ pairs, that recognize distinct ncAAs and are directed to distinct sense codons, with a cell run on a synthetic genome that uses a compressed genetic code, has enabled the encoded synthesis of polymers and macrocycles composed entirely of ncAA.^77,132,525^ In future, work it will be interesting to expand the length of genetically encoded polymers that can be synthesized in cells to enable the synthesis of ncAA based foldamers. This may require strategies for generating more efficient synthetase/tRNA pairs, and strategies for continuous evolution may be particularly valuable for addressing this challenge.^180−185^

Recent work has developed direct selections for PylRS variants that acylate their cognate tRNAs with ncMs (including β-linked monomers, and α-,α-disubstituted monomers) beyond α-*L*-amino acids,^19^ and this has enabled an expansion of the classes of monomers that can be added to the genetic code of cells. It will now be possible to use translation-based selections, including double-sieve selections, to optimize other components of the translational machinery–including tRNAs, E-Tu and other translation factors, and ribosomes–to further increase the efficiency and specificity of ncM incorporation. Future work will also expand the range of ncMs, and the classes of ncMs that can be genetically encoded in cells.

The cellular genetic encoding of ncMs into proteins may provide new ways to control the secondary and tertiary structure of proteins and to generate protease resistant proteins;^531,532^ this may enable diverse applications, including the scalable biosynthesis of modified protein therapeutics and the discovery of new therapeutics. The genetic encoding of ncMs may also enable the discovery and optimization of macrocycles with improved cellular uptake and stability.^77,132,210^

By combining strategies for encoding ncMs, with the development of mutually orthogonal pairs, and strategies for generating free codons it may be possible to genetically encode the cellular synthesis of polymers composed entirely of ncMs, with complete control over sequence and composition. This may enable the directed evolution of polymers with diverse backbone structures to generate foldamers^533^ that can replace and expand the functions carried out by natural proteins.

## References

[ref1] Liu C. C.; Schultz P. G. Adding New Chemistries to the Genetic Code. Annu. Rev. Biochem. 2010, 79, 413–444. 10.1146/annurev.biochem.052308.105824. 20307192

[ref2] de la Torre D.; Chin J. W. Reprogramming the Genetic Code. Nat. Rev. Genet. 2021, 22, 169–184. 10.1038/s41576-020-00307-7. 33318706

[ref3] Chin J. W. Expanding and Reprogramming the Genetic Code of Cells and Animals. Annu. Rev. Biochem. 2014, 83, 379–408. 10.1146/annurev-biochem-060713-035737. 24555827

[ref4] Chin J. W. Expanding and Reprogramming the Genetic Code. Nature 2017, 550, 53–60. 10.1038/nature24031. 28980641

[ref5] Young D. D.; Schultz P. G. Playing with the Molecules of Life. ACS Chem. Biol. 2018, 13, 854–870. 10.1021/acschembio.7b00974. 29345901 PMC6061972

[ref6] Davis L.; Chin J. W. Designer Proteins: Applications of Genetic Code Expansion in Cell Biology. Nat. Rev. Mol. Cell. Biol. 2012, 13, 168–182. 10.1038/nrm3286. 22334143

[ref7] Yanagisawa T.; Seki E.; Tanabe H.; Fujii Y.; Sakamoto K.; Yokoyama S. Crystal Structure of Pyrrolysyl-Trna Synthetase from a Methanogenic Archaeon Iso4-G1 and Its Structure-Based Engineering for Highly-Productive Cell-Free Genetic Code Expansion with Non-Canonical Amino Acids. Int. J. Mol. Sci. 2023, 24, 6256 10.3390/ijms24076256. 37047230 PMC10094482

[ref8] Ranji Charna A.; Des Soye B. J.; Ntai I.; Kelleher N. L.; Jewett M. C. An Efficient Cell-Free Protein Synthesis Platform for Producing Proteins with Pyrrolysine-Based Noncanonical Amino Acids. Biotechnol. J. 2022, 17, e2200096 10.1002/biot.202200096. 35569121 PMC9452482

[ref9] Seki E.; Yanagisawa T.; Kuratani M.; Sakamoto K.; Yokoyama S. Fully Productive Cell-Free Genetic Code Expansion by Structure-Based Engineering of Methanomethylophilus Alvus Pyrrolysyl-Trna Synthetase. ACS Synth. Biol. 2020, 9, 718–732. 10.1021/acssynbio.9b00288. 32182048

[ref10] Gerrits M.; Budisa N.; Merk H. Site-Specific Chemoselective Pyrrolysine Analogues Incorporation Using the Cell-Free Protein Synthesis System. ACS Synth. Biol. 2019, 8, 381–390. 10.1021/acssynbio.8b00421. 30589532

[ref11] Chemla Y.; Ozer E.; Shaferman M.; Zaad B.; Dandela R.; Alfonta L. Simplified Methodology for a Modular and Genetically Expanded Protein Synthesis in Cell-Free Systems. Synth. Syst. Biotechnol. 2019, 4, 189–196. 10.1016/j.synbio.2019.10.002. 31890924 PMC6926333

[ref12] Chemla Y.; Ozer E.; Schlesinger O.; Noireaux V.; Alfonta L. Genetically Expanded Cell-Free Protein Synthesis Using Endogenous Pyrrolysyl Orthogonal Translation System. Biotechnol. Bioeng. 2015, 112, 1663–1672. 10.1002/bit.25587. 25753985

[ref13] Burke S. A.; Lo S. L.; Krzycki J. A. Clustered Genes Encoding the Methyltransferases of Methanogenesis from Monomethylamine. J. Bacteriol. 1998, 180, 3432–3440. 10.1128/JB.180.13.3432-3440.1998. 9642198 PMC107300

[ref14] Paul L.; Ferguson D. J. Jr; Krzycki J. A. The Trimethylamine Methyltransferase Gene and Multiple Dimethylamine Methyltransferase Genes of Methanosarcina Barkeri Contain in-Frame and Read-through Amber Codons. J. Bacteriol. 2000, 182, 2520–2529. 10.1128/JB.182.9.2520-2529.2000. 10762254 PMC111316

[ref15] James C. M.; Ferguson T. K.; Leykam J. F.; Krzycki J. A. The Amber Codon in the Gene Encoding the Monomethylamine Methyltransferase Isolated from Methanosarcina Barkeri Is Translated as a Sense Codon. J. Biol. Chem. 2001, 276, 34252–34258. 10.1074/jbc.M102929200. 11435424

[ref16] Hao B.; Gong W.; Ferguson T. K.; James C. M.; Krzycki J. A.; Chan M. K. A New Uag-Encoded Residue in the Structure of a Methanogen Methyltransferase. Science 2002, 296, 1462–1466. 10.1126/science.1069556. 12029132

[ref17] Soares J. A.; Zhang L.; Pitsch R. L.; Kleinholz N. M.; Jones R. B.; Wolff J. J.; Amster J.; Green-Church K. B.; Krzycki J. A. The Residue Mass of L-Pyrrolysine in Three Distinct Methylamine Methyltransferases. J. Biol. Chem. 2005, 280, 36962–36969. 10.1074/jbc.M506402200. 16096277

[ref18] Li J.; Kang P. T.; Jiang R.; Lee J. Y.; Soares J. A.; Krzycki J. A.; Chan M. K. Insights into Pyrrolysine Function from Structures of a Trimethylamine Methyltransferase and Its Corrinoid Protein Complex. Commun. Biol. 2023, 6, 54 10.1038/s42003-022-04397-3. 36646841 PMC9842639

[ref19] Dunkelmann D. L.; Piedrafita C.; Dickson A.; Liu K. C.; Elliott T. S.; Fiedler M.; Bellini D.; Zhou A.; Cervettini D.; Chin J. W. Adding Alpha,Alpha-Disubstituted and Beta-Linked Monomers to the Genetic Code of an Organism. Nature 2024, 625, 603–610. 10.1038/s41586-023-06897-6. 38200312 PMC10794150

[ref20] Srinivasan G.; James C. M.; Krzycki J. A. Pyrrolysine Encoded by Uag in Archaea: Charging of a Uag-Decoding Specialized Trna. Science 2002, 296, 1459–1462. 10.1126/science.1069588. 12029131

[ref21] Mahapatra A.; Patel A.; Soares J. A.; Larue R. C.; Zhang J. K.; Metcalf W. W.; Krzycki J. A. Characterization of a Methanosarcina Acetivorans Mutant Unable to Translate Uag as Pyrrolysine. Mol. Microbiol. 2006, 59, 56–66. 10.1111/j.1365-2958.2005.04927.x. 16359318

[ref22] Blight S. K.; Larue R. C.; Mahapatra A.; Longstaff D. G.; Chang E.; Zhao G.; Kang P. T.; Green-Church K. B.; Chan M. K.; Krzycki J. A. Direct Charging of Trna(Cua) with Pyrrolysine in Vitro and in Vivo. Nature 2004, 431, 333–335. 10.1038/nature02895. 15329732

[ref23] Longstaff D. G.; Larue R. C.; Faust J. E.; Mahapatra A.; Zhang L.; Green-Church K. B.; Krzycki J. A. A Natural Genetic Code Expansion Cassette Enables Transmissible Biosynthesis and Genetic Encoding of Pyrrolysine. Proc. Natl. Acad. Sci. U. S. A. 2007, 104, 1021–1026. 10.1073/pnas.0610294104. 17204561 PMC1783357

[ref24] Gaston M. A.; Zhang L.; Green-Church K. B.; Krzycki J. A. The Complete Biosynthesis of the Genetically Encoded Amino Acid Pyrrolysine from Lysine. Nature 2011, 471, 647–650. 10.1038/nature09918. 21455182 PMC3070376

[ref25] Quitterer F.; List A.; Eisenreich W.; Bacher A.; Groll M. Crystal Structure of Methylornithine Synthase (Pylb): Insights into the Pyrrolysine Biosynthesis. Angew. Chem., Int. Ed. 2012, 51, 1339–1342. 10.1002/anie.201106765. 22095926

[ref26] Namy O.; Zhou Y.; Gundllapalli S.; Polycarpo C. R.; Denise A.; Rousset J. P.; Soll D.; Ambrogelly A. Adding Pyrrolysine to the Escherichia Coli Genetic Code. FEBS Lett. 2007, 581, 5282–5288. 10.1016/j.febslet.2007.10.022. 17967457

[ref27] Quitterer F.; List A.; Beck P.; Bacher A.; Groll M. Biosynthesis of the 22nd Genetically Encoded Amino Acid Pyrrolysine: Structure and Reaction Mechanism of Pylc at 1.5a Resolution. J. Mol. Biol. 2012, 424, 270–282. 10.1016/j.jmb.2012.09.007. 22985965

[ref28] Quitterer F.; Beck P.; Bacher A.; Groll M. Structure and Reaction Mechanism of Pyrrolysine Synthase (Pyld). Angew. Chem., Int. Ed. 2013, 52, 7033–7037. 10.1002/anie.201301164. 23720358

[ref29] Cellitti S. E.; Ou W.; Chiu H. P.; Grunewald J.; Jones D. H.; Hao X.; Fan Q.; Quinn L. L.; Ng K.; Anfora A. T.; et al. D-Ornithine Coopts Pyrrolysine Biosynthesis to Make and Insert Pyrroline-Carboxy-Lysine. Nat. Chem. Biol. 2011, 7, 528–530. 10.1038/nchembio.586. 21525873

[ref30] Prat L.; Heinemann I. U.; Aerni H. R.; Rinehart J.; O’Donoghue P.; Soll D. Carbon Source-Dependent Expansion of the Genetic Code in Bacteria. Proc. Natl. Acad. Sci. U. S. A. 2012, 109, 21070–21075. 10.1073/pnas.1218613110. 23185002 PMC3529041

[ref31] Gaston M. A.; Jiang R.; Krzycki J. A. Functional Context, Biosynthesis, and Genetic Encoding of Pyrrolysine. Curr. Opin. Microbiol. 2011, 14, 342–349. 10.1016/j.mib.2011.04.001. 21550296 PMC3119745

[ref32] Zhang Y.; Baranov P. V.; Atkins J. F.; Gladyshev V. N. Pyrrolysine and Selenocysteine Use Dissimilar Decoding Strategies. J. Biol. Chem. 2005, 280, 20740–20751. 10.1074/jbc.M501458200. 15788401

[ref33] Heinemann I. U.; O’Donoghue P.; Madinger C.; Benner J.; Randau L.; Noren C. J.; Soll D. The Appearance of Pyrrolysine in Trnahis Guanylyltransferase by Neutral Evolution. Proc. Natl. Acad. Sci. U. S. A. 2009, 106, 21103–21108. 10.1073/pnas.0912072106. 19965368 PMC2795538

[ref34] Guan Y.; Haroon M. F.; Alam I.; Ferry J. G.; Stingl U. Single-Cell Genomics Reveals Pyrrolysine-Encoding Potential in Members of Uncultivated Archaeal Candidate Division Msbl1. Environ. Microbiol. Rep. 2017, 9, 404–410. 10.1111/1758-2229.12545. 28493460

[ref35] Borrel G.; Gaci N.; Peyret P.; O’Toole P. W.; Gribaldo S.; Brugere J. F. Unique Characteristics of the Pyrrolysine System in the 7th Order of Methanogens: Implications for the Evolution of a Genetic Code Expansion Cassette. Archaea 2014, 2014, 374146 10.1155/2014/374146. 24669202 PMC3941956

[ref36] Borrel G.; Parisot N.; Harris H. M.; Peyretaillade E.; Gaci N.; Tottey W.; Bardot O.; Raymann K.; Gribaldo S.; Peyret P.; et al. Comparative Genomics Highlights the Unique Biology of Methanomassiliicoccales, a Thermoplasmatales-Related Seventh Order of Methanogenic Archaea That Encodes Pyrrolysine. BMC Genomics 2014, 15, 679 10.1186/1471-2164-15-679. 25124552 PMC4153887

[ref37] Brugere J. F.; Atkins J. F.; O’Toole P. W.; Borrel G. Pyrrolysine in Archaea: A 22nd Amino Acid Encoded through a Genetic Code Expansion. Emerg. Top. Life Sci. 2018, 2, 607–618. 10.1042/ETLS20180094. 33525836

[ref38] Willis J. C. W.; Chin J. W. Mutually Orthogonal Pyrrolysyl-Trna Synthetase/Trna Pairs. Nat. Chem. 2018, 10, 831–837. 10.1038/s41557-018-0052-5. 29807989 PMC6055992

[ref39] Dunkelmann D. L.; Willis J. C. W.; Beattie A. T.; Chin J. W. Engineered Triply Orthogonal Pyrrolysyl-Trna Synthetase/Trna Pairs Enable the Genetic Encoding of Three Distinct Non-Canonical Amino Acids. Nat. Chem. 2020, 12, 535–544. 10.1038/s41557-020-0472-x. 32472101 PMC7116526

[ref40] Beattie A. T.; Dunkelmann D. L.; Chin J. W. Quintuply Orthogonal Pyrrolysyl-Trna Synthetase/Trna(Pyl) Pairs. Nat. Chem. 2023, 15, 948–959. 10.1038/s41557-023-01232-y. 37322102 PMC7615293

[ref41] Fadhlaoui K.; Arnal M.-E.; Martineau M.; Camponova P.; Ollivier B.; O’Toole P. W.; Brugère J.-F. Archaea, Specific Genetic Traits, and Development of Improved Bacterial Live Biotherapeutic Products: Another Face of Next-Generation Probiotics. Appl. Microbiol. Biotechnol. 2020, 104, 4705–4716. 10.1007/s00253-020-10599-8. 32281023

[ref42] Guo L. T.; Amikura K.; Jiang H. K.; Mukai T.; Fu X.; Wang Y. S.; O’Donoghue P.; Soll D.; Tharp J. M. Ancestral Archaea Expanded the Genetic Code with Pyrrolysine. J. Biol. Chem. 2022, 298, 102521 10.1016/j.jbc.2022.102521. 36152750 PMC9630628

[ref43] Sprinzl M.; Horn C.; Brown M.; Ioudovitch A.; Steinberg S. Compilation of Trna Sequences and Sequences of Trna Genes. Nucleic Acids Res. 1998, 26, 148–153. 10.1093/nar/26.1.148. 9399820 PMC147216

[ref44] Yanagisawa T.; Ishii R.; Fukunaga R.; Nureki O.; Yokoyama S. Crystallization and Preliminary X-Ray Crystallographic Analysis of the Catalytic Domain of Pyrrolysyl-Trna Synthetase from the Methanogenic Archaeon Methanosarcina Mazei. Acta Crystallogr. F-Struct. Biol. Commun. 2006, 62, 1031–1033. 10.1107/S1744309106036700. PMC2225182 17012805

[ref45] Kavran J. M.; Gundllapalli S.; O’Donoghue P.; Englert M.; Soll D.; Steitz T. A. Structure of Pyrrolysyl-Trna Synthetase, an Archaeal Enzyme for Genetic Code Innovation. Proc. Natl. Acad. Sci. U. S. A. 2007, 104, 11268–11273. 10.1073/pnas.0704769104. 17592110 PMC2040888

[ref46] Cusack S.; Berthet-Colominas C.; Härtlein M.; Nassar N.; Leberman R. A Second Class of Synthetase Structure Revealed by X-Ray Analysis of Escherichia Coli Seryl-Trna Synthetase at 2.5 Å. Nature 1990, 347, 249–255. 10.1038/347249a0. 2205803

[ref47] Eriani G.; Delarue M.; Poch O.; Gangloff J.; Moras D. Partition of Trna Synthetases into Two Classes Based on Mutually Exclusive Sets of Sequence Motifs. Nature 1990, 347, 203–206. 10.1038/347203a0. 2203971

[ref48] Lee M. M.; Jiang R.; Jain R.; Larue R. C.; Krzycki J.; Chan M. K. Structure of Desulfitobacterium Hafniense Pylsc, a Pyrrolysyl-Trna Synthetase. Biochem. Biophys. Res. Commun. 2008, 374, 470–474. 10.1016/j.bbrc.2008.07.074. 18656445 PMC2572580

[ref49] Gottfried-Lee I.; Perona J. J.; Karplus P. A.; Mehl R. A.; Cooley R. B. Structures of Methanomethylophilus Alvus Pyrrolysine Trna-Synthetases Support the Need for De Novo Selections When Altering the Substrate Specificity. ACS Chem. Biol. 2022, 17, 3470–3477. 10.1021/acschembio.2c00640. 36395426 PMC9833844

[ref50] Vatansever E. C.; Yang K. S.; Geng Z. Z.; Qiao Y.; Li P.; Xu S.; Liu W. R. A Designed, Highly Efficient Pyrrolysyl-Trna Synthetase Mutant Binds O-Chlorophenylalanine Using Two Halogen Bonds. J. Mol. Biol. 2022, 434, 167534 10.1016/j.jmb.2022.167534. 35278475 PMC9018553

[ref51] Yanagisawa T.; Kuratani M.; Seki E.; Hino N.; Sakamoto K.; Yokoyama S. Structural Basis for Genetic-Code Expansion with Bulky Lysine Derivatives by an Engineered Pyrrolysyl-Trna Synthetase. Cell Chem. Biol. 2019, 26, 936–949. 10.1016/j.chembiol.2019.03.008. 31031143

[ref52] Yanagisawa T.; Sumida T.; Ishii R.; Yokoyama S. A Novel Crystal Form of Pyrrolysyl-Trna Synthetase Reveals the Pre- and Post-Aminoacyl-Trna Synthesis Conformational States of the Adenylate and Aminoacyl Moieties and an Asparagine Residue in the Catalytic Site. Acta Crystallogr. Sect. D-Biol. Crystallogr. 2013, 69, 5–15. 10.1107/S0907444912039881. 23275158

[ref53] Schneider S.; Gattner M. J.; Vrabel M.; Flugel V.; Lopez-Carrillo V.; Prill S.; Carell T. Structural Insights into Incorporation of Norbornene Amino Acids for Click Modification of Proteins. ChemBioChem. 2013, 14, 2114–2118. 10.1002/cbic.201300435. 24027216

[ref54] Flugel V.; Vrabel M.; Schneider S. Structural Basis for the Site-Specific Incorporation of Lysine Derivatives into Proteins. PLoS One 2014, 9, e96198 10.1371/journal.pone.0096198. 24760130 PMC3997565

[ref55] Schmidt M. J.; Weber A.; Pott M.; Welte W.; Summerer D. Structural Basis of Furan-Amino Acid Recognition by a Polyspecific Aminoacyl-Trna-Synthetase and Its Genetic Encoding in Human Cells. ChemBioChem. 2014, 15, 1755–1760. 10.1002/cbic.201402006. 24737732

[ref56] Takimoto J. K.; Dellas N.; Noel J. P.; Wang L. Stereochemical Basis for Engineered Pyrrolysyl-Trna Synthetase and the Efficient in Vivo Incorporation of Structurally Divergent Non-Native Amino Acids. ACS Chem. Biol. 2011, 6, 733–743. 10.1021/cb200057a. 21545173 PMC3137230

[ref57] Nozawa K.; O’Donoghue P.; Gundllapalli S.; Araiso Y.; Ishitani R.; Umehara T.; Soll D.; Nureki O. Pyrrolysyl-Trna Synthetase-Trna(Pyl) Structure Reveals the Molecular Basis of Orthogonality. Nature 2009, 457, 1163–1167. 10.1038/nature07611. 19118381 PMC2648862

[ref58] Yanagisawa T.; Ishii R.; Fukunaga R.; Kobayashi T.; Sakamoto K.; Yokoyama S. Multistep Engineering of Pyrrolysyl-Trna Synthetase to Genetically Encode N(Epsilon)-(O-Azidobenzyloxycarbonyl) Lysine for Site-Specific Protein Modification. Chem. Biol. 2008, 15, 1187–1197. 10.1016/j.chembiol.2008.10.004. 19022179

[ref59] Yanagisawa T.; Ishii R.; Fukunaga R.; Kobayashi T.; Sakamoto K.; Yokoyama S. Crystallographic Studies on Multiple Conformational States of Active-Site Loops in Pyrrolysyl-Trna Synthetase. J. Mol. Biol. 2008, 378, 634–652. 10.1016/j.jmb.2008.02.045. 18387634

[ref60] Suzuki T.; Miller C.; Guo L. T.; Ho J. M. L.; Bryson D. I.; Wang Y. S.; Liu D. R.; Soll D. Crystal Structures Reveal an Elusive Functional Domain of Pyrrolysyl-Trna Synthetase. Nat. Chem. Biol. 2017, 13, 1261–1266. 10.1038/nchembio.2497. 29035363 PMC5698177

[ref61] Reshetnikova L.; Moor N.; Lavrik O.; Vassylyev D. G. Crystal Structures of Phenylalanyl-Trna Synthetase Complexed with Phenylalanine and a Phenylalanyl-Adenylate Analogue. J. Mol. Biol. 1999, 287, 555–568. 10.1006/jmbi.1999.2617. 10092459

[ref62] Wan W.; Tharp J. M.; Liu W. R. Pyrrolysyl-Trna Synthetase: An Ordinary Enzyme but an Outstanding Genetic Code Expansion Tool. Biochim. Biophys. Acta. Proteins Proteom. 2014, 1844, 1059–1070. 10.1016/j.bbapap.2014.03.002. PMC4016821 24631543

[ref63] Jiang R.; Krzycki J. A. Pylsn and the Homologous N-Terminal Domain of Pyrrolysyl-Trna Synthetase Bind the Trna That Is Essential for the Genetic Encoding of Pyrrolysine. J. Biol. Chem. 2012, 287, 32738–32746. 10.1074/jbc.M112.396754. 22851181 PMC3463324

[ref64] Herring S.; Ambrogelly A.; Gundllapalli S.; O’Donoghue P.; Polycarpo C. R.; Soll D. The Amino-Terminal Domain of Pyrrolysyl-Trna Synthetase Is Dispensable in Vitro but Required for in Vivo Activity. FEBS Lett. 2007, 581, 3197–3203. 10.1016/j.febslet.2007.06.004. 17582401 PMC2074874

[ref65] Katayama H.; Nozawa K.; Nureki O.; Nakahara Y.; Hojo H. Pyrrolysine Analogs as Substrates for Bacterial Pyrrolysyl-Trna Synthetase in Vitro and in Vivo. Biosci. Biotechnol. Biochem. 2012, 76, 205–208. 10.1271/bbb.110653. 22232266

[ref66] Theobald-Dietrich A.; Frugier M.; Giege R.; Rudinger-Thirion J. Atypical Archaeal Trna Pyrrolysine Transcript Behaves Towards Ef-Tu as a Typical Elongator Trna. Nucleic Acids Res. 2004, 32, 1091–1096. 10.1093/nar/gkh266. 14872064 PMC373401

[ref67] Herring S.; Ambrogelly A.; Polycarpo C. R.; Soll D. Recognition of Pyrrolysine Trna by the Desulfitobacterium Hafniense Pyrrolysyl-Trna Synthetase. Nucleic Acids Res. 2007, 35, 1270–1278. 10.1093/nar/gkl1151. 17267409 PMC1851642

[ref68] Polycarpo C.; Ambrogelly A.; Berube A.; Winbush S. M.; McCloskey J. A.; Crain P. F.; Wood J. L.; Soll D. An Aminoacyl-Trna Synthetase That Specifically Activates Pyrrolysine. Proc. Natl. Acad. Sci. U. S. A. 2004, 101, 12450–12454. 10.1073/pnas.0405362101. 15314242 PMC515082

[ref69] Ibba M.; Soll D. Aminoacyl-Trna Synthesis. Annu. Rev. Biochem. 2000, 69, 617–650. 10.1146/annurev.biochem.69.1.617. 10966471

[ref70] Polycarpo C. R.; Herring S.; Berube A.; Wood J. L.; Soll D.; Ambrogelly A. Pyrrolysine Analogues as Substrates for Pyrrolysyl-Trna Synthetase. FEBS Lett. 2006, 580, 6695–6700. 10.1016/j.febslet.2006.11.028. 17126325 PMC1817836

[ref71] Li W. T.; Mahapatra A.; Longstaff D. G.; Bechtel J.; Zhao G.; Kang P. T.; Chan M. K.; Krzycki J. A. Specificity of Pyrrolysyl-Trna Synthetase for Pyrrolysine and Pyrrolysine Analogs. J. Mol. Biol. 2009, 385, 1156–1164. 10.1016/j.jmb.2008.11.032. 19063902

[ref72] Hao B.; Zhao G.; Kang P. T.; Soares J. A.; Ferguson T. K.; Gallucci J.; Krzycki J. A.; Chan M. K. Reactivity and Chemical Synthesis of L-Pyrrolysine- the 22(Nd) Genetically Encoded Amino Acid. Chem. Biol. 2004, 11, 1317–1324. 10.1016/j.chembiol.2004.07.011. 15380192

[ref73] Wong M. L.; Guzei I. A.; Kiessling L. L. An Asymmetric Synthesis of L-Pyrrolysine. Org. Lett. 2012, 14, 1378–1381. 10.1021/ol300045c. 22394273 PMC3326344

[ref74] Han M. Y.; Wang H. Z.; An W. K.; Jia J. Y.; Ma B. C.; Zhang Y.; Wang W. A Concise Synthesis of L-Pyrrolysine. Chem. Eur. J. 2013, 19, 8078–8081. 10.1002/chem.201300403. 23649505

[ref75] Ambrogelly A.; Gundllapalli S.; Herring S.; Polycarpo C.; Frauer C.; Soll D. Pyrrolysine Is Not Hardwired for Cotranslational Insertion at Uag Codons. Proc. Natl. Acad. Sci. U. S. A. 2007, 104, 3141–3146. 10.1073/pnas.0611634104. 17360621 PMC1805618

[ref76] Wang K.; Sachdeva A.; Cox D. J.; Wilf N. M.; Lang K.; Wallace S.; Mehl R. A.; Chin J. W. Optimized Orthogonal Translation of Unnatural Amino Acids Enables Spontaneous Protein Double-Labelling and Fret. Nat. Chem. 2014, 6, 393–403. 10.1038/nchem.1919. 24755590 PMC4430801

[ref77] Robertson W. E.; Funke L. F. H.; de la Torre D.; Fredens J.; Elliott T. S.; Spinck M.; Christova Y.; Cervettini D.; Boge F. L.; Liu K. C.; et al. Sense Codon Reassignment Enables Viral Resistance and Encoded Polymer Synthesis. Science 2021, 372, 1057–1062. 10.1126/science.abg3029. 34083482 PMC7611380

[ref78] Zhang Y.; Ptacin J. L.; Fischer E. C.; Aerni H. R.; Caffaro C. E.; San Jose K.; Feldman A. W.; Turner C. R.; Romesberg F. E. A Semi-Synthetic Organism That Stores and Retrieves Increased Genetic Information. Nature 2017, 551, 644–647. 10.1038/nature24659. 29189780 PMC5796663

[ref79] Fischer E. C.; Hashimoto K.; Zhang Y.; Feldman A. W.; Dien V. T.; Karadeema R. J.; Adhikary R.; Ledbetter M. P.; Krishnamurthy R.; Romesberg F. E. New Codons for Efficient Production of Unnatural Proteins in a Semisynthetic Organism. Nat. Chem. Biol. 2020, 16, 570–576. 10.1038/s41589-020-0507-z. 32251411 PMC7263176

[ref80] Niu W.; Schultz P. G.; Guo J. An Expanded Genetic Code in Mammalian Cells with a Functional Quadruplet Codon. ACS Chem. Biol. 2013, 8, 1640–1645. 10.1021/cb4001662. 23662731 PMC4501474

[ref81] Konevega A. L.; Soboleva N. G.; Makhno V. I.; Semenkov Y. P.; Wintermeyer W.; Rodnina M. V.; Katunin V. I. Purine Bases at Position 37 of Trna Stabilize Codon-Anticodon Interaction in the Ribosomal a Site by Stacking and Mg2+-Dependent Interactions. RNA 2004, 10, 90–101. 10.1261/rna.5142404. 14681588 PMC1370521

[ref82] Neumann H.; Peak-Chew S. Y.; Chin J. W. Genetically Encoding Nε-Acetyllysine in Recombinant Proteins. Nat. Chem. Biol. 2008, 4, 232–234. 10.1038/nchembio.73. 18278036

[ref83] Cervettini D.; Tang S.; Fried S. D.; Willis J. C. W.; Funke L. F. H.; Colwell L. J.; Chin J. W. Rapid Discovery and Evolution of Orthogonal Aminoacyl-Trna Synthetase-Trna Pairs. Nat. Biotechnol. 2020, 38, 989–999. 10.1038/s41587-020-0479-2. 32284585 PMC7116527

[ref84] Wang L.; Brock A.; Herberich B.; Schultz P. G. Expanding the Genetic Code of Escherichia Coli. Science 2001, 292, 498–500. 10.1126/science.1060077. 11313494

[ref85] Rogerson D. T.; Sachdeva A.; Wang K.; Haq T.; Kazlauskaite A.; Hancock S. M.; Huguenin-Dezot N.; Muqit M. M.; Fry A. M.; Bayliss R.; et al. Efficient Genetic Encoding of Phosphoserine and Its Nonhydrolyzable Analog. Nat. Chem. Biol. 2015, 11, 496–503. 10.1038/nchembio.1823. 26030730 PMC4830402

[ref86] Park H. S.; Hohn M. J.; Umehara T.; Guo L. T.; Osborne E. M.; Benner J.; Noren C. J.; Rinehart J.; Soll D. Expanding the Genetic Code of Escherichia Coli with Phosphoserine. Science 2011, 333, 1151–1154. 10.1126/science.1207203. 21868676 PMC5547737

[ref87] Hughes R. A.; Ellington A. D. Rational Design of an Orthogonal Tryptophanyl Nonsense Suppressor Trna. Nucleic Acids Res. 2010, 38, 6813–6830. 10.1093/nar/gkq521. 20571084 PMC2965240

[ref88] Chatterjee A.; Sun S. B.; Furman J. L.; Xiao H.; Schultz P. G. A Versatile Platform for Single- and Multiple-Unnatural Amino Acid Mutagenesis in Escherichia Coli. Biochem. 2013, 52, 1828–1837. 10.1021/bi4000244. 23379331 PMC3855549

[ref89] Italia J. S.; Addy P. S.; Wrobel C. J.; Crawford L. A.; Lajoie M. J.; Zheng Y.; Chatterjee A. An Orthogonalized Platform for Genetic Code Expansion in Both Bacteria and Eukaryotes. Nat. Chem. Biol. 2017, 13, 446–450. 10.1038/nchembio.2312. 28192410

[ref90] Neumann H.; Slusarczyk A. L.; Chin J. W. De Novo Generation of Mutually Orthogonal Aminoacyl-Trna Synthetase/Trna Pairs. J. Am. Chem. Soc. 2010, 132, 2142–2144. 10.1021/ja9068722. 20121121

[ref91] Beranek V.; Willis J. C. W.; Chin J. W. An Evolved Methanomethylophilus Alvus Pyrrolysyl-Trna Synthetase/Trna Pair Is Highly Active and Orthogonal in Mammalian Cells. Biochem. 2019, 58, 387–390. 10.1021/acs.biochem.8b00808. 30260626 PMC6365905

[ref92] Chatterjee A.; Xiao H.; Schultz P. G. Evolution of Multiple, Mutually Orthogonal Prolyl-Trna Synthetase/Trna Pairs for Unnatural Amino Acid Mutagenesis in Escherichia Coli. Proc. Natl. Acad. Sci. U. S. A. 2012, 109, 14841–14846. 10.1073/pnas.1212454109. 22927411 PMC3443146

[ref93] Mukai T.; Kobayashi T.; Hino N.; Yanagisawa T.; Sakamoto K.; Yokoyama S. Adding L-Lysine Derivatives to the Genetic Code of Mammalian Cells with Engineered Pyrrolysyl-Trna Synthetases. Biochem. Biophys. Res. Commun. 2008, 371, 818–822. 10.1016/j.bbrc.2008.04.164. 18471995

[ref94] Bianco A.; Townsley F. M.; Greiss S.; Lang K.; Chin J. W. Expanding the Genetic Code of Drosophila Melanogaster. Nat. Chem. Biol. 2012, 8, 748–750. 10.1038/nchembio.1043. 22864544

[ref95] Greiss S.; Chin J. W. Expanding the Genetic Code of an Animal. J. Am. Chem. Soc. 2011, 133, 14196–14199. 10.1021/ja2054034. 21819153 PMC3168933

[ref96] Ernst R. J.; Krogager T. P.; Maywood E. S.; Zanchi R.; Beranek V.; Elliott T. S.; Barry N. P.; Hastings M. H.; Chin J. W. Genetic Code Expansion in the Mouse Brain. Nat. Chem. Biol. 2016, 12, 776–778. 10.1038/nchembio.2160. 27571478 PMC5215917

[ref97] Li F.; Zhang H.; Sun Y.; Pan Y.; Zhou J.; Wang J. Expanding the Genetic Code for Photoclick Chemistry in E. Coli, Mammalian Cells, and A. Thaliana. Angew. Chem., Int. Ed. 2013, 52, 9700–9704. 10.1002/anie.201303477. 23873613

[ref98] Davis L.; Radman I.; Goutou A.; Tynan A.; Baxter K.; Xi Z.; O’Shea J. M.; Chin J. W.; Greiss S. Precise Optical Control of Gene Expression in C Elegans Using Improved Genetic Code Expansion and Cre Recombinase. Elife 2021, 10, 10.7554/eLife.67075. PMC8448529 34350826

[ref99] Weaver J. B.; Boxer S. G. Genetic Code Expansion in Rhodobacter Sphaeroides to Incorporate Noncanonical Amino Acids into Photosynthetic Reaction Centers. ACS Synth. Biol. 2018, 7, 1618–1628. 10.1021/acssynbio.8b00100. 29763307

[ref100] Takahashi H.; Dohmae N.; Kim K. S.; Shimuta K.; Ohnishi M.; Yokoyama S.; Yanagisawa T. Genetic Incorporation of Non-Canonical Amino Acid Photocrosslinkers in Neisseria Meningitidis: New Method Provides Insights into the Physiological Function of the Function-Unknown Nmb1345 Protein. PLoS One 2020, 15, e0237883 10.1371/journal.pone.0237883. 32866169 PMC7458321

[ref101] Chemla Y.; Friedman M.; Heltberg M.; Bakhrat A.; Nagar E.; Schwarz R.; Jensen M. H.; Alfonta L. Expanding the Genetic Code of a Photoautotrophic Organism. Biochem. 2017, 56, 2161–2165. 10.1021/acs.biochem.7b00131. 28394580

[ref102] Zheng H.; Lin S.; Chen P. R. Genetically Encoded Protein Labeling and Crosslinking in Living Pseudomonas Aeruginosa. Bioorg. Med. Chem. 2020, 28, 115545 10.1016/j.bmc.2020.115545. 32503693

[ref103] Bartholomae M.; Baumann T.; Nickling J. H.; Peterhoff D.; Wagner R.; Budisa N.; Kuipers O. P. Expanding the Genetic Code of Lactococcus Lactis and Escherichia Coli to Incorporate Non-Canonical Amino Acids for Production of Modified Lantibiotics. Front. Microbiol. 2018, 9, 657 10.3389/fmicb.2018.00657. 29681891 PMC5897534

[ref104] Lopatniuk M.; Myronovskyi M.; Luzhetskyy A. Streptomyces Albus: A New Cell Factory for Non-Canonical Amino Acids Incorporation into Ribosomally Synthesized Natural Products. ACS Chem. Biol. 2017, 12, 2362–2370. 10.1021/acschembio.7b00359. 28758722

[ref105] Lin S.; Zhang Z.; Xu H.; Li L.; Chen S.; Li J.; Hao Z.; Chen P. R. Site-Specific Incorporation of Photo-Cross-Linker and Bioorthogonal Amino Acids into Enteric Bacterial Pathogens. J. Am. Chem. Soc. 2011, 133, 20581–20587. 10.1021/ja209008w. 22084898

[ref106] Elsasser S. J.; Ernst R. J.; Walker O. S.; Chin J. W. Genetic Code Expansion in Stable Cell Lines Enables Encoded Chromatin Modification. Nat. Methods 2016, 13, 158–164. 10.1038/nmeth.3701. 26727110 PMC4888942

[ref107] Liu J.; Hemphill J.; Samanta S.; Tsang M.; Deiters A. Genetic Code Expansion in Zebrafish Embryos and Its Application to Optical Control of Cell Signaling. J. Am. Chem. Soc. 2017, 139, 9100–9103. 10.1021/jacs.7b02145. 28657738 PMC6022368

[ref108] Han S.; Yang A.; Lee S.; Lee H.-W.; Park C. B.; Park H.-S. Expanding the Genetic Code of Mus Musculus. Nat. Commun. 2017, 8, 14568 10.1038/ncomms14568. 28220771 PMC5321798

[ref109] Chin J. W.; Martin A. B.; King D. S.; Wang L.; Schultz P. G. Addition of a Photocrosslinking Amino Acid to the Genetic Code of Escherichiacoli. Proc. Natl. Acad. Sci. U. S. A. 2002, 99, 11020–11024. 10.1073/pnas.172226299. 12154230 PMC123203

[ref110] Chin J. W.; Cropp T. A.; Anderson J. C.; Mukherji M.; Zhang Z.; Schultz P. G. An Expanded Eukaryotic Genetic Code. Science 2003, 301, 964–967. 10.1126/science.1084772. 12920298

[ref111] Owens A. E.; Grasso K. T.; Ziegler C. A.; Fasan R. Two-Tier Screening Platform for Directed Evolution of Aminoacyl-Trna Synthetases with Enhanced Stop Codon Suppression Efficiency. ChemBioChem. 2017, 18, 1109–1116. 10.1002/cbic.201700039. 28383180 PMC5586079

[ref112] Kuhn S. M.; Rubini M.; Fuhrmann M.; Theobald I.; Skerra A. Engineering of an Orthogonal Aminoacyl-Trna Synthetase for Efficient Incorporation of the Non-Natural Amino Acid O-Methyl-L-Tyrosine Using Fluorescence-Based Bacterial Cell Sorting. J. Mol. Biol. 2010, 404, 70–87. 10.1016/j.jmb.2010.09.001. 20837025

[ref113] Hohl A.; Karan R.; Akal A.; Renn D.; Liu X.; Ghorpade S.; Groll M.; Rueping M.; Eppinger J. Engineering a Polyspecific Pyrrolysyl-Trna Synthetase by a High Throughput Facs Screen. Sci. Rep. 2019, 9, 11971 10.1038/s41598-019-48357-0. 31427620 PMC6700097

[ref114] Lin A. E.; Lin Q. Rapid Identification of Functional Pyrrolysyl-Trna Synthetases Via Fluorescence-Activated Cell Sorting. Int. J. Mol. Sci. 2019, 20, 29 10.3390/ijms20010029. PMC6337664 30577609

[ref115] Santoro S. W.; Wang L.; Herberich B.; King D. S.; Schultz P. G. An Efficient System for the Evolution of Aminoacyl-Trna Synthetase Specificity. Nat. Biotechnol. 2002, 20, 1044–1048. 10.1038/nbt742. 12244330

[ref116] Stieglitz J. T.; Van Deventer J. A. High-Throughput Aminoacyl-Trna Synthetase Engineering for Genetic Code Expansion in Yeast. ACS Synth. Biol. 2022, 11, 2284–2299. 10.1021/acssynbio.1c00626. 35793554 PMC10065163

[ref117] Uyeda A.; Watanabe T.; Kato Y.; Watanabe H.; Yomo T.; Hohsaka T.; Matsuura T. Liposome-Based in Vitro Evolution of Aminoacyl-Trna Synthetase for Enhanced Pyrrolysine Derivative Incorporation. ChemBioChem. 2015, 16, 1797–1802. 10.1002/cbic.201500174. 26052693

[ref118] Umehara T.; Kim J.; Lee S.; Guo L. T.; Soll D.; Park H. S. N-Acetyl Lysyl-Trna Synthetases Evolved by a Ccdb-Based Selection Possess N-Acetyl Lysine Specificity in Vitro and in Vivo. FEBS Lett. 2012, 586, 729–733. 10.1016/j.febslet.2012.01.029. 22289181

[ref119] Amiram M.; Haimovich A. D.; Fan C.; Wang Y. S.; Aerni H. R.; Ntai I.; Moonan D. W.; Ma N. J.; Rovner A. J.; Hong S. H.; et al. Evolution of Translation Machinery in Recoded Bacteria Enables Multi-Site Incorporation of Nonstandard Amino Acids. Nat. Biotechnol. 2015, 33, 1272–1279. 10.1038/nbt.3372. 26571098 PMC4784704

[ref120] Guo L. T.; Wang Y. S.; Nakamura A.; Eiler D.; Kavran J. M.; Wong M.; Kiessling L. L.; Steitz T. A.; O’Donoghue P.; Soll D. Polyspecific Pyrrolysyl-Trna Synthetases from Directed Evolution. Proc. Natl. Acad. Sci. U. S. A. 2014, 111, 16724–16729. 10.1073/pnas.1419737111. 25385624 PMC4250173

[ref121] Cooley R. B.; Feldman J. L.; Driggers C. M.; Bundy T. A.; Stokes A. L.; Karplus P. A.; Mehl R. A. Structural Basis of Improved Second-Generation 3-Nitro-Tyrosine Trna Synthetases. Biochem. 2014, 53, 1916–1924. 10.1021/bi5001239. 24611875 PMC3985459

[ref122] Wang Y. S.; Russell W. K.; Wang Z.; Wan W.; Dodd L. E.; Pai P. J.; Russell D. H.; Liu W. R. The De Novo Engineering of Pyrrolysyl-Trna Synthetase for Genetic Incorporation of L-Phenylalanine and Its Derivatives. Mol. Biosyst. 2011, 7, 714–717. 10.1039/c0mb00217h. 21234492

[ref123] Wang Y. S.; Fang X.; Chen H. Y.; Wu B.; Wang Z. U.; Hilty C.; Liu W. R. Genetic Incorporation of Twelve Meta-Substituted Phenylalanine Derivatives Using a Single Pyrrolysyl-Trna Synthetase Mutant. ACS Chem. Biol. 2013, 8, 405–415. 10.1021/cb300512r. 23138887 PMC3574229

[ref124] Wang Y. S.; Fang X.; Wallace A. L.; Wu B.; Liu W. R. A Rationally Designed Pyrrolysyl-Trna Synthetase Mutant with a Broad Substrate Spectrum. J. Am. Chem. Soc. 2012, 134, 2950–2953. 10.1021/ja211972x. 22289053 PMC3288562

[ref125] Tharp J. M.; Wang Y. S.; Lee Y. J.; Yang Y.; Liu W. R. Genetic Incorporation of Seven Ortho-Substituted Phenylalanine Derivatives. ACS Chem. Biol. 2014, 9, 884–890. 10.1021/cb400917a. 24451054 PMC3997995

[ref126] Tuley A.; Wang Y. S.; Fang X.; Kurra Y.; Rezenom Y. H.; Liu W. R. The Genetic Incorporation of Thirteen Novel Non-Canonical Amino Acids. Chem. Commun. 2014, 50, 2673–2675. 10.1039/C3CC49068H. PMC4011397 24473369

[ref127] Lacey V. K.; Louie G. V.; Noel J. P.; Wang L. Expanding the Library and Substrate Diversity of the Pyrrolysyl-Trna Synthetase to Incorporate Unnatural Amino Acids Containing Conjugated Rings. ChemBioChem. 2013, 14, 2100–2105. 10.1002/cbic.201300400. 24019075 PMC3947478

[ref128] Tseng H. W.; Baumann T.; Sun H.; Wang Y. S.; Ignatova Z.; Budisa N. Expanding the Scope of Orthogonal Translation with Pyrrolysyl-Trna Synthetases Dedicated to Aromatic Amino Acids. Molecules 2020, 25, 4418 10.3390/molecules25194418. 32992991 PMC7582959

[ref129] Tseng H. W.; Moldenhauer M.; Friedrich T.; Maksimov E. G.; Budisa N. Probing the Spectral Signatures of Orange Carotenoid Protein by Orthogonal Translation with Aromatic Non-Canonical Amino Acids. Biochem. Biophys. Res. Commun. 2022, 607, 96–102. 10.1016/j.bbrc.2022.03.118. 35367834

[ref130] Wang Y. H.; Jian M. L.; Chen P. J.; Tsou J. C.; Truong L. P.; Wang Y. S. Ferritin Conjugates with Multiple Clickable Amino Acids Encoded by C-Terminal Engineered Pyrrolysyl-Trna Synthetase. Front. Chem. 2021, 9, 779976 10.3389/fchem.2021.779976. 34900939 PMC8655692

[ref131] Galles G. D.; Infield D. T.; Clark C. J.; Hemshorn M. L.; Manikandan S.; Fazan F.; Rasouli A.; Tajkhorshid E.; Galpin J. D.; Cooley R. B.; et al. Tuning Phenylalanine Fluorination to Assess Aromatic Contributions to Protein Function and Stability in Cells. Nat. Commun. 2023, 14, 59 10.1038/s41467-022-35761-w. 36599844 PMC9813137

[ref132] Spinck M.; Piedrafita C.; Robertson W. E.; Elliott T. S.; Cervettini D.; de la Torre D.; Chin J. W. Genetically Programmed Cell-Based Synthesis of Non-Natural Peptide and Depsipeptide Macrocycles. Nat. Chem. 2023, 15, 61–69. 10.1038/s41557-022-01082-0. 36550233 PMC9836938

[ref133] Pott M.; Tinzl M.; Hayashi T.; Ota Y.; Dunkelmann D.; Mittl P. R. E.; Hilvert D. Noncanonical Heme Ligands Steer Carbene Transfer Reactivity in an Artificial Metalloenzyme. Angew. Chem., Int. Ed. 2021, 60, 15063–15068. 10.1002/anie.202103437. 33880851

[ref134] Xiao H.; Peters F. B.; Yang P. Y.; Reed S.; Chittuluru J. R.; Schultz P. G. Genetic Incorporation of Histidine Derivatives Using an Engineered Pyrrolysyl-Trna Synthetase. ACS Chem. Biol. 2014, 9, 1092–1096. 10.1021/cb500032c. 24506189 PMC4033645

[ref135] Jang H. S.; Gu X.; Cooley R. B.; Porter J. J.; Henson R. L.; Willi T.; DiDonato J. A.; Hazen S. L.; Mehl R. A. Efficient Site-Specific Prokaryotic and Eukaryotic Incorporation of Halotyrosine Amino Acids into Proteins. ACS Chem. Biol. 2020, 15, 562–574. 10.1021/acschembio.9b01026. 31994864 PMC7207724

[ref136] Luo J.; Torres-Kolbus J.; Liu J.; Deiters A. Genetic Encoding of Photocaged Tyrosines with Improved Light-Activation Properties for the Optical Control of Protease Function. ChemBioChem. 2017, 18, 1442–1447. 10.1002/cbic.201700147. 28608946

[ref137] Hoppmann C.; Wong A.; Yang B.; Li S.; Hunter T.; Shokat K. M.; Wang L. Site-Specific Incorporation of Phosphotyrosine Using an Expanded Genetic Code. Nat. Chem. Biol. 2017, 13, 842–844. 10.1038/nchembio.2406. 28604697 PMC5577362

[ref138] Arbely E.; Torres-Kolbus J.; Deiters A.; Chin J. W. Photocontrol of Tyrosine Phosphorylation in Mammalian Cells Via Genetic Encoding of Photocaged Tyrosine. J. Am. Chem. Soc. 2012, 134, 11912–11915. 10.1021/ja3046958. 22758385

[ref139] Qianzhu H.; Abdelkader E. H.; Herath I. D.; Otting G.; Huber T. Site-Specific Incorporation of 7-Fluoro-L-Tryptophan into Proteins by Genetic Encoding to Monitor Ligand Binding by (19)F Nmr Spectroscopy. ACS Sens. 2022, 7, 44–49. 10.1021/acssensors.1c02467. 35005899

[ref140] Ortmayer M.; Hardy F. J.; Quesne M. G.; Fisher K.; Levy C.; Heyes D. J.; Catlow C. R. A.; De Visser S. P.; Rigby S. E. J.; Hay S.; et al. A Noncanonical Tryptophan Analogue Reveals an Active Site Hydrogen Bond Controlling Ferryl Reactivity in a Heme Peroxidase. JACS Au 2021, 1, 913–918. 10.1021/jacsau.1c00145. 34337604 PMC8317151

[ref141] Jiang H. K.; Wang Y. H.; Weng J. H.; Kurkute P.; Li C. L.; Lee M. N.; Chen P. J.; Tseng H. W.; Tsai M. D.; Wang Y. S. Probing the Active Site of Deubiquitinase Usp30 with Noncanonical Tryptophan Analogues. Biochem. 2020, 59, 2205–2209. 10.1021/acs.biochem.0c00307. 32484330

[ref142] Icking L. S.; Riedlberger A. M.; Krause F.; Widder J.; Frederiksen A. S.; Stockert F.; Spadt M.; Edel N.; Armbruster D.; Forlani G.; et al. Inclusive: A Database Collecting Useful Information on Non-Canonical Amino Acids and Their Incorporation into Proteins for Easier Genetic Code Expansion Implementation. Nucleic Acids Res. 2024, 52, D476–D482. 10.1093/nar/gkad1090. 37986218 PMC10767842

[ref143] Zhang M. S.; Brunner S. F.; Huguenin-Dezot N.; Liang A. D.; Schmied W. H.; Rogerson D. T.; Chin J. W. Biosynthesis and Genetic Encoding of Phosphothreonine through Parallel Selection and Deep Sequencing. Nat. Methods 2017, 14, 729–736. 10.1038/nmeth.4302. 28553966 PMC5493988

[ref144] Ding W.; Zhao H.; Chen Y.; Zhang B.; Yang Y.; Zang J.; Wu J.; Lin S. Chimeric Design of Pyrrolysyl-Trna Synthetase/Trna Pairs and Canonical Synthetase/Trna Pairs for Genetic Code Expansion. Nat. Commun. 2020, 11, 3154 10.1038/s41467-020-16898-y. 32572025 PMC7308279

[ref145] Zhao H.; Ding W.; Zang J.; Yang Y.; Liu C.; Hu L.; Chen Y.; Liu G.; Fang Y.; Yuan Y.; et al. Directed-Evolution of Translation System for Efficient Unnatural Amino Acids Incorporation and Generalizable Synthetic Auxotroph Construction. Nat. Commun. 2021, 12, 7039 10.1038/s41467-021-27399-x. 34857769 PMC8639764

[ref146] Zhu Y.; Ding W.; Chen Y.; Shan Y.; Liu C.; Fan X.; Lin S.; Chen P. R. Genetically Encoded Bioorthogonal Tryptophan Decaging in Living Cells. Nat. Chem. 2024, 16, 533 10.1038/s41557-024-01463-7. 38418535

[ref147] Cohen M.; Ozer E.; Kushmaro A.; Alfonta L. Cellular Localization of Cytochrome Bd in Cyanobacteria Using Genetic Code Expansion. Biotechnol. Bioeng. 2020, 117, 523–530. 10.1002/bit.27194. 31612992

[ref148] Scheidler C. M.; Vrabel M.; Schneider S. Genetic Code Expansion, Protein Expression, and Protein Functionalization in Bacillus Subtilis. ACS Synth. Biol. 2020, 9, 486–493. 10.1021/acssynbio.9b00458. 32053368

[ref149] Schmied W. H.; Elsasser S. J.; Uttamapinant C.; Chin J. W. Efficient Multisite Unnatural Amino Acid Incorporation in Mammalian Cells Via Optimized Pyrrolysyl Trna Synthetase/Trna Expression and Engineered Erf1. J. Am. Chem. Soc. 2014, 136, 15577–15583. 10.1021/ja5069728. 25350841 PMC4333590

[ref150] Hopper A. K.; Phizicky E. M. Trna Transfers to the Limelight. Genes Dev. 2003, 17, 162–180. 10.1101/gad.1049103. 12533506

[ref151] Hancock S. M.; Uprety R.; Deiters A.; Chin J. W. Expanding the Genetic Code of Yeast for Incorporation of Diverse Unnatural Amino Acids Via a Pyrrolysyl-Trna Synthetase/Trna Pair. J. Am. Chem. Soc. 2010, 132, 14819–14824. 10.1021/ja104609m. 20925334 PMC2956376

[ref152] Stieglitz J. T.; Lahiri P.; Stout M. I.; Van Deventer J. A. Exploration of Methanomethylophilus Alvus Pyrrolysyl-Trna Synthetase Activity in Yeast. ACS Synth. Biol. 2022, 11, 1824–1834. 10.1021/acssynbio.2c00001. 35417129 PMC10112046

[ref153] Gautier A.; Nguyen D. P.; Lusic H.; An W.; Deiters A.; Chin J. W. Genetically Encoded Photocontrol of Protein Localization in Mammalian Cells. J. Am. Chem. Soc. 2010, 132, 4086–4088. 10.1021/ja910688s. 20218600

[ref154] Meineke B.; Heimgartner J.; Lafranchi L.; Elsasser S. J. Methanomethylophilus Alvus Mx1201 Provides Basis for Mutual Orthogonal Pyrrolysyl Trna/Aminoacyl-Trna Synthetase Pairs in Mammalian Cells. ACS Chem. Biol. 2018, 13, 3087–3096. 10.1021/acschembio.8b00571. 30260624 PMC6243396

[ref155] Meineke B.; Heimgartner J.; Eirich J.; Landreh M.; Elsasser S. J. Site-Specific Incorporation of Two Ncaas for Two-Color Bioorthogonal Labeling and Crosslinking of Proteins on Live Mammalian Cells. Cell. Rep. 2020, 31, 107811 10.1016/j.celrep.2020.107811. 32579937

[ref156] Chen P. R.; Groff D.; Guo J.; Ou W.; Cellitti S.; Geierstanger B. H.; Schultz P. G. A Facile System for Encoding Unnatural Amino Acids in Mammalian Cells. Angew. Chem., Int. Ed. 2009, 48, 4052–4055. 10.1002/anie.200900683. PMC2873846 19378306

[ref157] Morgan C. W.; Dale I. L.; Thomas A. P.; Hunt J.; Chin J. W. Selective Craf Inhibition Elicits Transactivation. J. Am. Chem. Soc. 2021, 143, 4600–4606. 10.1021/jacs.0c11958. 33750116 PMC8041278

[ref158] Zhang Z.; Xu H.; Si L.; Chen Y.; Zhang B.; Wang Y.; Wu Y.; Zhou X.; Zhang L.; Zhou D. Construction of an Inducible Stable Cell Line for Efficient Incorporation of Unnatural Amino Acids in Mammalian Cells. Biochem. Biophys. Res. Commun. 2017, 489, 490–496. 10.1016/j.bbrc.2017.05.178. 28576486

[ref159] Roy G.; Reier J.; Garcia A.; Martin T.; Rice M.; Wang J.; Prophet M.; Christie R.; Dall’Acqua W.; Ahuja S.; et al. Development of a High Yielding Expression Platform for the Introduction of Non-Natural Amino Acids in Protein Sequences. mAbs. 2020, 12, 1684749 10.1080/19420862.2019.1684749. 31775561 PMC6927762

[ref160] Si L.; Xu H.; Zhou X.; Zhang Z.; Tian Z.; Wang Y.; Wu Y.; Zhang B.; Niu Z.; Zhang C.; et al. Generation of Influenza a Viruses as Live but Replication-Incompetent Virus Vaccines. Science 2016, 354, 1170–1173. 10.1126/science.aah5869. 27934767

[ref161] Lin S.; Yan H.; Li L.; Yang M.; Peng B.; Chen S.; Li W.; Chen P. R. Site-Specific Engineering of Chemical Functionalities on the Surface of Live Hepatitis D Virus. Angew. Chem., Int. Ed. 2013, 52, 13970–13974. 10.1002/anie.201305787. 24222614

[ref162] Wang T. Y.; Sang G. J.; Wang Q.; Leng C. L.; Tian Z. J.; Peng J. M.; Wang S. J.; Sun M. X.; Meng F. D.; Zheng H.; et al. Generation of Premature Termination Codon (Ptc)-Harboring Pseudorabies Virus (Prv) Via Genetic Code Expansion Technology. Viruses 2022, 14, 572 10.3390/v14030572. 35336979 PMC8950157

[ref163] Lu W.; Wang R.; Wang P.; Ma S.; Xia Q. Genetic Code Expansion System for Tight Control of Gene Expression in Bombyx Mori Cell Lines. Insects 2021, 12, 1081 10.3390/insects12121081. 34940169 PMC8709394

[ref164] van Husen L. S.; Katsori A. M.; Meineke B.; Tjernberg L. O.; Schedin-Weiss S.; Elsasser S. J. Engineered Human Induced Pluripotent Cells Enable Genetic Code Expansion in Brain Organoids. ChemBioChem. 2021, 22, 3208–3213. 10.1002/cbic.202100399. 34431592 PMC9290828

[ref165] Xi Z.; Davis L.; Baxter K.; Tynan A.; Goutou A.; Greiss S. Using a Quadruplet Codon to Expand the Genetic Code of an Animal. Nucleic Acids Res. 2022, 50, 4801–4812. 10.1093/nar/gkab1168. 34882769 PMC9122531

[ref166] Elliott T. S.; Townsley F. M.; Bianco A.; Ernst R. J.; Sachdeva A.; Elsässer S. J.; Davis L.; Lang K.; Pisa R.; Greiss S.; et al. Proteome Labeling and Protein Identification in Specific Tissues and at Specific Developmental Stages in an Animal. Nat. Biotechnol. 2014, 32, 465–472. 10.1038/nbt.2860. 24727715 PMC4107302

[ref167] Elliott T. S.; Bianco A.; Townsley F. M.; Fried S. D.; Chin J. W. Tagging and Enriching Proteins Enables Cell-Specific Proteomics. Cell Chem. Biol. 2016, 23, 805–815. 10.1016/j.chembiol.2016.05.018. 27447048 PMC4959846

[ref168] Chen C.; Yu G.; Huang Y.; Cheng W.; Li Y.; Sun Y.; Ye H.; Liu T. Genetic-Code-Expanded Cell-Based Therapy for Treating Diabetes in Mice. Nat. Chem. Biol. 2022, 18, 47–55. 10.1038/s41589-021-00899-z. 34782743

[ref169] Syed J.; Palani S.; Clarke S. T.; Asad Z.; Bottrill A. R.; Jones A. M. E.; Sampath K.; Balasubramanian M. K. Expanding the Zebrafish Genetic Code through Site-Specific Introduction of Azido-Lysine, Bicyclononyne-Lysine, and Diazirine-Lysine. Int. J. Mol. Sci. 2019, 20, 2577 10.3390/ijms20102577. 31130675 PMC6566996

[ref170] Johnson D. B.; Xu J.; Shen Z.; Takimoto J. K.; Schultz M. D.; Schmitz R. J.; Xiang Z.; Ecker J. R.; Briggs S. P.; Wang L. Rf1 Knockout Allows Ribosomal Incorporation of Unnatural Amino Acids at Multiple Sites. Nat. Chem. Biol. 2011, 7, 779–786. 10.1038/nchembio.657. 21926996 PMC3201715

[ref171] Mukai T.; Hayashi A.; Iraha F.; Sato A.; Ohtake K.; Yokoyama S.; Sakamoto K. Codon Reassignment in the Escherichia Coli Genetic Code. Nucleic Acids Res. 2010, 38, 8188–8195. 10.1093/nar/gkq707. 20702426 PMC3001078

[ref172] Wang K.; Neumann H.; Peak-Chew S. Y.; Chin J. W. Evolved Orthogonal Ribosomes Enhance the Efficiency of Synthetic Genetic Code Expansion. Nat. Biotechnol. 2007, 25, 770–777. 10.1038/nbt1314. 17592474

[ref173] Bartoschek M. D.; Ugur E.; Nguyen T. A.; Rodschinka G.; Wierer M.; Lang K.; Bultmann S. Identification of Permissive Amber Suppression Sites for Efficient Non-Canonical Amino Acid Incorporation in Mammalian Cells. Nucleic Acids Res. 2021, 49, e62 10.1093/nar/gkab132. 33684219 PMC8216290

[ref174] Bryson D. I.; Fan C.; Guo L. T.; Miller C.; Soll D.; Liu D. R. Continuous Directed Evolution of Aminoacyl-Trna Synthetases. Nat. Chem. Biol. 2017, 13, 1253–1260. 10.1038/nchembio.2474. 29035361 PMC5724969

[ref175] Williams T. L.; Iskandar D. J.; Nodling A. R.; Tan Y.; Luk L. Y. P.; Tsai Y. H. Transferability of N-Terminal Mutations of Pyrrolysyl-Trna Synthetase in One Species to That in Another Species on Unnatural Amino Acid Incorporation Efficiency. Amino Acids 2021, 53, 89–96. 10.1007/s00726-020-02927-z. 33331978 PMC7822784

[ref176] Sharma V.; Zeng Y.; Wang W. W.; Qiao Y.; Kurra Y.; Liu W. R. Evolving the N-Terminal Domain of Pyrrolysyl-Trna Synthetase for Improved Incorporation of Noncanonical Amino Acids. ChemBioChem. 2018, 19, 26–30. 10.1002/cbic.201700268. 29096043 PMC5989136

[ref177] Jiang H. K.; Lee M. N.; Tsou J. C.; Chang K. W.; Tseng H. W.; Chen K. P.; Li Y. K.; Wang Y. S. Linker and N-Terminal Domain Engineering of Pyrrolysyl-Trna Synthetase for Substrate Range Shifting and Activity Enhancement. Front. Bioeng. Biotechnol. 2020, 8, 235 10.3389/fbioe.2020.00235. 32322577 PMC7156790

[ref178] Koch N. G.; Baumann T.; Budisa N. Efficient Unnatural Protein Production by Pyrrolysyl-Trna Synthetase with Genetically Fused Solubility Tags. Front. Bioeng. Biotechnol. 2021, 9, 807438 10.3389/fbioe.2021.807438. 35284428 PMC8905625

[ref179] Cho C. C.; Blankenship L. R.; Ma X.; Xu S.; Liu W. The Pyrrolysyl-Trna Synthetase Activity Can Be Improved by a P188 Mutation That Stabilizes the Full-Length Enzyme. J. Mol. Biol. 2022, 434, 167453 10.1016/j.jmb.2022.167453. 35033561 PMC9018550

[ref180] Esvelt K. M.; Carlson J. C.; Liu D. R. A System for the Continuous Directed Evolution of Biomolecules. Nature 2011, 472, 499–503. 10.1038/nature09929. 21478873 PMC3084352

[ref181] Tian R.; Zhao R.; Guo H.; Yan K.; Wang C.; Lu C.; Lv X.; Li J.; Liu L.; Du G.; et al. Engineered Bacterial Orthogonal DNA Replication System for Continuous Evolution. Nat. Chem. Biol. 2023, 19, 1504–1512. 10.1038/s41589-023-01387-2. 37443393

[ref182] Tian R.; Rehm F. B. H.; Czernecki D.; Gu Y.; Zurcher J. F.; Liu K. C.; Chin J. W. Establishing a Synthetic Orthogonal Replication System Enables Accelerated Evolution in E. Coli. Science 2024, 383, 421–426. 10.1126/science.adk1281. 38271510

[ref183] Ravikumar A.; Arrieta A.; Liu C. C. An Orthogonal DNA Replication System in Yeast. Nat. Chem. Biol. 2014, 10, 175–177. 10.1038/nchembio.1439. 24487693

[ref184] Ravikumar A.; Arzumanyan G. A.; Obadi M. K. A.; Javanpour A. A.; Liu C. C. Scalable, Continuous Evolution of Genes at Mutation Rates above Genomic Error Thresholds. Cell 2018, 175, 1946–1957. 10.1016/j.cell.2018.10.021. 30415839 PMC6343851

[ref185] Molina R. S.; Rix G.; Mengiste A. A.; Alvarez B.; Seo D.; Chen H.; Hurtado J.; Zhang Q.; Garcia-Garcia J. D.; Heins Z. J.; et al. In Vivo Hypermutation and Continuous Evolution. Nat. Rev. Methods Primers 2022, 2, 36 10.1038/s43586-022-00119-5. PMC10108624 37073402

[ref186] Fan C.; Xiong H.; Reynolds N. M.; Soll D. Rationally Evolving Trnapyl for Efficient Incorporation of Noncanonical Amino Acids. Nucleic Acids Res. 2015, 43, e156 10.1093/nar/gkv800. 26250114 PMC4678846

[ref187] LaRiviere F. J.; Wolfson A. D.; Uhlenbeck O. C. Uniform Binding of Aminoacyl-Trnas to Elongation Factor Tu by Thermodynamic Compensation. Science 2001, 294, 165–168. 10.1126/science.1064242. 11588263

[ref188] Serfling R.; Lorenz C.; Etzel M.; Schicht G.; Bottke T.; Morl M.; Coin I. Designer Trnas for Efficient Incorporation of Non-Canonical Amino Acids by the Pyrrolysine System in Mammalian Cells. Nucleic Acids Res. 2018, 46, 1–10. 10.1093/nar/gkx1156. 29177436 PMC5758916

[ref189] Jewel D.; Kelemen R. E.; Huang R. L.; Zhu Z.; Sundaresh B.; Cao X.; Malley K.; Huang Z.; Pasha M.; Anthony J.; et al. Virus-Assisted Directed Evolution of Enhanced Suppressor Trnas in Mammalian Cells. Nat. Methods 2023, 20, 95–103. 10.1038/s41592-022-01706-w. 36550276 PMC9855281

[ref190] Jewel D.; Kelemen R. E.; Huang R. L.; Zhu Z.; Sundaresh B.; Malley K.; Pham Q.; Loynd C.; Huang Z.; van Opijnen T.; et al. Enhanced Directed Evolution in Mammalian Cells Yields a Hyperefficient Pyrrolysyl Trna for Noncanonical Amino Acid Mutagenesis. Angew. Chem., Int. Ed. 2024, 63, e202316428 10.1002/anie.202316428. PMC10922736 38279536

[ref191] Nikic I.; Estrada Girona G.; Kang J. H.; Paci G.; Mikhaleva S.; Koehler C.; Shymanska N. V.; Ventura Santos C.; Spitz D.; Lemke E. A. Debugging Eukaryotic Genetic Code Expansion for Site-Specific Click-Paint Super-Resolution Microscopy. Angew. Chem., Int. Ed. 2016, 55, 16172–16176. 10.1002/anie.201608284. PMC5215487 27804198

[ref192] Uttamapinant C.; Howe J. D.; Lang K.; Beránek V.; Davis L.; Mahesh M.; Barry N. P.; Chin J. W. Genetic Code Expansion Enables Live-Cell and Super-Resolution Imaging of Site-Specifically Labeled Cellular Proteins. J. Am. Chem. Soc. 2015, 137, 4602–4605. 10.1021/ja512838z. 25831022 PMC4506205

[ref193] Hu L.; Qin X.; Huang Y.; Cao W.; Wang C.; Wang Y.; Ling X.; Chen H.; Wu D.; Lin Y.; et al. Thermophilic Pyrrolysyl-Trna Synthetase Mutants for Enhanced Mammalian Genetic Code Expansion. ACS Synth. Biol. 2020, 9, 2723–2736. 10.1021/acssynbio.0c00257. 32931698

[ref194] Cohen S.; Arbely E. Single-Plasmid-Based System for Efficient Noncanonical Amino Acid Mutagenesis in Cultured Mammalian Cells. ChemBioChem. 2016, 17, 1008–1011. 10.1002/cbic.201500681. 27120490

[ref195] Kita A.; Hino N.; Higashi S.; Hirota K.; Narumi R.; Adachi J.; Takafuji K.; Ishimoto K.; Okada Y.; Sakamoto K.; et al. Adenovirus Vector-Based Incorporation of a Photo-Cross-Linkable Amino Acid into Proteins in Human Primary Cells and Cancerous Cell Lines. Sci. Rep. 2016, 6, 36946 10.1038/srep36946. 27833131 PMC5105139

[ref196] Shi N.; Tong L.; Lin H.; Zheng Z.; Zhang H.; Dong L.; Yang Y.; Shen Y.; Xia Q. Optimizing Erf1 to Enable the Genetic Encoding of Three Distinct Noncanonical Amino Acids in Mammalian Cells. Adv. Biol. 2022, 6, e2200092 10.1002/adbi.202200092. 35818694

[ref197] Lang K.; Davis L.; Wallace S.; Mahesh M.; Cox D. J.; Blackman M. L.; Fox J. M.; Chin J. W. Genetic Encoding of Bicyclononynes and Trans-Cyclooctenes for Site-Specific Protein Labeling in Vitro and in Live Mammalian Cells Via Rapid Fluorogenic Diels-Alder Reactions. J. Am. Chem. Soc. 2012, 134, 10317–10320. 10.1021/ja302832g. 22694658 PMC3687367

[ref198] Reinkemeier C. D.; Girona G. E.; Lemke E. A. Designer Membraneless Organelles Enable Codon Reassignment of Selected Mrnas in Eukaryotes. Science 2019, 363, eaaw2644 10.1126/science.aaw2644. 30923194 PMC7611745

[ref199] Reinkemeier C. D.; Lemke E. A. Dual Film-Like Organelles Enable Spatial Separation of Orthogonal Eukaryotic Translation. Cell 2021, 184, 4886–4903. 10.1016/j.cell.2021.08.001. 34433013 PMC8480389

[ref200] Chen Y.; Tang J.; Wang L.; Tian Z.; Cardenas A.; Fang X.; Chatterjee A.; Xiao H. Creation of Bacterial Cells with 5-Hydroxytryptophan as a 21(St) Amino Acid Building Block. Chem. 2020, 6, 2717–2727. 10.1016/j.chempr.2020.07.013. 33102928 PMC7583639

[ref201] Mehl R. A.; Anderson J. C.; Santoro S. W.; Wang L.; Martin A. B.; King D. S.; Horn D. M.; Schultz P. G. Generation of a Bacterium with a 21 Amino Acid Genetic Code. J. Am. Chem. Soc. 2003, 125, 935–939. 10.1021/ja0284153. 12537491

[ref202] Ou W.; Uno T.; Chiu H. P.; Grunewald J.; Cellitti S. E.; Crossgrove T.; Hao X.; Fan Q.; Quinn L. L.; Patterson P.; et al. Site-Specific Protein Modifications through Pyrroline-Carboxy-Lysine Residues. Proc. Natl. Acad. Sci. U. S. A. 2011, 108, 10437–10442. 10.1073/pnas.1105197108. 21670250 PMC3127931

[ref203] Ehrlich M.; Gattner M. J.; Viverge B.; Bretzler J.; Eisen D.; Stadlmeier M.; Vrabel M.; Carell T. Orchestrating the Biosynthesis of an Unnatural Pyrrolysine Amino Acid for Its Direct Incorporation into Proteins inside Living Cells. Chem. Eur. J. 2015, 21, 7701–7704. 10.1002/chem.201500971. 25845346

[ref204] Tai J.; Wang L.; Chan W. S.; Cheng J.; Chan Y. H.; Lee M. M.; Chan M. K. Pyrrolysine-Inspired in Cellulo Synthesis of an Unnatural Amino Acid for Facile Macrocyclization of Proteins. J. Am. Chem. Soc. 2023, 145, 10249–10258. 10.1021/jacs.3c01291. 37125745 PMC10176472

[ref205] Ho J. M. L.; Miller C. A.; Smith K. A.; Mattia J. R.; Bennett M. R. Improved Pyrrolysine Biosynthesis through Phage Assisted Non-Continuous Directed Evolution of the Complete Pathway. Nat. Commun. 2021, 12, 3914 10.1038/s41467-021-24183-9. 34168131 PMC8225853

[ref206] Exner M. P.; Kuenzl T.; To T. M.; Ouyang Z.; Schwagerus S.; Hoesl M. G.; Hackenberger C. P.; Lensen M. C.; Panke S.; Budisa N. Design of S-Allylcysteine in Situ Production and Incorporation Based on a Novel Pyrrolysyl-Trna Synthetase Variant. ChemBioChem. 2017, 18, 85–90. 10.1002/cbic.201600537. 27862817

[ref207] Lang K.; Chin J. W. Bioorthogonal Reactions for Labeling Proteins. ACS Chem. Biol. 2014, 9, 16–20. 10.1021/cb4009292. 24432752

[ref208] Lang K.; Chin J. W. Cellular Incorporation of Unnatural Amino Acids and Bioorthogonal Labeling of Proteins. Chem. Rev. 2014, 114, 4764–4806. 10.1021/cr400355w. 24655057

[ref209] Nguyen T. A.; Cigler M.; Lang K. Expanding the Genetic Code to Study Protein-Protein Interactions. Angew. Chem., Int. Ed. 2018, 57, 14350–14361. 10.1002/anie.201805869. 30144241

[ref210] Passioura T.; Suga H. Reprogramming the Genetic Code in Vitro. Trends Biochem. Sci. 2014, 39, 400–408. 10.1016/j.tibs.2014.07.005. 25129886

[ref211] Dawson P. E.; Muir T. W.; Clark-Lewis I.; Kent S. B. Synthesis of Proteins by Native Chemical Ligation. Science 1994, 266, 776–779. 10.1126/science.7973629. 7973629

[ref212] Yang A.; Cho K.; Park H. S. Chemical Biology Approaches for Studying Posttranslational Modifications. RNA Biol. 2018, 15, 427–440. 10.1080/15476286.2017.1360468. 28901832 PMC6103722

[ref213] Beranek V.; Reinkemeier C. D.; Zhang M. S.; Liang A. D.; Kym G.; Chin J. W. Genetically Encoded Protein Phosphorylation in Mammalian Cells. Cell Chem. Biol. 2018, 25, 1067–1074. 10.1016/j.chembiol.2018.05.013. 29937407 PMC6162345

[ref214] Italia J. S.; Peeler J. C.; Hillenbrand C. M.; Latour C.; Weerapana E.; Chatterjee A. Genetically Encoded Protein Sulfation in Mammalian Cells. Nat. Chem. Biol. 2020, 16, 379–382. 10.1038/s41589-020-0493-1. 32198493 PMC7564891

[ref215] Luo X.; Fu G.; Wang R. E.; Zhu X.; Zambaldo C.; Liu R.; Liu T.; Lyu X.; Du J.; Xuan W.; et al. Genetically Encoding Phosphotyrosine and Its Nonhydrolyzable Analog in Bacteria. Nat. Chem. Biol. 2017, 13, 845–849. 10.1038/nchembio.2405. 28604693 PMC5577365

[ref216] Fottner M.; Brunner A.-D.; Bittl V.; Horn-Ghetko D.; Jussupow A.; Kaila V. R. I.; Bremm A.; Lang K. Site-Specific Ubiquitylation and Sumoylation Using Genetic-Code Expansion and Sortase. Nat. Chem. Biol. 2019, 15, 276–284. 10.1038/s41589-019-0227-4. 30770915

[ref217] Virdee S.; Kapadnis P. B.; Elliott T.; Lang K.; Madrzak J.; Nguyen D. P.; Riechmann L.; Chin J. W. Traceless and Site-Specific Ubiquitination of Recombinant Proteins. J. Am. Chem. Soc. 2011, 133, 10708–10711. 10.1021/ja202799r. 21710965 PMC3135006

[ref218] Virdee S.; Ye Y.; Nguyen D. P.; Komander D.; Chin J. W. Engineered Diubiquitin Synthesis Reveals Lys29-Isopeptide Specificity of an Otu Deubiquitinase. Nat. Chem. Biol. 2010, 6, 750–757. 10.1038/nchembio.426. 20802491

[ref219] Zhang Z. J.; Pedicord V. A.; Peng T.; Hang H. C. Site-Specific Acylation of a Bacterial Virulence Regulator Attenuates Infection. Nat. Chem. Biol. 2020, 16, 95–103. 10.1038/s41589-019-0392-5. 31740807 PMC8439376

[ref220] Lammers M.; Neumann H.; Chin J. W.; James L. C. Acetylation Regulates Cyclophilin a Catalysis, Immunosuppression and Hiv Isomerization. Nat. Chem. Biol. 2010, 6, 331–337. 10.1038/nchembio.342. 20364129 PMC3867001

[ref221] Neumann H.; Hancock S. M.; Buning R.; Routh A.; Chapman L.; Somers J.; Owen-Hughes T.; Van Noort J.; Rhodes D.; Chin J. W. A Method for Genetically Installing Site-Specific Acetylation in Recombinant Histones Defines the Effects of H3 K56 Acetylation. Mol. Cell 2009, 36, 153–163. 10.1016/j.molcel.2009.07.027. 19818718 PMC2856916

[ref222] Arbely E.; Natan E.; Brandt T.; Allen M. D.; Veprintsev D. B.; Robinson C. V.; Chin J. W.; Joerger A. C.; Fersht A. R. Acetylation of Lysine 120 of P53 Endows DNA-Binding Specificity at Effective Physiological Salt Concentration. Proc. Natl. Acad. Sci. U. S. A. 2011, 108, 8251–8256. 10.1073/pnas.1105028108. 21525412 PMC3100949

[ref223] Kuhlmann N.; Wroblowski S.; Knyphausen P.; de Boor S.; Brenig J.; Zienert A. Y.; Meyer-Teschendorf K.; Praefcke G. J. K.; Nolte H.; Kruger M.; et al. Structural and Mechanistic Insights into the Regulation of the Fundamental Rho Regulator Rhogdialpha by Lysine Acetylation. J. Biol. Chem. 2016, 291, 5484–5499. 10.1074/jbc.M115.707091. 26719334 PMC4786691

[ref224] Kuhlmann N.; Wroblowski S.; Scislowski L.; Lammers M. Rhogdialpha Acetylation at K127 and K141 Affects Binding toward Nonprenylated Rhoa. Biochem. 2016, 55, 304–312. 10.1021/acs.biochem.5b01242. 26695096

[ref225] Knyphausen P.; de Boor S.; Kuhlmann N.; Scislowski L.; Extra A.; Baldus L.; Schacherl M.; Baumann U.; Neundorf I.; Lammers M. Insights into Lysine Deacetylation of Natively Folded Substrate Proteins by Sirtuins. J. Biol. Chem. 2016, 291, 14677–14694. 10.1074/jbc.M116.726307. 27226597 PMC4938187

[ref226] de Boor S.; Knyphausen P.; Kuhlmann N.; Wroblowski S.; Brenig J.; Scislowski L.; Baldus L.; Nolte H.; Kruger M.; Lammers M. Small Gtp-Binding Protein Ran Is Regulated by Posttranslational Lysine Acetylation. Proc. Natl. Acad. Sci. U. S. A. 2015, 112, E3679-3688 10.1073/pnas.1505995112. 26124124 PMC4507232

[ref227] Spinck M.; Ecke M.; Sievers S.; Neumann H. Highly Sensitive Lysine Deacetylase Assay Based on Acetylated Firefly Luciferase. Biochem. 2018, 57, 3552–3555. 10.1021/acs.biochem.8b00483. 29851343

[ref228] Neumann-Staubitz P.; Lammers M.; Neumann H. Genetic Code Expansion Tools to Study Lysine Acylation. Adv. Biol. 2021, 5, e2100926 10.1002/adbi.202100926. 34713630

[ref229] Ren J.; Sang Y.; Tan Y.; Tao J.; Ni J.; Liu S.; Fan X.; Zhao W.; Lu J.; Wu W.; et al. Acetylation of Lysine 201 Inhibits the DNA-Binding Ability of Phop to Regulate Salmonella Virulence. PLoS Pathog. 2016, 12, e1005458 10.1371/journal.ppat.1005458. 26943369 PMC4778762

[ref230] Qin R.; Sang Y.; Ren J.; Zhang Q.; Li S.; Cui Z.; Yao Y. F. The Bacterial Two-Hybrid System Uncovers the Involvement of Acetylation in Regulating of Lrp Activity in Salmonella Typhimurium. Front. Microbiol. 2016, 7, 1864 10.3389/fmicb.2016.01864. 27909434 PMC5112231

[ref231] Li S.; Zhang Q.; Xu Z.; Yao Y. F. Acetylation of Lysine 243 Inhibits the Oric Binding Ability of Dnaa in Escherichia Coli. Front. Microbiol. 2017, 8, 699 10.3389/fmicb.2017.00699. 28473824 PMC5397419

[ref232] Kirchgassner S.; Braun M. B.; Bartlick N.; Koc C.; Reinkemeier C. D.; Lemke E. A.; Stehle T.; Schwarzer D. Synthesis, Biochemical Characterization, and Genetic Encoding of a 1,2,4-Triazole Amino Acid as an Acetyllysine Mimic for Bromodomains of the Bet Family. Angew. Chem., Int. Ed. 2023, 62, e202215460 10.1002/anie.202215460. 36585954

[ref233] Venkat S.; Nannapaneni D. T.; Gregory C.; Gan Q.; McIntosh M.; Fan C. Genetically Encoding Thioacetyl-Lysine as a Non-Deacetylatable Analog of Lysine Acetylation in Escherichia Coli. FEBS Open Bio. 2017, 7, 1805–1814. 10.1002/2211-5463.12320. PMC5666399 29123988

[ref234] Huang Y.; Wan W.; Russell W. K.; Pai P. J.; Wang Z.; Russell D. H.; Liu W. Genetic Incorporation of an Aliphatic Keto-Containing Amino Acid into Proteins for Their Site-Specific Modifications. Bioorg. Med. Chem. Lett. 2010, 20, 878–880. 10.1016/j.bmcl.2009.12.077. 20074948

[ref235] Wang T.; Zhou Q.; Li F.; Yu Y.; Yin X.; Wang J. Genetic Incorporation of Nε-Formyllysine, a New Histone Post-Translational Modification. ChemBioChem. 2015, 16, 1440–1442. 10.1002/cbic.201500170. 25914338

[ref236] Wilkins B. J.; Hahn L. E.; Heitmuller S.; Frauendorf H.; Valerius O.; Braus G. H.; Neumann H. Genetically Encoding Lysine Modifications on Histone H4. ACS Chem. Biol. 2015, 10, 939–944. 10.1021/cb501011v. 25590375

[ref237] Gattner M. J.; Vrabel M.; Carell T. Synthesis of Epsilon-N-Propionyl-, Epsilon-N-Butyryl-, and Epsilon-N-Crotonyl-Lysine Containing Histone H3 Using the Pyrrolysine System. Chem. Commun. 2013, 49, 379–381. 10.1039/C2CC37836A. 23192406

[ref238] Xiao H.; Xuan W.; Shao S.; Liu T.; Schultz P. G. Genetic Incorporation of Epsilon-N-2-Hydroxyisobutyryl-Lysine into Recombinant Histones. ACS Chem. Biol. 2015, 10, 1599–1603. 10.1021/cb501055h. 25909834 PMC4506705

[ref239] Ren C.; Wu Q.; Xiao R.; Ji Y.; Yang X.; Zhang Z.; Qin H.; Ma J. A.; Xuan W. Expanding the Scope of Genetically Encoded Lysine Post-Translational Modifications with Lactylation, Beta-Hydroxybutyrylation and Lipoylation. ChemBioChem. 2022, 23, e202200302 10.1002/cbic.202200302. 35906721

[ref240] Kim C. H.; Kang M.; Kim H. J.; Chatterjee A.; Schultz P. G. Site-Specific Incorporation of Εn-Crotonyllysine into Histones. Angew. Chem., Int. Ed. 2012, 51, 7246–7249. 10.1002/anie.201203349. PMC3783207 22689270

[ref241] Cao L.; Liu J.; Ghelichkhani F.; Rozovsky S.; Wang L. Genetic Incorporation of ϵ-N-Benzoyllysine by Engineering Methanomethylophilus Alvus Pyrrolysyl-Trna Synthetase. ChemBioChem. 2021, 22, 2530–2534. 10.1002/cbic.202100218. 34118176 PMC8406699

[ref242] Tian H.; Yang J.; Guo A. D.; Ran Y.; Yang Y. Z.; Yang B.; Huang R.; Liu H.; Chen X. H. Genetically Encoded Benzoyllysines Serve as Versatile Probes for Interrogating Histone Benzoylation and Interactions in Living Cells. ACS Chem. Biol. 2021, 16, 2560–2569. 10.1021/acschembio.1c00614. 34618427

[ref243] Ji Y.; Ren C.; Miao H.; Pang Z.; Xiao R.; Yang X.; Xuan W. Genetically Encoding Ε-N-Benzoyllysine in Proteins. Chem. Commun. 2021, 57, 1798–1801. 10.1039/D0CC07954E. 33475635

[ref244] Zang J.; Chen Y.; Liu C.; Lin S. Probing the Role of Aurora Kinase a Threonylation with Site-Specific Lysine Threonylation. ACS Chem. Biol. 2023, 18, 674–678. 10.1021/acschembio.1c00682. 35230082

[ref245] Exner M. P.; Kohling S.; Rivollier J.; Gosling S.; Srivastava P.; Palyancheva Z. I.; Herdewijn P.; Heck M. P.; Rademann J.; Budisa N. Incorporation of Amino Acids with Long-Chain Terminal Olefins into Proteins. Molecules 2016, 21, 287 10.3390/molecules21030287. 26938510 PMC6272937

[ref246] Wright D. E.; Altaany Z.; Bi Y.; Alperstein Z.; O’Donoghue P. Acetylation Regulates Thioredoxin Reductase Oligomerization and Activity. Antioxid. Redox Signal 2018, 29, 377–388. 10.1089/ars.2017.7082. 29117711 PMC6025699

[ref247] Weyh M.; Jokisch M.-L.; Nguyen T.-A.; Fottner M.; Lang K. Deciphering Functional Roles of Protein Succinylation and Glutarylation Using Genetic Code Expansion. Nat. Chem. 2024, 16, 913–921. 10.1038/s41557-024-01500-5. 38531969 PMC11164685

[ref248] Wang Z. A.; Kurra Y.; Wang X.; Zeng Y.; Lee Y. J.; Sharma V.; Lin H.; Dai S. Y.; Liu W. R. A Versatile Approach for Site-Specific Lysine Acylation in Proteins. Angew. Chem., Int. Ed. 2017, 56, 1643–1647. 10.1002/anie.201611415. PMC5550319 28042700

[ref249] Neumann-Staubitz P.; Lammers M.; Neumann H. Genetic Code Expansion Tools to Study Lysine Acylation. Adv. Biol. 2021, 5, 2100926 10.1002/adbi.202100926. 34713630

[ref250] Nguyen D. P.; Garcia Alai M. M.; Kapadnis P. B.; Neumann H.; Chin J. W. Genetically Encoding N(Epsilon)-Methyl-L-Lysine in Recombinant Histones. J. Am. Chem. Soc. 2009, 131, 14194–14195. 10.1021/ja906603s. 19772323

[ref251] Ai H. W.; Lee J. W.; Schultz P. G. A Method to Site-Specifically Introduce Methyllysine into Proteins in E. Coli. Chem. Commun. 2010, 46, 5506–5508. 10.1039/c0cc00108b. PMC2928331 20571694

[ref252] Yanagisawa T.; Takahashi M.; Mukai T.; Sato S.; Wakamori M.; Shirouzu M.; Sakamoto K.; Umehara T.; Yokoyama S. Multiple Site-Specific Installations of Nepsilon-Monomethyl-L-Lysine into Histone Proteins by Cell-Based and Cell-Free Protein Synthesis. ChemBioChem. 2014, 15, 1830–1838. 10.1002/cbic.201402291. 25067793

[ref253] Rehn A.; Lawatscheck J.; Jokisch M.-L.; Mader S. L.; Luo Q.; Tippel F.; Blank B.; Richter K.; Lang K.; Kaila V. R. I.; A Methylated Lysine Is a Switch Point for Conformational Communication in the Chaperone Hsp90. Nat. Commun. 2020, 11, 10.1038/s41467-020-15048-8. PMC7057950 32139682

[ref254] Groff D.; Chen P. R.; Peters F. B.; Schultz P. G. A Genetically Encoded Epsilon-N-Methyl Lysine in Mammalian Cells. ChemBioChem. 2010, 11, 1066–1068. 10.1002/cbic.200900690. 20422671 PMC2882943

[ref255] Wang Y. S.; Wu B.; Wang Z.; Huang Y.; Wan W.; Russell W. K.; Pai P. J.; Moe Y. N.; Russell D. H.; Liu W. R. A Genetically Encoded Photocaged Nepsilon-Methyl-L-Lysine. Mol. Biosyst. 2010, 6, 1557–1560. 10.1039/c002155e. 20711534

[ref256] Nguyen D. P.; Garcia Alai M. M.; Virdee S.; Chin J. W. Genetically Directing Varepsilon-N, N-Dimethyl-L-Lysine in Recombinant Histones. Chem. Biol. 2010, 17, 1072–1076. 10.1016/j.chembiol.2010.07.013. 21035729

[ref257] Wang Z. A.; Zeng Y.; Kurra Y.; Wang X.; Tharp J. M.; Vatansever E. C.; Hsu W. W.; Dai S.; Fang X.; Liu W. R. A Genetically Encoded Allysine for the Synthesis of Proteins with Site-Specific Lysine Dimethylation. Angew. Chem., Int. Ed. 2017, 56, 212–216. 10.1002/anie.201609452. PMC5206893 27910233

[ref258] Mali S. M.; Singh S. K.; Eid E.; Brik A. Ubiquitin Signaling: Chemistry Comes to the Rescue. J. Am. Chem. Soc. 2017, 139, 4971–4986. 10.1021/jacs.7b00089. 28328208

[ref259] Trang V. H.; Valkevich E. M.; Minami S.; Chen Y. C.; Ge Y.; Strieter E. R. Nonenzymatic Polymerization of Ubiquitin: Single-Step Synthesis and Isolation of Discrete Ubiquitin Oligomers. Angew. Chem., Int. Ed. 2012, 51, 13085–13088. 10.1002/anie.201207171. PMC4083817 23161800

[ref260] Chen J.; Ai Y.; Wang J.; Haracska L.; Zhuang Z. Chemically Ubiquitylated Pcna as a Probe for Eukaryotic Translesion DNA Synthesis. Nat. Chem. Biol. 2010, 6, 270–272. 10.1038/nchembio.316. 20208521

[ref261] Weikart N. D.; Mootz H. D. Generation of Site-Specific and Enzymatically Stable Conjugates of Recombinant Proteins with Ubiquitin-Like Modifiers by the Cu(I)-Catalyzed Azide-Alkyne Cycloaddition. ChemBioChem. 2010, 11, 774–777. 10.1002/cbic.200900738. 20209558

[ref262] Eger S.; Scheffner M.; Marx A.; Rubini M. Synthesis of Defined Ubiquitin Dimers. J. Am. Chem. Soc. 2010, 132, 16337–16339. 10.1021/ja1072838. 21033666

[ref263] Li X.; Fekner T.; Ottesen J. J.; Chan M. K. A Pyrrolysine Analogue for Site-Specific Protein Ubiquitination. Angew. Chem., Int. Ed. 2009, 48, 9184–9187. 10.1002/anie.200904472. 19882608

[ref264] Stanley M.; Virdee S. Genetically Directed Production of Recombinant, Isosteric and Nonhydrolysable Ubiquitin Conjugates. ChemBioChem. 2016, 17, 1472–1480. 10.1002/cbic.201600138. 27197715 PMC5094518

[ref265] Fottner M.; Weyh M.; Gaussmann S.; Schwarz D.; Sattler M.; Lang K. A Modular Toolbox to Generate Complex Polymeric Ubiquitin Architectures Using Orthogonal Sortase Enzymes. Nat. Commun. 2021, 12, 6515 10.1038/s41467-021-26812-9. 34764289 PMC8585875

[ref266] Fottner M.; Heimgartner J.; Gantz M.; Muhlhofer R.; Nast-Kolb T.; Lang K. Site-Specific Protein Labeling and Generation of Defined Ubiquitin-Protein Conjugates Using an Asparaginyl Endopeptidase. J. Am. Chem. Soc. 2022, 144, 13118–13126. 10.1021/jacs.2c02191. 35850488 PMC9335880

[ref267] Ajish Kumar K. S.; Haj-Yahya M.; Olschewski D.; Lashuel H. A.; Brik A. Highly Efficient and Chemoselective Peptide Ubiquitylation. Angew. Chem., Int. Ed. 2009, 48, 8090–8094. 10.1002/anie.200902936. 19780082

[ref268] Wright T. H.; Bower B. J.; Chalker J. M.; Bernardes G. J.; Wiewiora R.; Ng W. L.; Raj R.; Faulkner S.; Vallee M. R.; Phanumartwiwath A.; et al. Posttranslational Mutagenesis: A Chemical Strategy for Exploring Protein Side-Chain Diversity. Science 2016, 354, 597–623. 10.1126/science.aag1465. 27708059

[ref269] Yang A.; Ha S.; Ahn J.; Kim R.; Kim S.; Lee Y.; Kim J.; Soll D.; Lee H. Y.; Park H. S. A Chemical Biology Route to Site-Specific Authentic Protein Modifications. Science 2016, 354, 623–626. 10.1126/science.aah4428. 27708052 PMC5135561

[ref270] Wang Z. U.; Wang Y. S.; Pai P. J.; Russell W. K.; Russell D. H.; Liu W. R. A Facile Method to Synthesize Histones with Posttranslational Modification Mimics. Biochem. 2012, 51, 5232–5234. 10.1021/bi300535a. 22697363 PMC3448024

[ref271] Machida T.; Lang K.; Xue L.; Chin J. W.; Winssinger N. Site-Specific Glycoconjugation of Protein Via Bioorthogonal Tetrazine Cycloaddition with a Genetically Encoded Trans-Cyclooctene or Bicyclononyne. Bioconjugate Chem. 2015, 26, 802–806. 10.1021/acs.bioconjchem.5b00101. PMC4673905 25897481

[ref272] Kaya E.; Gutsmiedl K.; Vrabel M.; Muller M.; Thumbs P.; Carell T. Synthesis of Threefold Glycosylated Proteins Using Click Chemistry and Genetically Encoded Unnatural Amino Acids. ChemBioChem. 2009, 10, 2858–2861. 10.1002/cbic.200900625. 19899090

[ref273] Baker A. S.; Deiters A. Optical Control of Protein Function through Unnatural Amino Acid Mutagenesis and Other Optogenetic Approaches. ACS Chem. Biol. 2014, 9, 1398–1407. 10.1021/cb500176x. 24819585

[ref274] Courtney T.; Deiters A. Recent Advances in the Optical Control of Protein Function through Genetic Code Expansion. Curr. Opin. Chem. Biol. 2018, 46, 99–107. 10.1016/j.cbpa.2018.07.011. 30056281 PMC7083171

[ref275] Wang J.; Wang X.; Fan X.; Chen P. R. Unleashing the Power of Bond Cleavage Chemistry in Living Systems. ACS Cent. Sci. 2021, 7, 929–943. 10.1021/acscentsci.1c00124. 34235254 PMC8227596

[ref276] Ling X.; Zuo Y.; Chen H.; Ji D.; Wang J.; Chang L.; Liu T. Genetic Encoding of a Photocaged Glutamate for Optical Control of Protein Functions. CCS Chemistry 2023, 5, 1301–1307. 10.31635/ccschem.023.202202471.

[ref277] Deiters A.; Groff D.; Ryu Y.; Xie J.; Schultz P. G. A Genetically Encoded Photocaged Tyrosine. Angew. Chem., Int. Ed. 2006, 45, 2728–2731. 10.1002/anie.200600264. 16548032

[ref278] Wu N.; Deiters A.; Cropp T. A.; King D.; Schultz P. G. A Genetically Encoded Photocaged Amino Acid. J. Am. Chem. Soc. 2004, 126, 14306–14307. 10.1021/ja040175z. 15521721

[ref279] Lemke E. A.; Summerer D.; Geierstanger B. H.; Brittain S. M.; Schultz P. G. Control of Protein Phosphorylation with a Genetically Encoded Photocaged Amino Acid. Nat. Chem. Biol. 2007, 3, 769–772. 10.1038/nchembio.2007.44. 17965709

[ref280] Luo J.; Uprety R.; Naro Y.; Chou C.; Nguyen D. P.; Chin J. W.; Deiters A. Genetically Encoded Optochemical Probes for Simultaneous Fluorescence Reporting and Light Activation of Protein Function with Two-Photon Excitation. J. Am. Chem. Soc. 2014, 136, 15551–15558. 10.1021/ja5055862. 25341086 PMC4333581

[ref281] Nguyen D. P.; Mahesh M.; Elsasser S. J.; Hancock S. M.; Uttamapinant C.; Chin J. W. Genetic Encoding of Photocaged Cysteine Allows Photoactivation of Tev Protease in Live Mammalian Cells. J. Am. Chem. Soc. 2014, 136, 2240–2243. 10.1021/ja412191m. 24479649 PMC4333589

[ref282] Uprety R.; Luo J.; Liu J.; Naro Y.; Samanta S.; Deiters A. Genetic Encoding of Caged Cysteine and Caged Homocysteine in Bacterial and Mammalian Cells. ChemBioChem. 2014, 15, 1793–1799. 10.1002/cbic.201400073. 24976145

[ref283] Zhang X.; Huang H.; Liu Y.; Wu Z.; Wang F.; Fan X.; Chen P. R.; Wang J. Optical Control of Protein Functions Via Genetically Encoded Photocaged Aspartic Acids. J. Am. Chem. Soc. 2023, 145, 19218–19224. 10.1021/jacs.3c03701. 37632461

[ref284] Cheung J. W.; Kinney W. D.; Wesalo J. S.; Reed M.; Nicholson E. M.; Deiters A.; Cropp T. A. Genetic Encoding of a Photocaged Histidine for Light-Control of Protein Activity. ChemBioChem. 2023, 24, e202200721 10.1002/cbic.202200721. 36642698 PMC10407765

[ref285] Gautier A.; Deiters A.; Chin J. W. Light-Activated Kinases Enable Temporal Dissection of Signaling Networks in Living Cells. J. Am. Chem. Soc. 2011, 133, 2124–2127. 10.1021/ja1109979. 21271704 PMC3048767

[ref286] Liaunardy-Jopeace A.; Murton B. L.; Mahesh M.; Chin J. W.; James J. R. Encoding Optical Control in Lck Kinase to Quantitatively Investigate Its Activity in Live Cells. Nat. Struct. Mol. Biol. 2017, 24, 1155–1163. 10.1038/nsmb.3492. 29083415 PMC5736103

[ref287] Hemphill J.; Chou C.; Chin J. W.; Deiters A. Genetically Encoded Light-Activated Transcription for Spatiotemporal Control of Gene Expression and Gene Silencing in Mammalian Cells. J. Am. Chem. Soc. 2013, 135, 13433–13439. 10.1021/ja4051026. 23931657 PMC4188981

[ref288] Luo J.; Kong M.; Liu L.; Samanta S.; Van Houten B.; Deiters A. Optical Control of DNA Helicase Function through Genetic Code Expansion. ChemBioChem. 2017, 18, 466–469. 10.1002/cbic.201600624. 28120472 PMC5516474

[ref289] Luo J.; Arbely E.; Zhang J.; Chou C.; Uprety R.; Chin J. W.; Deiters A. Genetically Encoded Optical Activation of DNA Recombination in Human Cells. Chem. Commun. 2016, 52, 8529–8532. 10.1039/C6CC03934K. PMC5048445 27277957

[ref290] Wang J.; Liu Y.; Liu Y.; Zheng S.; Wang X.; Zhao J.; Yang F.; Zhang G.; Wang C.; Chen P. R. Time-Resolved Protein Activation by Proximal Decaging in Living Systems. Nature 2019, 569, 509–513. 10.1038/s41586-019-1188-1. 31068699

[ref291] Welegedara A. P.; Adams L. A.; Huber T.; Graham B.; Otting G. Site-Specific Incorporation of Selenocysteine by Genetic Encoding as a Photocaged Unnatural Amino Acid. Bioconjugate Chem. 2018, 29, 2257–2264. 10.1021/acs.bioconjchem.8b00254. 29874064

[ref292] Li J.; Yu J.; Zhao J.; Wang J.; Zheng S.; Lin S.; Chen L.; Yang M.; Jia S.; Zhang X.; et al. Palladium-Triggered Deprotection Chemistry for Protein Activation in Living Cells. Nat. Chem. 2014, 6, 352–361. 10.1038/nchem.1887. 24651204

[ref293] Li J.; Jia S.; Chen P. R. Diels-Alder Reaction-Triggered Bioorthogonal Protein Decaging in Living Cells. Nat. Chem. Biol. 2014, 10, 1003–1005. 10.1038/nchembio.1656. 25362360

[ref294] Fan X.; Ge Y.; Lin F.; Yang Y.; Zhang G.; Ngai W. S.; Lin Z.; Zheng S.; Wang J.; Zhao J.; et al. Optimized Tetrazine Derivatives for Rapid Bioorthogonal Decaging in Living Cells. Angew. Chem., Int. Ed. 2016, 55, 14046–14050. 10.1002/anie.201608009. 27735133

[ref295] Carlson J. C. T.; Mikula H.; Weissleder R. Unraveling Tetrazine-Triggered Bioorthogonal Elimination Enables Chemical Tools for Ultrafast Release and Universal Cleavage. J. Am. Chem. Soc. 2018, 140, 3603–3612. 10.1021/jacs.7b11217. 29384666 PMC5857921

[ref296] Zhang G.; Li J.; Xie R.; Fan X.; Liu Y.; Zheng S.; Ge Y.; Chen P. R. Bioorthogonal Chemical Activation of Kinases in Living Systems. ACS Cent. Sci. 2016, 2, 325–331. 10.1021/acscentsci.6b00024. 27280167 PMC4882735

[ref297] Liu L.; Liu Y.; Zhang G.; Ge Y.; Fan X.; Lin F.; Wang J.; Zheng H.; Xie X.; Zeng X.; et al. Genetically Encoded Chemical Decaging in Living Bacteria. Biochem. 2018, 57, 446–450. 10.1021/acs.biochem.7b01017. 29171270

[ref298] Luo J.; Liu Q.; Morihiro K.; Deiters A. Small-Molecule Control of Protein Function through Staudinger Reduction. Nat. Chem. 2016, 8, 1027–1034. 10.1038/nchem.2573. 27768095 PMC5119652

[ref299] Ge Y.; Fan X.; Chen P. R. A Genetically Encoded Multifunctional Unnatural Amino Acid for Versatile Protein Manipulations in Living Cells. Chem. Sci. 2016, 7, 7055–7060. 10.1039/C6SC02615J. 28451140 PMC5355830

[ref300] Zhang Y.; Du Y.; Li M.; Zhang D.; Xiang Z.; Peng T. Activity-Based Genetically Encoded Fluorescent and Luminescent Probes for Detecting Formaldehyde in Living Cells. Angew. Chem., Int. Ed. 2020, 59, 16352–16356. 10.1002/anie.202001425. 32537908

[ref301] Wang X.; Liu Y.; Fan X.; Wang J.; Ngai W. S. C.; Zhang H.; Li J.; Zhang G.; Lin J.; Chen P. R. Copper-Triggered Bioorthogonal Cleavage Reactions for Reversible Protein and Cell Surface Modifications. J. Am. Chem. Soc. 2019, 141, 17133–17141. 10.1021/jacs.9b05833. 31580665

[ref302] Wang J.; Zheng S.; Liu Y.; Zhang Z.; Lin Z.; Li J.; Zhang G.; Wang X.; Li J.; Chen P. R. Palladium-Triggered Chemical Rescue of Intracellular Proteins Via Genetically Encoded Allene-Caged Tyrosine. J. Am. Chem. Soc. 2016, 138, 15118–15121. 10.1021/jacs.6b08933. 27797486

[ref303] Liu J.; Zheng F.; Cheng R.; Li S.; Rozovsky S.; Wang Q.; Wang L. Site-Specific Incorporation of Selenocysteine Using an Expanded Genetic Code and Palladium-Mediated Chemical Deprotection. J. Am. Chem. Soc. 2018, 140, 8807–8816. 10.1021/jacs.8b04603. 29984990 PMC6082430

[ref304] Reille-Seroussi M.; Mayer S. V.; Dörner W.; Lang K.; Mootz H. D. Expanding the Genetic Code with a Lysine Derivative Bearing an Enzymatically Removable Phenylacetyl Group. Chem. Commun. 2019, 55, 4793–4796. 10.1039/C9CC00475K. 30945708

[ref305] Reille-Seroussi M.; Meyer-Ahrens P.; Aust A.; Feldberg A. L.; Mootz H. D. Genetic Encoding and Enzymatic Deprotection of a Latent Thiol Side Chain to Enable New Protein Bioconjugation Applications. Angew. Chem. 2021, 133, 16108–16115. 10.1002/ange.202102343. PMC8361980 33844389

[ref306] Walsh S. J.; Bargh J. D.; Dannheim F. M.; Hanby A. R.; Seki H.; Counsell A. J.; Ou X.; Fowler E.; Ashman N.; Takada Y.; et al. Site-Selective Modification Strategies in Antibody-Drug Conjugates. Chem. Soc. Rev. 2021, 50, 1305–1353. 10.1039/D0CS00310G. 33290462

[ref307] Wen G.; Leen V.; Rohand T.; Sauer M.; Hofkens J. Current Progress in Expansion Microscopy: Chemical Strategies and Applications. Chem. Rev. 2023, 123, 3299–3323. 10.1021/acs.chemrev.2c00711. 36881995

[ref308] Rezhdo A.; Islam M.; Huang M.; Van Deventer J. A. Future Prospects for Noncanonical Amino Acids in Biological Therapeutics. Curr. Opin. Biotechnol. 2019, 60, 168–178. 10.1016/j.copbio.2019.02.020. 30974337 PMC6783319

[ref309] Nikic I.; Lemke E. A. Genetic Code Expansion Enabled Site-Specific Dual-Color Protein Labeling: Superresolution Microscopy and Beyond. Curr. Opin. Chem. Biol. 2015, 28, 164–173. 10.1016/j.cbpa.2015.07.021. 26302384

[ref310] Krauskopf K.; Lang K. Increasing the Chemical Space of Proteins in Living Cells Via Genetic Code Expansion. Curr. Opin. Chem. Biol. 2020, 58, 112–120. 10.1016/j.cbpa.2020.07.012. 32911429

[ref311] Lee S.; Kim J.; Koh M. Recent Advances in Fluorescence Imaging by Genetically Encoded Non-Canonical Amino Acids. J. Mol. Biol. 2022, 434, 167248 10.1016/j.jmb.2021.167248. 34547330

[ref312] Aphicho K.; Kittipanukul N.; Uttamapinant C. Visualizing the Complexity of Proteins in Living Cells with Genetic Code Expansion. Curr. Opin. Chem. Biol. 2022, 66, 102108 10.1016/j.cbpa.2021.102108. 35026612

[ref313] Scinto S. L.; Bilodeau D. A.; Hincapie R.; Lee W.; Nguyen S. S.; Xu M.; Am Ende C. W.; Finn M. G.; Lang K.; Lin Q.; Bioorthogonal Chemistry. Nat. Rev. Methods Primers 2021, 1, 10.1038/s43586-021-00028-z. PMC8469592 34585143

[ref314] Nguyen D. P.; Lusic H.; Neumann H.; Kapadnis P. B.; Deiters A.; Chin J. W. Genetic Encoding and Labeling of Aliphatic Azides and Alkynes in Recombinant Proteins Via a Pyrrolysyl-Trna Synthetase/Trna(Cua) Pair and Click Chemistry. J. Am. Chem. Soc. 2009, 131, 8720–8721. 10.1021/ja900553w. 19514718

[ref315] Fekner T.; Li X.; Lee M. M.; Chan M. K. A Pyrrolysine Analogue for Protein Click Chemistry. Angew. Chem., Int. Ed. 2009, 48, 1633–1635. 10.1002/anie.200805420. 19156778

[ref316] Li X.; Fekner T.; Chan M. K. N6-(2-(R)-Propargylglycyl)Lysine as a Clickable Pyrrolysine Mimic. Chem.-Asian J. 2010, 5, 1765–1769. 10.1002/asia.201000205. 20544791 PMC3517070

[ref317] Hao Z.; Song Y.; Lin S.; Yang M.; Liang Y.; Wang J.; Chen P. R. A Readily Synthesized Cyclic Pyrrolysine Analogue for Site-Specific Protein ″Click″ Labeling. Chem. Commun. 2011, 47, 4502–4504. 10.1039/c1cc00024a. 21387054

[ref318] Milles S.; Tyagi S.; Banterle N.; Koehler C.; Vandelinder V.; Plass T.; Neal A. P.; Lemke E. A. Click Strategies for Single-Molecule Protein Fluorescence. J. Am. Chem. Soc. 2012, 134, 5187–5195. 10.1021/ja210587q. 22356317

[ref319] Sapienza P. J.; Currie M. M.; Lancaster N. M.; Li K.; Aube J.; Goldfarb D.; Cloer E. W.; Major M. B.; Lee A. L. Visualizing an Allosteric Intermediate Using Cuaac Stabilization of an Nmr Mixed Labeled Dimer. ACS Chem. Biol. 2021, 16, 2766–2775. 10.1021/acschembio.1c00617. 34784173 PMC9141113

[ref320] Wang W. W.; Angulo-Ibanez M.; Lyu J.; Kurra Y.; Tong Z.; Wu B.; Zhang L.; Sharma V.; Zhou J.; Lin H.; et al. A Click Chemistry Approach Reveals the Chromatin-Dependent Histone H3k36 Deacylase Nature of Sirt7. J. Am. Chem. Soc. 2019, 141, 2462–2473. 10.1021/jacs.8b12083. 30653310 PMC6812484

[ref321] Yang M.; Jalloh A. S.; Wei W.; Zhao J.; Wu P.; Chen P. R. Biocompatible Click Chemistry Enabled Compartment-Specific Ph Measurement inside E. Coli. Nat. Commun. 2014, 5, 4981 10.1038/ncomms5981. 25236616 PMC4174402

[ref322] Meineke B.; Heimgartner J.; Craig A. J.; Landreh M.; Moodie L. W. K.; Elsasser S. J. A Genetically Encoded Picolyl Azide for Improved Live Cell Copper Click Labeling. Front. Chem. 2021, 9, 768535 10.3389/fchem.2021.768535. 34858945 PMC8632528

[ref323] Plass T.; Milles S.; Koehler C.; Szymanski J.; Mueller R.; Wiessler M.; Schultz C.; Lemke E. A. Amino Acids for Diels-Alder Reactions in Living Cells. Angew. Chem., Int. Ed. 2012, 51, 4166–4170. 10.1002/anie.201108231. 22473599

[ref324] Agard N. J.; Prescher J. A.; Bertozzi C. R. A Strain-Promoted [3 + 2] Azide-Alkyne Cycloaddition for Covalent Modification of Biomolecules in Living Systems. J. Am. Chem. Soc. 2004, 126, 15046–15047. 10.1021/ja044996f. 15547999

[ref325] Li J.; Lin S.; Wang J.; Jia S.; Yang M.; Hao Z.; Zhang X.; Chen P. R. Ligand-Free Palladium-Mediated Site-Specific Protein Labeling inside Gram-Negative Bacterial Pathogens. J. Am. Chem. Soc. 2013, 135, 7330–7338. 10.1021/ja402424j. 23641876

[ref326] Li N.; Ramil C. P.; Lim R. K.; Lin Q. A Genetically Encoded Alkyne Directs Palladium-Mediated Protein Labeling on Live Mammalian Cell Surface. ACS Chem. Biol. 2015, 10, 379–384. 10.1021/cb500649q. 25347611 PMC4340352

[ref327] Kwan T. T.; Boutureira O.; Frye E. C.; Walsh S. J.; Gupta M. K.; Wallace S.; Wu Y.; Zhang F.; Sore H. F.; Galloway W.; et al. Protein Modification Via Alkyne Hydrosilylation Using a Substoichiometric Amount of Ruthenium(Ii) Catalyst. Chem. Sci. 2017, 8, 3871–3878. 10.1039/C6SC05313K. 28966779 PMC5578368

[ref328] Zhao Q.; Guo G.; Zhu W.; Zhu L.; Da Y.; Han Y.; Xu H.; Wu S.; Cheng Y.; Zhou Y.; et al. Suzuki Cross-Coupling Reaction with Genetically Encoded Fluorosulfates for Fluorogenic Protein Labeling. Chem. Eur. J. 2020, 26, 15938–15943. 10.1002/chem.202002037. 32776653

[ref329] Li Y.; Pan M.; Li Y.; Huang Y.; Guo Q. Thiol-Yne Radical Reaction Mediated Site-Specific Protein Labeling Via Genetic Incorporation of an Alkynyl-L-Lysine Analogue. Org. Biomol. Chem. 2013, 11, 2624–2629. 10.1039/c3ob27116a. 23450369

[ref330] Li Y. M.; Yang M. Y.; Huang Y. C.; Song X. D.; Liu L.; Chen P. R. Genetically Encoded Alkenyl-Pyrrolysine Analogues for Thiol-Ene Reaction Mediated Site-Specific Protein Labeling. Chem. Sci. 2012, 3, 2766–2770. 10.1039/c2sc20433a.

[ref331] Torres-Kolbus J.; Chou C.; Liu J.; Deiters A. Synthesis of Non-Linear Protein Dimers through a Genetically Encoded Thiol-Ene Reaction. PLoS One 2014, 9, e105467 10.1371/journal.pone.0105467. 25181502 PMC4152134

[ref332] Lee M. M.; Fekner T.; Tang T. H.; Wang L.; Chan A. H.; Hsu P. H.; Au S. W.; Chan M. K. A Click-and-Release Pyrrolysine Analogue. ChemBioChem. 2013, 14, 805–808. 10.1002/cbic.201300124. 23589397

[ref333] Tuley A.; Lee Y. J.; Wu B.; Wang Z. U.; Liu W. R. A Genetically Encoded Aldehyde for Rapid Protein Labelling. Chem. Commun. 2014, 50, 7424–7426. 10.1039/C4CC02000F. PMC4062584 24756176

[ref334] Brabham R. L.; Spears R. J.; Walton J.; Tyagi S.; Lemke E. A.; Fascione M. A. Palladium-Unleashed Proteins: Gentle Aldehyde Decaging for Site-Selective Protein Modification. Chem. Commun. 2018, 54, 1501–1504. 10.1039/C7CC07740H. 29363688

[ref335] Row R. D.; Shih H. W.; Alexander A. T.; Mehl R. A.; Prescher J. A. Cyclopropenones for Metabolic Targeting and Sequential Bioorthogonal Labeling. J. Am. Chem. Soc. 2017, 139, 7370–7375. 10.1021/jacs.7b03010. 28478678

[ref336] Tomlin F. M.; Gordon C. G.; Han Y.; Wu T. S.; Sletten E. M.; Bertozzi C. R. Site-Specific Incorporation of Quadricyclane into a Protein and Photocleavage of the Quadricyclane Ligation Adduct. Bioorg. Med. Chem. 2018, 26, 5280–5290. 10.1016/j.bmc.2018.04.009. 29754834 PMC6170726

[ref337] Hohl A.; Mideksa Y. G.; Karan R.; Akal A.; Vogler M.; Groll M.; Rueping M.; Lang K.; Feige M. J.; Eppinger J. Genetically Encoded Biotin Analogues: Incorporation and Application in Bacterial and Mammalian Cells. ChemBioChem. 2019, 20, 1795–1798. 10.1002/cbic.201900015. 30900320

[ref338] Nguyen D. P.; Elliott T.; Holt M.; Muir T. W.; Chin J. W. Genetically Encoded 1,2-Aminothiols Facilitate Rapid and Site-Specific Protein Labeling Via a Bio-Orthogonal Cyanobenzothiazole Condensation. J. Am. Chem. Soc. 2011, 133, 11418–11421. 10.1021/ja203111c. 21736333

[ref339] Lang K.; Davis L.; Torres-Kolbus J.; Chou C.; Deiters A.; Chin J. W. Genetically Encoded Norbornene Directs Site-Specific Cellular Protein Labelling Via a Rapid Bioorthogonal Reaction. Nat. Chem. 2012, 4, 298–304. 10.1038/nchem.1250. 22437715 PMC3758886

[ref340] Kaya E.; Vrabel M.; Deiml C.; Prill S.; Fluxa V. S.; Carell T. A Genetically Encoded Norbornene Amino Acid for the Mild and Selective Modification of Proteins in a Copper-Free Click Reaction. Angew. Chem., Int. Ed. 2012, 51, 4466–4469. 10.1002/anie.201109252. 22438179

[ref341] Kurra Y.; Odoi K. A.; Lee Y. J.; Yang Y.; Lu T.; Wheeler S. E.; Torres-Kolbus J.; Deiters A.; Liu W. R. Two Rapid Catalyst-Free Click Reactions for in Vivo Protein Labeling of Genetically Encoded Strained Alkene/Alkyne Functionalities. Bioconjugate Chem. 2014, 25, 1730–1738. 10.1021/bc500361d. PMC4166034 25158039

[ref342] Yu Z.; Pan Y.; Wang Z.; Wang J.; Lin Q. Genetically Encoded Cyclopropene Directs Rapid, Photoclick-Chemistry-Mediated Protein Labeling in Mammalian Cells. Angew. Chem., Int. Ed. 2012, 51, 10600–10604. 10.1002/anie.201205352. PMC3517012 22997015

[ref343] Peng T.; Hang H. C. Site-Specific Bioorthogonal Labeling for Fluorescence Imaging of Intracellular Proteins in Living Cells. J. Am. Chem. Soc. 2016, 138, 14423–14433. 10.1021/jacs.6b08733. 27768298 PMC5100829

[ref344] Nikić I.; Plass T.; Schraidt O.; Szymański J.; Briggs J. A. G.; Schultz C.; Lemke E. A. Minimal Tags for Rapid Dual-Color Live-Cell Labeling and Super-Resolution Microscopy. Angew. Chem., Int. Ed. 2014, 53, 2245–2249. 10.1002/anie.201309847. 24474648

[ref345] Reinkemeier C. D.; Koehler C.; Sauter P. F.; Shymanska N. V.; Echalier C.; Rutkowska A.; Will D. W.; Schultz C.; Lemke E. A. Synthesis and Evaluation of Novel Ring-Strained Noncanonical Amino Acids for Residue-Specific Bioorthogonal Reactions in Living Cells. Chem. Eur. J. 2021, 27, 6094–6099. 10.1002/chem.202100322. 33577120 PMC8049044

[ref346] Kozma E.; Nikić I.; Varga B. R.; Aramburu I. V.; Kang J. H.; Fackler O. T.; Lemke E. A.; Kele P. Hydrophilic Trans-Cyclooctenylated Noncanonical Amino Acids for Fast Intracellular Protein Labeling. ChemBioChem. 2016, 17, 1518–1524. 10.1002/cbic.201600284. 27223658

[ref347] Hoffmann J. E.; Plass T.; Nikic I.; Aramburu I. V.; Koehler C.; Gillandt H.; Lemke E. A.; Schultz C. Highly Stable Trans-Cyclooctene Amino Acids for Live-Cell Labeling. Chem. Eur. J. 2015, 21, 12266–12270. 10.1002/chem.201501647. 26177861

[ref348] Lee Y. J.; Kurra Y.; Yang Y.; Torres-Kolbus J.; Deiters A.; Liu W. R. Genetically Encoded Unstrained Olefins for Live Cell Labeling with Tetrazine Dyes. Chem. Commun. 2014, 50, 13085–13088. 10.1039/C4CC06435F. PMC4184966 25224663

[ref349] Zhang J.; Yan S.; He Z.; Ding C.; Zhai T.; Chen Y.; Li H.; Yang G.; Zhou X.; Wang P. Small Unnatural Amino Acid Carried Raman Tag for Molecular Imaging of Genetically Targeted Proteins. J. Phys. Chem. Lett. 2018, 9, 4679–4685. 10.1021/acs.jpclett.8b01991. 30067370

[ref350] Liu K.; Enns B.; Evans B.; Wang N.; Shang X.; Sittiwong W.; Dussault P. H.; Guo J. A Genetically Encoded Cyclobutene Probe for Labelling of Live Cells. Chem. Commun. 2017, 53, 10604–10607. 10.1039/C7CC05580C. PMC5648060 28902227

[ref351] Chen Y.; Wu K. L.; Tang J.; Loredo A.; Clements J.; Pei J.; Peng Z.; Gupta R.; Fang X.; Xiao H. Addition of Isocyanide-Containing Amino Acids to the Genetic Code for Protein Labeling and Activation. ACS Chem. Biol. 2019, 14, 2793–2799. 10.1021/acschembio.9b00678. 31682403 PMC6925311

[ref352] Garst E. H.; Lee H.; Das T.; Bhattacharya S.; Percher A.; Wiewiora R.; Witte I. P.; Li Y.; Peng T.; Im W.; et al. Site-Specific Lipidation Enhances Ifitm3Membrane Interactions and Antiviral Activity. ACS Chem. Biol. 2021, 16, 844–856. 10.1021/acschembio.1c00013. 33887136 PMC9112659

[ref353] Lafranchi L.; Schlesinger D.; Kimler K. J.; Elsasser S. J. Universal Single-Residue Terminal Labels for Fluorescent Live Cell Imaging of Microproteins. J. Am. Chem. Soc. 2020, 142, 20080–20087. 10.1021/jacs.0c09574. 33175524

[ref354] Spence J. S.; He R.; Hoffmann H. H.; Das T.; Thinon E.; Rice C. M.; Peng T.; Chandran K.; Hang H. C. Ifitm3 Directly Engages and Shuttles Incoming Virus Particles to Lysosomes. Nat. Chem. Biol. 2019, 15, 259–268. 10.1038/s41589-018-0213-2. 30643282 PMC6466627

[ref355] MacNevin C. J.; Watanabe T.; Weitzman M.; Gulyani A.; Fuehrer S.; Pinkin N. K.; Tian X.; Liu F.; Jin J.; Hahn K. M. Membrane-Permeant, Environment-Sensitive Dyes Generate Biosensors within Living Cells. J. Am. Chem. Soc. 2019, 141, 7275–7282. 10.1021/jacs.8b09841. 30994345 PMC6572722

[ref356] Demeter O.; Kormos A.; Koehler C.; Mezo G.; Nemeth K.; Kozma E.; Takacs L. B.; Lemke E. A.; Kele P. Bisazide Cyanine Dyes as Fluorogenic Probes for Bis-Cyclooctynylated Peptide Tags and as Fluorogenic Cross-Linkers of Cyclooctynylated Proteins. Bioconjugate Chem. 2017, 28, 1552–1559. 10.1021/acs.bioconjchem.7b00178. 28441009

[ref357] Borrmann A.; Fatunsin O.; Dommerholt J.; Jonker A. M.; Lowik D. W.; van Hest J. C.; van Delft F. L. Strain-Promoted Oxidation-Controlled Cyclooctyne-1,2-Quinone Cycloaddition (Spocq) for Fast and Activatable Protein Conjugation. Bioconjugate Chem. 2015, 26, 257–261. 10.1021/bc500534d. 25521043

[ref358] Steiert F.; Schultz P.; Hofinger S.; Muller T. D.; Schwille P.; Weidemann T. Insights into Receptor Structure and Dynamics at the Surface of Living Cells. Nat. Commun. 2023, 14, 1596 10.1038/s41467-023-37284-4. 36949079 PMC10033668

[ref359] Stajkovic N.; Liu Y.; Arsic A.; Meng N.; Lyu H.; Zhang N.; Grimm D.; Lerche H.; Nikic-Spiegel I. Direct Fluorescent Labeling of Nf186 and Nav1.6 in Living Primary Neurons Using Bioorthogonal Click Chemistry. J. Cell Sci. 2023, 136, 10.1242/jcs.260600. PMC10323244 37288813

[ref360] Mattheisen J. M.; Wollowitz J. S.; Huber T.; Sakmar T. P. Genetic Code Expansion to Enable Site-Specific Bioorthogonal Labeling of Functional G Protein-Coupled Receptors in Live Cells. Protein Sci. 2023, 32, e4550 10.1002/pro.4550. 36540928 PMC9847076

[ref361] Arsic A.; Hagemann C.; Stajkovic N.; Schubert T.; Nikic-Spiegel I. Minimal Genetically Encoded Tags for Fluorescent Protein Labeling in Living Neurons. Nat. Commun. 2022, 13, 314 10.1038/s41467-022-27956-y. 35031604 PMC8760255

[ref362] Mihaila T. S.; Bate C.; Ostersehlt L. M.; Pape J. K.; Keller-Findeisen J.; Sahl S. J.; Hell S. W. Enhanced Incorporation of Subnanometer Tags into Cellular Proteins for Fluorescence Nanoscopy Via Optimized Genetic Code Expansion. Proc. Natl. Acad. Sci. U. S. A. 2022, 119, e2201861119 10.1073/pnas.2201861119. 35858298 PMC9304028

[ref363] Tian Y. L.; Fang M.; Lin Q. Intracellular Bioorthogonal Labeling of Glucagon Receptor Via Tetrazine Ligation. Biorg. Med. Chem. 2021, 43, 116256 10.1016/j.bmc.2021.116256. PMC8488902 34153838

[ref364] Sappakhaw K.; Jantarug K.; Slavoff S. A.; Israsena N.; Uttamapinant C. A Genetic Code Expansion-Derived Molecular Beacon for the Detection of Intracellular Amyloid-Β Peptide Generation. Angew. Chem. 2021, 133, 3980–3985. 10.1002/ange.202010703. PMC10946459 38504667

[ref365] Witte A.; Muñoz-López Á.; Metz M.; Schweiger M. R.; Janning P.; Summerer D. Encoded, Click-Reactive DNA-Binding Domains for Programmable Capture of Specific Chromatin Segments. Chem. Sci. 2020, 11, 12506–12511. 10.1039/D0SC02707C. 34123231 PMC8162481

[ref366] Hsieh H.-H.; Lee J. H.; Chandrasekar S.; Shan S.-o. A Ribosome-Associated Chaperone Enables Substrate Triage in a Cotranslational Protein Targeting Complex. Nat. Commun. 2020, 11, 5840 10.1038/s41467-020-19548-5. 33203865 PMC7673040

[ref367] Li Y.; Wang S.; Chen Y.; Li M.; Dong X.; Hang H. C.; Peng T. Site-Specific Chemical Fatty-Acylation for Gain-of-Function Analysis of Protein S-Palmitoylation in Live Cells. Chem. Commun. 2020, 56, 13880–13883. 10.1039/D0CC06073A. PMC8177487 33094750

[ref368] Mideksa Y. G.; Fottner M.; Braus S.; Weiß C. A. M.; Nguyen T. A.; Meier S.; Lang K.; Feige M. J. Site-Specific Protein Labeling with Fluorophores as a Tool to Monitor Protein Turnover. ChemBioChem. 2020, 21, 1861–1867. 10.1002/cbic.201900651. 32011787 PMC7383901

[ref369] Kugele A.; Silkenath B.; Langer J.; Wittmann V.; Drescher M. Protein Spin Labeling with a Photocaged Nitroxide Using Diels-Alder Chemistry. ChemBioChem. 2019, 20, 2479–2484. 10.1002/cbic.201900318. 31090999 PMC6790680

[ref370] Baumdick M.; Gelléri M.; Uttamapinant C.; Beránek V.; Chin J. W.; Bastiaens P. I. H. A Conformational Sensor Based on Genetic Code Expansion Reveals an Autocatalytic Component in Egfr Activation. Nat. Commun. 2018, 9, 3847 10.1038/s41467-018-06299-7. 30242154 PMC6155120

[ref371] Schvartz T.; Aloush N.; Goliand I.; Segal I.; Nachmias D.; Arbely E.; Elia N. Direct Fluorescent-Dye Labeling of Α-Tubulin in Mammalian Cells for Live Cell and Superresolution Imaging. Mol. Biol. Cell 2017, 28, 2747–2756. 10.1091/mbc.e17-03-0161. 28835375 PMC5638579

[ref372] Perdios L.; Lowe A. R.; Saladino G.; Bunney T. D.; Thiyagarajan N.; Alexandrov Y.; Dunsby C.; French P. M.; Chin J. W.; Gervasio F. L.; et al. Conformational Transition of Fgfr Kinase Activation Revealed by Site-Specific Unnatural Amino Acid Reporter and Single Molecule Fret. Sci. Rep. 2017, 7, 39841 10.1038/srep39841. 28045057 PMC5206623

[ref373] Baumdick M.; Brüggemann Y.; Schmick M.; Xouri G.; Sabet O.; Davis L.; Chin J. W.; Bastiaens P. I. H. Egf-Dependent Re-Routing of Vesicular Recycling Switches Spontaneous Phosphorylation Suppression to Egfr Signaling. eLife 2015, 4, 10.7554/eLife.12223. PMC4716840 26609808

[ref374] Tsai Y. H.; Essig S.; James J. R.; Lang K.; Chin J. W. Selective, Rapid and Optically Switchable Regulation of Protein Function in Live Mammalian Cells. Nat. Chem. 2015, 7, 554–561. 10.1038/nchem.2253. 26100803 PMC4673907

[ref375] Nikić I.; Kang J. H.; Girona G. E.; Aramburu I. V.; Lemke E. A. Labeling Proteins on Live Mammalian Cells Using Click Chemistry. Nat. Protoc. 2015, 10, 780–791. 10.1038/nprot.2015.045. 25906116

[ref376] Dommerholt J.; van Rooijen O.; Borrmann A.; Guerra C. F.; Bickelhaupt F. M.; van Delft F. L. Highly Accelerated Inverse Electron-Demand Cycloaddition of Electron-Deficient Azides with Aliphatic Cyclooctynes. Nat. Commun. 2014, 5, 5378 10.1038/ncomms6378. 25382411

[ref377] Zheng S.; Zhang G.; Li J.; Chen P. R. Monitoring Endocytic Trafficking of Anthrax Lethal Factor by Precise and Quantitative Protein Labeling. Angew. Chem. 2014, 126, 6567–6571. 10.1002/ange.201403945. 24828812

[ref378] Lukinavicius G.; Umezawa K.; Olivier N.; Honigmann A.; Yang G.; Plass T.; Mueller V.; Reymond L.; Correa I. R. Jr; Luo Z. G.; et al. A near-Infrared Fluorophore for Live-Cell Super-Resolution Microscopy of Cellular Proteins. Nat. Chem. 2013, 5, 132–139. 10.1038/nchem.1546. 23344448

[ref379] Oller-Salvia B.; Kym G.; Chin J. W. Rapid and Efficient Generation of Stable Antibody-Drug Conjugates Via an Encoded Cyclopropene and an Inverse-Electron-Demand Diels-Alder Reaction. Angew. Chem., Int. Ed. 2018, 57, 2831–2834. 10.1002/anie.201712370. PMC5861662 29356244

[ref380] Oller-Salvia B.; Chin J. W. Efficient Phage Display with Multiple Distinct Non-Canonical Amino Acids Using Orthogonal Ribosome-Mediated Genetic Code Expansion. Angew. Chem., Int. Ed. 2019, 58, 10844–10848. 10.1002/anie.201902658. PMC6771915 31157495

[ref381] Wallace S.; Chin J. W. Strain-Promoted Sydnone Bicyclo-[6.1.0]-Nonyne Cycloaddition. Chem. Sci. 2014, 5, 1742–1744. 10.1039/C3SC53332H. 25580211 PMC4285100

[ref382] Kormos A.; Koehler C.; Fodor E. A.; Rutkai Z. R.; Martin M. E.; Mezo G.; Lemke E. A.; Kele P. Bistetrazine-Cyanines as Double-Clicking Fluorogenic Two-Point Binder or Crosslinker Probes. Chem. Eur. J. 2018, 24, 8841–8847. 10.1002/chem.201800910. 29676491

[ref383] van Husen L. S.; Schedin-Weiss S.; Trung M. N.; Kazmi M. A.; Winblad B.; Sakmar T. P.; Elsässer S. J.; Tjernberg L. O. Dual Bioorthogonal Labeling of the Amyloid-Β Protein Precursor Facilitates Simultaneous Visualization of the Protein and Its Cleavage Products. JAD 2019, 72, 537–548. 10.3233/JAD-190898. 31609694 PMC6918917

[ref384] Mayer S. V.; Murnauer A.; von Wrisberg M. K.; Jokisch M. L.; Lang K. Photo-Induced and Rapid Labeling of Tetrazine-Bearing Proteins Via Cyclopropenone-Caged Bicyclononynes. Angew. Chem., Int. Ed. 2019, 58, 15876–15882. 10.1002/anie.201908209. PMC6856800 31476269

[ref385] Jang H. S.; Jana S.; Blizzard R. J.; Meeuwsen J. C.; Mehl R. A. Access to Faster Eukaryotic Cell Labeling with Encoded Tetrazine Amino Acids. J. Am. Chem. Soc. 2020, 142, 7245–7249. 10.1021/jacs.9b11520. 32251579 PMC7771912

[ref386] Seitchik J. L.; Peeler J. C.; Taylor M. T.; Blackman M. L.; Rhoads T. W.; Cooley R. B.; Refakis C.; Fox J. M.; Mehl R. A. Genetically Encoded Tetrazine Amino Acid Directs Rapid Site-Specific in Vivo Bioorthogonal Ligation with Trans-Cyclooctenes. J. Am. Chem. Soc. 2012, 134, 2898–2901. 10.1021/ja2109745. 22283158 PMC3369569

[ref387] Jana S.; Evans E. G. B.; Jang H. S.; Zhang S.; Zhang H.; Rajca A.; Gordon S. E.; Zagotta W. N.; Stoll S.; Mehl R. A. Ultrafast Bioorthogonal Spin-Labeling and Distance Measurements in Mammalian Cells Using Small, Genetically Encoded Tetrazine Amino Acids. J. Am. Chem. Soc. 2023, 145, 14608–14620. 10.1021/jacs.3c00967. 37364003 PMC10440187

[ref388] Yu Z.; Lin Q. Design of Spiro[2.3]Hex-1-Ene, a Genetically Encodable Double-Strained Alkene for Superfast Photoclick Chemistry. J. Am. Chem. Soc. 2014, 136, 4153–4156. 10.1021/ja5012542. 24592808 PMC3971965

[ref389] An P.; Wu H. Y.; Lewandowski T. M.; Lin Q. Hydrophilic Azaspiroalkenes as Robust Bioorthogonal Reporters. Chem. Commun. 2018, 54, 14005–14008. 10.1039/C8CC07432A. PMC6299827 30483687

[ref390] Xiong Q.; Zheng T.; Shen X.; Li B.; Fu J.; Zhao X.; Wang C.; Yu Z. Expanding the Functionality of Proteins with Genetically Encoded Dibenzo[B,F][1,4,5]Thiadiazepine: A Photo-Transducer for Photo-Click Decoration. Chem. Sci. 2022, 13, 3571–3581. 10.1039/D1SC05710C. 35432856 PMC8943893

[ref391] Sachdeva A.; Wang K.; Elliott T.; Chin J. W. Concerted, Rapid, Quantitative, and Site-Specific Dual Labeling of Proteins. J. Am. Chem. Soc. 2014, 136, 7785–7788. 10.1021/ja4129789. 24857040 PMC4333588

[ref392] Yang Y.; Lin S.; Lin W.; Chen P. R. Ligand-Assisted Dual-Site Click Labeling of Egfr on Living Cells. ChemBioChem. 2014, 15, 1738–1743. 10.1002/cbic.201400057. 24810988

[ref393] Kim S.; Ko W.; Sung B. H.; Kim S. C.; Lee H. S. Direct Protein-Protein Conjugation by Genetically Introducing Bioorthogonal Functional Groups into Proteins. Biorg. Med. Chem. 2016, 24, 5816–5822. 10.1016/j.bmc.2016.09.035. 27670101

[ref394] Quast R. B.; Fatemi F.; Kranendonk M.; Margeat E.; Truan G. Accurate Determination of Human Cpr Conformational Equilibrium by Smfret Using Dual Orthogonal Noncanonical Amino Acid Labeling. ChemBioChem. 2019, 20, 659–666. 10.1002/cbic.201800607. 30427570

[ref395] Osgood A. O.; Zheng Y.; Roy S. J. S.; Biris N.; Hussain M.; Loynd C.; Jewel D.; Italia J. S.; Chatterjee A. An Efficient Opal-Suppressor Tryptophanyl Pair Creates New Routes for Simultaneously Incorporating up to Three Distinct Noncanonical Amino Acids into Proteins in Mammalian Cells. Angew. Chem., Int. Ed. 2023, 62, e202219269 10.1002/anie.202219269. PMC10133189 36905325

[ref396] Saal K. A.; Richter F.; Rehling P.; Rizzoli S. O. Combined Use of Unnatural Amino Acids Enables Dual-Color Super-Resolution Imaging of Proteins Via Click Chemistry. ACS Nano 2018, 12, 12247–12254. 10.1021/acsnano.8b06047. 30525434

[ref397] Zheng Y.; Mukherjee R.; Chin M. A.; Igo P.; Gilgenast M. J.; Chatterjee A. Expanding the Scope of Single- and Double-Noncanonical Amino Acid Mutagenesis in Mammalian Cells Using Orthogonal Polyspecific Leucyl-Trna Synthetases. Biochem. 2018, 57, 441–445. 10.1021/acs.biochem.7b00952. 29106828 PMC6413323

[ref398] Lahiri P.; Martin M. S.; Lino B. R.; Scheck R. A.; Van Deventer J. A. Dual Noncanonical Amino Acid Incorporation Enabling Chemoselective Protein Modification at Two Distinct Sites in Yeast. Biochem. 2023, 62, 2098–2114. 10.1021/acs.biochem.2c00711. 37377426 PMC11146674

[ref399] Das D. K.; Govindan R.; Nikic-Spiegel I.; Krammer F.; Lemke E. A.; Munro J. B. Direct Visualization of the Conformational Dynamics of Single Influenza Hemagglutinin Trimers. Cell 2018, 174, 926–937. 10.1016/j.cell.2018.05.050. 29961575 PMC6086748

[ref400] Lu M.; Ma X.; Castillo-Menendez L. R.; Gorman J.; Alsahafi N.; Ermel U.; Terry D. S.; Chambers M.; Peng D.; Zhang B.; et al. Associating Hiv-1 Envelope Glycoprotein Structures with States on the Virus Observed by Smfret. Nature 2019, 568, 415–419. 10.1038/s41586-019-1101-y. 30971821 PMC6655592

[ref401] Xiao H.; Chatterjee A.; Choi S. H.; Bajjuri K. M.; Sinha S. C.; Schultz P. G. Genetic Incorporation of Multiple Unnatural Amino Acids into Proteins in Mammalian Cells. Angew. Chem., Int. Ed. 2013, 52, 14080–14083. 10.1002/anie.201308137. PMC11360239 24353230

[ref402] Zheng Y.; Addy P. S.; Mukherjee R.; Chatterjee A. Defining the Current Scope and Limitations of Dual Noncanonical Amino Acid Mutagenesis in Mammalian Cells. Chem. Sci. 2017, 8, 7211–7217. 10.1039/C7SC02560B. 29081953 PMC5633785

[ref403] Chaparro Sosa A. F.; Bednar R. M.; Mehl R. A.; Schwartz D. K.; Kaar J. L. Faster Surface Ligation Reactions Improve Immobilized Enzyme Structure and Activity. J. Am. Chem. Soc. 2021, 143, 7154–7163. 10.1021/jacs.1c02375. 33914511 PMC8574164

[ref404] Lammers C.; Hahn L. E.; Neumann H. Optimized Plasmid Systems for the Incorporation of Multiple Different Unnatural Amino Acids by Evolved Orthogonal Ribosomes. ChemBioChem. 2014, 15, 1800–1804. 10.1002/cbic.201402033. 24890611

[ref405] Wu B.; Wang Z.; Huang Y.; Liu W. R. Catalyst-Free and Site-Specific One-Pot Dual-Labeling of a Protein Directed by Two Genetically Incorporated Noncanonical Amino Acids. ChemBioChem. 2012, 13, 1405–1408. 10.1002/cbic.201200281. 22628069 PMC3389564

[ref406] Neumann H.; Wang K.; Davis L.; Garcia-Alai M.; Chin J. W. Encoding Multiple Unnatural Amino Acids Via Evolution of a Quadruplet-Decoding Ribosome. Nature 2010, 464, 441–444. 10.1038/nature08817. 20154731

[ref407] He D.; Xie X.; Yang F.; Zhang H.; Su H.; Ge Y.; Song H.; Chen P. R. Quantitative and Comparative Profiling of Protease Substrates through a Genetically Encoded Multifunctional Photocrosslinker. Angew. Chem., Int. Ed. 2017, 56, 14521–14525. 10.1002/anie.201708151. 28940571

[ref408] Yamaguchi A.; Matsuda T.; Ohtake K.; Yanagisawa T.; Yokoyama S.; Fujiwara Y.; Watanabe T.; Hohsaka T.; Sakamoto K. Incorporation of a Doubly Functionalized Synthetic Amino Acid into Proteins for Creating Chemical and Light-Induced Conjugates. Bioconjugate Chem. 2016, 27, 198–206. 10.1021/acs.bioconjchem.5b00602. 26625213

[ref409] Wang Y.; Zhang J.; Han B.; Tan L.; Cai W.; Li Y.; Su Y.; Yu Y.; Wang X.; Duan X.; et al. Noncanonical Amino Acids as Doubly Bio-Orthogonal Handles for One-Pot Preparation of Protein Multiconjugates. Nat. Commun. 2023, 14, 974 10.1038/s41467-023-36658-y. 36810592 PMC9944564

[ref410] Bednar R. M.; Karplus P. A.; Mehl R. A. Site-Specific Dual Encoding and Labeling of Proteins Via Genetic Code Expansion. Cell Chem. Biol. 2023, 30, 343–361. 10.1016/j.chembiol.2023.03.004. 36977415 PMC10764108

[ref411] Bose M.; Groff D.; Xie J.; Brustad E.; Schultz P. G. The Incorporation of a Photoisomerizable Amino Acid into Proteins in E. Coli. J. Am. Chem. Soc. 2006, 128, 388–389. 10.1021/ja055467u. 16402807

[ref412] Luo J.; Samanta S.; Convertino M.; Dokholyan N. V.; Deiters A. Reversible and Tunable Photoswitching of Protein Function through Genetic Encoding of Azobenzene Amino Acids in Mammalian Cells. ChemBioChem. 2018, 19, 2178–2185. 10.1002/cbic.201800226. 30277634 PMC6540996

[ref413] Hoppmann C.; Lacey V. K.; Louie G. V.; Wei J.; Noel J. P.; Wang L. Genetically Encoding Photoswitchable Click Amino Acids in Escherichia Coli and Mammalian Cells. Angew. Chem., Int. Ed. 2014, 53, 3932–3936. 10.1002/anie.201400001. PMC4051619 24615769

[ref414] John A. A.; Ramil C. P.; Tian Y.; Cheng G.; Lin Q. Synthesis and Site-Specific Incorporation of Red-Shifted Azobenzene Amino Acids into Proteins. Org. Lett. 2015, 17, 6258–6261. 10.1021/acs.orglett.5b03268. 26650435 PMC4685939

[ref415] Hoppmann C.; Maslennikov I.; Choe S.; Wang L. In Situ Formation of an Azo Bridge on Proteins Controllable by Visible Light. J. Am. Chem. Soc. 2015, 137, 11218–11221. 10.1021/jacs.5b06234. 26301538 PMC5356928

[ref416] Jones C. M.; Robkis D. M.; Blizzard R. J.; Munari M.; Venkatesh Y.; Mihaila T. S.; Eddins A. J.; Mehl R. A.; Zagotta W. N.; Gordon S. E.; et al. Genetic Encoding of a Highly Photostable, Long Lifetime Fluorescent Amino Acid for Imaging in Mammalian Cells. Chem. Sci. 2021, 12, 11955–11964. 10.1039/D1SC01914G. 34976337 PMC8634729

[ref417] Li M.; Wang F.; Yan L.; Lu M.; Zhang Y.; Peng T. Genetically Encoded Fluorescent Unnatural Amino Acids and Fret Probes for Detecting Deubiquitinase Activities. Chem. Commun. 2022, 58, 10186–10189. 10.1039/D2CC03623A. 36000311

[ref418] Zhang F.; Zhou Q.; Yang G.; An L.; Li F.; Wang J. A Genetically Encoded (19)F Nmr Probe for Lysine Acetylation. Chem. Commun. 2018, 54, 3879–3882. 10.1039/C7CC09825A. 29595201

[ref419] Abdelkader E. H.; Qianzhu H.; Tan Y. J.; Adams L. A.; Huber T.; Otting G. Genetic Encoding of N(6)-(((Trimethylsilyl)Methoxy)Carbonyl)-L-Lysine for Nmr Studies of Protein-Protein and Protein-Ligand Interactions. J. Am. Chem. Soc. 2021, 143, 1133–1143. 10.1021/jacs.0c11971. 33399460

[ref420] Schmidt M. J.; Fedoseev A.; Bucker D.; Borbas J.; Peter C.; Drescher M.; Summerer D. Epr Distance Measurements in Native Proteins with Genetically Encoded Spin Labels. ACS Chem. Biol. 2015, 10, 2764–2771. 10.1021/acschembio.5b00512. 26421438

[ref421] Wu X. Y.; Li M. Y.; Yang S. J.; Jiang J.; Ying Y. L.; Chen P. R.; Long Y. T. Controlled Genetic Encoding of Unnatural Amino Acids in a Protein Nanopore. Angew. Chem., Int. Ed. 2023, 62, e202300582 10.1002/anie.202300582. 37195576

[ref422] Yang Y.; Song H.; Chen P. R. Genetically Encoded Photocrosslinkers for Identifying and Mapping Protein-Protein Interactions in Living Cells. IUBMB Life 2016, 68, 879–886. 10.1002/iub.1560. 27670842

[ref423] Aydin Y.; Coin I. Genetically Encoded Crosslinkers to Address Protein-Protein Interactions. Protein Sci. 2023, 32, e4637 10.1002/pro.4637. 37027152 PMC10117390

[ref424] Coin I. Application of Non-Canonical Crosslinking Amino Acids to Study Protein-Protein Interactions in Live Cells. Curr. Opin. Chem. Biol. 2018, 46, 156–163. 10.1016/j.cbpa.2018.07.019. 30077876

[ref425] Sato S.; Mimasu S.; Sato A.; Hino N.; Sakamoto K.; Umehara T.; Yokoyama S. Crystallographic Study of a Site-Specifically Cross-Linked Protein Complex with a Genetically Incorporated Photoreactive Amino Acid. Biochem. 2011, 50, 250–257. 10.1021/bi1016183. 21128684

[ref426] Kashiwagi K.; Takahashi M.; Nishimoto M.; Hiyama T. B.; Higo T.; Umehara T.; Sakamoto K.; Ito T.; Yokoyama S. Crystal Structure of Eukaryotic Translation Initiation Factor 2b. Nature 2016, 531, 122–125. 10.1038/nature16991. 26901872

[ref427] Chin J. W.; Santoro S. W.; Martin A. B.; King D. S.; Wang L.; Schultz P. G. Addition of P-Azido-L-Phenylalanine to the Genetic Code of Escherichia Coli. J. Am. Chem. Soc. 2002, 124, 9026–9027. 10.1021/ja027007w. 12148987

[ref428] Chou C.; Uprety R.; Davis L.; Chin J. W.; Deiters A. Genetically Encoding an Aliphatic Diazirine for Protein Photocrosslinking. Chem. Sci. 2011, 2, 480–483. 10.1039/C0SC00373E.

[ref429] Yanagisawa T.; Hino N.; Iraha F.; Mukai T.; Sakamoto K.; Yokoyama S. Wide-Range Protein Photo-Crosslinking Achieved by a Genetically Encoded N(Epsilon)-(Benzyloxycarbonyl)Lysine Derivative with a Diazirinyl Moiety. Mol. Biosyst. 2012, 8, 1131–1135. 10.1039/c2mb05321g. 22294092

[ref430] Ai H. W.; Shen W.; Sagi A.; Chen P. R.; Schultz P. G. Probing Protein-Protein Interactions with a Genetically Encoded Photo-Crosslinking Amino Acid. ChemBioChem. 2011, 12, 1854–1857. 10.1002/cbic.201100194. 21678540

[ref431] Zhang M.; Lin S.; Song X.; Liu J.; Fu Y.; Ge X.; Fu X.; Chang Z.; Chen P. R. A Genetically Incorporated Crosslinker Reveals Chaperone Cooperation in Acid Resistance. Nat. Chem. Biol. 2011, 7, 671–677. 10.1038/nchembio.644. 21892184

[ref432] Braun N.; Friis S.; Ihling C.; Sinz A.; Andersen J.; Pless S. A. High-Throughput Characterization of Photocrosslinker-Bearing Ion Channel Variants to Map Residues Critical for Function and Pharmacology. PLoS Biol. 2021, 19, e3001321 10.1371/journal.pbio.3001321. 34491979 PMC8448361

[ref433] Koh M.; Ahmad I.; Ko Y.; Zhang Y.; Martinez T. F.; Diedrich J. K.; Chu Q.; Moresco J. J.; Erb M. A.; Saghatelian A.; A Short Orf-Encoded Transcriptional Regulator. Proc. Natl. Acad. Sci. U. S. A. 2021, 118, 10.1073/pnas.2021943118. PMC7848545 33468658

[ref434] Watson L.; Soliman T. N.; Davis K.; Kelly J.; Lockwood N.; Yang X.; Lynham S.; Scott J. D.; Crossland V.; McDonald N. Q.; et al. Co-Ordinated Control of the Aurora B Abscission Checkpoint by Pkcepsilon Complex Assembly, Midbody Recruitment and Retention. Biochem. J. 2021, 478, 2247–2263. 10.1042/BCJ20210283. 34143863 PMC8238520

[ref435] Wu X.; Spence J. S.; Das T.; Yuan X.; Chen C.; Zhang Y.; Li Y.; Sun Y.; Chandran K.; Hang H. C.; et al. Site-Specific Photo-Crosslinking Proteomics Reveal Regulation of Ifitm3 Trafficking and Turnover by Vcp/P97 Atpase. Cell Chem. Biol. 2020, 27, 571–585. e576 10.1016/j.chembiol.2020.03.004. 32243810 PMC7194980

[ref436] Pan D.; Walstein K.; Take A.; Bier D.; Kaiser N.; Musacchio A. Mechanism of Centromere Recruitment of the Cenp-a Chaperone Hjurp and Its Implications for Centromere Licensing. Nat. Commun. 2019, 10, 4046 10.1038/s41467-019-12019-6. 31492860 PMC6731319

[ref437] Martini S.; Davis K.; Faraway R.; Elze L.; Lockwood N.; Jones A.; Xie X.; McDonald N. Q.; Mann D. J.; Armstrong A.; et al. A Genetically-Encoded Crosslinker Screen Identifies Serbp1 as a Pkcepsilon Substrate Influencing Translation and Cell Division. Nat. Commun. 2021, 12, 6934 10.1038/s41467-021-27189-5. 34836941 PMC8626422

[ref438] Koch N. G.; Goettig P.; Rappsilber J.; Budisa N. Engineering Pyrrolysyl-Trna Synthetase for the Incorporation of Non-Canonical Amino Acids with Smaller Side Chains. Int. J. Mol. Sci. 2021, 22, 11194 10.3390/ijms222011194. 34681855 PMC8538471

[ref439] Tian Y.; Jacinto M. P.; Zeng Y.; Yu Z.; Qu J.; Liu W. R.; Lin Q. Genetically Encoded 2-Aryl-5-Carboxytetrazoles for Site-Selective Protein Photo-Cross-Linking. J. Am. Chem. Soc. 2017, 139, 6078–6081. 10.1021/jacs.7b02615. 28422494 PMC5423124

[ref440] Schmidt M. J.; Summerer D. Red-Light-Controlled Protein-Rna Crosslinking with a Genetically Encoded Furan. Angew. Chem., Int. Ed. 2013, 52, 4690–4693. 10.1002/anie.201300754. 23512703

[ref441] Hu W.; Yuan Y.; Wang C.-H.; Tian H.-T.; Guo A.-D.; Nie H.-J.; Hu H.; Tan M.; Tang Z.; Chen X.-H. Genetically Encoded Residue-Selective Photo-Crosslinker to Capture Protein-Protein Interactions in Living Cells. Chem. 2019, 5, 2955–2968. 10.1016/j.chempr.2019.08.020.

[ref442] Lin S.; He D.; Long T.; Zhang S.; Meng R.; Chen P. R. Genetically Encoded Cleavable Protein Photo-Cross-Linker. J. Am. Chem. Soc. 2014, 136, 11860–11863. 10.1021/ja504371w. 25084056

[ref443] Yang Y.; Song H.; He D.; Zhang S.; Dai S.; Lin S.; Meng R.; Wang C.; Chen P. R. Genetically Encoded Protein Photocrosslinker with a Transferable Mass Spectrometry-Identifiable Label. Nat. Commun. 2016, 7, 12299 10.1038/ncomms12299. 27460181 PMC4974458

[ref444] Yang Y.; Song H.; He D.; Zhang S.; Dai S.; Xie X.; Lin S.; Hao Z.; Zheng H.; Chen P. R. Genetically Encoded Releasable Photo-Cross-Linking Strategies for Studying Protein-Protein Interactions in Living Cells. Nat. Protoc. 2017, 12, 2147–2168. 10.1038/nprot.2017.090. 28933779

[ref445] Zhang S.; He D.; Lin Z.; Yang Y.; Song H.; Chen P. R. Conditional Chaperone-Client Interactions Revealed by Genetically Encoded Photo-Cross-Linkers. Acc. Chem. Res. 2017, 50, 1184–1192. 10.1021/acs.accounts.6b00647. 28467057

[ref446] Hoffmann J. E.; Dziuba D.; Stein F.; Schultz C. A Bifunctional Noncanonical Amino Acid: Synthesis, Expression, and Residue-Specific Proteome-Wide Incorporation. Biochem. 2018, 57, 4747–4752. 10.1021/acs.biochem.8b00397. 29932646

[ref447] Dziuba D.; Hoffmann J. E.; Hentze M. W.; Schultz C. A Genetically Encoded Diazirine Analogue for Rna-Protein Photo-Crosslinking. ChemBioChem. 2020, 21, 88–93. 10.1002/cbic.201900559. 31658407 PMC7003851

[ref448] Xie X.; Li X. M.; Qin F.; Lin J.; Zhang G.; Zhao J.; Bao X.; Zhu R.; Song H.; Li X. D.; et al. Genetically Encoded Photoaffinity Histone Marks. J. Am. Chem. Soc. 2017, 139, 6522–6525. 10.1021/jacs.7b01431. 28459554

[ref449] Zheng Y.; Gilgenast M. J.; Hauc S.; Chatterjee A. Capturing Post-Translational Modification-Triggered Protein-Protein Interactions Using Dual Noncanonical Amino Acid Mutagenesis. ACS Chem. Biol. 2018, 13, 1137–1141. 10.1021/acschembio.8b00021. 29544052 PMC6446081

[ref450] Bridge T.; Wegmann U.; Crack J. C.; Orman K.; Shaikh S. A.; Farndon W.; Martins C.; Saalbach G.; Sachdeva A. Site-Specific Encoding of Photoactivity and Photoreactivity into Antibody Fragments. Nat. Chem. Biol. 2023, 19, 740–749. 10.1038/s41589-022-01251-9. 36797401 PMC10229432

[ref451] Cao L.; Wang L. New Covalent Bonding Ability for Proteins. Protein Sci. 2022, 31, 312–322. 10.1002/pro.4228. 34761448 PMC8819847

[ref452] Furman J. L.; Kang M.; Choi S.; Cao Y.; Wold E. D.; Sun S. B.; Smider V. V.; Schultz P. G.; Kim C. H. A Genetically Encoded Aza-Michael Acceptor for Covalent Cross-Linking of Protein-Receptor Complexes. J. Am. Chem. Soc. 2014, 136, 8411–8417. 10.1021/ja502851h. 24846839 PMC4227728

[ref453] Li H.; Lv L.; Tang S.; Zang Y.; Wan T.; Wang D.; Cai L.; Ye H.; Tan R.; Wang N. Oxidation-Induced Protein Cross-Linking in Mammalian Cells. ACS Synth. Biol. 2023, 12, 984–992. 10.1021/acssynbio.3c00052. 37000479

[ref454] Shang X.; Chen Y.; Wang N.; Niu W.; Guo J. Oxidation-Induced Generation of a Mild Electrophile for Proximity-Enhanced Protein-Protein Crosslinking. Chem. Commun. 2018, 54, 4172–4175. 10.1039/C8CC01639A. PMC5908726 29629441

[ref455] Xuan W.; Shao S.; Schultz P. G. Protein Crosslinking by Genetically Encoded Noncanonical Amino Acids with Reactive Aryl Carbamate Side Chains. Angew. Chem., Int. Ed. 2017, 56, 5096–5100. 10.1002/anie.201611841. PMC5543777 28371162

[ref456] St Amant A. H.; Huang F.; Lin J.; Rickert K.; Oganesyan V.; Lemen D.; Mao S.; Harper J.; Marelli M.; Wu H.; et al. A Diene-Containing Noncanonical Amino Acid Enables Dual Functionality in Proteins: Rapid Diels-Alder Reaction with Maleimide or Proximity-Based Dimerization. Angew. Chem., Int. Ed. 2019, 58, 8489–8493. 10.1002/anie.201903494. PMC6555655 31018033

[ref457] Chen X. H.; Xiang Z.; Hu Y. S.; Lacey V. K.; Cang H.; Wang L. Genetically Encoding an Electrophilic Amino Acid for Protein Stapling and Covalent Binding to Native Receptors. ACS Chem. Biol. 2014, 9, 1956–1961. 10.1021/cb500453a. 25010185 PMC4168779

[ref458] Xiang Z.; Lacey V. K.; Ren H.; Xu J.; Burban D. J.; Jennings P. A.; Wang L. Proximity-Enabled Protein Crosslinking through Genetically Encoding Haloalkane Unnatural Amino Acids. Angew. Chem., Int. Ed. 2014, 53, 2190–2193. 10.1002/anie.201308794. PMC5356931 24449339

[ref459] Kobayashi T.; Hoppmann C.; Yang B.; Wang L. Using Protein-Confined Proximity to Determine Chemical Reactivity. J. Am. Chem. Soc. 2016, 138, 14832–14835. 10.1021/jacs.6b08656. 27797495 PMC5310709

[ref460] Cigler M.; Muller T. G.; Horn-Ghetko D.; von Wrisberg M. K.; Fottner M.; Goody R. S.; Itzen A.; Muller M. P.; Lang K. Proximity-Triggered Covalent Stabilization of Low-Affinity Protein Complexes in Vitro and in Vivo. Angew. Chem., Int. Ed. 2017, 56, 15737–15741. 10.1002/anie.201706927. 28960788

[ref461] Wang N.; Yang B.; Fu C.; Zhu H.; Zheng F.; Kobayashi T.; Liu J.; Li S.; Ma C.; Wang P. G.; et al. Genetically Encoding Fluorosulfate-L-Tyrosine to React with Lysine, Histidine, and Tyrosine Via Sufex in Proteins N Vivo. J. Am. Chem. Soc. 2018, 140, 4995–4999. 10.1021/jacs.8b01087. 29601199 PMC6031228

[ref462] Sun W.; Wang N.; Liu H.; Yu B.; Jin L.; Ren X.; Shen Y.; Wang L. Genetically Encoded Chemical Crosslinking of Rna in Vivo. Nat. Chem. 2023, 15, 21–32. 10.1038/s41557-022-01038-4. 36202986 PMC9840682

[ref463] Yang B.; Tang S.; Ma C.; Li S. T.; Shao G. C.; Dang B.; DeGrado W. F.; Dong M. Q.; Wang P. G.; Ding S.; et al. Spontaneous and Specific Chemical Cross-Linking in Live Cells to Capture and Identify Protein Interactions. Nat. Commun. 2017, 8, 2240 10.1038/s41467-017-02409-z. 29269770 PMC5740110

[ref464] Aydin Y.; Bottke T.; Lam J. H.; Ernicke S.; Fortmann A.; Tretbar M.; Zarzycka B.; Gurevich V. V.; Katritch V.; Coin I. Structural Details of a Class B Gpcr-Arrestin Complex Revealed by Genetically Encoded Crosslinkers in Living Cells. Nat. Commun. 2023, 14, 1151 10.1038/s41467-023-36797-2. 36859440 PMC9977954

[ref465] Bottke T.; Ernicke S.; Serfling R.; Ihling C.; Burda E.; Gurevich V. V.; Sinz A.; Coin I. Exploring Gpcr-Arrestin Interfaces with Genetically Encoded Crosslinkers. EMBO Rep. 2020, 21, e50437 10.15252/embr.202050437. 32929862 PMC7645262

[ref466] Liu C.; Wu T.; Shu X.; Li S. T.; Wang D. R.; Wang N.; Zhou R.; Yang H.; Jiang H.; Hendriks I. A.; et al. Identification of Protein Direct Interactome with Genetic Code Expansion and Search Engine Openuaa. Adv. Biol. 2021, 5, e2000308 10.1002/adbi.202000308. 33729691

[ref467] Tremel S.; Ohashi Y.; Morado D. R.; Bertram J.; Perisic O.; Brandt L. T. L.; Von Wrisberg M.-K.; Chen Z. A.; Maslen S. L.; Kovtun O.; et al. Structural Basis for Vps34 Kinase Activation by Rab1 and Rab5 on Membranes. Nat. Commun. 2021, 12, 1564 10.1038/s41467-021-21695-2. 33692360 PMC7946940

[ref468] Du J.; Wrisberg M. V.; Gulen B.; Stahl M.; Pett C.; Hedberg C.; Lang K.; Schneider S.; Itzen A. Rab1-Ampylation by Legionella Drra Is Allosterically Activated by Rab1. Nat. Commun. 2021, 12, 460 10.1038/s41467-020-20702-2. 33469029 PMC7815794

[ref469] Li Q.; Chen Q.; Klauser P. C.; Li M.; Zheng F.; Wang N.; Li X.; Zhang Q.; Fu X.; Wang Q.; et al. Developing Covalent Protein Drugs Via Proximity-Enabled Reactive Therapeutics. Cell 2020, 182, 85–97. 10.1016/j.cell.2020.05.028. 32579975

[ref470] Li S.; Wang N.; Yu B.; Sun W.; Wang L. Genetically Encoded Chemical Crosslinking of Carbohydrate. Nat. Chem. 2023, 15, 33–42. 10.1038/s41557-022-01059-z. 36216893 PMC9840686

[ref471] Huguenin-Dezot N.; Alonzo D. A.; Heberlig G. W.; Mahesh M.; Nguyen D. P.; Dornan M. H.; Boddy C. N.; Schmeing T. M.; Chin J. W. Trapping Biosynthetic Acyl-Enzyme Intermediates with Encoded 2,3-Diaminopropionic Acid. Nature 2019, 565, 112–117. 10.1038/s41586-018-0781-z. 30542153 PMC6436733

[ref472] Tang S.; Beattie A. T.; Kafkova L.; Petris G.; Huguenin-Dezot N.; Fiedler M.; Freeman M.; Chin J. W. Mechanism-Based Traps Enable Protease and Hydrolase Substrate Discovery. Nature 2022, 602, 701–707. 10.1038/s41586-022-04414-9. 35173328 PMC8866121

[ref473] Eiamthong B.; Meesawat P.; Wongsatit T.; Jitdee J.; Sangsri R.; Patchsung M.; Aphicho K.; Suraritdechachai S.; Huguenin-Dezot N.; Tang S.; et al. Discovery and Genetic Code Expansion of a Polyethylene Terephthalate (Pet) Hydrolase from the Human Saliva Metagenome for the Degradation and Bio-Functionalization of Pet. Angew. Chem., Int. Ed. 2022, 61, e202203061 10.1002/anie.202210188. PMC7613822 35656865

[ref474] Jackson J. C.; Duffy S. P.; Hess K. R.; Mehl R. A. Improving Nature’s Enzyme Active Site with Genetically Encoded Unnatural Amino Acids. J. Am. Chem. Soc. 2006, 128, 11124–11127. 10.1021/ja061099y. 16925430

[ref475] Pagar A. D.; Jeon H.; Khobragade T. P.; Sarak S.; Giri P.; Lim S.; Yoo T. H.; Ko B. J.; Yun H. Non-Canonical Amino Acid-Based Engineering of (R)-Amine Transaminase. Front. Chem. 2022, 10, 839636 10.3389/fchem.2022.839636. 35295971 PMC8918476

[ref476] Wilkinson H. C.; Dalby P. A. Fine-Tuning the Activity and Stability of an Evolved Enzyme Active-Site through Noncanonical Amino-Acids. Febs J. 2021, 288, 1935–1955. 10.1111/febs.15560. 32897608

[ref477] Sharma V.; Wang Y. S.; Liu W. R. Probing the Catalytic Charge-Relay System in Alanine Racemase with Genetically Encoded Histidine Mimetics. ACS Chem. Biol. 2016, 11, 3305–3309. 10.1021/acschembio.6b00940. 27978711 PMC5478170

[ref478] Seyedsayamdost M. R.; Xie J.; Chan C. T.; Schultz P. G.; Stubbe J. Site-Specific Insertion of 3-Aminotyrosine into Subunit Alpha2 of E. Coli Ribonucleotide Reductase: Direct Evidence for Involvement of Y730 and Y731 in Radical Propagation. J. Am. Chem. Soc. 2007, 129, 15060–15071. 10.1021/ja076043y. 17990884

[ref479] Parsons J. F.; Xiao G.; Gilliland G. L.; Armstrong R. N. Enzymes Harboring Unnatural Amino Acids: Mechanistic and Structural Analysis of the Enhanced Catalytic Activity of a Glutathione Transferase Containing 5-Fluorotryptophan. Biochem. 1998, 37, 6286–6294. 10.1021/bi980219e. 9572843

[ref480] Ugwumba I. N.; Ozawa K.; Xu Z. Q.; Ely F.; Foo J. L.; Herlt A. J.; Coppin C.; Brown S.; Taylor M. C.; Ollis D. L.; et al. Improving a Natural Enzyme Activity through Incorporation of Unnatural Amino Acids. J. Am. Chem. Soc. 2011, 133, 326–333. 10.1021/ja106416g. 21162578

[ref481] Yu Y.; Lv X.; Li J.; Zhou Q.; Cui C.; Hosseinzadeh P.; Mukherjee A.; Nilges M. J.; Wang J.; Lu Y. Defining the Role of Tyrosine and Rational Tuning of Oxidase Activity by Genetic Incorporation of Unnatural Tyrosine Analogs. J. Am. Chem. Soc. 2015, 137, 4594–4597. 10.1021/ja5109936. 25672571 PMC4676419

[ref482] Green A. P.; Hayashi T.; Mittl P. R. E.; Hilvert D. A Chemically Programmed Proximal Ligand Enhances the Catalytic Properties of a Heme Enzyme. J. Am. Chem. Soc. 2016, 138, 11344–11352. 10.1021/jacs.6b07029. 27500802

[ref483] Pott M.; Hayashi T.; Mori T.; Mittl P. R. E.; Green A. P.; Hilvert D. A Noncanonical Proximal Heme Ligand Affords an Efficient Peroxidase in a Globin Fold. J. Am. Chem. Soc. 2018, 140, 1535–1543. 10.1021/jacs.7b12621. 29309143

[ref484] Hayashi T.; Tinzl M.; Mori T.; Krengel U.; Proppe J.; Soetbeer J.; Klose D.; Jeschke G.; Reiher M.; Hilvert D. Capture and Characterization of a Reactive Haem-Carbenoid Complex in an Artificial Metalloenzyme. Nat. Catal. 2018, 1, 578–584. 10.1038/s41929-018-0105-6.

[ref485] Bjelic S.; Nivon L. G.; Celebi-Olcum N.; Kiss G.; Rosewall C. F.; Lovick H. M.; Ingalls E. L.; Gallaher J. L.; Seetharaman J.; Lew S.; et al. Computational Design of Enone-Binding Proteins with Catalytic Activity for the Morita-Baylis-Hillman Reaction. ACS Chem. Biol. 2013, 8, 749–757. 10.1021/cb3006227. 23330600 PMC3647451

[ref486] Burke A. J.; Lovelock S. L.; Frese A.; Crawshaw R.; Ortmayer M.; Dunstan M.; Levy C.; Green A. P. Design and Evolution of an Enzyme with a Non-Canonical Organocatalytic Mechanism. Nature 2019, 570, 219–223. 10.1038/s41586-019-1262-8. 31132786

[ref487] Maywood E. S.; Elliott T. S.; Patton A. P.; Krogager T. P.; Chesham J. E.; Ernst R. J.; Beranek V.; Brancaccio M.; Chin J. W.; Hastings M. H. Translational Switching of Cry1 Protein Expression Confers Reversible Control of Circadian Behavior in Arrhythmic Cry-Deficient Mice. Proc. Natl. Acad. Sci. U. S. A. 2018, 115, E12388-E12397 10.1073/pnas.1811438115. 30487216 PMC6310849

[ref488] Wang N.; Li Y.; Niu W.; Sun M.; Cerny R.; Li Q.; Guo J. Construction of a Live-Attenuated Hiv-1 Vaccine through Genetic Code Expansion. Angew. Chem., Int. Ed. 2014, 53, 4867–4871. 10.1002/anie.201402092. PMC4984542 24715496

[ref489] Chen Y.; Wan Y.; Wang N.; Yuan Z.; Niu W.; Li Q.; Guo J. Controlling the Replication of a Genomically Recoded Hiv-1 with a Functional Quadruplet Codon in Mammalian Cells. ACS Synth. Biol. 2018, 7, 1612–1617. 10.1021/acssynbio.8b00096. 29787233 PMC6003876

[ref490] Zheng Z.; Wu X.; Wang Y.; Yang X.; Chen H.; Shen Y.; Yang Y.; Xia Q. Attenuating Rna Viruses with Expanded Genetic Codes to Evoke Adjustable Immune Response in Pylrs-Trnacuapyl Transgenic Mice. Vaccines 2023, 11, 1606 10.3390/vaccines11101606. 37897007 PMC10610612

[ref491] McManus D.; Polidarova L.; Smyllie N. J.; Patton A. P.; Chesham J. E.; Maywood E. S.; Chin J. W.; Hastings M. H. Cryptochrome 1 as a State Variable of the Circadian Clockwork of the Suprachiasmatic Nucleus: Evidence from Translational Switching. Proc. Natl. Acad. Sci. U. S. A. 2022, 119, e2203563119 10.1073/pnas.2203563119. 35976881 PMC9407638

[ref492] Shi N.; Yang Q.; Zhang H.; Lu J.; Lin H.; Yang X.; Abulimiti A.; Cheng J.; Wang Y.; Tong L.; et al. Restoration of Dystrophin Expression in Mice by Suppressing a Nonsense Mutation through the Incorporation of Unnatural Amino Acids. Nat. Biomed. Eng. 2022, 6, 195–206. 10.1038/s41551-021-00774-1. 34341535

[ref493] Mahdavi A.; Segall-Shapiro T. H.; Kou S.; Jindal G. A.; Hoff K. G.; Liu S.; Chitsaz M.; Ismagilov R. F.; Silberg J. J.; Tirrell D. A. A Genetically Encoded and Gate for Cell-Targeted Metabolic Labeling of Proteins. J. Am. Chem. Soc. 2013, 135, 2979–2982. 10.1021/ja400448f. 23406315 PMC3620012

[ref494] Thomas E. E.; Pandey N.; Knudsen S.; Ball Z. T.; Silberg J. J. Programming Post-Translational Control over the Metabolic Labeling of Cellular Proteins with a Noncanonical Amino Acid. ACS Synth. Biol. 2017, 6, 1572–1583. 10.1021/acssynbio.7b00100. 28419802 PMC6858787

[ref495] Jiang H. K.; Ambrose N. L.; Chung C. Z.; Wang Y. S.; Soll D.; Tharp J. M. Split Aminoacyl-Trna Synthetases for Proximity-Induced Stop Codon Suppression. Proc. Natl. Acad. Sci. U. S. A. 2023, 120, e2219758120 10.1073/pnas.2219758120. 36787361 PMC9974479

[ref496] Fahnestock S.; Neumann H.; Shashoua V.; Rich A. Ribosome-Catalyzed Ester Formation. Biochem. 1970, 9, 2477–2483. 10.1021/bi00814a013. 4912484

[ref497] Li Y. M.; Yang M. Y.; Huang Y. C.; Li Y. T.; Chen P. R.; Liu L. Ligation of Expressed Protein Alpha-Hydrazides Via Genetic Incorporation of an Alpha-Hydroxy Acid. ACS Chem. Biol. 2012, 7, 1015–1022. 10.1021/cb300020s. 22424086

[ref498] Bindman N. A.; Bobeica S. C.; Liu W. R.; van der Donk W. A. Facile Removal of Leader Peptides from Lanthipeptides by Incorporation of a Hydroxy Acid. J. Am. Chem. Soc. 2015, 137, 6975–6978. 10.1021/jacs.5b04681. 26006047 PMC4505723

[ref499] Kobayashi T.; Yanagisawa T.; Sakamoto K.; Yokoyama S. Recognition of Non-Alpha-Amino Substrates by Pyrrolysyl-Trna Synthetase. J. Mol. Biol. 2009, 385, 1352–1360. 10.1016/j.jmb.2008.11.059. 19100747

[ref500] Ohtake K.; Mukai T.; Iraha F.; Takahashi M.; Haruna K. I.; Date M.; Yokoyama K.; Sakamoto K. Engineering an Automaturing Transglutaminase with Enhanced Thermostability by Genetic Code Expansion with Two Codon Reassignments. ACS Synth. Biol. 2018, 7, 2170–2176. 10.1021/acssynbio.8b00157. 30063837

[ref501] Yamaguchi A.; Iraha F.; Ohtake K.; Sakamoto K. Pyrrolysyl-Trna Synthetase with a Unique Architecture Enhances the Availability of Lysine Derivatives in Synthetic Genetic Codes. Molecules 2018, 23, 2460 10.3390/molecules23102460. 30261594 PMC6222415

[ref502] Guo J.; Wang J.; Anderson J. C.; Schultz P. G. Addition of an Alpha-Hydroxy Acid to the Genetic Code of Bacteria. Angew. Chem., Int. Ed. 2008, 47, 722–725. 10.1002/anie.200704074. 18069708

[ref503] Terasawa K.; Seike T.; Sakamoto K.; Ohtake K.; Terada T.; Iwata T.; Watabe T.; Yokoyama S.; Hara-Yokoyama M. Site-Specific Photo-Crosslinking/Cleavage for Protein-Protein Interface Identification Reveals Oligomeric Assembly of Lysosomal-Associated Membrane Protein Type 2a in Mammalian Cells. Protein Sci. 2023, 32, e4823 10.1002/pro.4823. 37906694 PMC10659947

[ref504] Kuroda T.; Huang Y.; Nishio S.; Goto Y.; Suga H. Post-Translational Backbone-Acyl Shift Yields Natural Product-Like Peptides Bearing Hydroxyhydrocarbon Units. Nat. Chem. 2022, 14, 1413–1420. 10.1038/s41557-022-01065-1. 36329180

[ref505] Sohma Y.; Kiso Y. Synthesis of O-Acyl Isopeptides. Chem. Rec. 2013, 13, 218–223. 10.1002/tcr.201200023. 23512813

[ref506] Dose C.; Seitz O. New Isocysteine Building Blocks and Chemoselective Peptide Ligation. Org. Biomol. Chem. 2004, 2, 59–65. 10.1039/b309235f. 14737660

[ref507] Fricke R.; Swenson C. V.; Roe L. T.; Hamlish N. X.; Shah B.; Zhang Z.; Ficaretta E.; Ad O.; Smaga S.; Gee C. L.; et al. Expanding the Substrate Scope of Pyrrolysyl-Transfer Rna Synthetase Enzymes to Include Non-Alpha-Amino Acids in Vitro and in Vivo. Nat. Chem. 2023, 15, 960–971. 10.1038/s41557-023-01224-y. 37264106 PMC10322718

[ref508] Hamlish N. X.; Abramyan A. M.; Shah B.; Zhang Z.; Schepartz A. Incorporation of Multiple Beta(2)-Hydroxy Acids into a Protein in Vivo Using an Orthogonal Aminoacyl-Trna Synthetase. ACS Cent. Sci. 2024, 10, 1044–1053. 10.1021/acscentsci.3c01366. 38799653 PMC11117724

[ref509] Melo Czekster C.; Robertson W. E.; Walker A. S.; Soll D.; Schepartz A. In Vivo Biosynthesis of a Beta-Amino Acid-Containing Protein. J. Am. Chem. Soc. 2016, 138, 5194–5197. 10.1021/jacs.6b01023. 27086674 PMC6640638

[ref510] Soni C.; Prywes N.; Hall M.; Nair M. A.; Savage D. F.; Schepartz A.; Chatterjee A. A Translation-Independent Directed Evolution Strategy to Engineer Aminoacyl-Trna Synthetases. ACS Central Science 2024, 10, 1211–1220. 10.1021/acscentsci.3c01557. 38947215 PMC11212135

[ref511] Dittmar K. A.; Sorensen M. A.; Elf J.; Ehrenberg M.; Pan T. Selective Charging of Trna Isoacceptors Induced by Amino-Acid Starvation. EMBO Rep. 2005, 6, 151–157. 10.1038/sj.embor.7400341. 15678157 PMC1299251

[ref512] Giege R.; Sissler M.; Florentz C. Universal Rules and Idiosyncratic Features in Trna Identity. Nucleic Acids Res. 1998, 26, 5017–5035. 10.1093/nar/26.22.5017. 9801296 PMC147952

[ref513] Zhang H.; Gong X.; Zhao Q.; Mukai T.; Vargas-Rodriguez O.; Zhang H.; Zhang Y.; Wassel P.; Amikura K.; Maupin-Furlow J.; et al. The Trna Discriminator Base Defines the Mutual Orthogonality of Two Distinct Pyrrolysyl-Trna Synthetase/Trnapyl Pairs in the Same Organism. Nucleic Acids Res. 2022, 50, 4601–4615. 10.1093/nar/gkac271. 35466371 PMC9071458

[ref514] Krahn N.; Zhang J.; Melnikov S. V.; Tharp J. M.; Villa A.; Patel A.; Howard R. J.; Gabir H.; Patel T. R.; Stetefeld J.; et al. tRNA Shape Is an Identity Element for an Archaeal Pyrrolysyl-Trna Synthetase from the Human Gut. Nucleic Acids Res. 2024, 52, 513 10.1093/nar/gkad1188. 38100361 PMC10810272

[ref515] Taylor C. J.; Hardy F. J.; Burke A. J.; Bednar R. M.; Mehl R. A.; Green A. P.; Lovelock S. L. Engineering Mutually Orthogonal Pylrs/Trna Pairs for Dual Encoding of Functional Histidine Analogues. Protein Sci. 2023, 32, e4640 10.1002/pro.4640. 37051694 PMC10127257

[ref516] Fischer J. T.; Soll D.; Tharp J. M. Directed Evolution of Methanomethylophilus Alvus Pyrrolysyl-Trna Synthetase Generates a Hyperactive and Highly Selective Variant. Front. Mol. Biosci. 2022, 9, 850613 10.3389/fmolb.2022.850613. 35372501 PMC8965510

[ref517] Avila-Crump S.; Hemshorn M. L.; Jones C. M.; Mbengi L.; Meyer K.; Griffis J. A.; Jana S.; Petrina G. E.; Pagar V. V.; Karplus P. A.; et al. Generating Efficient Methanomethylophilus Alvus Pyrrolysyl-Trna Synthetases for Structurally Diverse Non-Canonical Amino Acids. ACS Chem. Biol. 2022, 17, 3458–3469. 10.1021/acschembio.2c00639. 36383641 PMC9833845

[ref518] Dunkelmann D. L.; Oehm S. B.; Beattie A. T.; Chin J. W. A 68-Codon Genetic Code to Incorporate Four Distinct Non-Canonical Amino Acids Enabled by Automated Orthogonal Mrna Design. Nat. Chem. 2021, 13, 1110–1117. 10.1038/s41557-021-00764-5. 34426682 PMC7612796

[ref519] Ohtsuki T.; Manabe T.; Sisido M. Multiple Incorporation of Non-Natural Amino Acids into a Single Protein Using Trnas with Non-Standard Structures. FEBS Lett. 2005, 579, 6769–6774. 10.1016/j.febslet.2005.11.010. 16310775

[ref520] Taki M.; Tokuda Y.; Ohtsuki T.; Sisido M. Design of Carrier Trnas and Selection of Four-Base Codons for Efficient Incorporation of Various Nonnatural Amino Acids into Proteins in Spodoptera Frugiperda 21 (Sf21) Insect Cell-Free Translation System. J. Biosci. Bioeng. 2006, 102, 511–517. 10.1263/jbb.102.511. 17270715

[ref521] Mills E. M.; Barlow V. L.; Jones A. T.; Tsai Y. H. Development of Mammalian Cell Logic Gates Controlled by Unnatural Amino Acids. Cell Rep. Methods 2021, 1, 100073 10.1016/j.crmeth.2021.100073. 35474893 PMC9017196

[ref522] Mukai T.; Yamaguchi A.; Ohtake K.; Takahashi M.; Hayashi A.; Iraha F.; Kira S.; Yanagisawa T.; Yokoyama S.; Hoshi H.; et al. Reassignment of a Rare Sense Codon to a Non-Canonical Amino Acid in Escherichia Coli. Nucleic Acids Res. 2015, 43, 8111–8122. 10.1093/nar/gkv787. 26240376 PMC4652775

[ref523] Schwark D. G.; Schmitt M. A.; Fisk J. D. Directed Evolution of the Methanosarcina Barkeri Pyrrolysyl Trna/Aminoacyl Trna Synthetase Pair for Rapid Evaluation of Sense Codon Reassignment Potential. Int. J. Mol. Sci. 2021, 22, 895 10.3390/ijms22020895. 33477414 PMC7830368

[ref524] Ho J. M.; Reynolds N. M.; Rivera K.; Connolly M.; Guo L. T.; Ling J.; Pappin D. J.; Church G. M.; Soll D. Efficient Reassignment of a Frequent Serine Codon in Wild-Type Escherichia Coli. ACS Synth. Biol. 2016, 5, 163–171. 10.1021/acssynbio.5b00197. 26544153 PMC4807657

[ref525] Fredens J.; Wang K.; de la Torre D.; Funke L. F. H.; Robertson W. E.; Christova Y.; Chia T.; Schmied W. H.; Dunkelmann D. L.; Beranek V.; et al. Total Synthesis of Escherichia Coli with a Recoded Genome. Nature 2019, 569, 514–518. 10.1038/s41586-019-1192-5. 31092918 PMC7039709

[ref526] Dieterich D. C.; Lee J. J.; Link A. J.; Graumann J.; Tirrell D. A.; Schuman E. M. Labeling, Detection and Identification of Newly Synthesized Proteomes with Bioorthogonal Non-Canonical Amino-Acid Tagging. Nat. Protoc. 2007, 2, 532–540. 10.1038/nprot.2007.52. 17406607

[ref527] Alvarez-Castelao B.; Schanzenbacher C. T.; Hanus C.; Glock C.; Tom Dieck S.; Dorrbaum A. R.; Bartnik I.; Nassim-Assir B.; Ciirdaeva E.; Mueller A.; et al. Cell-Type-Specific Metabolic Labeling of Nascent Proteomes in Vivo. Nat. Biotechnol. 2017, 35, 1196–1201. 10.1038/nbt.4016. 29106408

[ref528] Wan W.; Huang Y.; Wang Z.; Russell W. K.; Pai P. J.; Russell D. H.; Liu W. R. A Facile System for Genetic Incorporation of Two Different Noncanonical Amino Acids into One Protein in Escherichia Coli. Angew. Chem., Int. Ed. 2010, 49, 3211–3214. 10.1002/anie.201000465. 20340150

[ref529] Italia J. S.; Addy P. S.; Erickson S. B.; Peeler J. C.; Weerapana E.; Chatterjee A. Mutually Orthogonal Nonsense-Suppression Systems and Conjugation Chemistries for Precise Protein Labeling at up to Three Distinct Sites. J. Am. Chem. Soc. 2019, 141, 6204–6212. 10.1021/jacs.8b12954. 30909694 PMC6500092

[ref530] Tharp J. M.; Vargas-Rodriguez O.; Schepartz A.; Soll D. Genetic Encoding of Three Distinct Noncanonical Amino Acids Using Reprogrammed Initiator and Nonsense Codons. ACS Chem. Biol. 2021, 16, 766–774. 10.1021/acschembio.1c00120. 33723984 PMC8336083

[ref531] Hintermann T.; Seebach D. The Biological Stability of Β-Peptides: No Interactions between Α- and Β-Peptidic Structures?. Chimia 1997, 51, 244–247. 10.2533/chimia.1997.244.

[ref532] Koksch B.; Sewald N.; Hofmann H.-J.; Burger K.; Jakubke H.-D. Proteolytically Stable Peptides by Incorporation of Α-Tfm Amino Acids. J. Pept. Sci. 1997, 3, 157–167. 10.1002/(SICI)1099-1387(199705)3:3<157::AID-PSC94>3.0.CO;2-W. 9230481

[ref533] Gellman S. H. Foldamers: A Manifesto. Acc. Chem. Res. 1998, 31, 173–180. 10.1021/ar960298r.

